# Photocatalytic construction of *N*-acyl-*N*,*O*-acetal-linked pyridines *via* aminocyclopropane ring opening

**DOI:** 10.1039/d5sc08055j

**Published:** 2025-12-09

**Authors:** Doyoung Kim, Eunseon Yang, Yoonhee Cho, Sungwoo Hong

**Affiliations:** a Department of Chemistry, Korea Advanced Institute of Science and Technology (KAIST) Daejeon 34141 Korea hongorg@kaist.ac.kr; b Center for Catalytic Hydrocarbon Functionalizations, Institute for Basic Science (IBS) Daejeon 34141 Korea

## Abstract

Pyridine and quinoline linkers are privileged motifs in medicinal chemistry, yet their site-selective installation into complex scaffolds remains challenging. Here, we report that aminocyclopropanes serve as precursors to *N*-acyl-*N*,*O*-acetal linkers installed onto pyridines *via* visible-light-driven ring opening under oxidant-free conditions. The ring-opened radical is captured by *N*-aminopyridinium salts to forge C(sp^3^)–C(aryl) bonds at the C4-selective site of the pyridine core, while the concomitantly released *N*-centered radical oxidizes the reduced photocatalyst, enabling efficient turnover. Subsequent nucleophile trapping furnishes *N*-acyl-*N*,*O*-acetals bearing pyridine or quinoline units with a broad scope across both heteroarenes and aminocyclopropanes, including late-stage diversification of complex molecules. Substituting methanol with TMSN_3_ provides azido-aminals, further expanding accessible architectures. The resulting *N*-acyl-*N*,*O*-acetal moieties function as versatile linchpins that engage diverse downstream manifolds, thereby enabling modular assembly and late-stage diversification of pyridine-containing targets.

## Abstract 2

Photocatalytic ring opening of aminocyclopropanes enables the selective installation of *N*-acyl-*N*,*O*-acetal linkers on pyridines, thereby facilitating diverse downstream functionalizations and efficient late-stage molecular editing.

## Graphical abstract

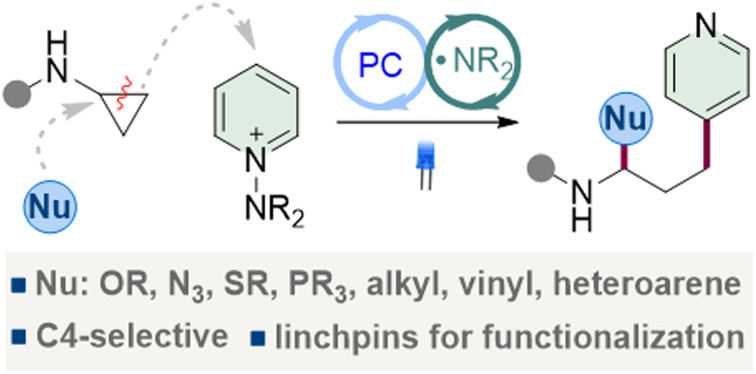

## Introduction

**Fig. 1:**
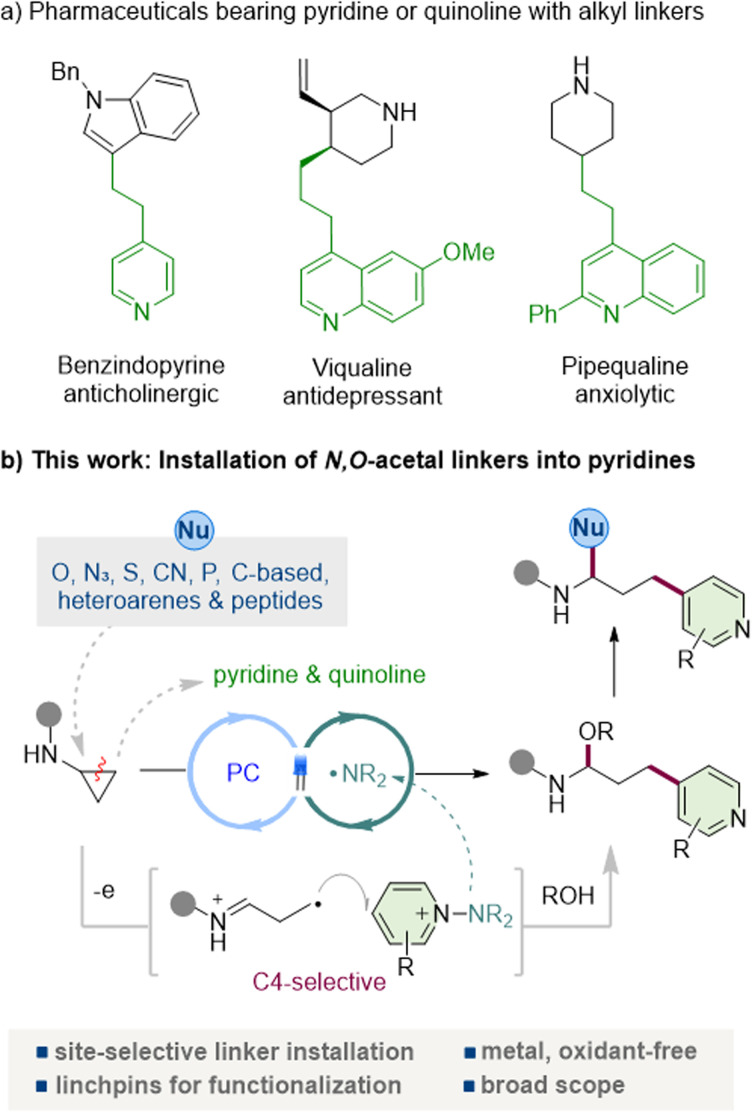
Photocatalytic construction of pyridyl-linked *N*-acyl-*N*,*O*-acetals *via* aminocyclopropane ring opening promoted by PC/amidyl radicals.

Pyridine and quinoline are among the most ubiquitous heteroarenes in pharmaceuticals, agrochemicals, ligands, and functional materials, reflecting their exceptional versatility and frequent occurrence in clinical candidates and approved drugs (Fig. 1a).^1^ Their tunable basicity, metabolic stability, and well-defined hydrogen-bond-accepting characteristics make them particularly attractive for pharmaceutical design, in which the incorporation of pyridine enables the introduction of solubilizing groups, photoaffinity tags, and heterobifunctional tethers with tailored functional properties.^2^

In parallel, *N*-acyl-*N*,*O*-acetals (and their hemiaminal congeners), which are frequently encountered as key motifs in bioactive and pharmaceutical molecules,^3^ represent bench-stable and readily diversifiable scaffolds that are attracting increasing attention.^4^ Nucleophile-induced substitution at the acetal carbon, acetal exchange, and controlled hydrolysis, oxidation, or reductive amination provide rapid entry to families of medicinally relevant building blocks. Owing to their facile activation under both Brønsted-acidic and metal-catalyzed conditions, these motifs function as reliable linchpins for fragment coupling, rearrangement, and heterofunctionalization.^5^ However, general and site-selective strategies that enable the direct installation of *N*,*O*-acetal linkers under mild conditions, and particularly those that can install them at the C4 position of pyridines and the corresponding site in quinolines, remain scarce. The C4 position offers an orthogonal exit vector from the pyridyl core, enabling bond formation in a distinct spatial direction and providing a well-defined platform for constructing bifunctional linkers, photoaffinity tags, and PROTAC-type heterobifunctional tethers.

Cyclopropanes have emerged as C3 synthons that combine operational stability with latent reactivity unmasked by ring opening.^6^ While the Lewis-acid and transition-metal activation of suitably polarized cyclopropanes is well established,^7^ substrates lacking strong electronic bias, such as cyclopropanes bearing only a single donor or single acceptor group, as well as arylcyclopropanes, often remain recalcitrant. Recently, photochemistry-driven single-electron transfer (SET) has emerged as a powerful strategy to achieve these demanding conversions.^8^ It has proven particularly effective in delivering ring-opening functionalizations under mild conditions.^9^ Among the cyclopropanes, aminocyclopropanes are particularly attractive: SET oxidation generates an amidyl radical that undergoes β-scission to give a distonic iminium radical primed for further functionalization.^10^ Despite advances in the radical chemistry of aminocyclopropanes, direct, C4-selective heteroarylation that can achieve both pyridyl- and quinolyl-linked *N*,*O*-acetal (or hemiaminal) frameworks has remained underexplored. The integration of C4-selective heteroaryl functionalization with concurrent *N*,*O*-acetal (or hemiaminal) formation would thus provide a powerful and modular platform for the synthesis of functional molecules and for late-stage diversification.

*N*-Aminopyridinium salts have recently emerged as versatile pyridine electrophiles that undergo radical coupling under mild, site-selective conditions.^11^ A key advantage is that N–N bond fragmentation releases a sulfonamidyl fragment that can act as an internal terminal oxidant, obviating external oxidants and enabling closed catalytic cycles.^12^ We reasoned that these attributes would permit efficient capture of ring-opened γ-radicals derived from aminocyclopropanes, forging C(sp^3^)–C(aryl) bonds with an intrinsic preference for addition at the pyridyl C4 site (and the corresponding quinoline position), thereby appending a functionalized linker in a single step. Here, we report a visible-light-driven coupling of aminocyclopropanes with *N*-aminopyridinium salts that directly installs *N*-acyl-*N*,*O*-acetal (hemiaminal) scaffolds into the pyridine or quinoline under neutral, oxidant-free conditions (Fig. 1b). The transformation displays broad functional-group tolerance across both heteroarenes and aminocyclopropanes, is operationally simple, and delivers excellent C4 regioselectivity *via* a SET-initiated β-scission/addition/rearomatization sequence.^11*d*,*f*^ Crucially, the resulting *N*-acyl-*N*,*O*-acetals serve as versatile synthetic linchpins that can engage diverse functionalization manifolds. The intact scaffold undergoes a variety of nucleophilic substitution reactions at the acetal carbon with organometallic reagents, indoles, thiols, cyanide, and phosphines, as well as Mukaiyama and Petasis-type reactions, underscoring its versatility for further functionalization. This strategy opens the way to structurally diverse pyridine-containing motifs that were previously difficult to obtain, broadening the accessible chemical space for pyridyl derivatives. It provides a modular late-stage route to 4-pyridyl-linked architectures, transforming aminocyclopropanes into general precursors that enable both structural remodeling and functional diversification.

## Results and discussion

To evaluate the feasibility of our design, we examined the model coupling between *N*-cyclopropyl-4-methoxybenzamide (1a) and *N*-aminopyridinium salt 2 in MeCN using the 3,6-di-*tert*-butyl-9-mesityl-10-phenylacridinium tetrafluoroborate as a photocatalyst (PC) and MeOH under blue-LED irradiation (Table 1). The initial reaction afforded the ring-opened γ-pyridyl *N*,*O*-acetal 3a in 65% yield with exclusive C4-selectivity (entry 1). Systematic variation of the reaction parameters identified temperature control as the key factor influencing performance; implementation of precise thermal regulation significantly improved consistency. Further thermal profiling revealed that mild cooling enhanced the yield, whereas heating led to diminished efficiency (entries 2 and 3). At −20 °C, the reaction rate decreased; however, prolonged irradiation at this temperature afforded 3a in 81% yield (entries 4 and 5). Screening of various photocatalysts confirmed that the original acridinium photocatalyst was optimal (entries 6–9, *E*_red_ = +2.08 V, 1.21 V, 0.66 V, 1.35 V *vs.* SCE, respectively),^13^ consistent with the need for a strong photooxidant to engage 1a. Examination of the aryl substituent on the pyridinium electrophile showed that both unsubstituted and CF_3_-substituted aryl groups maintained high C4-selectivity, with only a modest reduction in reactivity in the latter case (entries 10 and 11). In contrast, protonated or N–O-substituted salts (2d and 2e) exhibited reduced efficiency and diminished selectivity due to formation of the C2-substituted product, consistent with prior reports^11*j*^ (entries 12 and 13).

**Table 1:** Optimization of the reaction conditions^a^

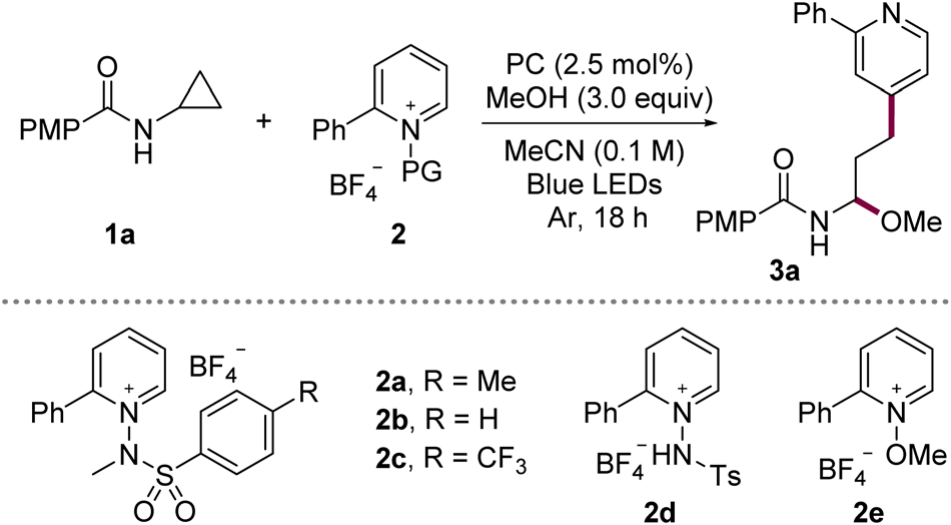				
Entry	PC	Salt	Temp.	Yield
1	[^*t*^Bu_2_-Mes-Ph-Acr]BF_4_	2a	25 °C	65%
2	[^*t*^Bu_2_-Mes-Ph-Acr]BF_4_	2a	40 °C	58%
3	[^*t*^Bu_2_-Mes-Ph-Acr]BF_4_	2a	10 °C	68%
4	[^*t*^Bu_2_-Mes-Ph-Acr]BF_4_	2a	−20 °C	34%
5	[^*t*^Bu_2_-Mes-Ph-Acr]BF_4_	2a	−20 °C	81%^b^ (80%)
6	[Mes-Acr]BF_4_	2a	−20 °C	69%^b^
7	[Ir{dF(CF_3_)ppy}_2_dtbbpy]PF_6_	2a	−20 °C	48%^b^^,^^c^
8	[Ir(ppy)_2_dtbbpy]PF_6_	2a	−20 °C	11%^b^^,^^c^
9	4CzIPN	2a	−20 °C	30%^b^
10	[^*t*^Bu_2_-Mes-Ph-Acr]BF_4_	2b	−20 °C	78%^b^
11	[^*t*^Bu_2_-Mes-Ph-Acr]BF_4_	2c	−20 °C	68%^b^
12	[^*t*^Bu_2_-Mes-Ph-Acr]BF_4_	2d	−20 °C	41%^b^ (1 : 1)
13	[^*t*^Bu_2_-Mes-Ph-Acr]BF_4_	2e	−20 °C	31%^b^ (1.4 : 1)

a Reaction conditions: 1a (0.05 mmol), 2 (1.5 equiv.), PC (2.5 mol%), MeOH (3.0 equiv.) in MeCN (0.5 mL) under irradiation with 440 nm LEDs (10 W) at 25 °C for 18 h under argon. Yields determined by ^1^H NMR spectroscopy using caffeine as an internal standard. Isolated yield and regioisomeric ratio (C4 *vs.* C2) are given in parentheses. PMP = *p*-methoxy phenyl.

b Reaction time: 40 h.

c 1 mol% of PC used.

With the optimized conditions in hand, we investigated the scope of the reaction to determine its generality and late-stage utility (Tables 2 and 3). Substitution at C2 of the pyridinium electrophile was broadly tolerated across electronic regimes, furnishing good yields for both electron-donating and electron-withdrawing groups (3a–3c). Phenyl-ring substitution proved productive, with methoxy- and trifluoromethyl-substituted 3d and 3e delivering high yields and bipyridyl-substituted 3f remaining competent. C3-substituted salts, including halide and ester variants, reacted smoothly (3g–3i). Likewise, disubstituted pyridines underwent the transformation with comparable efficiency (3j–3l). Notably, a fused-ring substrate afforded the desired product 3m in excellent yield, and unsubstituted pyridine underwent smooth conversion (3n). The platform was extended to quinolines bearing C2 or C6 substituents (3o, 3p), indicating its translatability across azine scaffolds. To demonstrate its utility in late-stage derivatization, derivatives of vismodegib, bisacodyl, clofibric acid, and valproic acid were functionalized in good efficiency with C4 selectivity throughout (3q–3t).

**Table 2:** Scope of pyridyl rings^a^

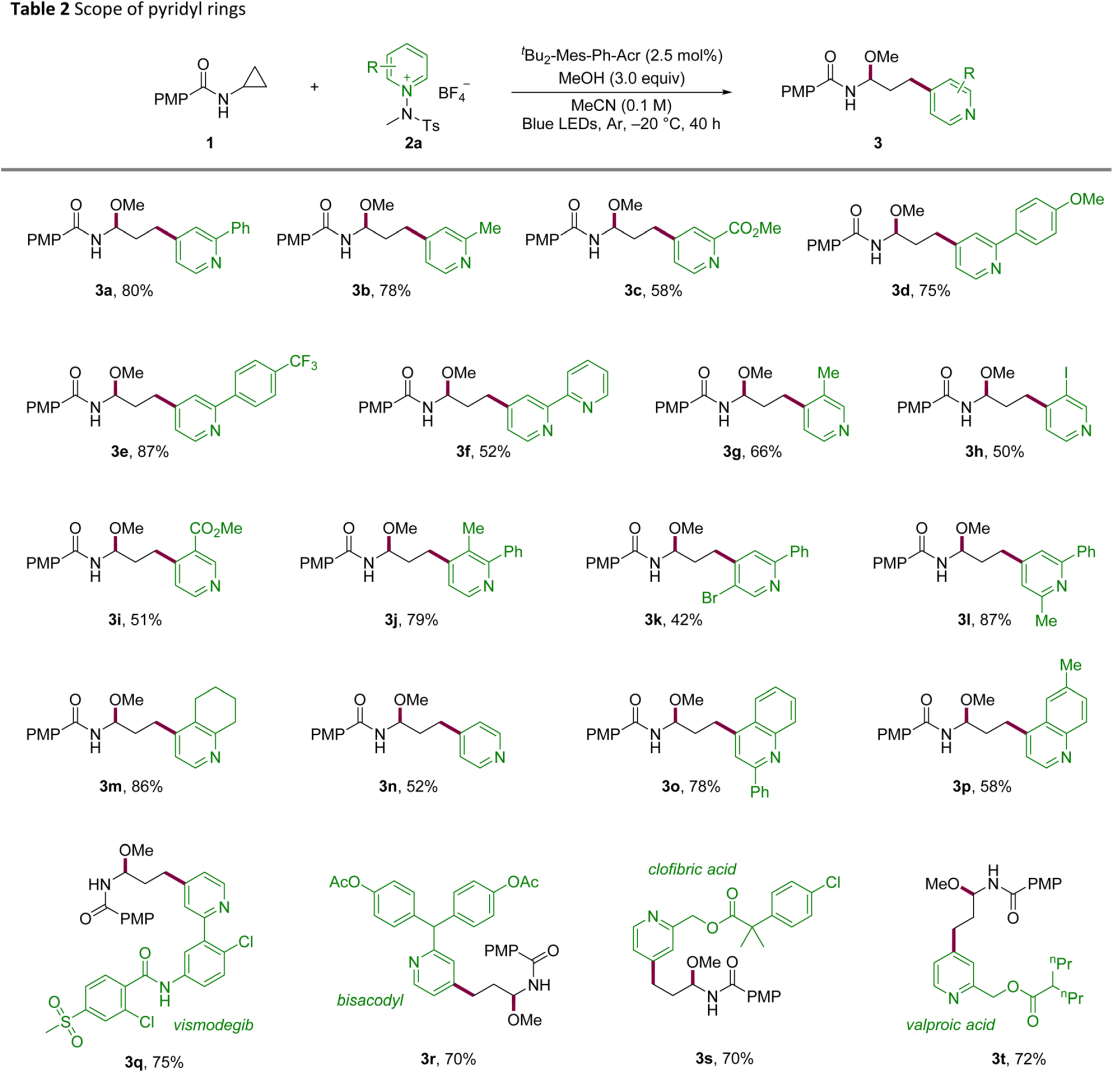

a Reaction conditions: 1a (0.1 mmol), 2 (0.15 mmol), ^*t*^Bu_2_-Mes-Ph-Acr (2.5 mol%), MeOH (0.3 mmol) in MeCN (1.0 mL) under irradiation with 440 nm LEDs (10 W) at −20 °C for 40 h under argon.

**Table 3:** Scope of aminocyclopropanes and extension to azide nucleophiles^a^

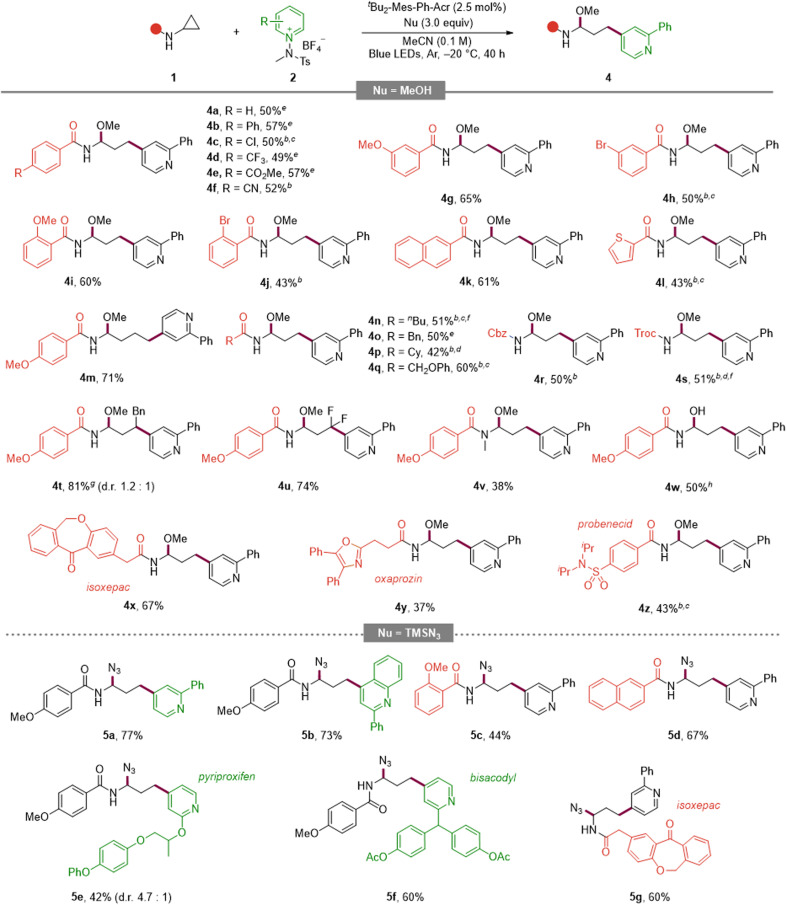

a Reaction conditions: 1 (0.1 mmol), 2a (0.15 mmol), ^*t*^Bu_2_-Mes-Ph-Acr (2.5 mol%), MeOH (0.3 mmol) in MeCN (1.0 mL) under irradiation with 440 nm LEDs (10 W) at −20 °C for 40 h under an argon atmosphere. Azidation: TMSN_3_ (0.3 mmol) was used instead of MeOH.

b Reaction at room temperature.

c 24 h reaction time.

d 72 h reaction time.

e Reaction at room temperature, 24 h reaction time, 5 mol% of PC used.

f 5 equiv. of MeOH and 2b used.

g Yield of inseparable diastereomer mixtures.

h H_2_O instead of MeOH. Diastereomeric ratios determined by ^1^H NMR analysis of the crude mixtures.

Turning to the aminocyclopropane partner (Table 3, top), an aryl-substituted cyclopropane delivered product 4a. Para-substituted arenes bearing electron-donating or electron-withdrawing groups were compatible (4b, 4c–4f), and *meta*/*ortho* substitution patterns were likewise tolerated with comparable efficiencies (4g–4j). Extended aromatics such as naphthalene and benzothiophene performed well (4k, 4l). Increasing the ring-size from cyclopropyl to cyclobutyl was viable (4m). Aliphatic and benzylic variants were accommodated (4n–4p), as were ether-containing substrates (4q), and common N-protecting groups (Cbz and Troc) were retained (4r, 4s).

To expand the versatility of the reaction, we tried changing the substituents. The reaction was also successfully carried out in the case of 1,2-disubstituted and difluorine-substituted aminocyclopropane (4t, 4u). Notably, the desired products were secured even from the tertiary amide substrate (4v), which was previously reported to be unreactive or susceptible to decomposition owing to product instability.^10*b*–*d*,10*f*^ Also, substituting water for methanol as the nucleophile furnished the corresponding product under the standard conditions (4w). Finally, late-stage diversification of pharmaceutically relevant scaffolds (isoxepac, oxaprozin, probenecid) proceeded in synthetically useful yields (4x–4z). Collectively, these scope examinations highlight the broad compatibility of this reaction across electronic properties, substitution patterns, and molecular complexity, enabling late-stage diversification while preserving C4 selectivity.

To expand the nucleophile scope, we explored the use of TMSN_3_ as an azide source (Table 3, bottom). Under the optimized photoredox conditions, a variety of pyridinium salts and aminocyclopropanes were smoothly converted into the corresponding azido aminals. Within the pyridinium series, both a C2-substituted pyridine (5a) and a C2-substituted quinoline (5b) furnished the desired products in high yields. Electron-rich and extended aromatic aminocyclopropanes were also competent partners: the *o*-methoxy benzamide derivative (5c) and the naphthamide analogue (5d) delivered the products efficiently. Furthermore, late-stage azidation of drug-derived substrates such as pyriproxyfen (5e), bisacodyl (5f), and the bioactive scaffold isoxepac (5g) proceeded in synthetically useful yields.

**Fig. 2:**
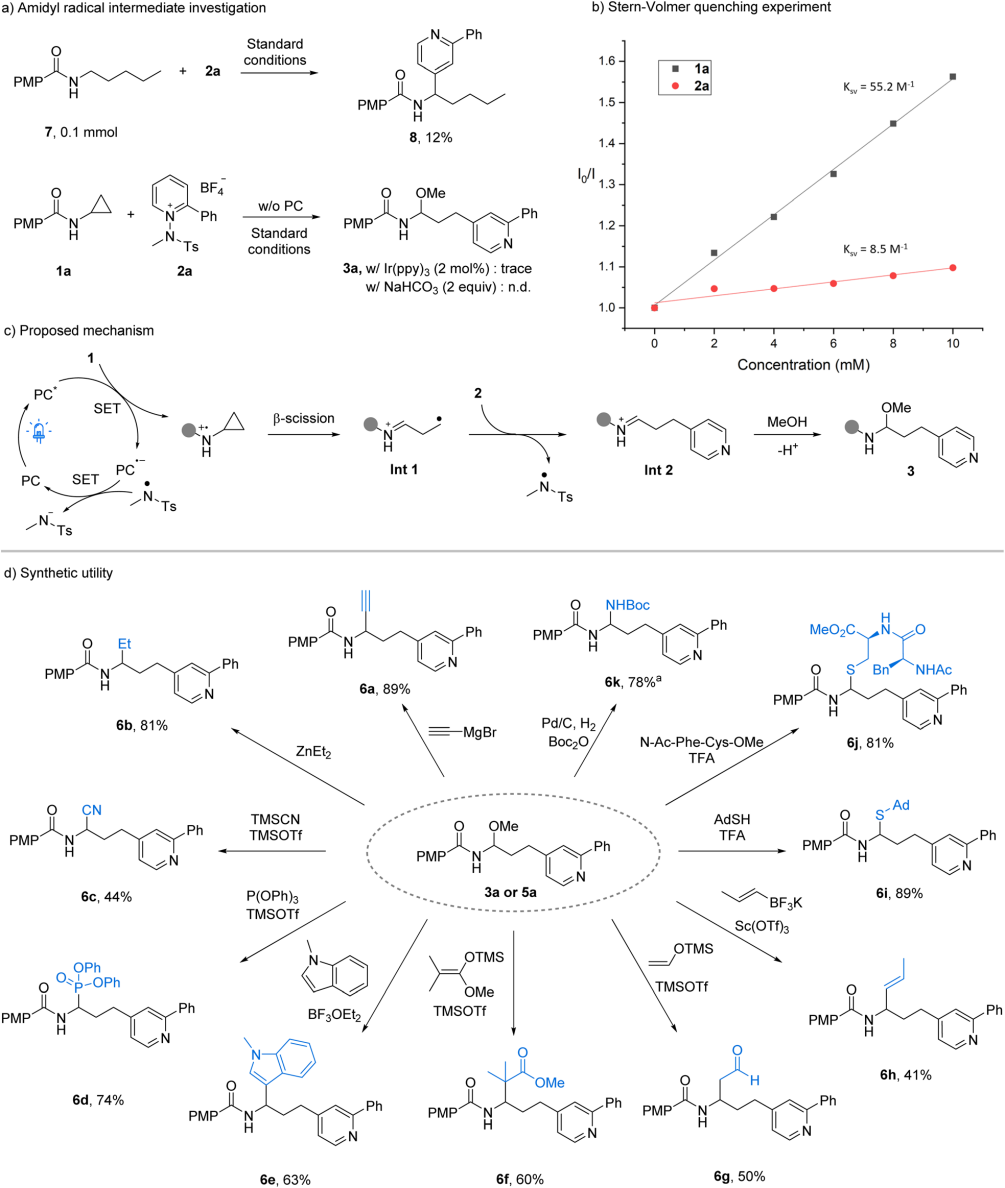
Mechanistic studies and synthetic utility. ^a^From 5a.

To elucidate the reaction pathway, we performed a series of control and trapping experiments. Excluding either the photocatalyst or light completely suppressed product formation, and the reaction proved sensitive to air (Table S6 in the SI), ruling out a two-electron mechanism. Radical inhibition by TEMPO halted the reaction, while butylated hydroxytoluene and 1,1-diphenylethylene significantly reduced conversion; the TEMPO adduct was detected by LC-MS, confirming radical intermediacy (Table S7 in the SI). Evidence for an *N*-centered radical was obtained using linear pentyl benzamide: under standard conditions, 7 underwent H-atom transfer to give 8, consistent with amidyl generation (Fig. 2a, top).^14^ The formation of product 4v from a tertiary amide lacking an N–H bond demonstrates that the reaction proceeds without N–H abstraction by the *N*-methyl-tosyl radical, thereby supporting a SET-based pathway. In addition, employing Ir(ppy)_3_, which is insufficiently oxidizing to activate the aminocyclopropane, led to only trace product formation. Likewise, the use of a base to promote an electron–donor–acceptor complex resulted in no reaction (Fig. 2a, bottom). Taken together, these data indicate that H-abstraction from the amide by the N-methyl-tosyl radical is unlikely to be a productive pathway. Finally, the low quantum yield (*Φ* = 0.145) further indicates that any chain-propagation pathway is minimal (see SI). Stern–Volmer quenching confirmed that 1a efficiently quenches the excited photocatalyst, supporting initial SET oxidation of the aminocyclopropane (Fig. 2b). On the basis of these results and precedents, we propose the mechanism shown in Fig. 2c. Photoexcited acridinium (*E*_red_ = +2.15 V *vs.* SCE)^13^ oxidizes the aminocyclopropane (*E*_p_ = +1.67 V *vs.* SCE);^10*c*^ subsequent β-scission yields a distonic iminium radical cation (Int-1). Coupling with the *N*-aminopyridinium electrophile forms the pyridylated iminium ion (Int-2) with concurrent N–N bond cleavage to release an *N*-methyl-tosyl radical (*E*_red,cal_ = +0.47 V *vs.* SCE in MeCN),^12*b*^ which closes the catalytic cycle *via* oxidation of the reduced photocatalyst (*E*_ox_ = −0.52 V *vs.* SCE).^13^ Trapping of the iminium by the nucleophile (MeOH or TMSN_3_) affords the observed *N*,*O*-acetal or azido aminal products.

Gram-scale reactions (5 mmol *N*,*O*-acetal, 3 mmol azido aminal) delivered the products in 61% and 65% yield, respectively (Table S8 in the SI), demonstrating the scalability and practicality of this approach. The resulting scaffolds underwent diverse downstream transformations (Fig. 2d): Grignard and organozinc additions furnished alkynylated and alkylated derivatives (6a, 6b); Lewis-acid activation enabled cyanation, phosphorylation, and indole C3-alkylation (6c–6e). Mukaiyama and Petasis-type reactions with silyl enol ethers or tetrafluoroborate salts provided ester, aldehyde, and alkene motifs (6f–6h). TFA-mediated thiol additions generated stable *N*,*S*-acetal (6i) and allowed peptide-based thiol to be incorporated (6j), demonstrating compatibility with biomolecular substrates. Finally, the reduction of azido aminal product 5a yielded the corresponding amine, which was readily protected as Boc derivative 6k. Overall, these mechanistic and synthetic studies underscore both the radical nature and the broad synthetic versatility of this transformation, establishing it as a robust platform for the late-stage diversification of pyridine-linked scaffolds.

## Conclusions

This work establishes a broadly applicable platform for the installation of *N*-acyl-*N*,*O*-acetal moieties onto pyridine and quinoline *via* visible-light activation of aminocyclopropanes. By converting strain release into selective C(sp^3^)–C(aryl) bond formation, this method enables the mild, oxidant-free installation of *N*-acyl-*N*,*O*-acetals linkers into pyridines, delivering excellent C4-regioselectivity and broad substrate scope. The transformation integrates radical reactivity with programmable functionalization, thus offering a general approach for late-stage diversification and modular assembly of heteroaromatic frameworks. Beyond providing a practical tool for medicinal chemistry, this strategy redefines aminocyclopropanes as versatile precursors for the installation of *N*-acyl-*N*,*O*-acetals and incorporation of diverse nucleophiles, enabling modular access to heteroaryl frameworks bearing tunable functionalities.

## Author contributions

D. K. and S. H. conceived the idea of the project. D. K., E. Y. and Y. C. performed the experiments and analyzed the data. All authors wrote the manuscript.

## Conflicts of interest

There are no conflicts to declare.

## Supplementary Material

SC-OLF-D5SC08055J-s001

## Data Availability

Detailed synthetic procedures, supporting experimental results, and complete characterization data for all new compounds can be found in the supplementary information (SI). Supplementary information: experimental procedure, characterization of new compounds (^1^H and ^13^C NMR spectra). See DOI: https://doi.org/10.1039/d5sc08055j.
