# Supplementary material for: Photocatalytic construction of N-acyl-N,O-acetal-linked pyridines via aminocyclopropane ring opening
Source: Chem Sci. 2025 Dec 9;17(7):3675–82. doi: 10.1039/d5sc08055j (PMC12757785; doi:10.1039/d5sc08055j)
Supplement: SC-017-D5SC08055J-s001 [file SC-017-D5SC08055J-s001.pdf]

Supplementary Information (SI) for Chemical Science.
This journal is © The Royal Society of Chemistry 2025

Supporting Information

Photocatalytic Installation of N-Acyl-N,O-Acetal Linkers at Pyridine through Aminocyclopropane Ring-Opening

Doyoung Kim, Eunseon Yang, Yoonhee Cho and Sungwoo Hong*

Department of Chemistry, Korea Advanced Institute of Science and Technology (KAIST), and Center for Catalytic Hydrocarbon Functionalizations, Institute for Basic Science (IBS), Daejeon 34141, Korea

Contents

I. General Methods and Materials

II. General Procedures for Substrate Preparation

III. General Procedures for Ring Opening and Further Transformation

IV. Optimization Studies

V. Mechanistic Studies

VI. Unsuccessful Result

VII. References

VIII. Compound Characterizations

IX. Spectral Copies of 1H-, 13C-, 19F-, 31P- NMR Data

I. General Methods and Materials

Unless stated otherwise, reactions were performed in oven-dried glassware. All commercially available reagents were purchased from Sigma-Aldrich, Alfa Aesar, TCI Chemicals, Acros Organics, Angene Chemicals, BLDpharm. Commercial grade reagents and solvents were used without further purification. Analytical thin layer chromatography (TLC) was performed on precoated silica gel 60 F^{254} plates and visualization on TLC was achieved by UV light (254 and 365 nm). Flash column chromatography was performed on silica gel (400-630 mesh) or a Combi*Flash*® R_f^+ system with Redi*Sep*® R_f silica columns (230-400 mesh) using a proper eluent. ^{1}H NMR was recorded on Bruker Ascend 400 MHz, Bruker Avance 500 MHz, Agilent Technologies DD2 600 MHz. Chemical shifts were quoted in parts per million (ppm) referenced to the appropriate solvent peak (e.g. 7.26 ppm for $CDCl_3$). The following abbreviations were used to describe peak splitting patterns when appropriate: br = broad, s = singlet, d = doublet, t = triplet, q = quartet, m = multiplet, dd = doublet of doublet, td = triplet of doublet, ddd = doublet of doublet of doublet. Coupling constants, *J*, were reported in the hertz unit (Hz). ^{13}C NMR Bruker Ascend 100 MHz, Bruker Avance 125 MHz and was fully decoupled by broad band proton decoupling. Chemical shifts were reported in ppm referenced to the centerline of a triplet at 77.16 ppm of $CDCl_3$. ^{19}F NMR was recorded on Bruker Ascend (301 MHz), Brucker Avance (376 MHz). High-resolution mass spectra were obtained by using ESI from Korea Basic Science Institute (Ochang).

II. General Procedures for Substrate Preparation

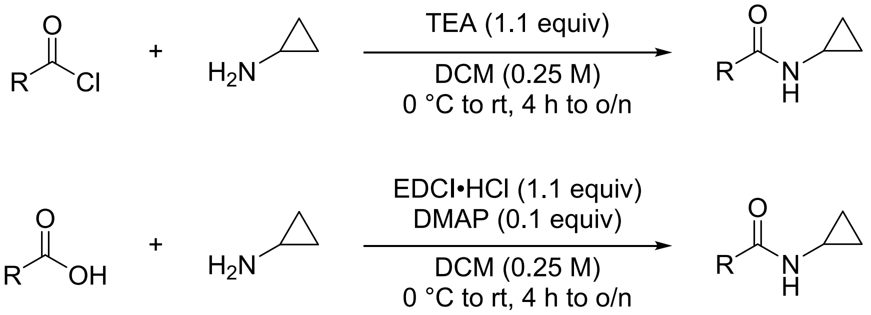

Scheme S1. Preparation of aminocyclopropane

Synthesis of aminocyclopropane from acyl chloride

To a solution of cyclopropylamine (1.1 equiv) in DCM (0.25 M) was added TEA (1.1 equiv), acyl chloride (1.0 equiv) in DCM (0.25 M) dropwise at 0 °C. Reaction mixture slowly warm up to rt and react for 4 to 16 h. After the reaction finished, quench (1N HCl) and extract with DCM for three times. The combined organic extracts were washed sequentially with 1N NaOH, brine, dried over sodium sulfate, filtered, and concentrated under reduced pressure. Purity was examined, and the compound was used without further purification when clean. Otherwise, additional purification was performed by column chromatography (EA & Hx or DCM & MeOH).

Synthesis of aminocyclopropane from carboxylic acid

To a solution of carboxylic acid (1.0 equiv) in DCM (0.25 M) was added 1-Ethyl-3-(3-dimethylaminopropyl)carbodiimide hydrochloride (EDCI·HCl, 1.1 equiv), 4-(Dimethylamino)pyridine (DMAP, 0.1 equiv) at 0 °C. After 15 min, cyclopropylamine (1.1 equiv) was added and slowly warm up to rt. After reaction finished, reaction mixture extracts with DCM for 3 times and washed with brine, dried over sodium sulfate, filtered, and concentrated under reduced pressure. The residue was purified by flash column chromatography (EA & Hx or DCM & MeOH), affording the desired aminocyclopropane.

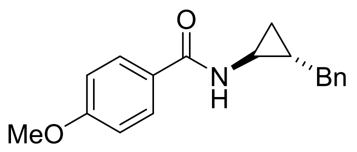

1t

N-(trans-2-benzylcyclopropyl)-4-methoxybenzamide (1t). Yield : 20% (223 mg). White solid. ^{1}H NMR (500 MHz, $CDCl_3$) δ 7.70 (d, *J* = 8.8 Hz, 2H), 7.33 – 7.24 (m, 4H), 7.25 – 7.15 (m, 1H), 6.93-6.80 (m, 2H), 6.28 (s, 1H), 3.82 (s, 3H), 2.89 (dd, *J* = 14.7, 6.3 Hz, 1H), 2.80 (dd, *J* = 7.1, 3.6 Hz, 1H), 2.51 (dd, *J* = 14.7, 7.5 Hz, 1H), 1.38 – 1.18 (m, 1H), 0.87 – 0.76 (m, 2H). ^{13}C NMR

(125 MHz, CDCl$_3$) 168.3, 162.3, 140.9, 128.8, 128.6, 128.5, 126.8, 126.2, 113.8, 55.5, 38.2, 29.7, 21.4, 14.0. HRMS (ESI, m/z) calculated for $C_{18}H_{20}NO_2^+$: 282.1494 , found : 282.1495, calculated for $C_{18}H_{19}NO_2Na^+$: 304.1313, found : 304.1314

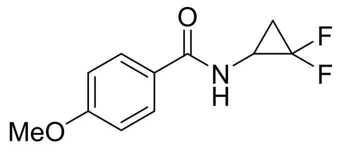

1u

N-(2,2-difluorocyclopropyl)-4-methoxybenzamide (1u). Yield : 18% (202 mg). White solid. ^{1}H NMR (600 MHz, CDCl$_3$) δ 7.73 (dd, *J* = 8.1, 1.5 Hz, 2H), 7.09 – 6.88 (m, 2H), 6.47 – 6.19 (m, 1H), 3.85 (d, *J* = 1.0 Hz, 3H), 3.52 (tdd, *J* = 9.6, 5.8, 2.9 Hz, 1H), 1.89 (dtd, *J* = 13.1, 9.3, 6.5 Hz, 1H), 1.44 (td, *J* = 9.1, 4.4 Hz, 1H). ^{13}C NMR (125 MHz, CDCl$_3$) δ 167.9, 162.6, 128.9, 125.5, 113.8, 110.3 (m), 55.4, 30.8 (dd, *J* = 15.1, 9.2 Hz), 19.1 (t, *J* = 10.0 Hz). ^{19}F NMR (376 MHz, CDCl$_3$) δ -131.18 (d, *J* = 162.4 Hz, 1F), -143.56 (d, *J* = 162.3 Hz, 1F). HRMS (ESI, m/z) calculated for $C_{11}H_{12}NO_2F_2^+$: 228.0836 , found : 228.0836, calculated for $C_{11}H_{11}NO_2F_2Na^+$: 250.0656 , found : 250.0655.

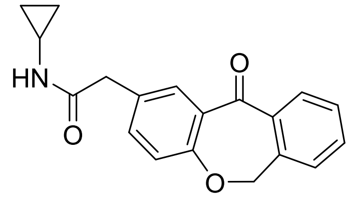

1x

N-cyclopropyl-2-(11-oxo-6,11-dihydrodibenzo[b,e]oxepin-2-yl)acetamide (1x). Yield : 48% (222.7 mg) Yellow solid. Rotamer. ^{1}H NMR (500 MHz, CDCl$_3$) δ 8.15 – 8.01 (m, 1H), 7.89 (d, *J* = 6.1 Hz, 1H), 7.57 (td, *J* = 7.5, 1.4 Hz, 1H), 7.48 (td, *J* = 7.6, 1.3 Hz, 1H), 7.42 (dd, *J* = 8.4, 2.4 Hz, 1H), 7.38 (dd, *J* = 7.5, 1.3 Hz, 1H), 7.05 (d, *J* = 8.5 Hz, 1H), 5.54 (s, 1H), 5.20 (s, 2H), 3.53 (s, 2H), 2.67 (tq, *J* = 7.1, 3.6 Hz, 1H), 0.78 – 0.71 (m, 2H), 0.47 – 0.41 (m, 2H). ^{13}C NMR (125 MHz, CDCl$_3$) δ 172.8, 162.5, 145.6, 135.0, 132.5, 129.0, 128.80, 128.77, 128.74, 128.68, 128.66, 128.3, 128.1, 128.0, 126.6, 33.1, 24.1, 23.3, 22.8, 8.5, 6.7. HRMS (ESI, m/z) calculated for $C_{19}H_{18}NO_3^+$: 308.1287, found : 308.1286, calculated for $C_{19}H_{17}NO_3Na^+$: 330.1107, found : 330.1107

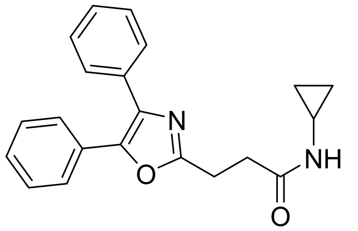

1y

N-cyclopropyl-3-(4,5-diphenyloxazol-2-yl)propanamide (1y). Yield : 32% (213.5 mg) White solid. Rotamer. ^{1}H NMR (500 MHz, CDCl$_3$) δ 7.68 – 7.55 (m, 4H), 7.43 – 7.32 (m, 6H), 6.28 (s, 1H), 3.21 (t, *J* = 7.2 Hz, 2H), 2.79 – 2.70 (m, 3H), 0.78 (td, *J* = 7.0, 5.3 Hz, 2H), 0.54 – 0.47 (m, 2H). ^{13}C NMR (125 MHz, CDCl$_3$) δ 172.8, 162.5, 145.6, 135.0, 132.5, 129.0, 128.80, 128.77, 128.74, 128.68, 128.66, 128.3, 128.1, 128.0, 126.6, 33.1, 24.1, 23.3, 22.8, 8.5, 6.7. HRMS (ESI, m/z) calculated for $C_{21}H_{21}N_2O_2^+$: 333.1603, found : 333.1604, calculated for $C_{21}H_{20}N_2O_2Na^+$: 355.1422, found : 355.1421

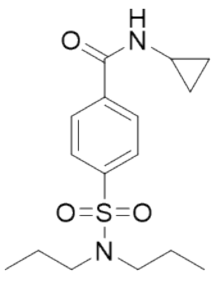

1z

N-cyclopropyl-4-(N,N-dipropylsulfamoyl)benzamide (1z). Yield : 89% (719.8 mg). White solid. ^{1}H NMR (400 MHz, $CDCl_3$) δ 7.82 (d, *J* = 0.9 Hz, 4H), 6.36 (s, 1H), 3.29 – 2.86 (m, 4H), 2.92 (dq, *J* = 7.1, 3.5 Hz, 1H), 1.58 – 1.44 (m, 4H), 0.96 – 0.78 (m, 8H), 0.72 – 0.59 (m, 2H). ^{13}C NMR (100 MHz, $CDCl_3$) δ 167.7, 143.0, 138.1, 127.7, 127.4, 50.0, 23.5, 22.0, 11.3, 6.9. HRMS (ESI, m/z) calculated for $C_{16}H_{25}N_2O_3S^+$: 325.1586, found : 325.1586, calculated for $C_{16}H_{24}N_2O_3NaS^+$: 347.1405, found : 347.1407.

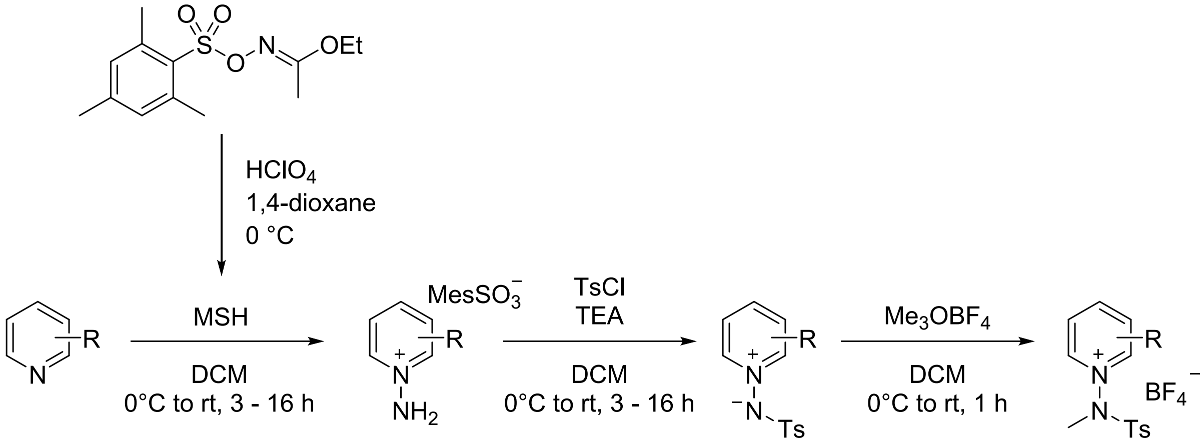

Scheme S2. Preparation of N-N pyridinium salt

Synthesis of N-N pyridinium ylides

MSH was synthesized according to a reported literature.[S1]

N-N pyridinium salts were prepared through a modified version of a reported protocol.[1] To a solution of pyridine (1.0 equiv) in dichloromethane (DCM, 0.5M) was added O-mesitylsulfonylhydroxylamine (MSH, 1.0 equiv) dropwise at 0 °C. After addition, slowly warm up to rt and react for 3-16 h. This aminopyridinium salt can be isolated by column chromatography (MC & MeOH for eluent) or directly used without purification. To a solution of 1-amidopyridinium (1.0 equiv) in DCM (0.5 M) was added triethylamine (TEA, 2.0 equiv) dropwise at 0 °C and react for 15 min. Add tosyl chloride (1.1 equiv), the mixture was slowly warm up to rt and stirred for 3-16 h. After the reaction finished, add saturated $NaHCO_3$ solution and extracted with DCM 3 times. The combined organic extracts were washed sequentially with saturated $NaHCO_3$, brine, dried over sodium sulfate, filtered, and concentrated under reduced pressure. The residue was purified by recrystallization (DCM & Hexane) or flash column chromatography (DCM & MeOH), affording the desired N-N pyridinium ylides.

Synthesis of N-aminopyridinium salts[1]

To a solution of N-N pyridinium ylides (1 equiv) in DCM (0.5~1.0 M), add trimethyloxonium tetrafluoroborate (1.1 equiv) at 0 °C. The mixture slowly warm up to rt and reaction for 1 h. The resulting mixture was concentrated under reduced pressure. The residue was purified by recrystallization (DCM & Et_2O) or flash column chromatography (DCM & MeOH), affording the desired N-N pyridinium salts.

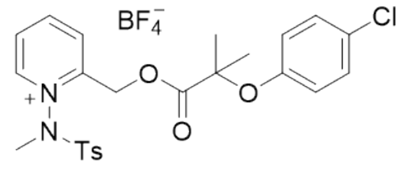

2s

2-(((2-(4-chlorophenoxy)-2-methylpropanoyl)oxy)methyl)-1-((N,4-dimethylphenyl)sulfonamido)pyridin-1-ium tetrafluoroborate (2s). Yield : 45% (187.3 mg). White solid. ^{1}H NMR (400 MHz, C_3D_6O) δ 8.95 (td, *J* = 8.0, 1.4 Hz, 1H), 8.73 (dd, *J* = 6.4, 1.5 Hz, 1H), 8.41 (dd, *J* = 8.2, 1.8 Hz, 1H), 8.34 – 8.16 (m, 1H), 7.84 (d, *J* = 8.4 Hz, 2H), 7.66 (d, *J* = 8.1 Hz, 2H), 7.37 – 7.22 (m, 2H), 7.18 – 6.90 (m, 2H), 6.28 – 5.70 (m, 2H), 3.73 (s, 3H), 2.55 (s, 3H), 1.72 (d, *J* = 4.1 Hz, 6H). ^{13}C NMR (100 MHz, C_3D_6O) δ 206.3, 173.2, 157.5, 155.0, 150.7, 149.2, 145.2, 132.1, 130.6, 130.1, 129.8, 129.2, 129.0, 128.0, 122.2, 80.5, 62.3, 40.6, 25.6, 21.8. ^{19}F NMR (376 MHz, C_3D_6O) δ -151.46. HRMS (ESI, m/z) calculated for $C_{24}H_{26}N_2O_5SCl^+$: 489.1251, found : 489.1252.

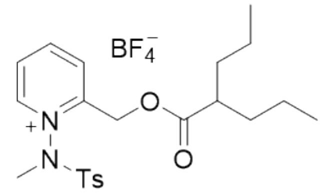

2t

1-((N,4-dimethylphenyl)sulfonamido)-2-(((2-propylpentanoyl)oxy)methyl)pyridin-1-ium tetrafluoroborate (2t). Yield : 39% (329.1 mg). White solid. ^{1}H NMR (400 MHz, C_3D_6O) δ 9.00 (t, *J* = 7.9 Hz, 1H), 8.77 (dt, *J* = 5.6, 2.8 Hz, 1H), 8.52 (d, *J* = 8.2 Hz, 1H), 8.29 (t, *J* = 7.3 Hz, 1H), 7.89 – 7.69 (m, 2H), 7.65 (d, *J* = 8.1 Hz, 2H), 5.89 (d, *J* = 16.5 Hz, 1H), 5.79 (d, *J* = 16.5 Hz, 1H), 3.75 (d, *J* = 1.6 Hz, 3H), 2.65 (tt, *J* = 8.6, 5.5 Hz, 1H), 2.54 (s, 3H), 1.78 – 1.61 (m, 2H), 1.55 (ttd, *J* = 13.0, 6.7, 3.0 Hz, 2H), 1.35 (dh, *J* = 9.8, 7.5 Hz, 4H), 0.91 (td, *J* = 7.3, 5.6 Hz, 6H).^{13}C NMR (100 MHz, C_3D_6O) δ 205.3, 174.7, 157.6, 149.9, 148.3, 144.4, 131.3, 129.7, 128.9, 128.4, 128.2, 60.4, 44.8, 39.8, 34.2, 20.9, 20.3, 20.3, 13.3. ^{19}F NMR (376 MHz, C_3D_6O) δ -151.65 (d, *J* = 10.8 Hz). HRMS (ESI, m/z) calculated for $C_{22}H_{31}N_2O_4S^+$: 419.2005 , found : 419.2005.

Synthesis of other materials

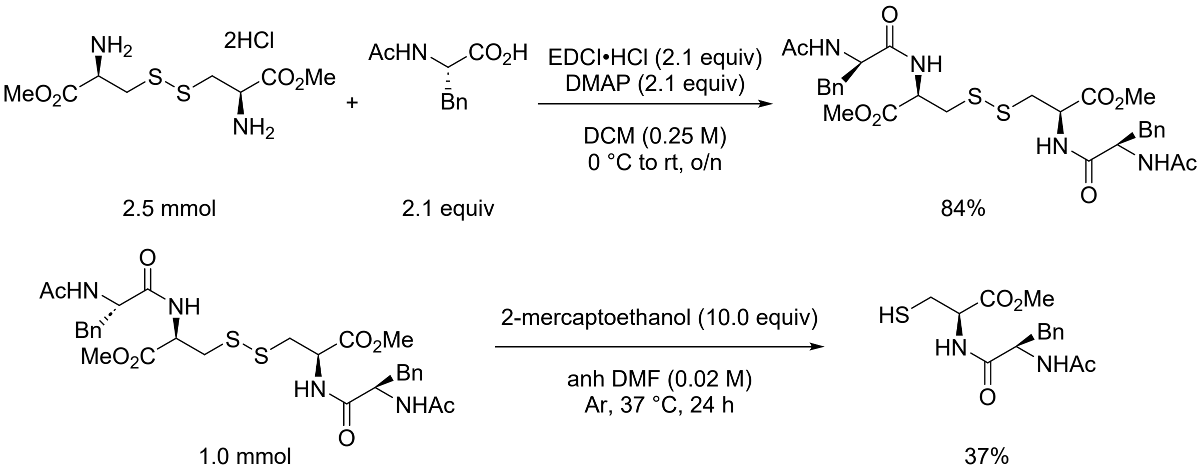

Scheme S3. Preparation of cysteine dipeptide (6ja)

To a solution of N-Acetyl-*L*-Phe (2.1 equiv) in DCM (0.25 M), EDCI·HCl (2.1 equiv) and DMAP (2.1 equiv) were added at at 0 °C. After 15 min, *L*-cystine dihydrochloride (1.0 equiv) was added and slowly warm up to rt. After reaction finished, reaction mixture extracts with DCM for 3 times and washed with brine, dried over sodium sulfate, filtered, and concentrated under reduced pressure. The residue was purified by flash column chromatography (DCM : MeOH = 3%), affording the desired *L*-phenylalanine-*L*-cysteinate.

Add anhydrous DMF (50 mL) to a *L*-phenylalanine-*L*-cysteinate under Ar atmosphere. To a solution of cysteinate, 2-

mercaptoethanol (10.0 equiv) was added and heated up to 37 °C. After reaction completed, add ice-cold water, reaction mixture extracts with EA for 3 times and washed with 5% LiCl solution, brine for each 2 times, dried over sodium sulfate, filtered, and concentrated under reduced pressure. The residue was purified by flash column chromatography (EA : Hx = 2 : 1) and reverse-phase column chromatography (MeOH : water = 1: 1), affording the desired N-acetyl-*L*-Phe-*L*-Cys-OMe (**6ja**).

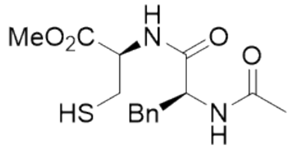

6ja

methyl acetyl-L-phenylalanyl-L-cysteinate (6ja). Yield : 37% (241.2 mg). White solid. With rotamer. ^{1}H NMR (500 MHz,C_2D_6SO) δ 8.55 (d, *J* = 8.0 Hz, 0.4H), 8.49 (d, *J* = 7.8 Hz, 0.6H), 8.15 (t, *J* = 7.8 Hz, 1H), 7.26 (p, *J* = 2.7 Hz, 4H), 7.19 (dq, *J* = 8.6, 2.9 Hz, 1H), 4.66 – 4.52 (m, 1H), 4.47 (qd, *J* = 9.0, 8.3, 6.1 Hz, 1H), 3.65 (d, *J* = 5.8 Hz, 3H), 2.98 (td, *J* = 13.5, 4.9 Hz, 1H), 2.87 (ddd, *J* = 13.7, 8.7, 5.0 Hz, 0.6H), 2.83 – 2.65 (m, 2.4H), 2.55 (t, *J* = 8.5 Hz, 0.6H), 2.34 (t, *J* = 8.6 Hz, 0.4H), 1.76 (d, *J* = 4.6 Hz, 3H). ^{13}C NMR (125 MHz, C_2D_6SO) δ 171.7, 171.5, 170.6, 170.5, 169.2, 169.1, 137.9, 137.8, 129.2, 129.1, 128.0, 126.3, 126.3, 54.5, 54.3, 53.7, 53.7, 52.1, 38.0, 37.5, 25.5, 25.2, 22.4. HRMS (ESI, m/z) calculated for $C_{15}H_{21}N_2O_4S^+$: 325.1222, found : 325.1221, calculated for $C_{15}H_{20}N_2O_4NaS^+$: 347.1041, found : 347.1040.

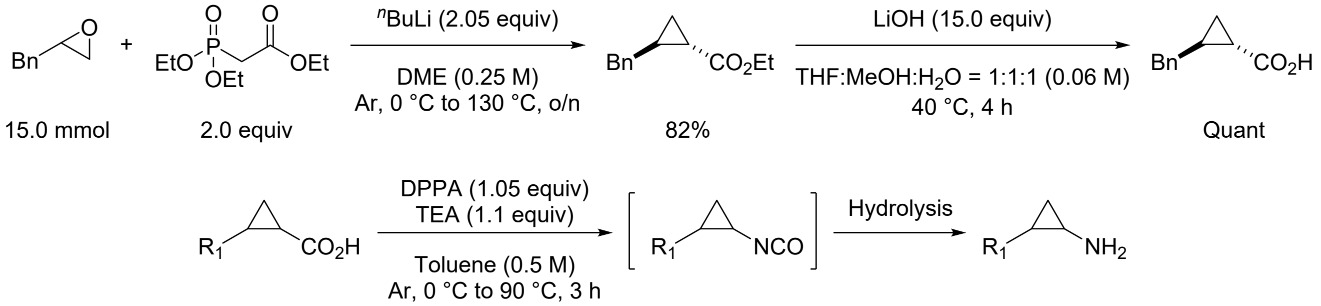

Scheme S4. Preparation of 2-substituted aminocyclopropane

To a solution of triethyl phosphonoacetate (2.0 equiv) in anhydrous DME (0.25 M), ^{n}BuLi(2.05 equiv) were added dropwise at 0 °C. After 15 min, epoxypropyl benzene (1.0 equiv) was added dropwise. The reaction mixture heated up to 130 °C for reflux, react for overnight. After reaction finished, the reaction mixture cooled to rt and quench with H_2O. Extracts with DCM for 3 times and washed with brine, dried over sodium sulfate, filtered, and concentrated under reduced pressure. The residue was purified by flash column chromatography (Hx : EA = 5%), affording the trans-ethyl 2-benzylcyclopropane-1-carboxylate as product.

To a solution of carboxylate in MeOH : THF : H_2O = 1 : 1 : 1 (0.06 M), LiOH (15 equiv) was added in a portion and heated up to 40 °C. After reaction finished, evaporate MeOH and THF, acidify the crude with 1 N HCl. Extracts with DCM for 3 times and washed with brine, dried over sodium sulfate, filtered, and concentrated under reduced pressure. Resulting trans-2-benzylcyclopropane-1-carboxylic acid was used for next step without further purification.

To a solution of cyclopropane carboxylic acid (1.0 equiv) in anhydrous toluene (0.5 M), trimethylamine (1.1 equiv) and diphenylphosphryl azide (1.05 equiv) were added dropwise at 0 °C. After 30 min, the reaction mixture heated up to 90 °C and react for 3 hours. After the reaction finished, purify resulting isocyanate with basic alumina short column and hydrolysis affording the desired cyclopropylamine after extraction.

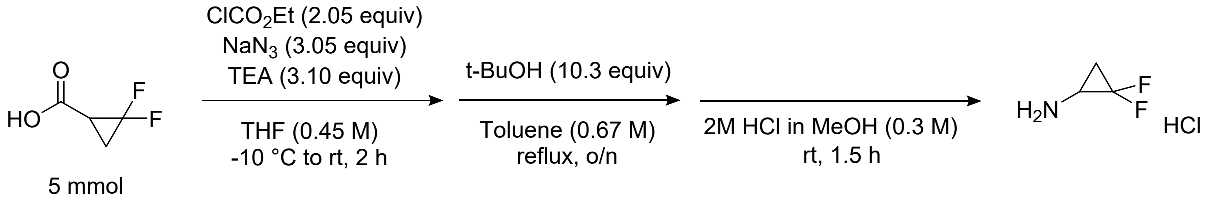

Scheme S5. Preparation of difluoro aminocyclopropane

To a solution of the corresponding acid (1.0 equiv) and Et_3N (3.10 equiv) in THF (0.45 M), ethyl chloroformate (2.05 equiv) was added dropwise at −10 °C. The reaction mixture was stirred at this temperature for 1 h, followed by the addition of NaN_3 (3.05

equiv) at −10 °C. The mixture was stirred at rt for 2 h, poured into water, and extracted with EtOAc. The combined organic layers were washed with saturated $NaHCO_3$, dried over anhydrous Na_2SO_4, filtered, and concentrated to 2 mL. The residue was diluted toluene (5.6 mL), and *t*-BuOH (10.3 equiv) was added in one portion. The mixture was refluxed overnight and concentrated under reduced pressure. The crude product was dissolved in 2M HCl in MeOH (0.3 M) and stirred at rt for 1.5 h. Concentration under vacuum followed by recrystallization from Et_2O afforded the desired amine product as a solid.

III. General Procedures for Ring Opening and Further Transformation

General Procedure for Photocatalyzed Ring Opening of Aminocyclopropane

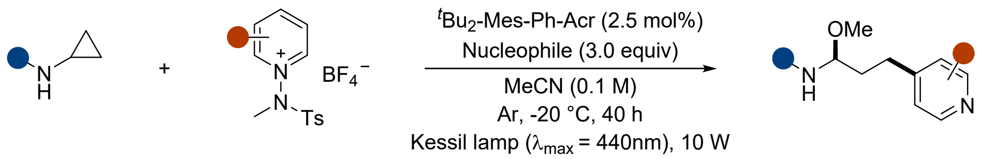

1 **2** **3**

Scheme S6. Photocatalyzed ring opening of aminocyclopropane

To an oven-dried 8 mL test tubes, **1** (0.1 mmol, 1.0 equiv), **2** (0.15 mmol, 1.5 equiv), 9-Mesityl-3,6-di-tert-butyl-10-phenylacridinium tetrafluoroborate (0.0025 mmol, 2.5 mol%) were added. The test tubes were transferred to a glove box, and nucleophile (0.3 mmol, 3.0 equiv) was added by stock solution in dry MeCN (1.0 mL, 0.1 M). After brief stirring to ensure complete dissolution of the reagents, the test tubes were removed from the glove box. The reaction mixture was stirred under irradiation with 440 nm LEDs (10 W) at –20 °C under an argon atmosphere for 40 h. Upon completion, the reaction mixture was washed with saturated sodium bicarbonate and extract with EA. The organic phase was dried over anhydrous Na_2SO_4 and concentrated under reduced pressure. Purification was carried out by flash column chromatography on silica gel or preparative thin-layer chromatography (Hx & EA or Hx & Et_2O). As wrote in main text, some scope demand longer reaction time or room temperature.

Reaction Set-up

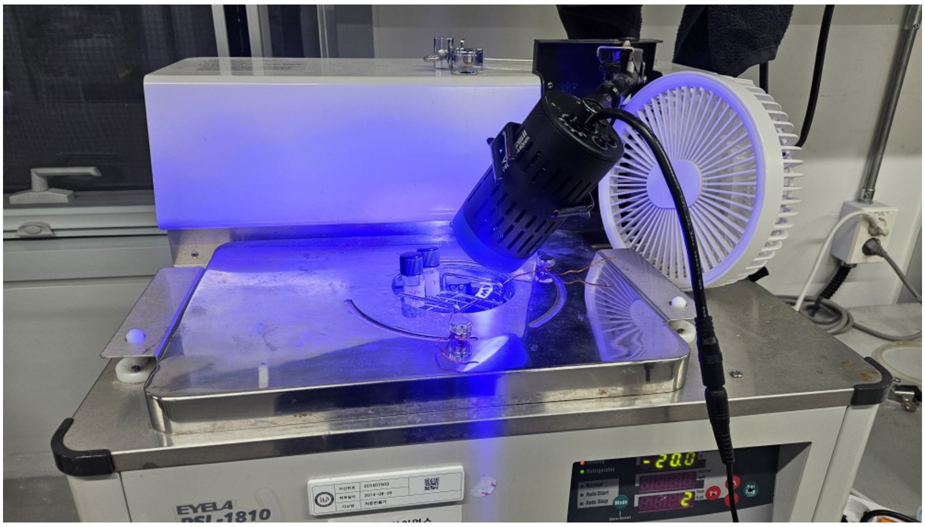

Figure S1. The reaction set up for photocatalyzed ring opening of aminocyclopropane

The Eyela PSL-1810 low-temperature reactor was used for the reaction. The reaction temperature was set to -20 °C, and the stirring rpm was set to 450 to 600 rpm. A 440 nm Kessil lamp (PR160-440 nm & PR160L-440 nm) was used at 10 W intensity, and the distance between the light source and the test tube was approximately 12 cm. The fan cooling to the kessil lamp proceeded during the reaction.

General Procedure for Further Functionalization

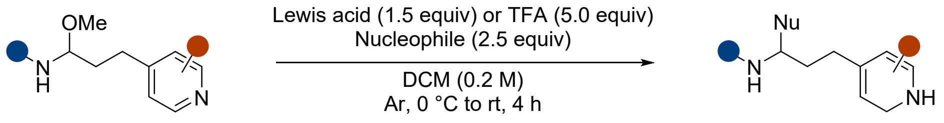

3 6

Scheme S7. Further functionalization of N-Acyl-N,O-Acetal-Linked Pyridines

To an oven-dried 8 mL test tubes, **3** (0.1 mmol, 1.0 equiv) were added in the glovebox. (Due to the hygroscopic property of **3**. If you add it in as stock, argon purging using Schlenk technic is also fine.) DCM (0.5 mL) and nucleophile (0.25 mmol, 2.5 equiv) were added at 0 °C. After brief stirring to ensure complete dissolution of the reagents, add the lewis acid (if necessary, 0.15 mmol, 1.5 equiv) or TFA (if necessary, 0.5 mmol, 5.0 equiv) to the reaction mixture and the slowly warm up to rt for 4 h. Upon completion, the reaction mixture was washed with saturated sodium bicarbonate and extract with EA. The organic phase was dried over anhydrous Na_2SO_4 and concentrated under reduced pressure. Purification was carried out by flash column chromatography on silica gel or preparative thin-layer chromatography (Hx & EA or DCM & MeOH).

IV. Optimization Studies

Table S1. Effect of solvent

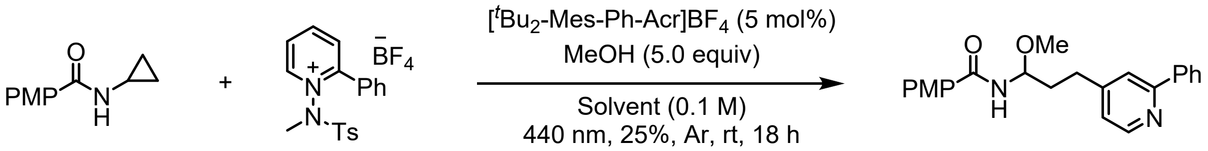

1a 0.05 mmol | **2a** 1.5 equiv | **3a**

Entry	Solvent	Yield
1	**MeCN**	50%
2	DCE	Trace
3	THF	trace
4	EA	9%
5	DMSO	8%
6	Acetone	17%
7	MeOH	11%
8	Toluene	11%

Table S2. Effect of reaction time & temperature

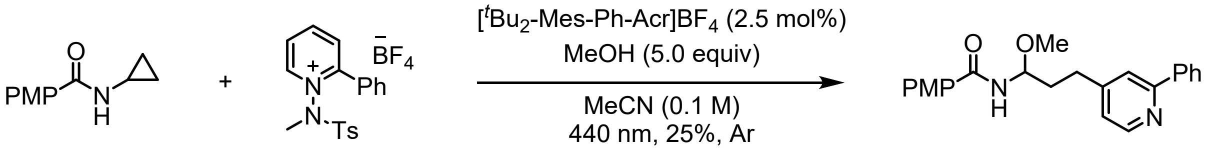

1a 0.05 mmol | **2a** 1.5 equiv | **3a**

Entry	Temperature	Rxn Time	Yield
1	- 30 °C	18 h	12%
2	- 20 °C	18 h	34%

3	0 °C	18 h	57%
4	10 °C	18 h	68%
5	25 °C	18 h	65%
6	40 °C	18 h	58%
7	- 30 °C	43 h	85%
**8**	**- 20 °C**	**48 h**	**83%**
9	40 °C	40 h	53%

Table S3. Effect of protecting group of **2**

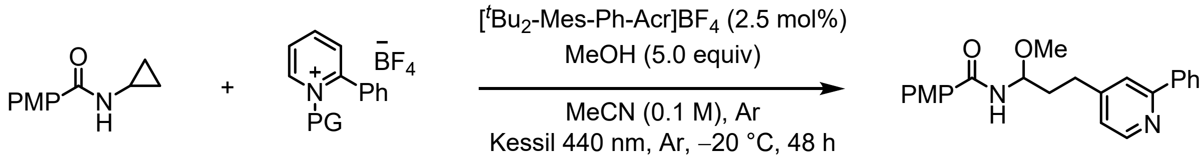

1a 0.05 mmol; **2** 1.5 equiv; **3a**

Entry	Protecting group	Yield
1	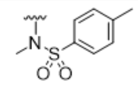	75%
2	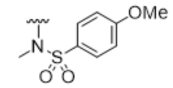	75%
3	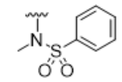	78%
4	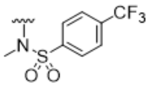	68%
5	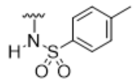	41%
6	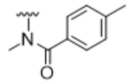	40%
7	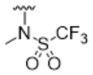	12%
8	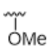	31%

Table S4. Effect of equivalent and concentration

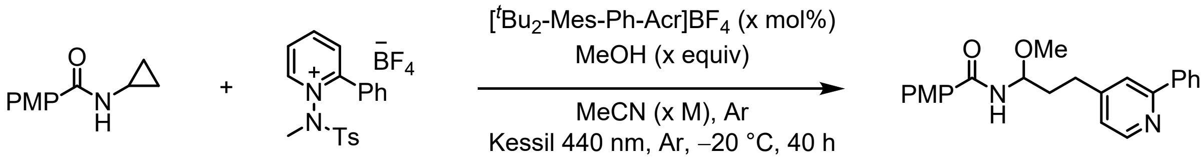

1a **2a** **3a**

0.05 mmol scale

**Entry**	CP equiv	Salt equiv	MeOH equiv	PC mol%	Concentration	Yield
1	1.0	1.0	3.0	2.5	0.1	60%

2	1.0	1.2	3.0	2.5	0.1	69%
**3**	**1.0**	**1.5**	**3.0**	**2.5**	**0.1**	**80%**
4	1.5	1.0	3.0	2.5	0.1	61%
5	1.0	1.5	3.0	1.5	0.1	66%
6	1.0	1.5	3.0	4.0	0.1	75%
7	1.0	1.5	3.0	2.5	0.05	24%
8	1.0	1.5	3.0	2.5	0.2	73%
9[a]	1.0	1.5	1.0	2.5	0.1	64%
10[a]	1.0	1.5	2.0	2.5	0.1	68%
11[a]	1.0	1.5	3.0	2.5	0.1	78%
12[a]	1.0	1.5	5.0	2.5	0.1	78%

[a]SO_2Ph Protecting group (**2b** in main text) used

Table S5. Effect of photocatalyst

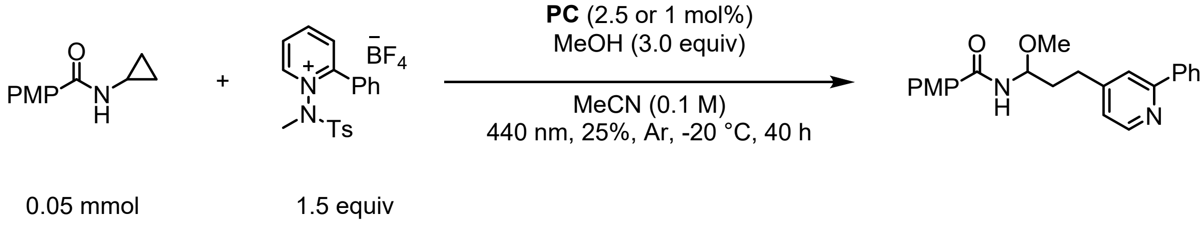

Entry	Photocatalyst	Yield
1	**[3,6[^{t}Bu$_2$-Mes-10-Ph-Acr]BF_4**	81%
2	[Mes-10-Me-Acr]BF_4	69%
3	[2,7-Me$_2$-Mes-10-Ph-Acr]BF_4	52%
4	[1,3,6,8-OMe$_4$-Mes-10-Ph-Acr]BF_4	14%
5	[Mes-10-Ph-Acr]BF_4	65%
6	[Ir{dF(CF_3)ppy$_2$}dtbbpy]PF_6	48%
7	[Ir{dF(CF_3)ppy$_2$}bpy]PF_6	49%
8	[Ir(ppy)$_2$dtbbpy]PF_6	11%
9	Ir(ppy)$_3$	Trace
10	Eosin Y	n.d.
11	4CzIPN	30%
12	TPPBF$_4$	9%

2.5 mol% for organic photocatalyst and 1 mol% for metal photocatalyst.

V. Mechanistic Study

Control experiment (Light, Air, H_2O)

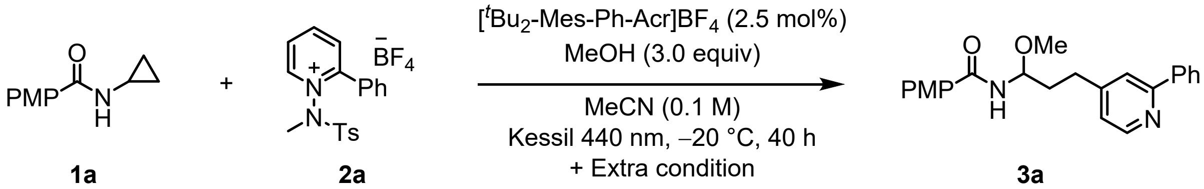

Scheme S8. Control experiments

To an oven-dried 8 mL test tubes, **1a** (0.1 mmol, 1.0 equiv), **2a** (0.15 mmol, 1.5 equiv), 9-Mesityl-3,6-di-tert-butyl-10-phenylacridinium tetrafluoroborate (0.0025 mmol, 2.5 mol%) were added (For entry 1, PC was not added). The test tubes were transferred to a glove box, and MeOH (0.3 mmol, 3.0 equiv) was added by stock solution in dry MeCN (1.0 mL, 0.1 M). After brief stirring to ensure complete dissolution of the reagents, the test tubes were removed from the glove box. For entry 3, the screw cap oppend and for entry 4, DW(1.0 mmol, 10 equiv) was added. The reaction mixture was stirred under irradiation with 440 nm LEDs (10 W) at –20 °C under an argon atmosphere for 40 h (Aluminum foil was used for entry 2). Upon completion, the reaction mixture was washed with saturated sodium bicarbonate and extract with EA. The organic phase was dried over anhydrous Na_2SO_4 and concentrated under reduced pressure. The crude was analysis by ^{1}H-NMR with the 1,3,5-trimethoxybenzene as an internal standard.

Table S6. Result of Control experiment

Entry	Condition	Yield (%)
1	No PC	n.d.
2	No Light	n.d.
3	Air	11
4	10 equiv of H_2O	55

Radical trapping experiment (TEMPO, BHT, 1,1-diphenylethylene)

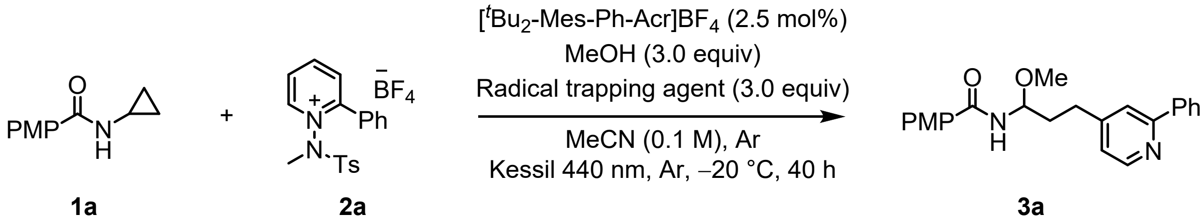

Scheme S9. Radical trapping experiments

To an oven-dried 8 mL test tubes, **1a** (0.1 mmol, 1.0 equiv), **2a** (0.15 mmol, 1.5 equiv), 9-Mesityl-3,6-di-tert-butyl-10-phenylacridinium tetrafluoroborate (0.0025 mmol, 2.5 mol%), radical trapping agent (3.0 equiv) were added. The test tubes were transferred to a glove box, and MeOH (0.3 mmol, 3.0 equiv) was added by stock solution in dry MeCN (1.0 mL, 0.1 M). After brief stirring to ensure complete dissolution of the reagents, the test tubes were removed from the glove box. The reaction mixture was stirred under irradiation with 440 nm LEDs (10 W) at –20 °C under an argon atmosphere for 40 h. Upon completion, the reaction mixture was washed with saturated sodium bicarbonate and extract with EA. The organic phase was dried over anhydrous Na_2SO_4 and concentrated under reduced pressure. The crude was analysis by ^{1}H-NMR with the 1,3,5-trimethoxybenzene as an internal standard. The TEMPO adduct was detected by LC-MS.

Table S7. Result of radical trapping experiment

Entry	Radical trapping agent	Yield (%)
1	TEMPO	n.d.
2	Butylated hydroxytoluene	13
3	1,1-diphenylethylene	18

Amidyl radical intermediate investigation

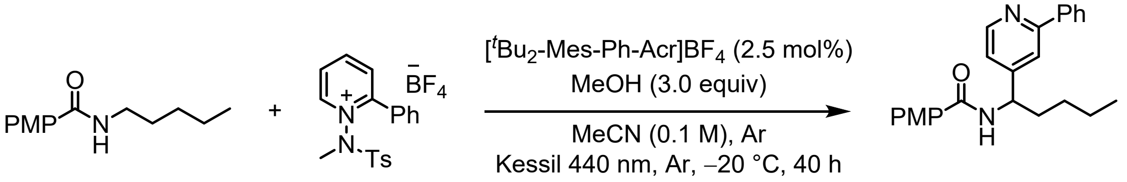

Scheme S10. Linear-alkyl substituted amide on standard reaction condition

To an oven-dried 8 mL test tubes, **7** (0.1 mmol, 1.0 equiv), **2a** (0.15 mmol, 1.5 equiv), 9-Mesityl-3,6-di-tert-butyl-10-phenylacridinium tetrafluoroborate (0.0025 mmol, 2.5 mol%) were added. The test tubes were transferred to a glove box, and MeOH (0.3 mmol, 3.0 equiv) was added by stock solution in dry MeCN (1.0 mL, 0.1 M). After brief stirring to ensure complete dissolution of the reagents, the test tubes were removed from the glove box. The reaction mixture was stirred under irradiation with 440 nm LEDs (10 W) at –20 °C under an argon atmosphere for 40 h. Upon completion, the reaction mixture was washed with saturated sodium bicarbonate and extract with EA. The organic phase was dried over anhydrous Na_2SO_4 and concentrated under reduced pressure. The yield was measured by 1H NMR with 1,3,5-trimethoxybenzene as internal standard. Purification was carried out by flash column chromatography on silica (Hx & EA). The yield measured by the 4 series of experiments.

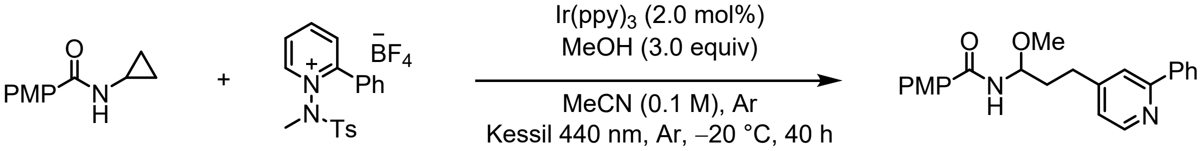

Scheme S11. Photocatalyzed ring opening reaction with Ir(ppy)$_3$

To an oven-dried 8 mL test tubes, **1a** (0.1 mmol, 1.0 equiv), **2a** (0.15 mmol, 1.5 equiv), Ir(ppy)$_3$ (0.002 mmol, 2.0 mol%) were added. The test tubes were transferred to a glove box, and MeOH (0.3 mmol, 3.0 equiv) was added by stock solution in dry MeCN (1.0 mL, 0.1M). After brief stirring to ensure complete dissolution of the reagents, the test tubes were removed from the glove box. The reaction mixture was stirred under irradiation with 440 nm LEDs (10 W) at –20 °C under an argon atmosphere for 40 h. Upon completion, the reaction mixture was washed with saturated sodium bicarbonate and extract with EA. The organic phase was dried over anhydrous Na_2SO_4 and concentrated under reduced pressure. The yield was measured by 1H NMR with 1,3,5-trimethoxybenzene as internal standard.

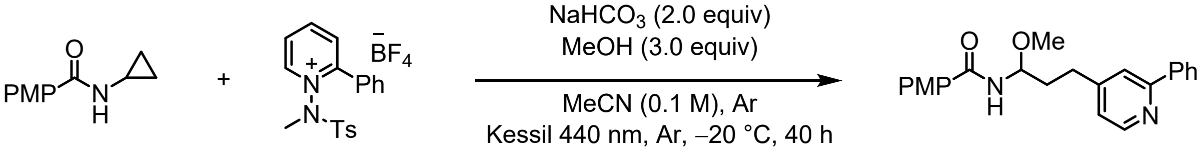

Scheme S12. Photocatalyzed ring opening reaction using EDA complexation

To an oven-dried 8 mL test tubes, **1a** (0.1 mmol, 1.0 equiv), **2a** (0.15 mmol, 1.5 equiv), $NaHCO_3$ (0.2 mmol, 2.0 equiv) were added. The test tubes were transferred to a glove box, and MeOH (0.3 mmol, 3.0 equiv) was added by stock solution in dry MeCN (1.0 mL, 0.1M). After brief stirring to ensure complete dissolution of the reagents, the test tubes were removed from the glove box. The reaction mixture was stirred under irradiation with 440 nm LEDs (10 W) at –20 °C under an argon atmosphere for 40 h. Upon completion, the reaction mixture was washed with saturated sodium bicarbonate and extract with EA. The organic phase was dried over anhydrous Na_2SO_4 and concentrated under reduced pressure. The yield was measured by 1H NMR with 1,3,5-trimethoxybenzene as internal standard.

Stern-Volmer quenching experiment

Photocatalyst (4 mM): 9-Mesityl-3,6-di-tert-butyl-10-phenylacridinium tetrafluoroborate (45.88 mg, 0.08 mmol) in 20.0 mL MeCN.
1a (80 mM): **1a** (91.79 mg, 0.48 mmol) in 6.0 mL MeCN
2a (80 mM): **2a** (102.30 mg, 0.24 mmol) in 3.0 mL MeCN

With the above solutions, the samples for the analysis were prepared as following recipe:
MeCN (3.5-x mL) + PC stock (0.5 mL) + substrate stock (x mL) = 4.0 mL

All solutions were excited at 417 nm and the fluorescence spectra was measured over the range of 400 – 700 nm, 600 nm/min among which the emission intensity was recorded at 512 nm.

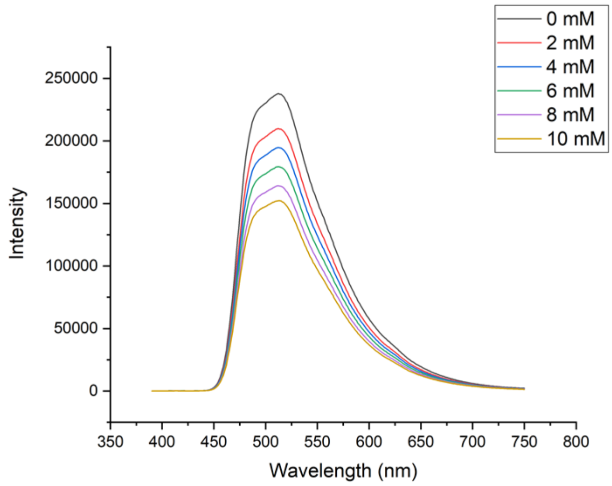

Figure S2. Quenching of the **Photocatalyst** emission in the presence of increasing amount of **1a**

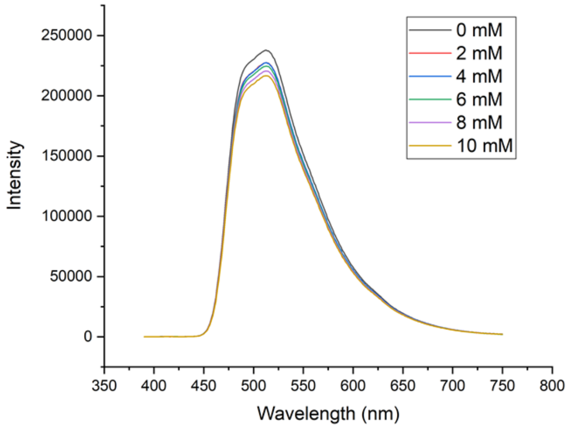

Figure S3. Quenching of the **Photocatalyst** emission in the presence of increasing amount of **2a**

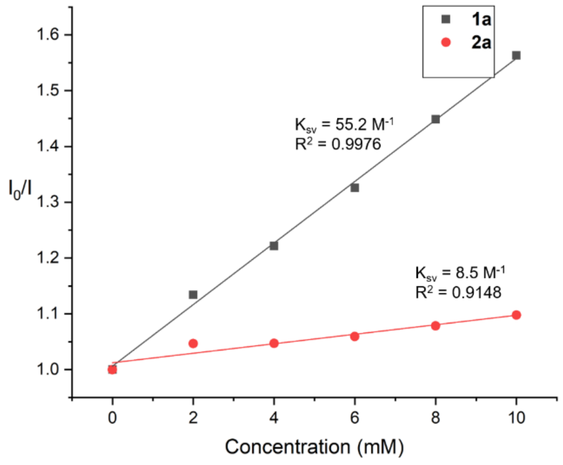

Figure S4. Stern-Volmer quenching plot with **1a, 2a**

Large-scale reaction

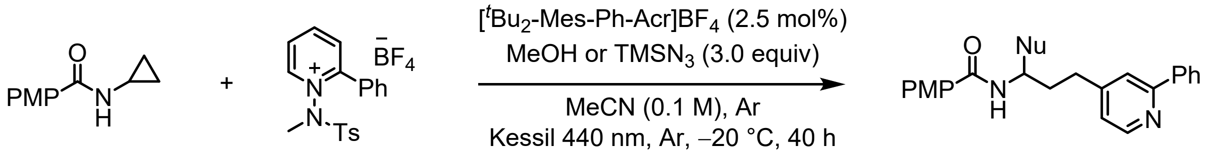

1a **2a** **3a / 5a**

Scheme S13. Large-scale reaction

To an oven-dried 250 mL rbf, **1a** (5.0 mmol or 3.0 mmol, 1.0 equiv), **2a** (1.5 equiv), 9-Mesityl-3,6-di-tert-butyl-10-phenylacridinium tetrafluoroborate (2.5 mol%) were added. The rbf were transferred to a glove box, and dry MeCN (1.0 mL, 0.1 M), MeOH or $TMSN_3$ (3.0 equiv) were added. After brief stirring to ensure complete dissolution of the reagents, the rbf were removed from the glove box. The reaction mixture was stirred under irradiation with 440 nm LEDs (10 W) at –20 °C under an argon atmosphere for 40 h. Upon completion, the reaction mixture was washed with saturated sodium bicarbonate and extract with DCM. The organic phase was dried over anhydrous Na_2SO_4 and concentrated under reduced pressure. Purification was carried out by flash column chromatography on silica gel (Hx & Et_2O).

Table S8. Result of large-scale reaction

Entry	Condition	Yield
1	MeOH, 5.0 mmol	61%
2	$TMSN_3$, 3.0 mmol	65%

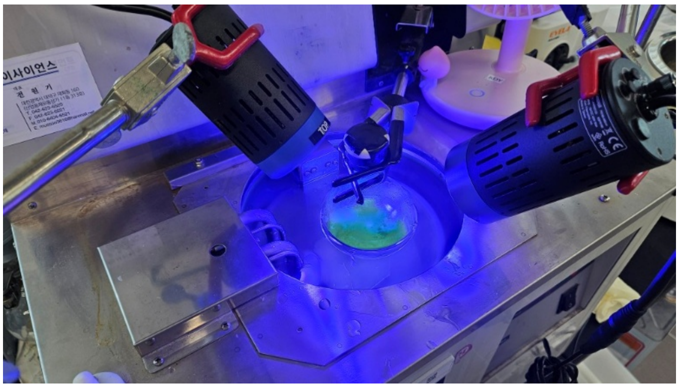

Figure S5. Reaction set-up of large-scale reaction

Quantum yield measurement

Determination of the photon flux of Kessil PR160-440 nm

The measurement was proceeded by using ferrioxalate actinometry following the literature procedures.[S2, S3]

Solution I : 89 mg of potassium ferrioxalate trihydrate, 42 μL of H_2SO_4 dissolved in 10 mL of D.W in amber vial.

Solution II : 2.5 of sodium acetate, 0.5 mL of H_2SO_4 dissolved in 50 mL of D.W.

Solution III : 15 mg of 1,10-phenanthroline dissolved in 3 mL of **Solution II** in amber vial**.** (Prepare 4 **Solution III**)

To a 8 mL test tube equipped with a magnetic bar was added 1 mL of **Solution I**. The test tube was placed 5 cm from the light source and irradiated for 15, 30, and 60 s. Immediately, 100 μL of the solution was transferred to **Solution III** and DW was added to make 10 mL solution. Shake the vial enough to mix and keep in dark for 20 min. The solution was transferred to a quartz cuvette (1.0 cm path length) to measure the absorbance at 510 nm by using UV/Vis spectroscopy.

The number of moles of Fe^{2+} formed was calculated by using Beer's law:

$$mol\ of\ Fe^{2+} = \frac{V_1V_3\Delta Abs(510\ nm)}{V_2 l\varepsilon(510\ nm)} \quad \text{(equation S1)}$$

Where,

V_1 = The volume of irradiated ferrioxalate solution (1.0×10^{-3} L)

V_2 = The volume of transferred ferrioxalate solution to measure number of moles of Fe^{2+} (1.0×10^{-4} L)

V_3 = The volume final volume of the solution after complexation with 1,10-phenanthroline (1.0×10^{-2} L)

ΔAbs(510 nm) = Difference of absorbance at 510 nm between the irradiated and non-irradiated solution.

l = Optical path-length of the cuvette (1 cm)

ε(510 nm) = Molar absorptivity of the $Fe(phen)_3^{2+}$ complex at 510 nm (11,110 L mol^{-1} cm^{-1})

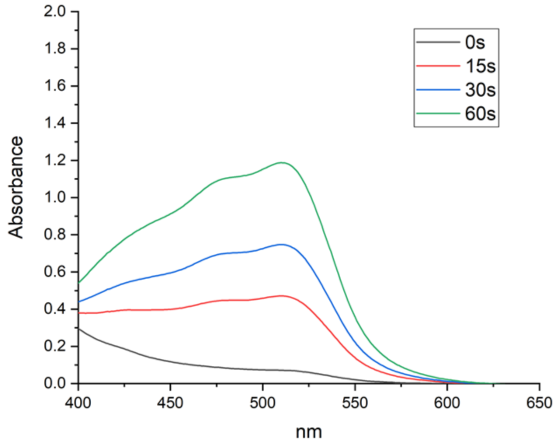

Figure S6. Absorption spectra of ferrioxalate and 1,10-phenanthroline solutions.

The photon flux was then calculated by using following equation

$$photon\ flux = \frac{mol\ Fe^{2+}}{\Phi t(1 - 10^{-Abs(\lambda)})} \quad \text{(equation S2)}$$

Φ = Quantum yield of formation Fe^{2+} at 440 nm (1.11)[S4]

t = Irradiation time

Abs(λ) = The absorbance of the 0.018 M ferrioxalate solution at 440 nm in a path length 1 cm quartz cuvette by UV/Vis spectroscopy (0.648)

Overall, the calculated photon flux is 2.22×10^{-7} einstein/s

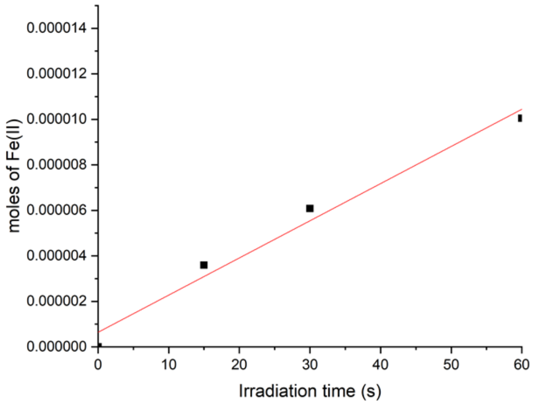

Figure S7. Moles of Fe(II) formed with different irradiation times.

Determination of the reaction quantum yield

To determine the reaction quantum yield, the cyclopropane ring opening reaction was conducted under the standard reaction setup for 15 min. The number of moles of the product was determined by ^{1}H NMR by using 1,3,5-trimethoxybenzene as an internal standard: 2.88×10^{-5} mol of **3a**. The reaction quantum yield was calculated by using following equation

$$\Phi = \frac{moles\ of\ product}{photon\ flux\ \times t(1 - 10^{-Abs(\lambda)})} \quad \text{(equation S3)}$$

Where,

Photon flux = 2.22×10^{-7} einstein/s

t = Reaction time (900 s)

Abs(λ) = The absorbance of the reaction solution at 440 nm in a path length 1 cm quartz cuvette by UV/Vis spectroscopy (2.233)

$$\Phi = \frac{2.88 \times 10^{-5}\ mol}{2.22 \times 10^{-7} s^{-1}\ \times\ 900\ s\ \times\ (1 - 10^{-2.233})} = 0.145 \quad \text{(equation S4)}$$

VI. Unsuccessful Result

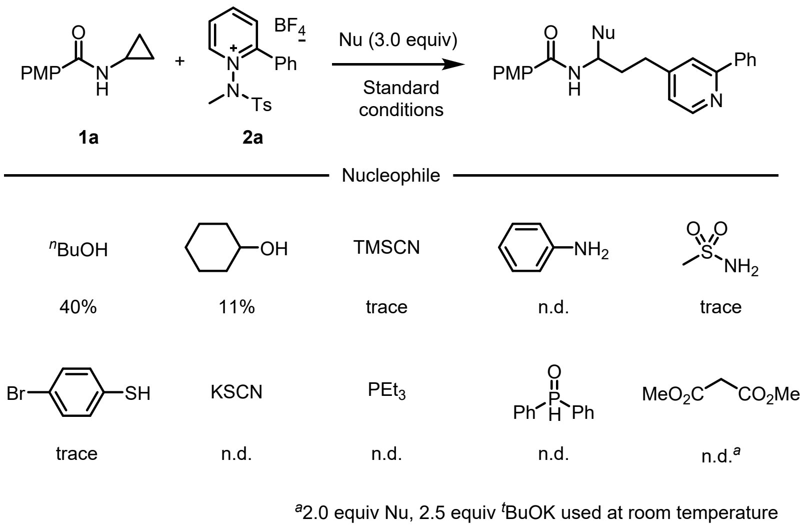

Figure S8. Other tried nucleophile

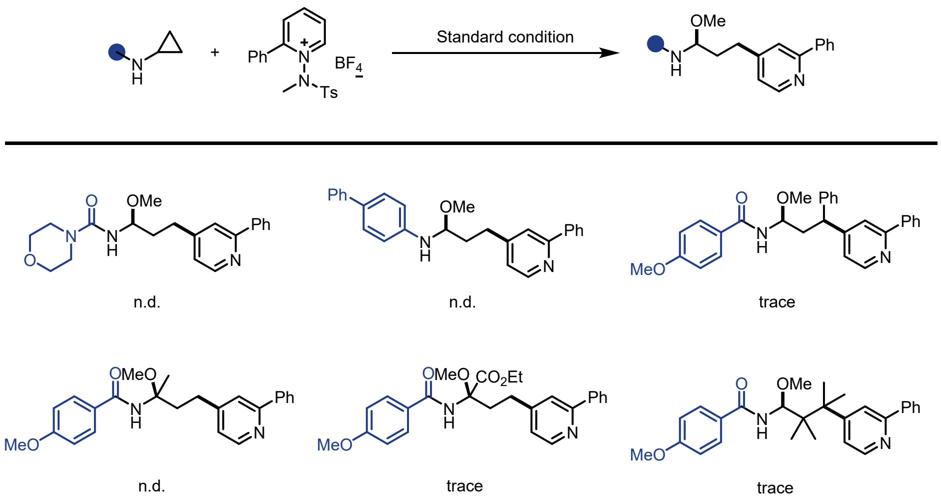

Figure S9. Failed type of cyclopropanes

VII. References

[1] Y. Moon, B. Park, I. Kim, G. Kang, S. Shin, D. Kang, M.-H. Baik, S. Hong, *Nat. Commun.,* 2019, **10**, 4117.

[2] A. Tlahuext-Aca, R. A. Garza-Sanchez, M. Schäfer, F. Glorius, *Org. Lett.,* 2018, **20**, 1546-1549.

[3] C. Kim, J. Jeong, M. Vellakkaran and S. Hong, ACS Catal., 2022, 12, 13225–13233.

[4] M. Montalti, A. Credi, L. Prodi, M. T. Gandolfi, *Handbook of Photochemistry*; CRC press, Boca Raton, 2006.

VIII. Compound Characterizations

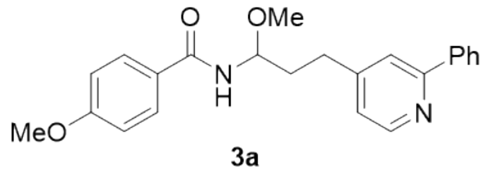

3a

4-methoxy-N-(1-methoxy-3-(2-phenylpyridin-4-yl)propyl)benzamide (3a). Yield : 80% (30.0 mg). White solid. ^{1}H NMR (400 MHz, $CDCl_3$) δ 8.57 (d, *J* = 5.0 Hz, 1H), 7.95 (dd, *J* = 8.4, 1.5z Hz, 2H), 7.70 (d, *J* = 8.8 Hz, 2H), 7.45 (dd, *J* = 8.2, 6.5 Hz, 2H), 7.41 (s, 1H), 7.08 (dd, *J* = 5.1, 1.6 Hz, 1H), 6.98 – 6.87 (m, 2H), 6.24 (d, *J* = 9.6 Hz, 1H), 5.41 (dt, *J* = 9.6, 6.2 Hz, 1H), 3.83 (d, *J* = 1.5 Hz, 3H), 3.41 (d, *J* = 1.5 Hz, 3H), 2.83 (dddd, *J* = 39.6, 14.5, 9.3, 6.4 Hz, 2H), 2.26 – 1.94 (m, 2H). ^{13}C NMR (100 MHz, $CDCl_3$) δ 167.0, 162.7, 157.8, 151.1, 149.9, 139.5, 129.0, 128.9, 128.8, 127.1, 125.9, 122.4, 120.9, 114.0, 81.3, 56.2, 55.6, 36.5, 31.0. HRMS (ESI, m/z) calculated for $C_{23}H_{25}N_2O_3^+$: 377.1865 , found : 377.1865

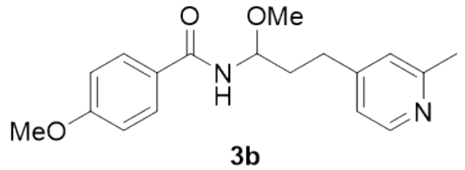

3b

4-methoxy-N-(1-methoxy-3-(2-methylpyridin-4-yl)propyl)benzamide (3b). Yield : 78% (24.4 mg). White solid. ^{1}H NMR (400 MHz, $CDCl_3$) δ 8.36 (d, *J* = 5.1 Hz, 1H), 8.01 – 7.60 (m, 2H), 6.98 (s, 1H), 6.92 (d, *J* = 9.1 Hz, 3H), 6.23 (d, *J* = 9.5 Hz, 1H), 5.35 (dt, *J* = 9.5, 6.2 Hz, 1H), 3.85 (s, 3H), 3.39 (s, 3H), 2.71 (dddd, *J* = 39.6, 14.5, 9.2, 6.3 Hz, 2H), 2.48 (s, 3H), 2.13 – 2.01 (m, 1H), 2.01 – 1.95 (m, 1H). ^{13}C NMR (100 MHz, $CDCl_3$) δ 167.0, 162.7, 158.6, 150.7, 149.3, 128.9, 126.0, 123.5, 121.0, 114.0, 81.2, 56.1, 55.6, 36.4, 30.7, 24.4. HRMS (ESI, m/z) calculated for $C_{18}H_{23}N_2O_3^+$: 315.1709 , found : 315.1710, calculated for $C_{18}H_{22}N_2O_3Na^+$: 337.1528, found : 337.1528.

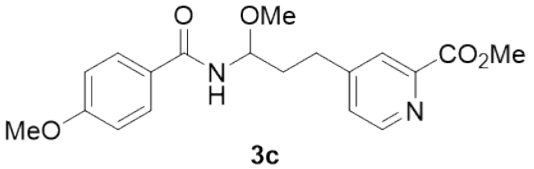

3c

methyl 4-(3-methoxy-3-(4-methoxybenzamido)propyl)picolinate (3c). Yield : 58% (20.6 mg). Yellow solid. ^{1}H NMR (400 MHz, $CDCl_3$) δ 8.61 (d, *J* = 4.9 Hz, 1H), 7.99 (d, *J* = 1.8 Hz, 1H), 7.86 – 7.69 (m, 2H), 7.32 (d, *J* = 1.9 Hz, 1H), 7.07 – 6.72 (m, 2H), 6.22 (d, *J* = 9.5 Hz, 1H), 5.63 – 5.23 (m, 1H), 3.99 (s, 3H), 3.85 (s, 3H), 3.39 (s, 3H), 3.01 – 2.68 (m, 2H), 2.36 – 1.92 (m, 2H). ^{13}C NMR (100 MHz, $CDCl_3$) δ 167.0, 166.0, 162.8, 151.9, 150.0, 148.1, 129.0, 127.2, 125.9, 125.4, 114.0, 81.1, 56.2, 55.6, 53.0, 36.3, 30.8. HRMS (ESI, m/z) calculated for $C_{19}H_{23}N_2O_5^+$: 359.1607 , found : 359.1609, calculated for $C_{19}H_{22}N_2O_5Na^+$: 381.1426, found : 381.1428.

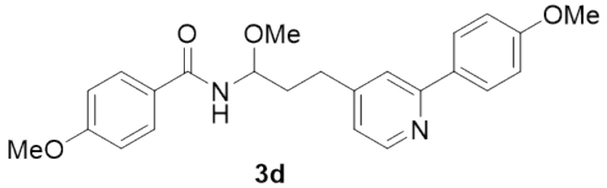

3d

4-methoxy-N-(1-methoxy-3-(2-(4-methoxyphenyl)pyridin-4-yl)propyl)benzamide (3d). Yield : 75% (30.2 mg). Yellow solid. ^{1}H NMR (500 MHz, $CDCl_3$) δ 8.53 (d, *J* = 5.0 Hz, 1H), 8.00 – 7.77 (m, 2H), 7.76 – 7.58 (m, 2H), 7.50 (d, *J* = 1.6 Hz, 1H), 7.03 (dd, *J* = 5.1, 1.6 Hz, 1H), 6.97 (d, *J* = 8.9 Hz, 2H), 6.91 (d, *J* = 8.8 Hz, 2H), 6.20 (d, *J* = 9.1 Hz, 1H), 5.41 (dt, *J* = 9.6, 6.2 Hz, 1H),

3.86 (s, 3H), 3.84 (s, 3H), 3.41 (s, 3H), 2.86 (ddd, *J* = 15.2, 9.5, 6.2 Hz, 1H), 2.76 (ddd, *J* = 14.1, 9.3, 6.4 Hz, 1H), 2.19 – 1.96 (m, 2H). ^{13}C NMR (125 MHz, $CDCl_3$) δ 167.0, 162.7, 160.5, 157.5, 151.0, 149.7, 132.1, 128.9, 128.4, 125.9, 121.8, 120.2, 114.2, 114.0, 81.3, 56.2, 55.6, 55.5, 36.5, 31.0. HRMS (ESI, m/z) calculated for $C_{24}H_{27}N_2O_4^+$: 407.1971 , found : 407.1970

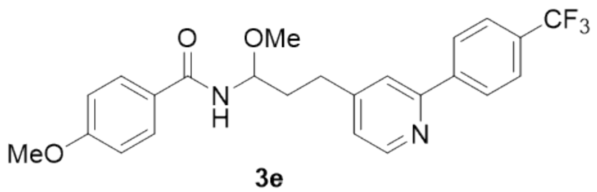

3e

4-methoxy-N-(1-methoxy-3-(2-(4-(trifluoromethyl)phenyl)pyridin-4-yl)propyl)benzamide (3e). Yield : 87% (38.6 mg). White solid. ^{1}H NMR (400 MHz, $CDCl_3$) δ 8.59 (d, *J* = 5.0 Hz, 1H), 8.06 (d, *J* = 8.1 Hz, 2H), 7.70 (t, *J* = 8.5 Hz, 4H), 7.58 (d, *J* = 1.6 Hz, 1H), 7.13 (dd, *J* = 5.0, 1.6 Hz, 1H), 6.90 (d, *J* = 8.8 Hz, 2H), 6.31 (d, *J* = 9.6 Hz, 1H), 5.40 (dt, *J* = 9.6, 6.3 Hz, 1H), 3.83 (s, 3H), 3.40 (s, 3H), 2.88 (ddd, *J* = 15.1, 9.2, 6.3 Hz, 1H), 2.79 (ddd, *J* = 14.5, 8.9, 6.6 Hz, 1H), 2.14 (ddt, *J* = 13.9, 9.7, 6.8 Hz, 1H), 2.09 – 1.96 (m, 1H). ^{13}C NMR (100 MHz, $CDCl_3$) δ 167.0, 162.8, 156.2, 151.5, 150.0, 142.8, 130.8 (q, *J* = 32.4 Hz), 129.0, 127.4, 125.8, 125.7 (q, *J* = 3.8 Hz), 123.2, 123.0, 121.3, 114.0, 81.2, 56.1, 55.5, 36.4, 31.0. ^{19}F NMR (376 MHz, $CDCl_3$) δ -62.53. HRMS (ESI, m/z) calculated for $C_{24}H_{24}N_2O_3F_3^+$: 445.1739 , found : 445.1738

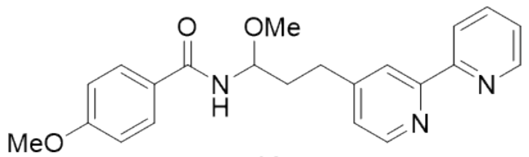

3f

N-(3-([2,2'-bipyridin]-4-yl)-1-methoxypropyl)-4-methoxybenzamide (3f). Yield : 52% (19.5 mg). Yellow solid. ^{1}H NMR (400 MHz, $CDCl_3$) δ 8.75 – 8.59 (m, 1H), 8.55 (d, *J* = 5.0 Hz, 1H), 8.37 (d, *J* = 8.0 Hz, 1H), 8.26 (d, *J* = 1.8 Hz, 1H), 7.81 (td, *J* = 7.7, 1.8 Hz, 1H), 7.70 (d, *J* = 8.8 Hz, 2H), 7.30 (ddd, *J* = 7.5, 4.8, 1.2 Hz, 1H), 7.16 (dd, *J* = 5.1, 1.8 Hz, 1H), 6.90 (d, *J* = 8.8 Hz, 2H), 6.23 (d, *J* = 9.6 Hz, 1H), 5.39 (dt, *J* = 9.5, 6.3 Hz, 1H), 3.84 (s, 3H), 3.41 (s, 3H), 2.86 (dddd, *J* = 37.6, 14.5, 9.4, 6.4 Hz, 2H), 2.12 (dddt, *J* = 26.1, 15.6, 13.5, 6.4 Hz, 2H). ^{13}C NMR (100 MHz, $CDCl_3$) δ 167.0, 162.7, 156.3, 156.3, 151.5, 149.5, 149.2, 137.1, 129.0, 126.0, 124.1, 123.9, 121.4, 121.2, 114.0, 81.3, 56.2, 55.6, 36.4, 31.0. HRMS (ESI, m/z) calculated for $C_{22}H_{24}N_3O_3^+$: 378.1818 , found : 378.1818, calculated for $C_{22}H_{24}N_3O_3Na^+$: 400.1637 , found : 400.1635

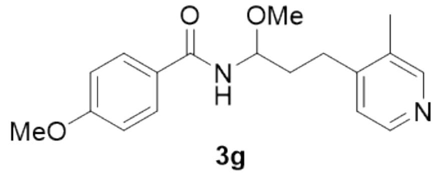

3g

4-methoxy-N-(1-methoxy-3-(3-methylpyridin-4-yl)propyl)benzamide (3g). Yield : 66% (20.5 mg). White solid. ^{1}H NMR (400 MHz, $CDCl_3$) δ 8.32 (s, 2H), 8.05 – 7.49 (m, 2H), 7.06 (d, *J* = 5.0 Hz, 1H), 6.93 (d, *J* = 8.9 Hz, 2H), 6.30 (d, *J* = 9.5 Hz, 1H), 5.54 – 5.17 (m, 1H), 3.85 (s, 3H), 3.41 (s, 3H), 2.74 (dddd, *J* = 39.1, 14.7, 9.7, 6.2 Hz, 2H), 2.27 (s, 3H), 1.98 (ddtd, *J* = 19.6, 15.7, 13.6, 6.2 Hz, 2H). ^{13}C NMR (100 MHz, $CDCl_3$ δ 167.1, 162.7, 150.8, 148.5, 147.7, 131.8, 129.0, 126.0, 123.5, 114.0, 81.3, 56.2, 55.6, 35.3, 28.0, 16.2. HRMS (ESI, m/z) calculated for $C_{18}H_{23}N_2O_3^+$: 315.1709 , found : 315.1709

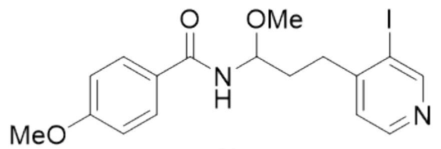

3h

N-(3-(3-iodopyridin-4-yl)-1-methoxypropyl)-4-methoxybenzamide (3h). Yield : 50% (21.3 mg). Yellow solid. ^{1}H NMR (400 MHz, $CDCl_3$) δ 8.89 (s, 1H), 8.43 (d, *J* = 4.9 Hz, 1H), 7.78 (d, *J* = 8.4 Hz, 2H), 7.22 (d, *J* = 4.9 Hz, 1H), 6.97 (d, *J* = 8.6 Hz, 2H), 6.26 (d, *J* = 9.5 Hz, 1H), 5.43 (dt, *J* = 9.8, 6.3 Hz, 1H), 3.89 (s, 3H), 3.44 (s, 3H), 3.02 – 2.59 (m, 2H), 2.03 (dddd, *J* = 23.5, 19.9, 13.9, 6.3 Hz, 2H). ^{13}C NMR (100 MHz, $CDCl_3$) δ 167.1, 162.8, 157.7, 152.8, 149.2, 129.0, 126.0, 125.1, 114.1, 100.1, 81.0, 56.2, 55.6, 35.7, 35.2. HRMS (ESI, m/z) calculated for $C_{17}H_{20}N_2O_3I^+$: 427.0519 , found : 427.0518, calculated for $C_{17}H_{19}N_2O_3NaI^+$: 449.0338 , found : 449.0337.

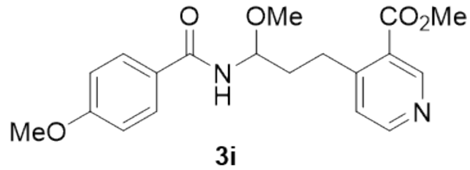

3i

methyl 4-(3-methoxy-3-(4-methoxybenzamido)propyl)nicotinate (3i). Yield : 51% (18.3 mg). Colorless oil. ^{1}H NMR (400 MHz, $CDCl_3$) δ 9.10 (s, 1H), 8.60 (d, *J* = 5.1 Hz, 1H), 8.13 – 7.75 (m, 2H), 7.22 (d, *J* = 5.1 Hz, 1H), 7.06 – 6.90 (m, 2H), 6.80 (d, *J* = 9.2 Hz, 1H), 5.40 (dt, *J* = 9.2, 6.1 Hz, 1H), 3.93 (s, 3H), 3.86 (s, 3H), 3.42 (s, 3H), 3.15 (ddd, *J* = 12.6, 9.1, 6.8 Hz, 1H), 3.01 (ddd, *J* = 12.7, 9.3, 6.8 Hz, 1H), 2.04 (qd, *J* = 6.6, 2.9 Hz, 2H). ^{13}C NMR (100 MHz, $CDCl_3$) δ 167.3, 166.5, 162.7, 153.0, 152.7, 152.2, 129.2, 126.1, 125.9, 125.1, 113.9, 81.0, 56.1, 55.6, 52.4, 36.3, 29.6. HRMS (ESI, m/z) calculated for $C_{19}H_{23}N_2O_5^+$: 359.1607 , found : 359.1608, calculated for $C_{19}H_{22}N_2O_5Na^+$: 381.1426, found : 381.1427.

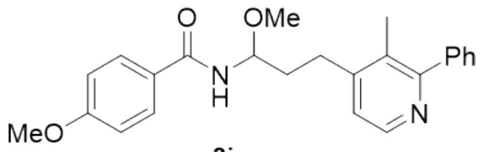

3j

4-methoxy-N-(1-methoxy-3-(3-methyl-2-phenylpyridin-4-yl)propyl)benzamide (3j). Yield : 79% (30.7 mg). White solid. ^{1}H NMR (500 MHz, $CDCl_3$) δ 8.41 (t, *J* = 3.6 Hz, 1H), 7.80 – 7.65 (m, 2H), 7.41 (q, *J* = 2.2, 1.8 Hz, 4H), 7.39 – 7.31 (m, 1H), 7.08 (t, *J* = 3.9 Hz, 1H), 6.99 – 6.77 (m, 3H), 6.37 (s, 1H), 5.43 (dt, *J* = 9.0, 6.0 Hz, 1H), 3.84 (d, *J* = 3.1 Hz, 3H), 3.42 (d, *J* = 2.4 Hz, 3H), 2.96 – 2.83 (m, 1H), 2.78 (tq, *J* = 12.0, 3.9, 3.5 Hz, 1H), 2.25 (d, *J* = 2.8 Hz, 3H), 2.08 (ddt, *J* = 12.8, 9.5, 6.3 Hz, 1H), 1.99 (ddq, *J* = 13.1, 10.9, 3.3 Hz, 1H). ^{13}C NMR (125 MHz, $CDCl_3$) δ 167.1, 162.7, 159.5, 149.7, 146.7, 141.2, 129.4, 129.2, 129.0, 128.2, 127.9, 126.0, 122.6, 114.0, 81.4, 56.2, 55.6, 35.4, 28.7, 16.0. HRMS (ESI, m/z) calculated for $C_{24}H_{27}N_2O_3^+$: 391.2022 , found : 391.2023

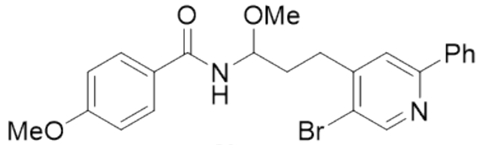

3k

N-(3-(5-bromo-2-phenylpyridin-4-yl)-1-methoxypropyl)-4-methoxybenzamide (3k). Yield : 42% (19.1 mg). White solid. ^{1}H

NMR (400 MHz, $CDCl_3$) δ 8.63 (s, 1H), 8.02 – 7.81 (m, 2H), 7.68 (d, J = 8.8 Hz, 2H), 7.54 (s, 1H), 7.45 – 7.27 (m, 3H), 7.02 – 6.51 (m, 2H), 6.18 (d, J = 9.5 Hz, 1H), 5.37 (dt, J = 9.5, 6.3 Hz, 1H), 3.78 (s, 3H), 3.36 (s, 3H), 2.86 (qdd, J = 13.8, 9.3, 6.5 Hz, 2H), 2.32 – 1.83 (m, 2H). ^{13}C NMR (100 MHz, $CDCl_3$) δ 167.1, 162.8, 156.7, 151.8, 149.9, 138.4, 129.4, 128.98, 128.95, 127.0, 125.9, 122.3, 121.6, 114.1, 81.2, 56.2, 55.6, 35.0, 31.5. HRMS (ESI, m/z) calculated for $C_{23}H_{24}N_2O_3Br^+$: 455.0970 , found : 455.0970

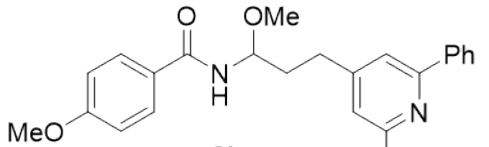

3l

4-methoxy-N-(1-methoxy-3-(2-methyl-6-phenylpyridin-4-yl)propyl)benzamide (3l). Yield : 87% (33.8 mg). White solid. ^1H NMR (400 MHz, $CDCl_3$) δ 7.97 – 7.88 (m, 2H), 7.76 – 7.64 (m, 2H), 7.49 – 7.40 (m, 2H), 7.40 – 7.36 (m, 1H), 7.36 – 7.33 (m, 1H), 6.93 (d, J = 1.4 Hz, 1H), 6.92 – 6.87 (m, 2H), 6.29 (d, J = 9.6 Hz, 1H), 5.40 (dt, J = 9.6, 6.2 Hz, 1H), 3.83 (s, 3H), 3.40 (s, 3H), 2.82 (ddd, J = 14.0, 9.4, 6.2 Hz, 1H), 2.72 (ddd, J = 14.0, 9.2, 6.5 Hz, 1H), 2.56 (s, 3H), 2.18 – 2.07 (m, 1H), 2.07 – 1.96 (m, 1H). δ 167.0, 162.7, 158.6, 157.4, 151.2, 139.8, 128.9, 128.8, 128.7, 127.2, 125.9, 121.9, 118.1, 114.0, 81.3, 56.1, 55.5, 36.4, 30.8, 24.7. HRMS (ESI, m/z) calculated for $C_{24}H_{27}N_2O_3^+$: 391.2022 , found : 391.2023.

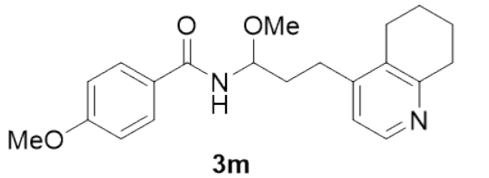

3m

4-methoxy-N-(1-methoxy-3-(5,6,7,8-tetrahydroquinolin-4-yl)propyl)benzamide (3m). Yield : 86% (30.5 mg). White solid. ^1H NMR (400 MHz, $CDCl_3$) δ 8.24 (d, J = 4.9 Hz, 1H), 8.00 – 7.54 (m, 2H), 6.90 (dd, J = 9.1, 6.8 Hz, 3H), 6.31 (d, J = 9.6 Hz, 1H), 5.39 (dt, J = 9.5, 6.2 Hz, 1H), 3.84 (s, 3H), 3.40 (s, 3H), 2.88 (d, J = 6.4 Hz, 2H), 2.78 – 2.53 (m, 4H), 2.06 – 1.89 (m, 2H), 1.80 (q, J = 6.3 Hz, 4H). ^{13}C NMR (100 MHz, $CDCl_3$) δ 167.0, 162.7, 157.4, 148.6, 146.6, 130.7, 129.0, 126.0, 121.2, 114.0, 81.4, 56.1, 55.6, 35.2, 33.2, 27.4, 25.5, 22.9, 22.8. HRMS (ESI, m/z) calculated for $C_{21}H_{27}N_2O_3^+$: 355.2022 , found : 355.2022

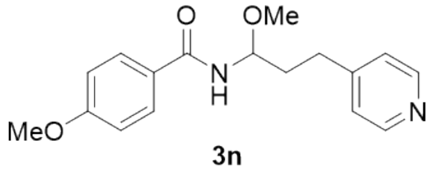

3n

4-methoxy-N-(1-methoxy-3-(pyridin-4-yl)propyl)benzamide (3n). Yield : 52% (15.4 mg). Yellow solid. ^1H NMR (400 MHz, $CDCl_3$) δ 8.49 (d, J = 6.0 Hz, 1H), 7.71 (d, J = 8.9 Hz, 2H), 7.13 (d, J = 6.1 Hz, 1H), 6.93 (d, J = 8.9 Hz, 2H), 6.20 (d, J = 9.6 Hz, 1H), 5.36 (ddd, J = 9.6, 6.8, 5.7 Hz, 1H), 3.85 (s, 3H), 3.40 (s, 3H), 2.81 (ddd, J = 14.1, 9.5, 6.2 Hz, 1H), 2.72 (ddd, J = 14.3, 9.2, 6.6 Hz, 1H), 2.08 (ddt, J = 12.3, 8.4, 6.1 Hz, 1H). ^{13}C NMR (125 MHz, $CDCl_3$) δ 166.9, 162.6, 150.5, 149.7, 128.8, 125.8, 123.9, 113.9, 81.1, 56.1, 55.5, 36.3, 30.7. HRMS (ESI, m/z) calculated for $C_{17}H_{21}N_2O_3^+$: 301.1552 , found : 301.1553, $C_{17}H_{20}N_2O_3Na^+$: 323.1372, found : 323.1371.

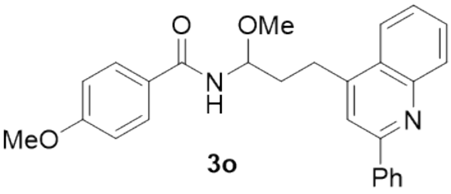

3o

4-methoxy-N-(1-methoxy-3-(2-phenylquinolin-4-yl)propyl)benzamidebenzamide (3o). Yield : 78% (37.9 mg). White solid. ^{1}H NMR (400 MHz, CDCl$_3$) δ 8.18 (dd, *J* = 8.5, 1.2 Hz, 1H), 8.15 – 8.07 (m, 1H), 8.02 (d, *J* = 8.3 Hz, 1H), 7.78 – 7.65 (m, 4H), 7.62 – 7.39 (m, 4H), 6.95 – 6.66 (m, 2H), 6.38 (dd, *J* = 10.0, 5.1 Hz, 1H), 5.51 (ddd, *J* = 9.6, 6.9, 5.4 Hz, 1H), 3.81 (d, *J* = 1.1 Hz, 3H), 3.44 (s, 3H), 3.34 (ddd, *J* = 15.3, 9.9, 5.9 Hz, 1H), 3.20 (ddd, *J* = 14.8, 9.8, 6.1 Hz, 1H), 2.34 – 2.20 (m, 1H), 2.20 – 2.04 (m, 1H). ^{13}C NMR (100 MHz, CDCl$_3$) δ 167.1, 162.7, 157.2, 148.6, 147.9, 139.8, 130.6, 129.5, 129.3, 129.0, 128.9, 127.6, 126.4, 126.3, 125.9, 123.4, 118.9, 114.0, 81.5, 56.2, 55.5, 36.2, 28.1. HRMS (ESI, m/z) calculated for $C_{27}H_{27}N_2O_3^+$: 427.2022 , found : 427.2022.

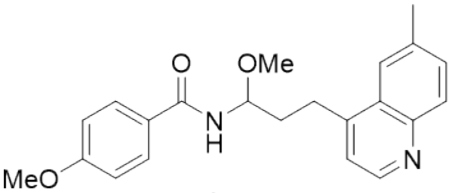

3p

4-methoxy-N-(1-methoxy-3-(6-methylquinolin-4-yl)propyl)benzamide (3p). Yield : 58% (20.7 mg). Yellow solid. ^{1}H NMR (400 MHz, CDCl$_3$) δ 8.71 (d, *J* = 4.4 Hz, 1H), 7.99 (d, *J* = 8.6 Hz, 1H), 7.78 (s, 1H), 7.52 (dd, *J* = 8.7, 1.9 Hz, 2H), 7.20 (d, *J* = 4.4 Hz, 1H), 7.06 – 6.63 (m, 2H), 6.30 (d, *J* = 9.4 Hz, 1H), 5.78 – 5.11 (m, 1H), 3.84 (s, 4H), 3.44 (s, 3H), 3.20 (dddd, *J* = 44.5, 14.7, 9.5, 6.2 Hz, 2H), 2.54 (s, 3H), 2.16 (dddd, *J* = 32.7, 13.8, 9.3, 7.3 Hz, 2H). ^{13}C NMR (100 MHz, CDCl$_3$) δ 167.1, 162.7, 149.4, 147.0, 146.6, 136.5, 131.5, 130.1, 129.0, 127.4, 126.0, 122.4, 120.9, 114.0, 81.4, 56.2, 55.6, 36.1, 27.6, 22.1. HRMS (ESI, m/z) calculated for $C_{22}H_{25}N_2O_3^+$: 365.1865 , found : 365.1865

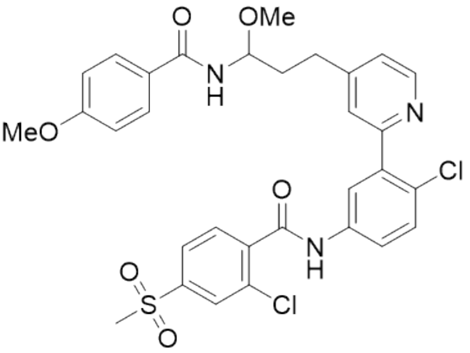

3q

2-chloro-N-(4-chloro-3-(4-(3-methoxy-3-(4-methoxybenzamido)propyl)pyridin-2-yl)phenyl)-4-(methylsulfonyl)benzamidebenzamide (3q). Yield : 75% (65.1 mg). White solid. ^{1}H NMR (500 MHz, CDCl$_3$) δ 10.61 (s, 1H), 8.18 (dd, *J* = 8.8, 2.6 Hz, 1H), 8.14 (d, *J* = 5.1 Hz, 1H), 7.88 – 7.74 (m, 3H), 7.68 (d, *J* = 2.7 Hz, 1H), 7.58 (dd, *J* = 7.9, 1.7 Hz, 1H), 7.52 (d, *J* = 1.7 Hz, 1H), 7.48 (d, *J* = 8.8 Hz, 1H), 7.37 (d, *J* = 7.9 Hz, 1H), 7.03 – 6.86 (m, 3H), 6.66 (d, *J* = 9.5 Hz, 1H), 5.35 (ddd, *J* = 9.4, 7.0, 5.6 Hz, 1H), 3.86 (s, 3H), 3.41 (s, 3H), 2.96 (s, 3H), 2.82 (ddd, *J* = 15.4, 10.2, 5.7 Hz, 1H), 2.75 (s, 1H), 2.09 (dddd, *J* = 13.1, 10.1, 7.1, 5.8 Hz, 1H), 2.04 – 1.85 (m, 2H). ^{13}C NMR (125 MHz, CDCl$_3$) δ 167.3, 164.2, 162.6, 155.5, 151.5, 148.7, 142.2, 141.1, 138.2, 137.6, 132.3, 131.1, 129.7, 129.2, 128.7, 127.0, 126.0, 125.6, 123.1, 122.9, 121.8, 113.9, 80.9, 56.0, 55.6, 44.4, 36.0, 30.8. HRMS (ESI, m/z) calculated for $C_{31}H_{30}N_3O_6SCl_2^+$: 642.1232 , found : 642.1230.

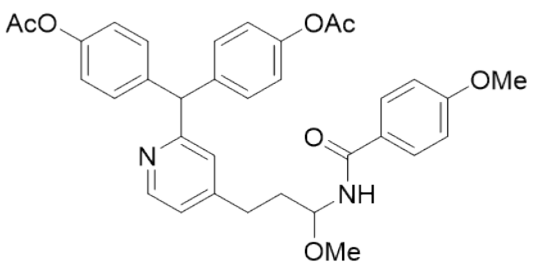

3r

((4-(3-methoxy-3-(4-methoxybenzamido)propyl)pyridin-2-yl)methylene)bis(4,1-phenylene) diacetate (3r). Yield : 70% (40.9 mg). White solid. ^{1}H NMR (400 MHz, CDCl$_3$) δ 8.45 (d, *J* = 5.1 Hz, 1H), 7.70 (d, *J* = 8.7 Hz, 2H), 7.15 (d, *J* = 8.4 Hz, 4H), 7.13 – 6.95 (m, 5H), 6.96 – 6.81 (m, 3H), 6.27 (d, *J* = 9.6 Hz, 1H), 5.60 (s, 1H), 5.32 (dt, *J* = 9.3, 6.1 Hz, 1H), 3.84 (s, 3H), 3.35 (s, 3H), 2.70 (dddd, *J* = 38.0, 14.6, 9.4, 6.2 Hz, 2H), 2.26 (s, 6H), 2.03 – 1.86 (m, 2H). ^{13}C NMR (100 MHz, CDCl$_3$) δ 169.6, 167.0, 162.8, 162.7, 151.1, 149.7, 149.4, 140.1, 130.4, 129.0, 126.0, 124.0, 121.9, 121.6, 114.0, 81.1, 58.1, 56.1, 55.6, 36.3, 30.8, 29.8, 21.3. HRMS (ESI, m/z) calculated for $C_{34}H_{35}N_2O_7^+$: 583.2444, found : 583.2444, calculated for $C_{34}H_{34}N_2O_7Na^+$: 605.2264, found : 605.2264.

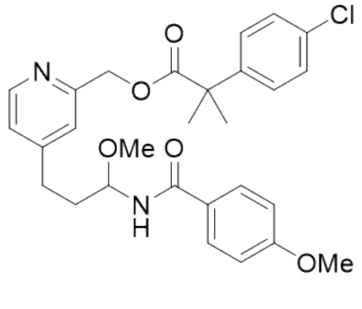

3s

(4-(3-methoxy-3-(4-methoxybenzamido)propyl)pyridin-2-yl)methyl 2-(4-chlorophenyl)-2-methylpropanoate (3s). Yield : 70% (36.6 mg). Colorless oil. ^{1}H NMR (400 MHz, CDCl$_3$) δ 8.42 (d, *J* = 5.0 Hz, 1H), 7.71 (d, *J* = 8.8 Hz, 2H), 7.13 (d, *J* = 8.9 Hz, 2H), 7.05 (dd, *J* = 5.0, 1.7 Hz, 1H), 6.99 – 6.87 (m, 3H), 6.77 (d, *J* = 8.9 Hz, 2H), 6.20 (d, *J* = 9.5 Hz, 1H), 5.43 – 5.30 (m, 1H), 5.25 (d, *J* = 1.5 Hz, 2H), 3.85 (s, 4H), 3.39 (s, 3H), 2.68 (dddd, *J* = 39.4, 14.5, 9.4, 6.3 Hz, 2H), 2.03 – 1.86 (m, 1H), 1.63 (s, 6H). ^{13}C NMR (100 MHz, CDCl$_3$) δ 173.7, 167.0, 162.7, 155.4, 154.2, 151.4, 149.5, 129.3, 129.0, 127.2, 125.9, 123.2, 121.9, 120.4, 114.0, 81.1, 79.6, 67.6, 56.1, 55.6, 36.3, 30.8, 29.8, 25.54, 25.47.HRMS (ESI, m/z) calculated for $C_{28}H_{32}N_2O_6Cl^+$: 527.1949, found : 527.1949, calculated for $C_{28}H_{31}N_2O_6NaCl^+$: 549.1768, found : 547.1767.

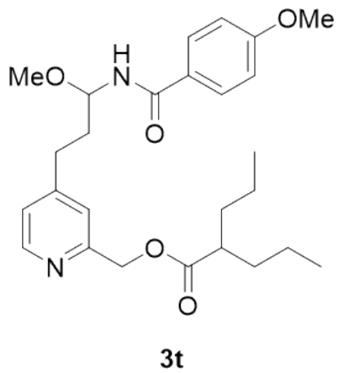

3t

(4-(3-methoxy-3-(4-methoxybenzamido)propyl)pyridin-2-yl)methyl 2-propylpentanoate (3t). Yield : 72% (32.8 mg). Colorless oil. ^{1}H NMR (400 MHz, CDCl$_3$) δ 8.45 (d, *J* = 5.1 Hz, 1H), 7.74 – 7.59 (m, 2H), 7.18 (d, *J* = 1.7 Hz, 1H), 7.06 (dd, *J* = 5.1, 1.7 Hz, 1H), 6.92 (d, *J* = 8.8 Hz, 2H), 6.21 (d, *J* = 9.5 Hz, 1H), 5.36 (dt, *J* = 9.6, 6.3 Hz, 1H), 5.18 (s, 2H), 3.85 (s, 3H), 3.39 (s, 3H), 2.77 (dddd, *J* = 39.1, 14.5, 9.5, 6.3 Hz, 2H), 2.47 (tt, *J* = 8.8, 5.4 Hz, 1H), 2.13 – 1.89 (m, 2H), 1.70 – 1.52 (m, 2H), 1.52 – 1.38 (m, 2H), 1.36 – 1.10 (m, 4H), 0.88 (t, *J* = 7.3 Hz, 6H). ^{13}C NMR (100 MHz, CDCl$_3$) δ 176.3, 167.0, 162.8, 156.4, 151.3, 149.6, 128.9, 125.9,

123.0, 121.8, 114.0, 81.2, 66.5, 56.2, 55.6, 45.3, 36.4, 34.7, 30.9, 29.8, 20.7, 14.1. HRMS (ESI, m/z) calculated for $C_{26}H_{37}N_2O_5^+$: 457.2702 , found : 457.2704, calculated for $C_{26}H_{36}N_2O_5Na^+$: 479.2522 , found : 479.2523.

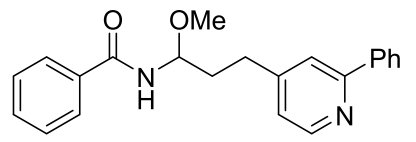

4a

N-(1-methoxy-3-(2-phenylpyridin-4-yl)propyl)benzamide (4a). Yield : 50% (17.2 mg). Colorless oil. ^{1}H NMR (500 MHz, $CDCl_3$) δ 8.58 (d, *J* = 5.0 Hz, 1H), 7.99 – 7.93 (m, 2H), 7.78 – 7.70 (m, 2H), 7.58 (s, 1H), 7.56 – 7.50 (m, 1H), 7.50 – 7.38 (m, 5H), 7.10 (dd, *J* = 5.1, 1.6 Hz, 1H), 6.24 (d, *J* = 9.6 Hz, 1H), 5.46 – 5.38 (m, 1H), 3.43 (s, 3H), 2.89 (ddd, *J* = 15.2, 9.4, 6.1 Hz, 1H), 2.85 – 2.76 (m, 1H), 2.20 – 2.01 (m, 2H). ^{13}C NMR (125 MHz, $CDCl_3$) δ 167.5, 157.9, 151.0, 149.9, 139.5, 133.8, 132.2, 129.1, 128.89, 128.85, 127.12, 127.05, 122.4, 120.9, 81.4, 56.3, 36.5, 31.0. HRMS (ESI, m/z) calculated for $C_{22}H_{23}N_2O_2^+$: 347.1760, found : 347.1761

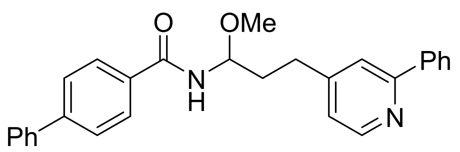

4b

N-(1-methoxy-3-(2-phenylpyridin-4-yl)propyl)-[1,1'-biphenyl]-4-carboxamide (4b). Yield : 57% (24.1 mg). White solid. ^{1}H NMR (500 MHz, $CDCl_3$) δ 8.59 (d, *J* = 5.0 Hz, 1H), 8.00 – 7.93 (m, 2H), 7.84 – 7.78 (m, 2H), 7.69 – 7.63 (m, 2H), 7.62 – 7.57 (m, 2H), 7.46 (ddd, *J* = 7.7, 6.7, 5.4 Hz, 4H), 7.42 – 7.36 (m, 2H), 7.11 (dd, *J* = 5.1, 1.6 Hz, 1H), 6.30 (s, 1H), 5.45 (ddd, *J* = 9.6, 6.7, 5.7 Hz, 1H), 3.45 (s, 3H), 2.86 (dddd, *J* = 46.7, 14.5, 9.3, 6.3 Hz, 2H), 2.22 – 2.03 (m, 2H). ^{13}C NMR (125 MHz, $CDCl_3$) δ 167.2, 157.9, 151.1, 149.9, 145.0, 140.0, 139.5, 132.4, 129.09, 129.05, 128.8, 128.3, 127.6, 127.5, 127.4, 127.1, 122.4, 121.0, 81.4, 56.3, 36.5, 31.0. HRMS (ESI, m/z) calculated for $C_{28}H_{27}N_2O_2^+$: 423.2073, found : 423.2075

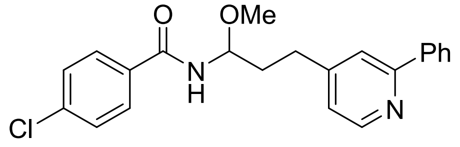

4c

4-chloro-N-(1-methoxy-3-(2-phenylpyridin-4-yl)propyl)benzamide (4c). Yield : 50% (19.0 mg). White solid. ^{1}H NMR (500 MHz, $CDCl_3$) δ 8.57 (dd, *J* = 5.1, 0.8 Hz, 1H), 7.97 – 7.91 (m, 2H), 7.69 – 7.62 (m, 2H), 7.56 (dd, *J* = 1.7, 0.8 Hz, 1H), 7.49 – 7.43 (m, 2H), 7.43 – 7.36 (m, 3H), 7.08 (dd, *J* = 5.0, 1.7 Hz, 1H), 6.29 (d, *J* = 9.4 Hz, 1H), 5.39 (ddd, *J* = 9.5, 6.7, 5.7 Hz, 1H), 3.41 (s, 3H), 2.88 (ddd, *J* = 15.1, 9.3, 6.2 Hz, 1H), 2.78 (ddd, *J* = 14.2, 9.2, 6.6 Hz, 1H), 2.13 (ddt, *J* = 13.1, 9.2, 6.4 Hz, 1H), 2.05 (dddd, *J* = 13.7, 9.4, 6.6, 5.7 Hz, 1H). ^{13}C NMR (125 MHz, $CDCl_3$) δ 166.5, 157.9, 151.0, 149.9, 139.4, 138.5, 132.1, 129.10, 129.09, 128.8, 128.5, 127.1, 122.4, 120.9, 81.5, 56.3, 36.4, 31.0. HRMS (ESI, m/z) calculated for $C_{22}H_{22}N_2O_2Cl^+$: 381.1370, found : 381.1373

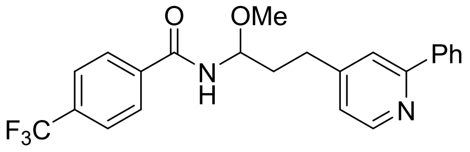

4d

N-(1-methoxy-3-(2-phenylpyridin-4-yl)propyl)-4-(trifluoromethyl)benzamide (4d). Yield : 49% (20.1 mg). White solid. ^{1}H NMR (500 MHz, $CDCl_3$) δ 8.58 (dd, *J* = 5.1, 2.0 Hz, 1H), 7.97 – 7.91 (m, 2H), 7.82 (d, *J* = 8.1 Hz, 2H), 7.74 – 7.64 (m, 2H), 7.58 – 7.54 (m, 1H), 7.45 (ddd, *J* = 7.8, 6.6, 1.7 Hz, 2H), 7.42 – 7.37 (m, 1H), 7.09 (dt, *J* = 5.1, 1.8 Hz, 1H), 6.41 (d, *J* = 17.0 Hz, 1H),

5.41 (dt, J = 9.5, 6.2 Hz, 1H), 3.43 (s, 3H), 2.94 – 2.84 (m, 1H), 2.79 (dtd, J = 14.3, 7.8, 2.1 Hz, 1H), 2.21 – 2.02 (m, 2H). ^{13}C NMR (125 MHz, $CDCl_3$) δ 166.3, 157.9, 151.0, 149.9, 139.4, 137.1, 134.0, 133.7, 129.1, 128.9, 127.6, 127.1, 125.93, 125.90, 125.87, 125.84, 122.4, 120.9, 81.7, 56.4, 36.3, 30.9. ^{19}F NMR (471 MHz, $CDCl_3$) δ -63.01. HRMS (ESI, m/z) calculated for $C_{23}H_{22}N_2O_2F_3^+$: 415.1633, found : 415.1632

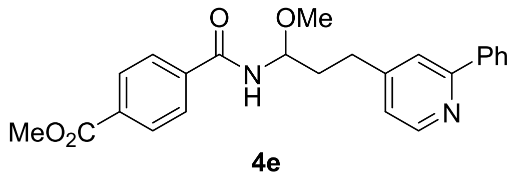

4e

methyl 4-((1-methoxy-3-(2-phenylpyridin-4-yl)propyl)carbamoyl)benzoate (4e). Yield : 57% (22.9 mg). Yellow oil. ^{1}H NMR (500 MHz, $CDCl_3$) δ 8.56 (d, J = 5.0 Hz, 1H), 8.10 – 8.04 (m, 2H), 7.94 (dd, J = 7.0, 1.5 Hz, 2H), 7.82 – 7.76 (m, 2H), 7.56 (dd, J = 1.7, 0.8 Hz, 1H), 7.47 – 7.42 (m, 2H), 7.42 – 7.37 (m, 1H), 7.08 (dd, J = 5.2, 1.7 Hz, 1H), 6.48 (d, J = 9.5 Hz, 1H), 5.40 (ddd, J = 9.5, 6.7, 5.5 Hz, 1H), 3.93 (s, 3H), 3.42 (s, 3H), 2.88 (ddd, J = 15.2, 9.5, 6.1 Hz, 1H), 2.79 (ddd, J = 14.1, 9.3, 6.5 Hz, 1H), 2.14 (ddt, J = 13.2, 9.3, 6.4 Hz, 1H), 2.06 (dddd, J = 13.8, 9.4, 6.6, 5.6 Hz, 1H). ^{13}C NMR (125 MHz, $CDCl_3$) δ 166.7, 166.3, 157.9, 151.0, 149.9, 149.8, 139.4, 137.7, 133.3, 130.0, 129.1, 128.8, 127.1, 122.4, 120.9, 81.6, 56.4, 52.5, 36.3, 31.0. HRMS (ESI, m/z) calculated for $C_{24}H_{25}N_2O_4^+$: 405.1814, found : 405.1815

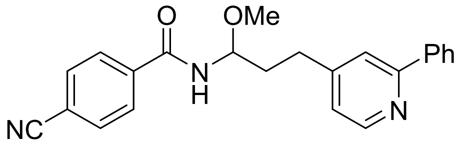

4f

4-cyano-N-(1-methoxy-3-(2-phenylpyridin-4-yl)propyl)benzamide (4f). Yield : 52% (19.2 mg). Colorless oil. ^{1}H NMR (500 MHz, $CDCl_3$) δ 8.6 (d, J = 5.0 Hz, 1H), 8.0 – 7.9 (m, 2H), 7.8 – 7.7 (m, 2H), 7.7 – 7.7 (m, 2H), 7.6 (s, 1H), 7.5 – 7.4 (m, 2H), 7.4 – 7.4 (m, 1H), 7.1 (dd, J = 5.0, 1.6 Hz, 1H), 6.3 (d, J = 9.4 Hz, 1H), 5.4 (ddd, J = 9.4, 6.8, 5.6 Hz, 1H), 3.4 (s, 3H), 2.9 (ddd, J = 14.9, 9.0, 6.3 Hz, 1H), 2.8 (ddd, J = 14.6, 8.9, 6.8 Hz, 1H), 2.2 – 2.0 (m, 2H). ^{13}C NMR (125 MHz, $CDCl_3$) δ 165.7, 157.9, 150.9, 150.0, 139.4, 137.6, 132.7, 129.2, 128.9, 127.7, 127.1, 122.4, 120.9, 117.9, 115.8, 81.8, 56.5, 36.3, 30.9. HRMS (ESI, m/z) calculated for $C_{23}H_{22}N_3O_2^+$: 372.1712, found : 372.1713

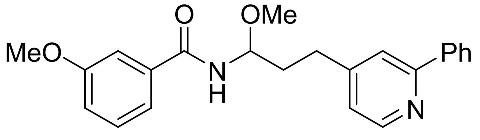

4g

3-methoxy-N-(1-methoxy-3-(2-phenylpyridin-4-yl)propyl)benzamide (4g). Yield : 65% (24.4 mg). Colorless oil. ^{1}H NMR (500 MHz, $CDCl_3$) δ 8.57 (d, J = 5.0 Hz, 1H), 7.99 – 7.91 (m, 2H), 7.55 (s, 1H), 7.49 – 7.42 (m, 2H), 7.42 – 7.37 (m, 1H), 7.36 (dd, J = 2.7, 1.6 Hz, 1H), 7.32 (t, J = 7.9 Hz, 1H), 7.28 – 7.21 (m, 1H), 7.10 – 7.02 (m, 2H), 6.41 (d, J = 9.6 Hz, 1H), 5.40 (ddd, J = 9.6, 6.7, 5.7 Hz, 1H), 3.82 (s, 3H), 3.42 (s, 3H), 2.82 (dddd, J = 44.5, 14.2, 9.5, 6.2 Hz, 2H), 2.12 (ddt, J = 13.1, 9.5, 6.4 Hz, 1H), 2.04 (ddt, J = 13.7, 9.6, 6.2 Hz, 1H). ^{13}C NMR (125 MHz, $CDCl_3$) δ 167.4, 160.0, 157.8, 151.1, 149.8, 139.4, 135.2, 129.8, 129.0, 128.8, 127.1, 122.4, 120.9, 118.7, 118.3, 112.6, 81.3, 56.2, 55.5, 36.4, 31.0, 29.8. HRMS (ESI, m/z) calculated $C_{23}H_{25}N_2O_3^+$: 377.1865, found : 377.1866

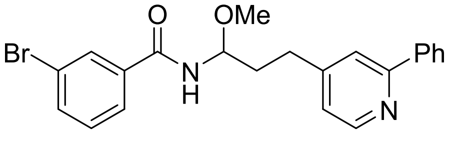

4h

3-bromo-N-(1-methoxy-3-(2-phenylpyridin-4-yl)propyl)benzamide (4h). Yield : 50% (21.1 mg). Colorless oil. ^{1}H NMR (500 MHz, $CDCl_3$) δ 8.59 (d, J = 4.9 Hz, 1H), 8.01 – 7.93 (m, 2H), 7.89 (s, 1H), 7.64 (dd, J = 13.4, 8.0 Hz, 2H), 7.57 (s, 1H), 7.47 (t, J = 7.6 Hz, 2H), 7.41 (d, J = 7.3 Hz, 1H), 7.31 (t, J = 7.8 Hz, 1H), 7.09 (d, J = 4.4 Hz, 1H), 6.18 (d, J = 9.5 Hz, 1H), 5.39 (q, J = 7.3 Hz, 1H), 3.43 (s, 3H), 2.94 – 2.84 (m, 1H), 2.80 (dt, J = 14.8, 7.8 Hz, 1H), 2.19 – 2.11 (m, 1H), 2.10 – 2.02 (m, 1H). ^{13}C NMR (125 MHz, $CDCl_3$) δ 166.1, 157.9, 150.9, 150.0, 139.5, 135.8, 135.1, 130.43, 130.38, 129.1, 128.9, 127.1, 125.5, 123.1, 122.4, 120.9, 81.6, 56.4, 36.5, 31.0. HRMS (ESI, m/z) calculated for $C_{22}H_{22}N_2O_2Br^+$: 425.0865, found : 425.0866

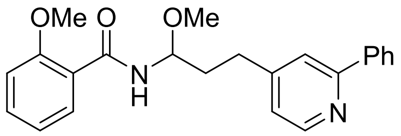

4i

2-methoxy-N-(1-methoxy-3-(2-phenylpyridin-4-yl)propyl)benzamide (4i). Yield : 60% (22.5 mg). Colorless oil. ^{1}H NMR (500 MHz, $CDCl_3$) δ 8.56 (d, J = 5.0 Hz, 1H), 8.20 (dd, J = 7.8, 1.9 Hz, 1H), 8.01 (d, J = 9.4 Hz, 1H), 7.97 – 7.91 (m, 2H), 7.56 (s, 1H), 7.49 – 7.42 (m, 3H), 7.42 – 7.36 (m, 1H), 7.09 (ddt, J = 7.4, 5.2, 2.3 Hz, 2H), 6.96 (dd, J = 8.4, 1.0 Hz, 1H), 5.47 (dt, J = 9.4, 6.2 Hz, 1H), 3.92 (s, 3H), 3.43 (s, 3H), 2.83 (dddd, J = 38.6, 14.1, 9.6, 6.2 Hz, 2H), 2.10 (dddt, J = 47.8, 13.7, 9.8, 6.3 Hz, 2H). ^{13}C NMR (125 MHz, $CDCl_3$) δ 165.7, 157.69, 157.67, 151.3, 149.7, 139.5, 133.4, 132.6, 128.9, 128.8, 127.0, 122.4, 121.5, 120.9, 111.5, 81.0, 56.08, 56.06, 36.6, 31.0, 29.8. HRMS (ESI, m/z) calculated for $C_{23}H_{25}N_2O_3^+$: 377.1865, found : 377.1864

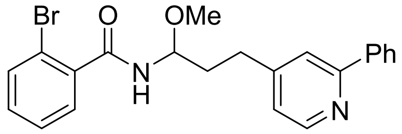

4j

2-bromo-N-(1-methoxy-3-(2-phenylpyridin-4-yl)propyl)benzamide (4j). Yield : 43% (18.2 mg). Ivory oil. ^{1}H NMR (500 MHz, $CDCl_3$) δ 8.59 (d, J = 5.0 Hz, 1H), 8.01 – 7.94 (m, 2H), 7.64 – 7.57 (m, 2H), 7.52 (dd, J = 7.6, 1.8 Hz, 1H), 7.49 – 7.44 (m, 2H), 7.43 – 7.39 (m, 1H), 7.37 (tt, J = 7.6, 1.9 Hz, 1H), 7.30 (td, J = 7.7, 1.8 Hz, 1H), 7.10 (dd, J = 5.1, 1.7 Hz, 1H), 6.19 (d, J = 9.9 Hz, 1H), 5.38 (ddd, J = 9.7, 6.8, 5.7 Hz, 1H), 3.51 (s, 3H), 2.94 – 2.79 (m, 2H), 2.18 – 2.00 (m, 2H). ^{13}C NMR (125 MHz, $CDCl_3$) δ 167.8, 157.9, 151.0, 149.9, 139.6, 137.5, 133.7, 131.7, 129.7, 129.0, 128.8, 127.8, 127.1, 122.4, 120.9, 119.1, 81.4, 56.5, 36.5, 31.0. HRMS (ESI, m/z) calculated for $C_{22}H_{22}N_2O_2Br^+$: 425.0865, found : 425.0866

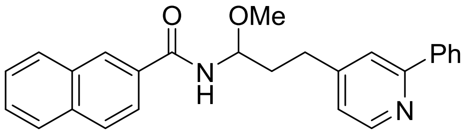

4k

N-(1-methoxy-3-(2-phenylpyridin-4-yl)propyl)-2-naphthamide (4k). Yield : 61% (24.2 mg). Colorless oil. ^{1}H NMR (500 MHz, $CDCl_3$) δ 8.57 (d, J = 5.0 Hz, 1H), 8.24 (s, 1H), 7.95 (d, J = 6.9 Hz, 2H), 7.92 – 7.84 (m, 3H), 7.80 (d, J = 8.5 Hz, 1H), 7.61 – 7.49 (m, 3H), 7.44 (t, J = 7.4 Hz, 2H), 7.39 (t, J = 7.2 Hz, 1H), 7.09 (d, J = 3.2 Hz, 1H), 6.57 – 6.51 (m, 1H), 5.48 (dt, J = 9.5, 6.2 Hz, 1H), 3.46 (s, 3H), 2.91 (ddd, J = 15.2, 9.4, 6.1 Hz, 1H), 2.81 (ddd, J = 14.5, 9.0, 6.4 Hz, 1H), 2.23 – 2.14 (m, 1H), 2.14 – 2.05 (m, 1H). ^{13}C NMR (125 MHz, $CDCl_3$) δ 167.7, 157.8, 151.1, 149.9, 139.5, 135.1, 132.6, 130.9, 129.1, 129.0, 128.81, 128.78, 128.1, 127.9, 127.7, 127.07, 127.05, 123.51, 123.48, 122.4, 120.9, 81.4, 56.3, 36.4, 31.0. HRMS (ESI, m/z) calculated for $C_{26}H_{25}N_2O_2^+$: 397.1916, found : 397.1916

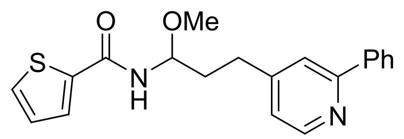

4l

N-(1-methoxy-3-(2-phenylpyridin-4-yl)propyl)thiophene-2-carboxamide (4l). Yield : 43% (15.1 mg) White solid. ^{1}H NMR (500 MHz, $CDCl_3$) δ 8.61 – 8.56 (m, 1H), 7.99 – 7.93 (m, 2H), 7.57 (s, 1H), 7.54 – 7.49 (m, 1H), 7.46 (q, *J* = 5.5 Hz, 3H), 7.41 (d, *J* = 7.2 Hz, 1H), 7.09 (d, *J* = 6.2 Hz, 2H), 6.04 (d, *J* = 9.6 Hz, 1H), 5.37 (q, *J* = 7.1 Hz, 1H), 3.43 (s, 3H), 2.89 (dt, *J* = 15.4, 7.7 Hz, 1H), 2.79 (dt, *J* = 14.8, 7.8 Hz, 1H), 2.14 (dt, *J* = 14.0, 7.2 Hz, 1H), 2.07 (p, *J* = 7.0 Hz, 1H). ^{13}C NMR (125 MHz, $CDCl_3$) δ 150.8, 149.8, 139.4, 130.8, 128.9, 128.7, 128.5, 127.8, 127.0, 122.2, 120.8, 81.2, 56.2, 36.3, 30.8. HRMS (ESI, m/z) calculated for $C_{20}H_{21}N_2O_2S^+$: 353.1324, found : 353.1324

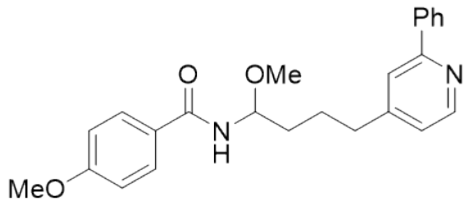

4m

4-methoxy-N-(1-methoxy-4-(2-phenylpyridin-4-yl)butyl)benzamide (4m). Yield : 71% (27.4 mg). White solid. ^{1}H NMR (500 MHz, $CDCl_3$) δ 8.55 (d, *J* = 5.0 Hz, 1H), 8.08 – 7.85 (m, 2H), 7.83 – 7.72 (m, 2H), 7.53 (d, *J* = 1.6 Hz, 1H), 7.50 – 7.43 (m, 2H), 7.43 – 7.33 (m, 1H), 7.04 (dd, *J* = 5.1, 1.6 Hz, 1H), 6.91 (d, *J* = 8.8 Hz, 2H), 6.26 (d, *J* = 9.6 Hz, 1H), 5.39 (dt, *J* = 9.6, 5.7 Hz, 1H), 3.83 (s, 3H), 3.38 (s, 3H), 2.71 (dq, *J* = 7.7, 3.2 Hz, 2H), 1.88 – 1.73 (m, 3H), 1.73 – 1.58 (m, 1H). ^{13}C NMR (125 MHz, $CDCl_3$) δ 167.1, 162.7, 157.7, 151.7, 149.7, 139.6, 128.98, 128.97, 128.8, 127.1, 126.0, 122.5, 121.0, 114.0, 81.5, 56.1, 55.6, 35.3, 35.0, 25.9. HRMS (ESI, m/z) calculated for $C_{24}H_{27}N_2O_3^+$: 391.2022 , found : 391.2023

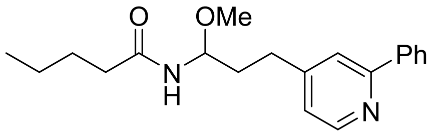

4n

N-(1-methoxy-3-(2-phenylpyridin-4-yl)propyl)pentanamide (4n). Yield : 51% (16.6 mg). Colorless oil. ^{1}H NMR (500 MHz, $CDCl_3$) δ 8.58 (d, *J* = 5.0 Hz, 1H), 7.96 (d, *J* = 7.1 Hz, 1H), 7.55 (s, 2H), 7.47 (t, *J* = 7.3 Hz, 1H), 7.41 (d, *J* = 6.9 Hz, 1H), 7.07 (d, *J* = 5.1 Hz, 1H), 5.59 (s, 1H), 5.19 (dt, *J* = 9.7, 6.2 Hz, 1H), 3.35 (s, 3H), 2.81 (ddd, *J* = 15.3, 9.7, 5.9 Hz, 1H), 2.72 (ddd, *J* = 14.5, 9.6, 6.4 Hz, 1H), 2.23 – 2.16 (m, 2H), 2.01 (ddt, *J* = 13.0, 9.5, 6.4 Hz, 1H), 1.91 (ddt, *J* = 13.1, 9.4, 5.8 Hz, 1H), 1.61 (p, *J* = 7.5 Hz, 2H), 1.41 – 1.29 (m, 2H), 0.95 – 0.87 (m, 3H). ^{13}C NMR (125 MHz, $CDCl_3$) δ 173.6, 157.8, 151.1, 149.8, 139.5, 129.1, 128.9, 127.1, 122.4, 120.9, 80.5, 56.0, 36.7, 36.4, 31.1, 27.8, 22.5, 13.9. HRMS (ESI, m/z) calculated for $C_{20}H_{27}N_2O_2^+$: 327.2073, found : 327.2073

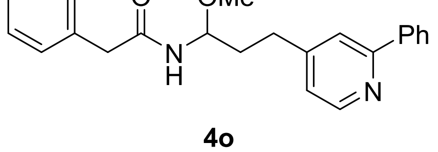

4o

N-(1-methoxy-3-(2-phenylpyridin-4-yl)propyl)-2-phenylacetamide (4o). Yield : 50% (17.9 mg). White solid. ^{1}H NMR (500 MHz, $CDCl_3$) δ 8.57 (d, *J* = 5.0 Hz, 1H), 7.96 (d, *J* = 8.5 Hz, 2H), 7.51 – 7.44 (m, 3H), 7.44 – 7.39 (m, 1H), 7.36 (td, *J* = 7.2, 1.7 Hz, 1H), 7.33 – 7.28 (m, 1H), 7.25 – 7.22 (m, 1H), 7.01 (d, *J* = 5.0 Hz, 1H), 5.46 (d, *J* = 9.7 Hz, 1H), 5.16 (q, *J* = 6.6 Hz, 1H), 3.59 (q, *J* = 16.0 Hz, 2H), 3.29 (s, 3H), 2.76 – 2.68 (m, 1H), 2.63 (dt, *J* = 14.7, 7.7 Hz, 1H), 1.98 – 1.87 (m, 1H), 1.82 (t, *J* = 7.3 Hz, 1H).

^{13}C NMR (125 MHz, $CDCl_3$) δ 171.4, 157.8, 151.0, 149.9, 139.6, 134.5, 129.5, 129.4, 129.1, 128.9, 127.8, 127.1, 122.4, 120.9, 80.8, 56.0, 44.2, 36.3, 30.9. HRMS (ESI, m/z) calculated for $C_{23}H_{25}N_2O_2^+$: 361.1916, found : 361.1917

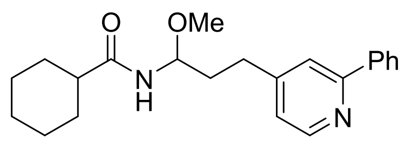

4p

N-(1-methoxy-3-(2-phenylpyridin-4-yl)propyl)cyclohexanecarboxamide (4p). Yield : 42% (14.8 mg). Colorless oil. ^{1}H NMR (500 MHz, $CDCl_3$) δ 8.58 (d, J = 5.0 Hz, 1H), 7.96 (d, J = 7.6 Hz, 2H), 7.54 (s, 1H), 7.46 (t, J = 7.5 Hz, 2H), 7.40 (t, J = 7.3 Hz, 1H), 7.06 (d, J = 5.0 Hz, 1H), 5.59 (t, J = 8.3 Hz, 1H), 5.20 (dt, J = 9.2, 6.3 Hz, 1H), 3.33 (s, 3H), 2.80 (ddd, J = 15.4, 8.6, 5.5 Hz, 1H), 2.72 (ddd, J = 14.6, 9.5, 6.4 Hz, 1H), 2.08 (tq, J = 11.0, 3.7 Hz, 1H), 2.04 – 1.97 (m, 1H), 1.97 – 1.88 (m, 1H), 1.88 – 1.82 (m, 2H), 1.80 – 1.74 (m, 1H), 1.74 – 1.62 (m, 2H), 1.43 (qd, J = 12.3, 3.3 Hz, 2H), 1.33 – 1.15 (m, 3H).z ^{13}C NMR (125 MHz, $CDCl_3$) δ 176.6, 157.8, 151.1, 149.9, 139.6, 129.0, 128.9, 127.1, 122.4, 120.9, 80.4, 56.0, 45.8, 36.5, 31.0, 30.0, 29.7, 25.80, 25.78, 25.72. HRMS (ESI, m/z) calculated for $C_{22}H_{29}N_2O_2^+$: 353.2229, found : 353.2227

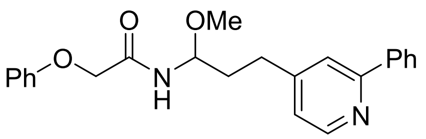

4q

N-(1-methoxy-3-(2-phenylpyridin-4-yl)propyl)-2-phenoxyacetamide (4q). Yield : 60% (22.4 mg). Yellow oil. ^{1}H NMR (500 MHz, $CDCl_3$) δ 8.59 (d, J = 5.0 Hz, 1H), 7.98 (d, J = 7.0 Hz, 2H), 7.55 (s, 1H), 7.48 (td, J = 7.4, 1.9 Hz, 2H), 7.45 – 7.40 (m, 1H), 7.40 – 7.31 (m, 2H), 7.05 (td, J = 7.8, 2.1 Hz, 2H), 6.97 – 6.91 (m, 2H), 6.79 (t, J = 9.5 Hz, 1H), 5.27 (ddd, J = 9.8, 6.8, 5.7 Hz, 1H), 4.55 (qd, J = 15.1, 1.9 Hz, 2H), 3.37 (s, 3H), 2.80 (d, J = 6.1 Hz, 1H), 2.74 (s, 1H), 2.09 (dqd, J = 19.1, 6.5, 3.5 Hz, 1H), 1.98 (dddd, J = 15.8, 11.8, 6.2, 2.3 Hz, 1H). ^{13}C NMR (125 MHz, $CDCl_3$) δ 169.1, 158.0, 157.3, 151.1, 150.0, 139.7, 130.2, 129.3, 129.1, 127.3, 122.7, 122.6, 121.1, 114.9, 80.7, 67.3, 56.4, 36.5, 31.0. HRMS (ESI, m/z) calculated for $C_{23}H_{25}N_2O_3^+$: 377.1865, found : 377.1864

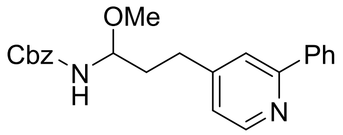

4r

benzyl (1-methoxy-3-(2-phenylpyridin-4-yl)propyl)carbamate (4r). Yield : 50% (18.6 mg). Colorless oil. ^{1}H NMR (500 MHz, $CDCl_3$) δ 8.57 (d, J = 5.0 Hz, 1H), 7.99 – 7.93 (m, 2H), 7.54 (s, 1H), 7.50 – 7.44 (m, 2H), 7.44 – 7.39 (m, 1H), 7.39 – 7.30 (m, 5H), 7.05 (d, J = 5.0 Hz, 1H), 5.12 (s, 2H), 5.01 (d, J = 10.1 Hz, 1H), 4.93 (dt, J = 10.5, 6.2 Hz, 1H), 3.37 (s, 3H), 2.84 – 2.69 (m, 2H), 2.02 (ddt, J = 13.2, 9.3, 6.5 Hz, 1H), 1.94 (dq, J = 12.7, 4.8 Hz, 1H). ^{13}C NMR (125 MHz, $CDCl_3$) δ 157.9, 156.1, 150.9, 149.9, 139.6, 136.2, 129.0, 128.9, 128.8, 128.5, 128.3, 127.1, 122.4, 120.9, 83.1, 67.2, 55.8, 36.6, 31.0. HRMS (ESI, m/z) calculated for $C_{23}H_{25}N_2O_3^+$: 377.1865, found : 377.1866

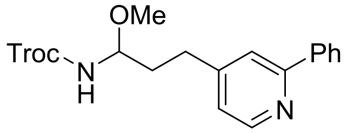

4s

2,2,2-trichloroethyl (1-methoxy-3-(2-phenylpyridin-4-yl)propyl)carbamate (4s). Yield : 51% (21.2 mg). Colorless oil. ^{1}H NMR

(500 MHz, $CDCl_3$) δ 8.59 (d, *J* = 5.0 Hz, 1H), 7.97 (d, *J* = 7.3 Hz, 2H), 7.55 (s, 1H), 7.47 (dd, *J* = 8.2, 6.7 Hz, 2H), 7.44 – 7.38 (m, 1H), 7.07 (dd, *J* = 5.0, 1.6 Hz, 1H), 5.24 (d, *J* = 10.1 Hz, 1H), 4.93 (dt, *J* = 10.1, 6.3 Hz, 1H), 4.81 (d, *J* = 12.1 Hz, 1H), 4.70 (d, *J* = 12.0 Hz, 1H), 3.39 (s, 3H), 2.87 – 2.72 (m, 2H), 2.07 (dq, *J* = 9.2, 6.8 Hz, 1H), 1.99 (ddd, *J* = 14.0, 8.4, 4.7 Hz, 1H). ^{13}C NMR (125 MHz, $CDCl_3$) δ 157.9, 154.4, 150.7, 149.9, 139.5, 129.1, 128.9, 127.1, 122.4, 120.9, 95.6, 83.4, 74.6, 55.9, 36.5, 31.0. HRMS (ESI, m/z) calculated for $C_{18}H_{20}N_2O_3Cl_3^+$: 417.0540, found : 417.0541

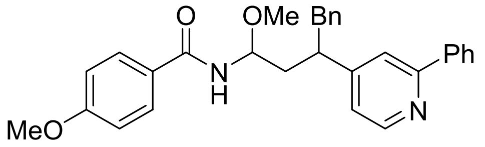

4t

4-methoxy-N-(1-methoxy-4-phenyl-3-(2-phenylpyridin-4-yl)butyl)benzamide (4t) Yield : 81% (37.6 mg). Colorless oil. Diastereomer mixture, 1.2:1. 1H NMR (400 MHz, CDCl3) δ 8.57 (dd, J = 12.2, 5.1 Hz, 1H), 8.07 – 7.89 (m, 1.2H), 7.85 (dd, J = 8.2, 1.6 Hz, 0.8H), 7.55 (dd, J = 8.8, 3.1 Hz, 2H), 7.52 – 7.34 (m, 4H), 7.25 – 7.13 (m, 3H), 7.05 (ddd, J = 8.1, 6.5, 2.8 Hz, 3H), 6.87 (d, J = 8.8 Hz, 1.1H), 6.85 – 6.76 (m, 0.8H), 6.03 (dd, J = 18.1, 9.5 Hz, 1H), 5.33 (dt, J = 9.6, 5.9 Hz, 0.6H), 5.18 (ddd, J = 9.5, 7.8, 5.7 Hz, 0.4H), 3.83 (s, 1.8H), 3.79 (s, 1.2H), 3.27 (d, J = 1.7 Hz, 3H), 3.18 (pd, J = 7.9, 6.9, 4.0 Hz, 1H), 3.07 – 2.85 (m, 2H), 2.32 – 1.71 (m, 2H). 13C NMR (100 MHz, CDCl3) δ 166.7, 166.6, 162.62, 162.56, 157.82, 157.76, 154.5, 153.9, 149.92, 149.87, 139.5, 139.1, 139.0, 129.2, 129.0, 128.9, 128.8, 128.74, 128.68, 128.5, 128.4, 127.12, 127.08, 126.5, 126.4, 125.8, 125.7, 121.7, 121.3, 120.5, 120.4, 113.9, 113.8, 80.3, 79.9, 77.4, 77.1, 76.8, 56.0, 55.9, 55.51, 55.45, 43.9, 43.4, 43.3, 42.3, 40.9, 40.6.HRMS (ESI, m/z) calculated for $C_{30}H_{31}N_2O_3^+$: 467.2335 , found : 467.2334.

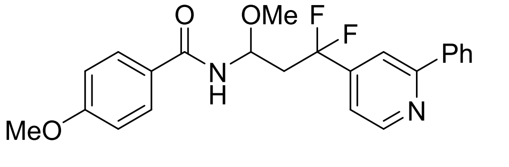

4u

N-(3,3-difluoro-1-methoxy-3-(2-phenylpyridin-4-yl)propyl)-4-methoxybenzamide (4u). Yield : 74% (30.2 mg). Colorless oil. ^{1}H NMR (500 MHz, $CDCl_3$) δ 8.74 (d, *J* = 5.1 Hz, 1H), 8.05 – 7.87 (m, 2H), 7.79 (s, 1H), 7.71 – 7.60 (m, 2H), 7.45 (tt, *J* = 14.7, 7.1 Hz, 3H), 7.32 – 7.28 (m, 1H), 6.90 – 6.85 (m, 2H), 6.31 (d, *J* = 9.6 Hz, 1H), 5.56 (dt, *J* = 9.9, 6.1 Hz, 1H), 3.83 (s, 3H), 3.30 (s, 3H), 2.68 (dqd, *J* = 55.6, 15.1, 6.1 Hz, 2H). ^{13}C NMR (100 MHz, $CDCl_3$) δ 166.5, 162.7, 158.3, 150.2, 145.6 (t, *J* = 27.3 Hz), 138.5, 129.4, 128.8, 127.0, 125.4, 122.6, 120.1, 117.8 (t, *J* = 5.9 Hz), 116.3 (t, *J* = 6.1 Hz), 55.6 (d, *J* = 39.3 Hz), 44.2 (t, *J* = 26.6 Hz). ^{19}F NMR (376 MHz, $CDCl_3$) δ -94.89. HRMS (ESI, m/z) calculated for $C_{23}H_{23}N_2O_3F_2^+$: 413.1677 , found : 413.1678.

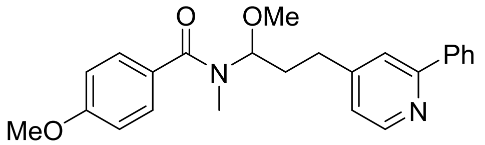

4v

4-methoxy-N-(1-methoxy-3-(2-phenylpyridin-4-yl)propyl)-N-methylbenzamide (4v) Yield : 38% (14.6 mg). White solid. With rotamer. ^{1}H NMR (500 MHz, C_2D_3N) δ 8.50 (d, J = 15.2 Hz, 1H), 8.02 (d, J = 8.3 Hz, 2H), 7.75 (s, 0.4H), 7.61 (s, 0.7H), 7.48 (t, J = 7.5 Hz, 2H), 7.43 (t, J = 7.2 Hz, 1.7H), 7.20 (s, 0.5H), 7.08 (d, J = 9.1 Hz, 2H), 6.95 (s, 0.8H), 6.67 (d, J = 8.3 Hz, 1.3H), 5.69 (s, 0.4H), 4.87 – 4.33 (m, 0.6H), 3.81 (s, 1.2H), 3.66 (s, 1.9H), 3.28 (s, 1.1H), 3.05 (s, 1.9H), 2.86 (s, 2H), 2.82 – 2.70 (m, 2.6H), 2.69 – 2.54 (m, 0.7H), 2.36 – 2.17 (m, 1.2H), 2.00 (s, 1H).^{13}C NMR (125 MHz, C_2D_3N) δ 172.5, 161.3, 157.8, 152.3, 152.0, 150.5, 140.3, 129.9, 129.7, 129.3, 127.7, 123.7, 121.6, 118.3, 114.5, 89.1, 84.7, 55.8, 55.3, 34.6, 33.4, 31.5, 26.2. HRMS (ESI, m/z) calculated for $C_{24}H_{27}N_2O_3^+$: 391.2022, found : 391.2023.

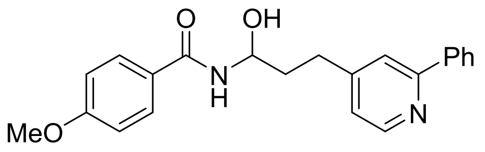

4w

N-(1-hydroxy-3-(2-phenylpyridin-4-yl)propyl)-4-methoxybenzamide (4w). Yield : 50% (17.9 mg). White solid. ^{1}H NMR (500 MHz, C_2D_6SO) δ 8.61 (d, J = 8.3 Hz, 1H), 8.54 (d, J = 5.0 Hz, 1H), 8.08 (dd, J = 7.7, 1.7 Hz, 2H), 7.96 – 7.84 (m, 2H), 7.81 (s, 1H), 7.48 (dd, J = 8.3, 6.7 Hz, 2H), 7.42 (t, J = 7.2 Hz, 1H), 7.22 (dd, J = 4.9, 1.6 Hz, 1H), 7.11 – 6.93 (m, 2H), 5.82 (dd, J = 4.8, 2.2 Hz, 1H), 5.48 – 5.31 (m, 1H), 3.81 (s, 3H), 2.73 (dh, J = 12.4, 6.9 Hz, 2H), 2.02 (dtt, J = 9.9, 6.5, 3.3 Hz, 2H). ^{13}C NMR (125 MHz, C_2D_6SO) δ 165.3, 161.6, 156.0, 151.8, 149.4, 138.8, 129.3, 128.9, 128.6, 126.6, 126.5, 122.7, 120.4, 113.4, 72.5, 55.3, 36.4, 30.7. HRMS (ESI, m/z) calculated for $C_{22}H_{23}N_2O_3^+$: 363.1709 , found : 363.1708. Due to its instability, hydrolyzed aldehyde containing pyridine detected majorly, calculated for calculated for C14H14NO$^+$: 212.1075 , found : 212.1075.

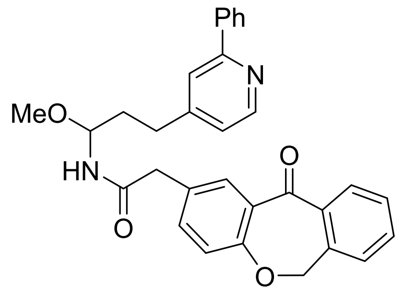

4x

N-(1-methoxy-3-(2-phenylpyridin-4-yl)propyl)-2-(11-oxo-6,11-dihydrodibenzo[b,e]oxepin-2-yl)acetamide (4x). Yield : 67% (32.6 mg). White solid. ^{1}H NMR (400 MHz, $CDCl_3$) δ 8.55 (d, J = 5.0 Hz, 1H), 8.11 (d, J = 2.4 Hz, 1H), 8.00 – 7.91 (m, 2H), 7.86 (dd, J = 7.8, 1.5 Hz, 1H), 7.55 (td, J = 7.5, 1.4 Hz, 1H), 7.53 – 7.49 (m, 1H), 7.49 – 7.33 (m, 6H), 7.07 – 6.92 (m, 2H), 5.80 (d, J = 9.6 Hz, 1H), 5.17 (s, 3H), 3.58 (d, J = 2.7 Hz, 2H), 3.30 (s, 3H), 2.70 (dddd, J = 30.1, 14.1, 9.6, 6.3 Hz, 2H), 1.95 (ddt, J = 13.2, 9.6, 6.6 Hz, 1H), 1.89 – 1.76 (m, 1H).^{13}C NMR (100 MHz, $CDCl_3$) δ 190.9, 171.2, 160.8, 157.8, 150.9, 149.8, 140.4, 139.5, 136.3, 135.6, 133.1, 132.5, 129.6, 129.5, 129.0, 128.8, 128.4, 128.0, 127.1, 125.4, 122.3, 121.8, 120.9, 80.9, 73.8, 56.1, 42.9, 36.3, 30.9. HRMS (ESI, m/z) calculated for $C_{31}H_{29}N_2O_4^+$: 493.2127 , found : 493.2129.

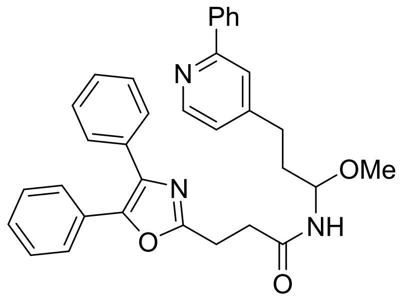

4y

3-(4,5-diphenyloxazol-2-yl)-N-(1-methoxy-3-(2-phenylpyridin-4-yl)propyl)propanamide (4y). Yield : 37% (18.7 mg). White solid. ^{1}H NMR (400 MHz, $CDCl_3$) δ 8.45 (d, J = 5.0 Hz, 1H), 8.07 – 7.67 (m, 2H), 7.53 – 7.43 (m, 4H), 7.42 – 7.30 (m, 5H), 7.25 – 7.19 (m, 4H), 6.88 (dd, J = 5.0, 1.7 Hz, 1H), 6.43 (d, J = 9.6 Hz, 1H), 5.12 (dt, J = 9.7, 6.3 Hz, 1H), 3.26 (s, 4H), 3.16 (t, J = 6.8 Hz, 2H), 2.74 (t, J = 6.9 Hz, 2H), 2.62 (dddd, J = 20.6, 18.9, 9.6, 4.4 Hz, 2H), 1.99 – 1.78 (m, 2H). ^{13}C NMR (100 MHz, $CDCl_3$) δ 171.9, 162.3, 157.7, 151.0, 149.8, 145.7, 139.5, 135.0, 132.3, 129.0, 128.82, 128.80, 128.73, 128.7, 128.3, 127.9, 127.1, 126.6, 122.3, 120.9, 80.7, 56.0, 36.3, 33.2, 30.9, 24.0. HRMS (ESI, m/z) calculated for $C_{33}H_{32}N_3O_3^+$: 518.2444 , found : 518.2444

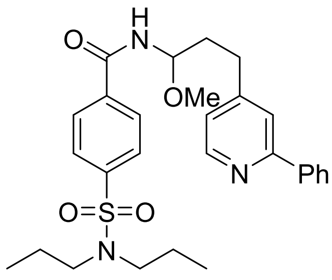

4z

4-(N,N-dipropylsulfamoyl)-N-(1-methoxy-3-(2-phenylpyridin-4-yl)propyl)benzamide (4z). Yield : 43% (13.9 mg). Ivory. ^{1}H NMR (500 MHz, $CDCl_3$) δ 8.58 (d, *J* = 5.0 Hz, 1H), 7.99 – 7.93 (m, 2H), 7.83 (q, *J* = 8.5 Hz, 4H), 7.58 (s, 1H), 7.51 – 7.45 (m, 2H), 7.43 – 7.39 (m, 1H), 7.10 (dd, *J* = 5.0, 1.6 Hz, 1H), 6.31 (d, *J* = 9.5 Hz, 1H), 5.44 – 5.36 (m, 1H), 3.44 (s, 3H), 3.10 – 3.04 (m, 4H), 2.90 (ddd, *J* = 15.0, 9.2, 6.1 Hz, 1H), 2.85 – 2.76 (m, 1H), 2.20 – 2.12 (m, 1H), 2.12 (s, 1H), 1.60 – 1.48 (m, 4H), 0.87 (t, *J* = 7.4 Hz, 6H). ^{13}C NMR (125 MHz, $CDCl_3$) δ 166.2, 157.9, 150.9, 150.0, 143.6, 139.5, 137.2, 129.1, 128.9, 127.8, 127.5, 127.1, 122.4, 120.9, 81.7, 56.5, 50.1, 36.4, 31.0, 22.1, 11.3. HRMS (ESI, m/z) calculated for $C_{28}H_{36}N_3O_4S^+$: 510.2427, found : 510.2428

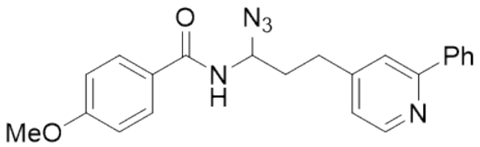

5a

N-(1-azido-3-(2-phenylpyridin-4-yl)propyl)-4-methoxybenzamide (5a). Yield : 77% (29.6 mg). White solid. ^{1}H NMR (500 MHz, $CDCl_3$) δ 8.53 (d, *J* = 5.0 Hz, 1H), 8.09 – 7.76 (m, 2H), 7.73 (d, *J* = 8.8 Hz, 2H), 7.49 (d, *J* = 1.6 Hz, 1H), 7.45 – 7.31 (m, 3H), 7.23 (d, *J* = 9.0 Hz, 1H), 6.99 (dd, *J* = 5.1, 1.6 Hz, 1H), 6.83 (d, *J* = 8.9 Hz, 2H), 5.72 (dt, *J* = 9.1, 6.9 Hz, 1H), 3.77 (s, 3H), 2.74 (ddt, *J* = 45.7, 14.7, 7.4 Hz, 2H), 1.99 (dtt, *J* = 13.5, 8.4, 4.1 Hz, 2H). ^{13}C NMR (125 MHz, $CDCl_3$) δ 167.4, 162.8, 157.8, 150.3, 149.8, 139.2, 129.3, 129.1, 128.8, 127.0, 125.1, 122.3, 120.8, 113.9, 66.8, 55.5, 35.0, 31.1. HRMS (ESI, m/z) calculated for $C_{22}H_{22}N_5O_2^+$:388.1773, found : 388.1772.

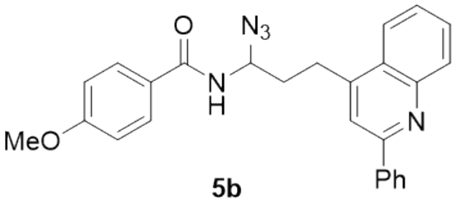

5b

N-(1-azido-3-(2-phenylquinolin-4-yl)propyl)-4-methoxybenzamide (5b). Yield : 73% (31.5 mg). White solid. ^{1}H NMR (400 MHz, CD_2Cl_2) δ 8.22 – 8.08 (m, 2H), 8.08 – 7.90 (m, 1H), 7.76 (s, 1H), 7.76 – 7.61 (m, 2H), 7.62 – 7.30 (m, 2H), 6.99 – 6.79 (m, 1H), 6.57 (d, *J* = 9.0 Hz, 1H), 5.83 (dt, *J* = 9.1, 6.9 Hz, 1H), 3.82 (s, 2H), 3.51 – 3.00 (m, 1H), 2.19 (dtd, *J* = 8.9, 6.7, 2.1 Hz, 1H). ^{13}C NMR (100 MHz, CD_2Cl_2) δ 167.1, 163.3, 157.1, 149.0, 147.3, 139.8, 130.9, 129.9, 129.7, 129.4, 129.1, 127.8, 126.8, 126.5, 125.6, 123.6, 119.1, 114.2, 67.5, 55.9, 35.3, 28.6. HRMS (ESI, m/z) calculated for $C_{26}H_{24}N_5O_2^+$: 438.1930 , found : 438.1931.

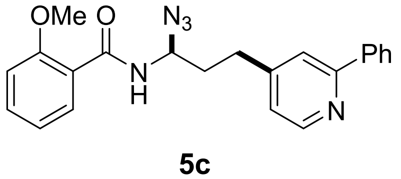

5c

N-(1-azido-3-(2-phenylpyridin-4-yl)propyl)-2-methoxybenzamide (5c). Yield : 44% (16.7 mg). Colorless oil. ^{1}H NMR (500 MHz, $CDCl_3$) δ 8.58 (d, *J* = 5.0 Hz, 1H), 8.31 (d, *J* = 8.9 Hz, 1H), 8.21 (dd, *J* = 7.8, 1.9 Hz, 1H), 8.03 – 7.74 (m, 2H), 7.55 (d, *J* = 1.6 Hz, 1H), 7.53 – 7.35 (m, 4H), 7.21 – 7.05 (m, 2H), 6.98 (d, *J* = 8.3 Hz, 1H), 5.83 (dt, *J* = 8.8, 6.8 Hz, 1H), 3.94 (s, 3H), 2.84 (dddd, *J* = 29.8, 14.1, 9.0, 6.5 Hz, 2H), 2.08 (ddt, *J* = 10.6, 9.2, 6.7 Hz, 2H). ^{13}C NMR (125 MHz, $CDCl_3$) δ 165.5, 157.8, 157.6, 150.1, 149.8, 139.2, 133.7, 132.6, 128.9, 128.7, 126.9, 122.2, 121.6, 120.7, 120.1, 111.4, 66.4, 56.1, 35.5, 31.1. HRMS (ESI, m/z) calculated for $C_{22}H_{22}N_5O_2^+$: 388.1773 , found : 388.1772

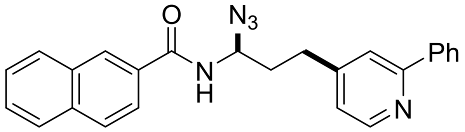

5d

N-(1-azido-3-(2-phenylpyridin-4-yl)propyl)-2-naphthamide (5d). Yield : 67% (27.6 mg). Colorless oil. ^{1}H NMR (500 MHz, $CDCl_3$) δ 8.65 – 8.55 (m, 1H), 8.21 (s, 1H), 7.96 (d, *J* = 7.6 Hz, 2H), 7.88 (d, *J* = 8.2 Hz, 3H), 7.77 (d, *J* = 8.5 Hz, 1H), 7.62 – 7.51 (m, 3H), 7.48 – 7.42 (m, 2H), 7.42 – 7.37 (m, 1H), 7.09 (d, *J* = 4.9 Hz, 1H), 6.77 (d, *J* = 9.3 Hz, 1H), 5.86 (q, *J* = 7.6 Hz, 1H), 2.93 (dt, *J* = 15.3, 7.7 Hz, 1H), 2.83 (dt, *J* = 14.9, 7.6 Hz, 1H), 2.14 (q, *J* = 7.4, 7.0 Hz, 2H). ^{13}C NMR (100 MHz, $CDCl_3$) δ 167.5, 158.0, 150.0, 149.9, 139.1, 135.1, 132.4, 130.0, 129.0, 128.9, 128.7, 128.1, 127.9, 127.8, 127.0, 126.9, 123.3, 122.2, 120.7, 66.8, 35.2, 31.0. HRMS (ESI, m/z) calculated for $C_{25}H_{22}N_5O^+$: 408.1824 , found : 408.1823

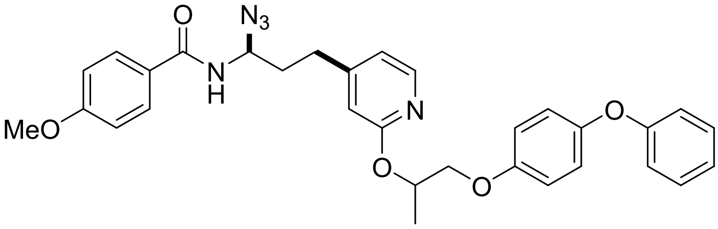

5e

N-(1-azido-3-(2-((1-(4-phenoxyphenoxy)propan-2-yl)oxy)pyridin-4-yl)propyl)-4-methoxybenzamide (5e). Yield : 42% (23.6 mg). Yellow oil. ^{1}H NMR (500 MHz, $CDCl_3$) δ 8.06 (d, *J* = 5.3 Hz, 1H), 7.68 (dd, *J* = 8.9, 2.6 Hz, 2H), 7.31 – 7.27 (m, 2H), 7.03 (t, *J* = 7.4 Hz, 1H), 6.98 – 6.89 (m, 8H), 6.72 (d, *J* = 5.0 Hz, 1H), 6.59 (s, 1H), 6.38 (d, *J* = 9.0 Hz, 1H), 5.74 (q, *J* = 7.2 Hz, 1H), 5.56 (q, *J* = 5.8 Hz, 1H), 4.16 (dt, *J* = 10.7, 5.5 Hz, 1H), 4.05 (dt, *J* = 9.9, 5.0 Hz, 1H), 3.85 (s, 3H), 2.73 (ddt, *J* = 46.7, 14.7, 7.7 Hz, 2H), 2.02 (qd, *J* = 7.8, 7.2, 3.0 Hz, 2H), 1.46 (t, *J* = 6.1 Hz, 3H). ^{13}C NMR (125 MHz, $CDCl_3$) δ 166.8, 163.7, 162.8, 158.5, 155.2, 152.2, 150.2, 147.0, 129.6, 129.0, 125.1, 122.4, 120.7, 117.6, 117.3, 115.8, 113.9, 111.0, 71.0, 69.4, 66.6, 55.5, 35.0, 30.6, 17.0. HRMS (ESI, m/z) calculated for $C_{31}H_{32}N_5O_5^+$: 554.2403 , found : 554.2404

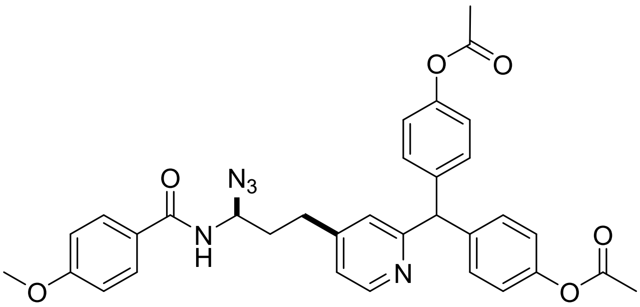

5f

((4-(3-azido-3-(4-methoxybenzamido)propyl)pyridin-2-yl)methylene)bis(4,1-phenylene) diacetate (5f). Yield : 60% (35.6 mg). Yellow oil. ^{1}H NMR (500 MHz, $CDCl_3$) δ 8.47 (d, *J* = 5.0 Hz, 1H), 7.70 (d, *J* = 8.8 Hz, 2H), 7.20 – 7.08 (m, 4H), 7.07 – 6.94 (m, 5H), 6.89 (dt, *J* = 7.0, 1.7 Hz, 3H), 6.68 (q, *J* = 7.5 Hz, 1H), 5.69 (dt, *J* = 9.1, 6.9 Hz, 1H), 5.60 (s, 1H), 3.84 (s, 3H), 2.73 (td, *J* = 9.2, 8.6, 4.4 Hz, 1H), 2.65 (dq, *J* = 14.7, 7.9, 7.4 Hz, 1H), 2.27 (s, 6H), 2.03 – 1.85 (m, 2H). ^{13}C NMR (125 MHz, $CDCl_3$) δ 169.5,

166.9, 162.9, 162.8, 150.0, 149.7, 149.3, 139.9, 139.8, 130.2, 129.1, 125.1, 123.7, 121.7, 121.5, 113.9, 66.6, 57.9, 55.4, 34.9, 30.8, 21.1. HRMS (ESI, m/z) calculated for $C_{33}H_{32}N_5O_6^+$: 594.2353 , found : 594.2354

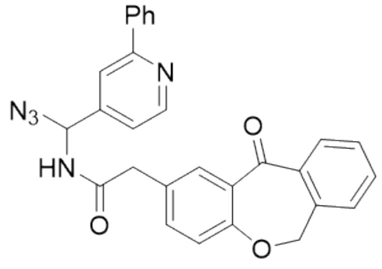

5g

N-(azido(2-phenylpyridin-4-yl)methyl)-2-(11-oxo-6,11-dihydrodibenzo[b,e]oxepin-2-yl)acetamide (5g). Yield : 60% (30.1 mg). White solid. ^{1}H NMR (400 MHz, CDCl$_3$) δ 8.56 (d, *J* = 5.0 Hz, 1H), 8.11 (d, *J* = 2.4 Hz, 1H), 7.99 – 7.86 (m, 2H), 7.84 (d, *J* = 7.7 Hz, 1H), 7.55 (t, *J* = 7.5 Hz, 1H), 7.50 – 7.32 (m, 7H), 7.05 (d, *J* = 8.5 Hz, 1H), 6.99 (d, *J* = 5.0 Hz, 1H), 6.23 (d, *J* = 9.6 Hz, 1H), 5.55 (dt, *J* = 8.9, 6.9 Hz, 1H), 5.16 (s, 2H), 3.61 (s, 2H), 2.70 (dqd, *J* = 20.8, 14.5, 7.4 Hz, 2H), 1.98 – 1.80 (m, 2H). ^{13}C NMR (100 MHz, CDCl$_3$ δ 191.0, 171.2, 160.9, 158.0, 150.0, 140.4, 139.3, 136.4, 135.6, 133.1, 132.5, 129.6, 129.5, 129.1, 128.9, 128.1, 128.0, 127.1, 125.4, 122.2, 121.9, 120.8, 73.7, 66.2, 42.5, 35.2, 31.1. HRMS (ESI, m/z) calculated for $C_{30}H_{26}N_5O_3^+$: 504.2036, found : 504.2036.

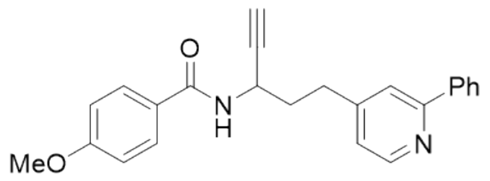

6a

4-methoxy-N-(5-(2-phenylpyridin-4-yl)pent-1-yn-3-yl)benzamide (6a). Yield : 89% (33.1 mg). Colorless oil. ^{1}H NMR (500 MHz, CDCl$_3$) δ 8.56 (d, *J* = 5.0 Hz, 1H), 8.06 – 7.80 (m, 2H), 7.70 (d, *J* = 8.9 Hz, 2H), 7.57 (s, 1H), 7.44 (td, *J* = 7.4, 6.3, 1.5 Hz, 2H), 7.41 – 7.33 (m, 1H), 7.08 (dd, *J* = 5.0, 1.6 Hz, 1H), 6.87 (d, *J* = 8.8 Hz, 2H), 6.46 (d, *J* = 8.1 Hz, 1H), 5.03 (tdd, *J* = 8.2, 6.1, 2.3 Hz, 1H), 3.81 (d, *J* = 1.2 Hz, 3H), 3.01 – 2.74 (m, 2H), 2.40 (d, *J* = 2.3 Hz, 1H), 2.26 – 2.05 (m, 2H). ^{13}C NMR (125 MHz, CDCl$_3$) δ 166.1, 162.5, 157.8, 150.8, 149.8, 139.5, 129.01, 128.96, 128.8, 127.1, 125.9, 122.4, 120.9, 113.9, 82.7, 72.5, 55.5, 41.7, 36.3, 31.7. HRMS (ESI, m/z) calculated for $C_{24}H_{23}N_2O_2^+$: 371.1761, found : 371.1760.

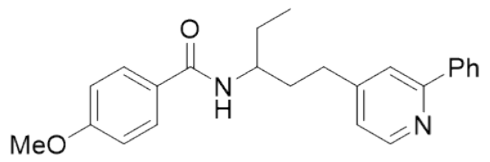

6b

4-methoxy-N-(1-(2-phenylpyridin-4-yl)pentan-3-yl)benzamide (6b). Yield : 81% (30.4 mg). Colorless oil. ^{1}H NMR (400 MHz, CDCl$_3$δ 8.53 (d, *J* = 5.1 Hz, 1H), 7.96 – 7.82 (m, 2H), 7.79 – 7.62 (m, 2H), 7.53 (s, 1H), 7.47 – 7.32 (m, 3H), 7.05 (dd, *J* = 5.0, 1.7 Hz, 1H), 6.98 – 6.77 (m, 2H), 5.95 (d, *J* = 9.1 Hz, 1H), 4.17 (qt, *J* = 8.9, 4.9 Hz, 1H), 3.81 (s, 3H), 2.74 (t, *J* = 8.0 Hz, 2H), 1.95 (ddd, *J* = 12.9, 9.3, 5.0 Hz, 1H), 1.81 (dq, *J* = 13.8, 8.1 Hz, 1H), 1.67 (dqd, *J* = 14.8, 7.4, 5.3 Hz, 1H), 1.59 – 1.42 (m, 1H), 0.96 (t, *J* = 7.4 Hz, 3H). ^{13}C NMR (100 MHz, CDCl$_3$) δ 167.0, 162.2, 157.6, 151.9, 149.7, 139.5, 128.9, 128.8, 128.7, 127.1, 127.0, 122.4, 120.9, 113.8, 55.5, 51.0, 35.9, 32.3, 28.4, 10.5. HRMS (ESI, m/z) calculated for $C_{24}H_{27}N_2O_2^+$: 375.2073 , found : 375.2074

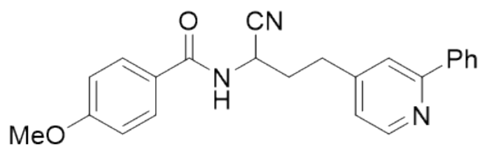

6c

N-(1-cyano-3-(2-phenylpyridin-4-yl)propyl)-4-methoxybenzamide (6c). Yield : 44% (16.2 mg). White solid. ^{1}H NMR (500 MHz, $CDCl_3$) δ 8.60 (dd, *J* = 5.0, 0.8 Hz, 1H), 8.02 – 7.87 (m, 2H), 7.76 – 7.62 (m, 2H), 7.63 – 7.54 (m, 1H), 7.52 – 7.43 (m, 2H), 7.43 – 7.33 (m, 1H), 7.08 (dd, *J* = 5.0, 1.6 Hz, 1H), 6.99 – 6.83 (m, 2H), 6.61 (d, *J* = 8.5 Hz, 1H), 5.17 (dt, *J* = 8.6, 7.3 Hz, 1H), 3.83 (s, 3H), 3.47 – 2.80 (m, 2H), 2.29 (q, *J* = 7.4 Hz, 2H). ^{13}C NMR (125 MHz, $CDCl_3$) δ 166.2, 163.0, 158.1, 150.1, 149.1, 139.1, 129.2, 129.1, 128.8, 127.0, 124.5, 122.1, 120.7, 118.4, 114.0, 55.5, 40.6, 33.9, 31.3.. HRMS (ESI, m/z) calculated for $C_{23}H_{22}N_3O_2^+$: 372.1712, found : 372.1713

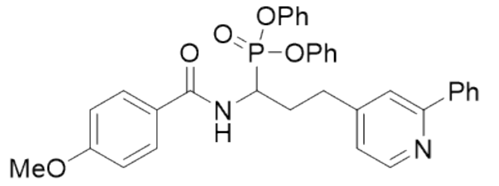

6d

diphenyl (1-(4-methoxybenzamido)-3-(2-phenylpyridin-4-yl)propyl)phosphonate (6d). Yield : 74% (42.5 mg). Colorless oil. ^{1}H NMR (400 MHz, $CDCl_3$) δ 8.50 (dd, *J* = 5.0, 2.1 Hz, 1H), 8.04 – 7.83 (m, 2H), 7.80 – 7.70 (m, 2H), 7.53 – 7.35 (m, 5H), 7.30 (t, *J* = 7.8 Hz, 2H), 7.17 (d, *J* = 8.0 Hz, 3H), 7.13 – 7.05 (m, 3H), 7.05 – 6.99 (m, 4H), 6.94 (dt, *J* = 5.6, 2.8 Hz, 1H), 6.84 – 6.69 (m, 2H), 3.78 (s, 2H), 2.81 (tdd, *J* = 11.0, 7.6, 4.5 Hz, 2H), 2.42 – 2.17 (m, 2H). ^{13}C NMR (100 MHz, $CDCl_3$) δ 167.1, 162.6, 157.7, 150.5, 150.45, 150.39, 150.1, 150.0, 149.8, 139.4, 130.1, 129.7, 129.33, 129.31, 129.0, 128.8, 127.1, 125.7, 125.4, 122.3, 120.9, 120.63, 120.58, 120.52, 120.48, 113.7, 55.5, 46.9, 45.4, 31.9, 31.7, 30.7. ^{31}P NMR (162 MHz, $CDCl_3$) δ 17.0. HRMS (ESI, m/z) calculated for $C_{34}H_{32}N_2O_5P^+$: 579.2049 , found : 579.2063, $C_{34}H_{31}N_2O_5NaP^+$: 601.1868, found : 601.1880.

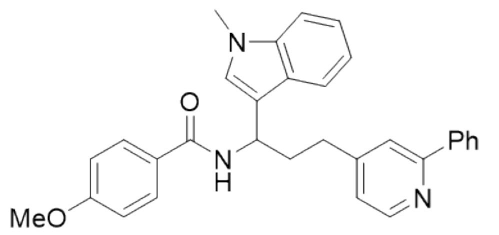

6e

4-methoxy-N-(1-(1-methyl-1H-indol-3-yl)-3-(2-phenylpyridin-4-yl)propyl)benzamide (6e). Yield : 63% (29.9 mg). White solid. ^{1}H NMR (500 MHz, CD_2Cl_2) δ 8.53 (d, *J* = 5.0 Hz, 1H), 8.06 – 7.89 (m, 2H), 7.75 – 7.65 (m, 3H), 7.62 (d, *J* = 1.5 Hz, 1H), 7.46 (dd, *J* = 8.3, 6.6 Hz, 2H), 7.42 – 7.37 (m, 1H), 7.33 (d, *J* = 8.1 Hz, 1H), 7.23 (ddd, *J* = 8.2, 7.0, 1.2 Hz, 1H), 7.12 (dd, *J* = 5.1, 1.6 Hz, 1H), 7.11 – 7.04 (m, 2H), 6.92 – 6.85 (m, 2H), 6.34 (d, *J* = 8.2 Hz, 1H), 5.57 (q, *J* = 7.5 Hz, 1H), 3.81 (s, 3H), 3.76 (s, 3H), 3.07 – 2.72 (m, 2H), 2.48 (dddd, *J* = 18.5, 13.4, 9.1, 6.5 Hz, 2H). ^{13}C NMR (125 MHz, CD_2Cl_2) δ 166.3, 162.5, 157.5, 152.1, 149.9, 137.8, 129.1, 128.99, 128.96, 127.4, 127.2, 126.95, 126.90, 122.8, 122.3, 121.0, 119.7, 119.6, 115.5, 114.0, 109.9, 55.8, 54.3, 54.1, 53.8, 53.6, 53.4, 46.9, 36.1, 33.1, 32.9.HRMS (ESI, m/z) calculated for $C_{31}H_{30}N_3O_2^+$: 476.2338, found : 476.2339.

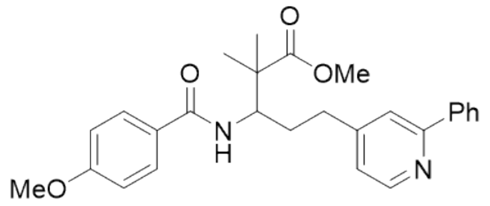

6f

methyl 3-(4-methoxybenzamido)-2,2-dimethyl-5-(2-phenylpyridin-4-yl)pentanoate (6f). Yield : 60% (26.7 mg). Colorless oil. δ 8.54 (d, *J* = 5.0 Hz, 1H), 8.07 – 7.82 (m, 2H), 7.77 (d, *J* = 8.8 Hz, 2H), 7.53 (s, 1H), 7.45 (t, *J* = 7.3 Hz, 2H), 7.41 – 7.34 (m, 1H), 7.07 (dd, *J* = 5.2, 1.6 Hz, 1H), 7.03 (d, *J* = 10.1 Hz, 1H), 6.93 (d, *J* = 8.8 Hz, 2H), 4.27 (td, *J* = 10.8, 2.5 Hz, 1H), 3.84 (s, 3H), 3.71 (s, 3H), 2.94 – 2.55 (m, 2H), 2.05 (dddd, *J* = 13.5, 9.2, 6.8, 2.6 Hz, 1H), 1.72 (dddd, *J* = 13.5, 11.3, 9.0, 6.4 Hz, 1H), 1.28 (d, *J* = 2.0 Hz, 6H). ^{13}C NMR (100 MHz, $CDCl_3$) δ 177.9, 167.0, 162.4, 157.6, 151.7, 149.7, 139.6, 128.9, 128.8, 128.8, 127.1, 126.6, 122.4, 121.0, 114.0, 56.1, 55.6, 52.2, 46.4, 32.8, 32.5, 24.7, 23.3. HRMS (ESI, m/z) calculated for $C_{27}H_{31}N_2O_4^+$: 447.2284, found : 447.2284.

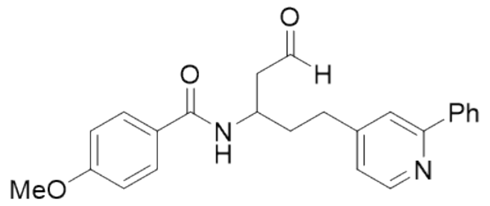

6g

4-methoxy-N-(1-oxo-5-(2-phenylpyridin-4-yl)pentan-3-yl)benzamide (6g). Yield : 50% (19.5 mg). White solid. ^{1}H NMR (400 MHz, $CDCl_3$) δ 9.79 (s, 1H), 8.55 (d, *J* = 5.0 Hz, 1H), 7.94 (dd, *J* = 7.4, 2.0 Hz, 2H), 7.67 (d, *J* = 8.5 Hz, 2H), 7.54 (s, 1H), 7.48 – 7.34 (m, 3H), 7.06 (dd, *J* = 5.1, 1.9 Hz, 1H), 6.96 – 6.75 (m, 2H), 6.57 (q, *J* = 7.8, 6.9 Hz, 1H), 4.59 (tq, *J* = 9.8, 5.1 Hz, 1H), 3.82 (s, 3H), 2.96 – 2.57 (m, 4H), 2.25 – 2.07 (m, 1H), 2.05 – 1.87 (m, 1H). ^{13}C NMR (100 MHz, $CDCl_3$) δ 201.4, 166.7, 162.5, 157.8, 151.1, 149.8, 139.4, 129.0, 128.8, 127.1, 126.3, 122.4, 120.9, 113.9, 55.5, 48.4, 45.5, 35.2, 32.5. HRMS (ESI, m/z) calculated for $C_{24}H_{25}N_2O_3^+$: 389.1865, found : 389.1869

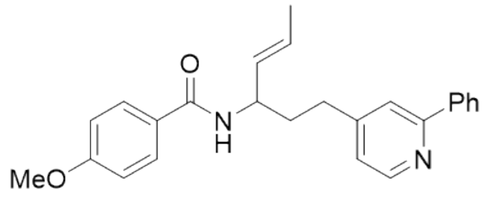

6h

(E)-4-methoxy-N-(1-(2-phenylpyridin-4-yl)hex-4-en-3-yl)benzamide (6h). Yield : 41% (22.4 mg). Colorless oil. ^{1}H NMR (500 MHz, $CDCl_3$) δ 8.58 (d, *J* = 5.0 Hz, 1H), 8.21 – 7.87 (m, 2H), 7.72 (d, *J* = 8.7 Hz, 2H), 7.58 (s, 1H), 7.48 (ddd, *J* = 7.8, 6.5, 1.5 Hz, 2H), 7.42 (td, *J* = 7.2, 1.4 Hz, 1H), 7.11 (dd, *J* = 5.1, 1.7 Hz, 1H), 6.95 – 6.78 (m, 2H), 5.97 (d, *J* = 8.4 Hz, 1H), 5.84 – 5.67 (m, 1H), 5.52 (ddt, *J* = 15.3, 6.6, 1.7 Hz, 1H), 4.72 (p, *J* = 7.2 Hz, 1H), 3.86 (d, *J* = 1.4 Hz, 3H), 2.92 – 2.68 (m, 2H), 2.17 – 1.87 (m, 2H), 1.75 (dd, *J* = 6.5, 1.4 Hz, 3H). ^{13}C NMR (125 MHz, $CDCl_3$) δ 166.4, 162.4, 157.7, 151.6, 149.8, 139.6, 130.8, 129.0, 128.8, 128.8, 127.8, 127.1, 126.9, 122.4, 120.9, 113.9, 55.6, 51.4, 36.1, 32.2, 18.0. HRMS (ESI, m/z) calculated for $C_{25}H_{27}N_2O_2^+$: 387.2073, found : 387.2073.

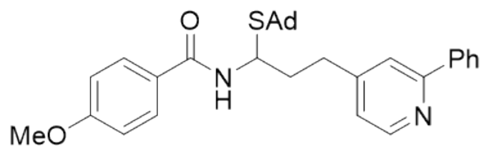

6i

N-(1-(((3s,5s,7s)-adamantan-1-yl)thio)-3-(2-phenylpyridin-4-yl)propyl)-4-methoxybenzamide (6i). Yield : 89% (45.5 mg). White solid. ^{1}H NMR (500 MHz, $CDCl_3$) ^{1}H NMR (400 MHz, $CDCl_3$) δ 8.56 (dd, *J* = 5.1, 0.7 Hz, 1H), 8.05 – 7.83 (m, 2H), 7.69 (d, *J* = 8.8 Hz, 2H), 7.58 (d, *J* = 1.7 Hz, 1H), 7.47 – 7.42 (m, 2H), 7.41 – 7.32 (m, 1H), 7.10 (dd, *J* = 5.0, 1.7 Hz, 1H), 7.00 – 6.82 (m, 2H), 6.31 (d, *J* = 8.4 Hz, 1H), 5.43 (ddd, *J* = 8.5, 7.4, 6.2 Hz, 1H), 3.83 (s, 3H), 2.92 (ddd, *J* = 14.0, 10.1, 5.6 Hz, 1H), 2.81 (ddd, *J* = 14.0, 10.1, 6.0 Hz, 1H), 2.20 (ddt, *J* = 13.8, 10.2, 6.1 Hz, 1H), 2.08 (dtd, *J* = 10.1, 7.6, 3.7 Hz, 1H), 1.99 (p, *J* = 3.1 Hz, 3H), 1.94 (dt, *J* = 11.9, 2.8 Hz, 3H), 1.87 (dt, *J* = 11.9, 2.9 Hz, 3H), 1.65 (d, *J* = 3.0 Hz, 6H) ^{13}C NMR (125 MHz, $CDCl_3$) δ 165.2, 162.5, 157.6, 151.1, 149.7, 139.5, 128.9, 128.8, 127.1, 126.3, 122.4, 121.0, 114.0, 55.5, 50.9, 46.9, 44.0, 38.0, 36.1, 32.3, 29.8. HRMS (ESI, m/z) calculated for $C_{32}H_{37}N_2O_2S^+$: 513.2576, found : 513.2576.

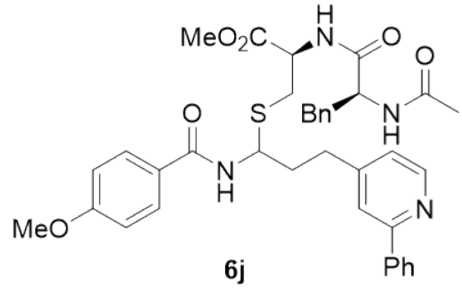

6j

methyl N-(acetyl-L-phenylalanyl)-S-(1-(4-methoxybenzamido)-3-(2-phenylpyridin-4-yl)propyl)-L-cysteinate (6j). Yield : 81% (54.1 mg). White solid. 2 dr mixture with 1 rotamer ^{1}H NMR (500 MHz, CD_2Cl_2 δ 8.56 (t, *J* = 5.1 Hz, 1H), 8.01 (ddq, *J* = 6.5, 3.1, 1.7 Hz, 2H), 7.93 – 7.85 (m, 0.5H), 7.85 – 7.75 (m, 2H), 7.70 – 7.59 (m, 1.2H), 7.55 – 7.39 (m, 3H), 7.38 – 7.23 (m, 4H), 7.21 (dd, *J* = 6.9, 1.7 Hz, 0.5H), 7.17 (dd, *J* = 6.9, 1.7 Hz, 0.7H), 7.13 (qd, *J* = 5.2, 1.6 Hz, 1H), 7.10 – 7.04 (m, 0.2H), 6.97 (t, *J* = 8.5 Hz, 2.4H), 6.89 (dd, *J* = 7.3, 3.0 Hz, 0.3H), 6.83 (dd, *J* = 9.3, 5.3 Hz, 0.5H), 6.55 (dd, *J* = 8.1, 4.1 Hz, 0.3H), 6.42 (dd, *J* = 8.4, 3.5 Hz, 0.5H), 6.19 (d, *J* = 8.2 Hz, 0.2H), 5.49 (dddd, *J* = 23.7, 11.1, 8.7, 6.4 Hz, 1H), 4.91 – 4.80 (m, 1H), 4.75 (ddt, *J* = 9.9, 6.6, 2.9 Hz, 0.5H), 4.70 (dd, *J* = 7.7, 5.9 Hz, 0.2H), 4.61 (q, *J* = 7.4 Hz, 0.3H), 3.91 – 3.84 (m, 3H), 3.70 (t, *J* = 2.1 Hz, 3H), 3.28 (dd, *J* = 14.0, 5.7 Hz, 0.5H), 3.21 – 3.10 (m, 0.9H), 3.09 – 2.96 (m, 2.1H), 2.90 (dddd, *J* = 27.9, 13.7, 6.1, 3.8 Hz, 2.8H), 2.21 (dddd, *J* = 15.8, 13.5, 9.3, 6.2 Hz, 2H), 2.08 – 1.90 (m, 3H).^{13}C NMR (125 MHz, $CDCl_3$) δ 171.7, 171.25, 171.18, 170.9, 170.8, 170.7, 170.6, 170.3, 167.3, 167.03, 167.00, 162.92, 162.89, 162.8, 157.85, 157.82, 150.8, 150.72, 150.67, 149.89, 149.85, 139.4, 139.3, 136.7, 136.5, 129.5, 129.42, 129.35, 129.3, 129.2, 129.11, 129.09, 129.07, 128.83, 128.80, 128.7, 127.2, 127.11, 127.09, 127.0, 125.6, 125.5, 125.3, 122.37, 122.35, 120.9, 114.1, 114.0, 113.9, 55.8, 55.605, 55.596, 55.56, 55.3, 54.9, 54.6, 54.3, 53.7, 53.0, 52.9, 52.84, 52.81, 38.2, 37.8, 37.6, 37.0, 36.7, 36.6, 32.7, 32.5, 32.4, 32.2, 32.1, 23.4, 23.27, 23.25.HRMS (ESI, m/z) calculated for $C_{37}H_{41}N_4O_6S^+$: 669.2747, found : 669.2749, calculated for $C_{37}H_{41}N_4O_6NaS^+$: 691.2566, found : 691.2565.

EXSY analysis (500 MHz, C_2H_6SO) established the presence of rotamers in the sample. Variable-temperature NMR experiments were conducted up to 80 °C; however, coalescence of the rotamer signals was not observed in the 1H NMR spectrum, and further heating was avoided due to concern about sample decomposition. Because a reliable dr value could not be determined, the relative integrals of the three peaks in sample corresponding to the diastereomers/rotamers are reported instead (0.3:0.5:0.2).

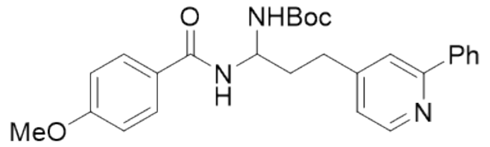

6k

tert-butyl (1-(4-methoxybenzamido)-3-(2-phenylpyridin-4-yl)propyl)carbamate (6k). Yield : 78% (35.8 mg). White solid. ^{1}H

NMR (500 MHz, $CDCl_3$) δ 8.53 (d, *J* = 5.1 Hz, 1H), 8.12 – 7.86 (m, 2H), 7.72 (d, *J* = 8.7 Hz, 2H), 7.52 (s, 1H), 7.43 (dd, *J* = 8.3, 6.6 Hz, 2H), 7.38 (t, *J* = 7.2 Hz, 1H), 7.08 – 7.00 (m, 1H), 6.86 (d, *J* = 8.5 Hz, 2H), 5.98 (s, 1H), 5.20 (s, 1H), 3.80 (s, 3H), 2.73 (hept, *J* = 7.6, 7.2 Hz, 2H), 2.39 (s, 2H), 2.07 (s, 1H), 1.41 (s, 9H). ^{13}C NMR (125 Hz, $CDCl_3$) δ 167.0, 162.6, 157.7, 155.6, 150.9, 149.8, 139.5, 129.0, 129.0, 128.8, 127.1, 126.2, 122.4, 120.9, 113.9, 80.3, 59.2, 55.5, 34.6, 32.1, 28.4. HRMS (ESI, m/z) calculated for $C_{27}H_{32}N_3O_4^+$: 462.2393, found : 462.2395.

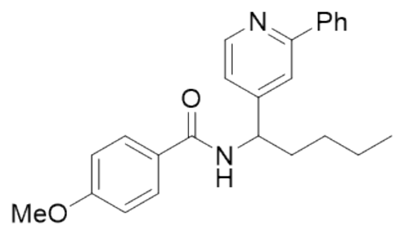

8

4-methoxy-N-(1-(2-phenylpyridin-4-yl)pentyl)benzamide (8). Yield : 12% (17.4 mg). White solid. ^1H NMR (500 MHz, $CDCl_3$) δ 8.63 (d, *J* = 5.1 Hz, 1H), 8.11 – 7.87 (m, 2H), 7.77 (s, 1H), 7.67 (d, *J* = 1.6 Hz, 1H), 7.46 (t, *J* = 7.4 Hz, 2H), 7.44 – 7.34 (m, 1H), 7.19 (dd, *J* = 5.1, 1.6 Hz, 1H), 7.09 – 6.77 (m, 2H), 6.39 (d, *J* = 7.8 Hz, 1H), 5.17 (q, *J* = 7.5 Hz, 1H), 3.84 (s, 3H), 1.90 (qt, *J* = 11.6, 5.5 Hz, 2H), 1.39 (dddt, *J* = 19.5, 14.0, 10.9, 5.0 Hz, 5H), 0.90 (t, *J* = 6.8 Hz, 3H). ^{13}C NMR (125 MHz, $CDCl_3$)) δ 166.6, 162.5, 158.1, 152.6, 150.1, 139.5, 129.1, 128.9, 128.8, 127.2, 126.5, 120.1, 118.9, 114.0, 55.6, 53.4, 35.8, 28.4, 22.6, 14.1. HRMS (ESI, m/z) calculated for $C_{24}H_{27}N_2O_2^+$: 375.2073, found : 375.2072.

IX. Spectral Copies of ^{1}H-, ^{13}C-, ^{19}F-, ^{31}P- NMR Data

N-(trans-2-benzylcyclopropyl)-4-methoxybenzamide (1t).

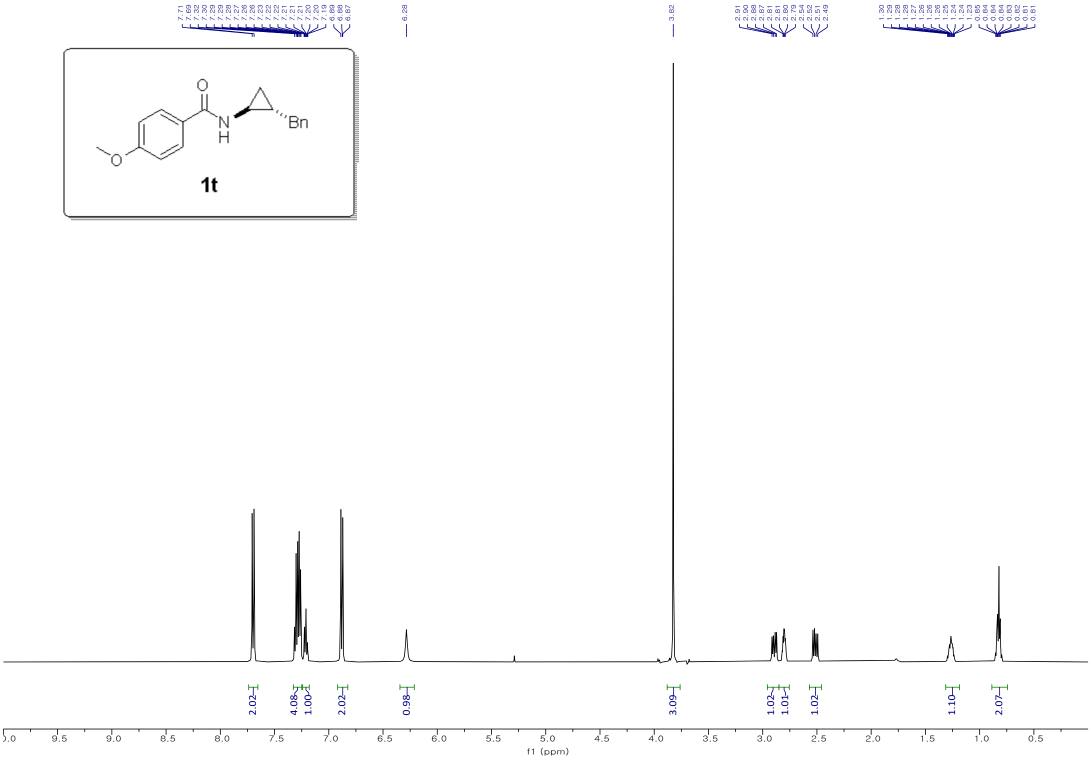

500 MHz, ^{1}H NMR in $CDCl_3$

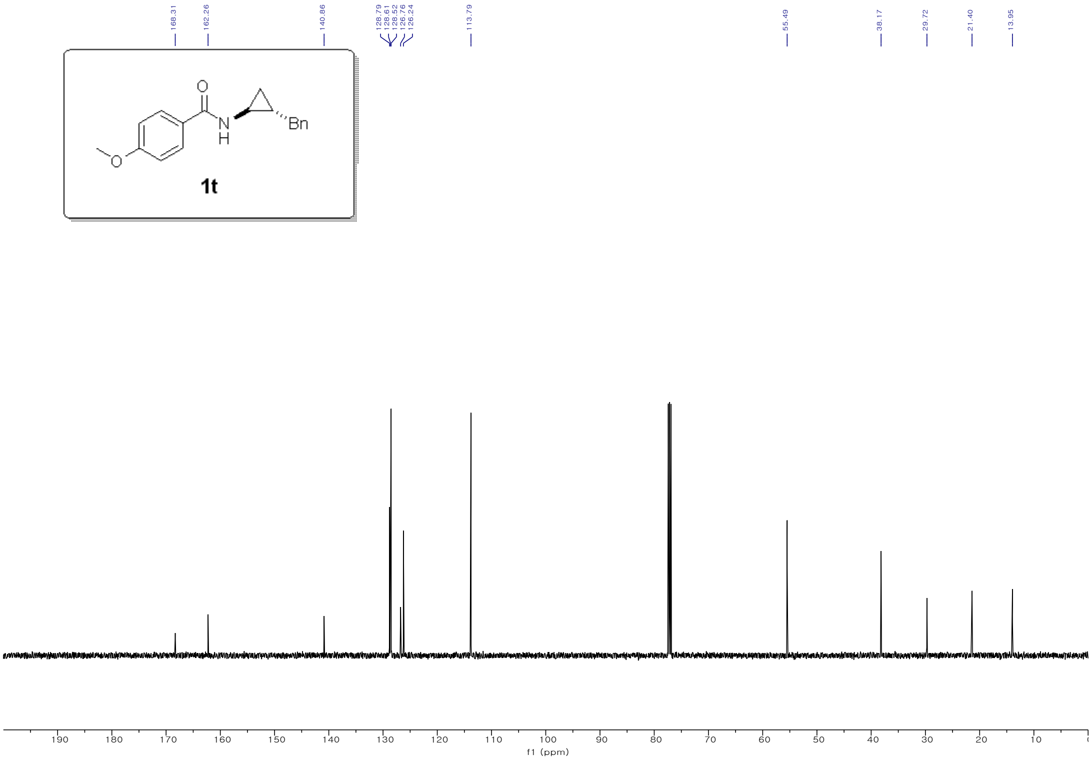

125 MHz, ^{13}C NMR in $CDCl_3$

N-(2,2-difluorocyclopropyl)-4-methoxybenzamide (1u).

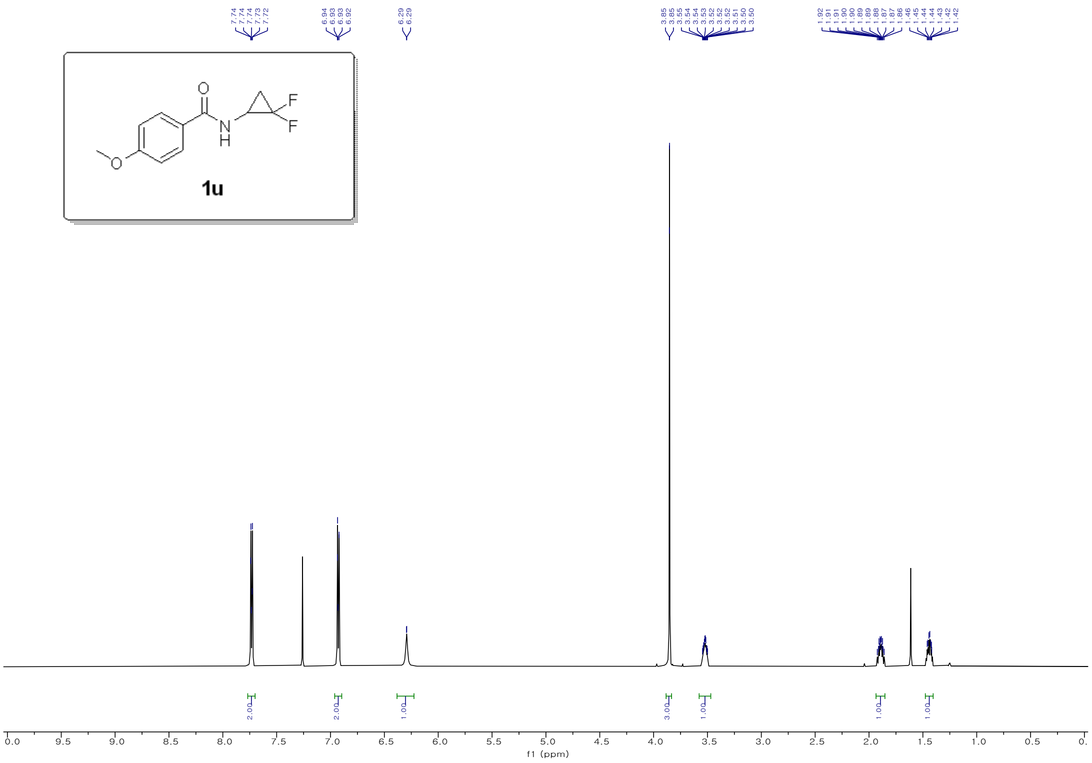

600 MHz, ^{1}H NMR in $CDCl_3$

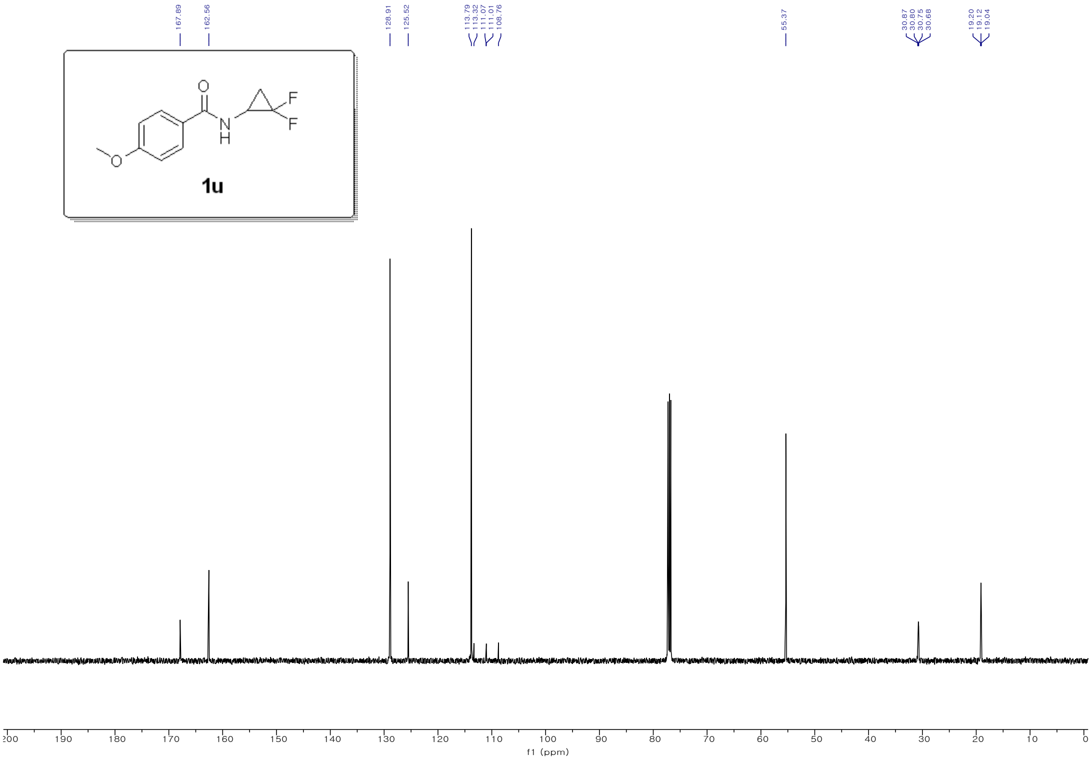

125 MHz, ^{13}C NMR in $CDCl_3$

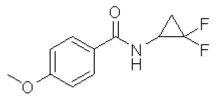

1u

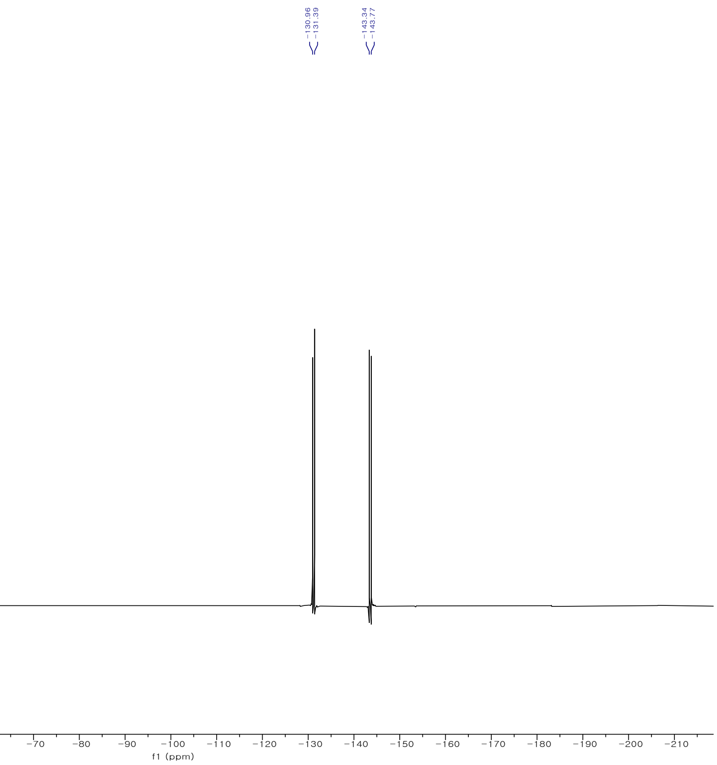

376 MHz, ^{19}F NMR in $CDCl_3$

N-cyclopropyl-2-(11-oxo-6,11-dihydrodibenzo[b,e]oxepin-2-yl)acetamide (1x)

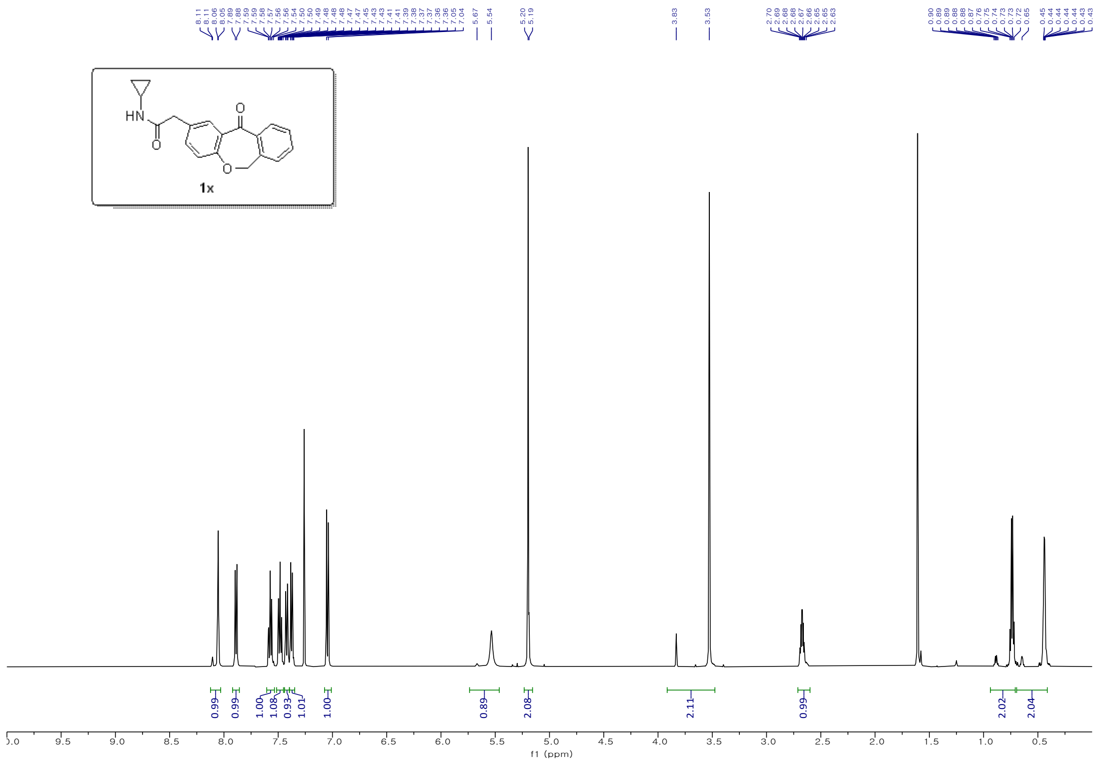

500 MHz, ^{1}H NMR in $CDCl_3$

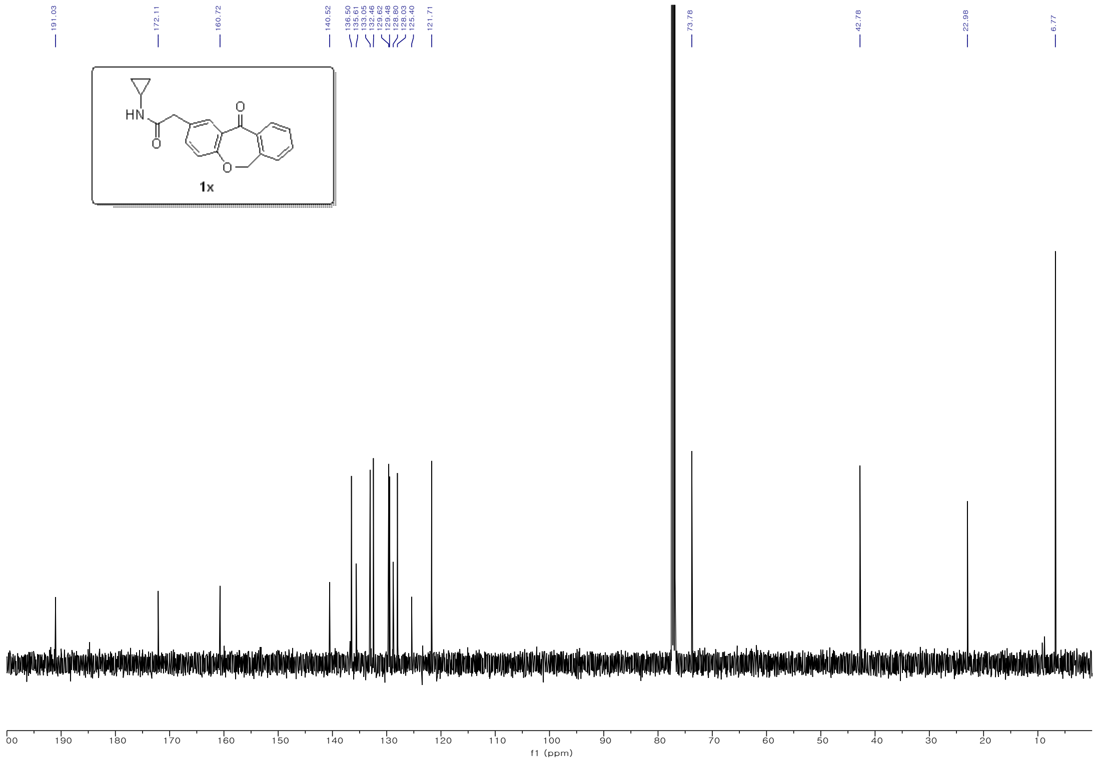

125 MHz, ^{13}C NMR in $CDCl_3$

N-cyclopropyl-3-(4,5-diphenyloxazol-2-yl)propenamide (1y)

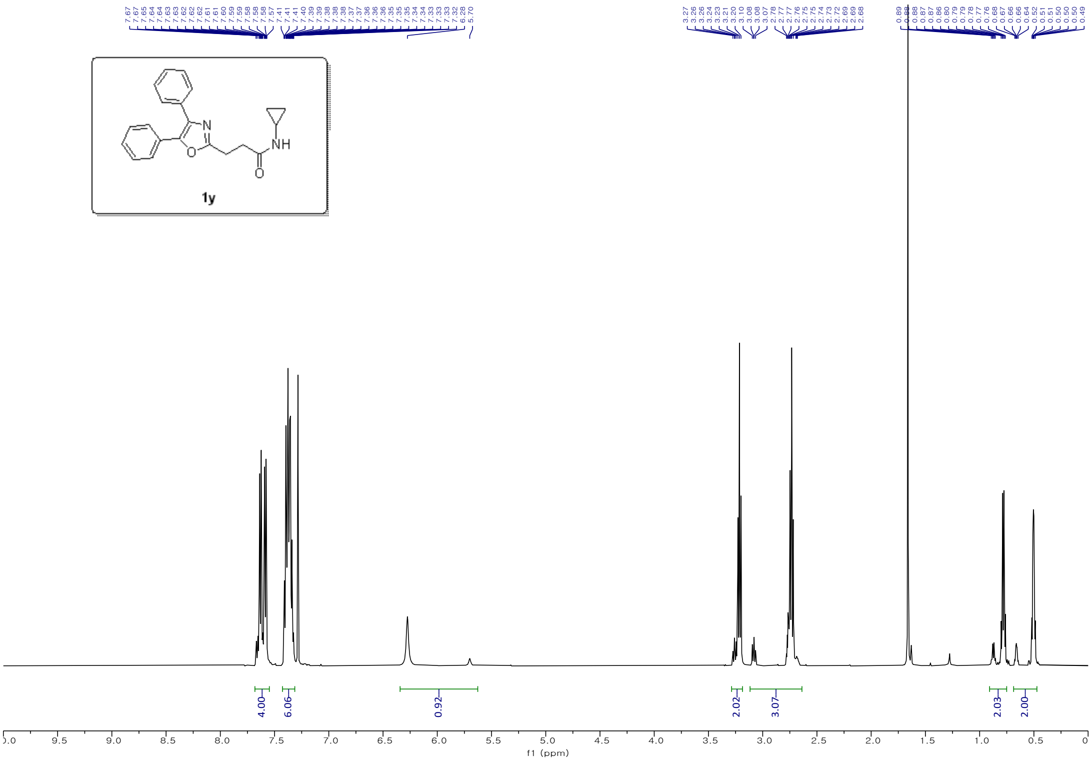

500 MHz, ^{1}H NMR in CDCl$_3$

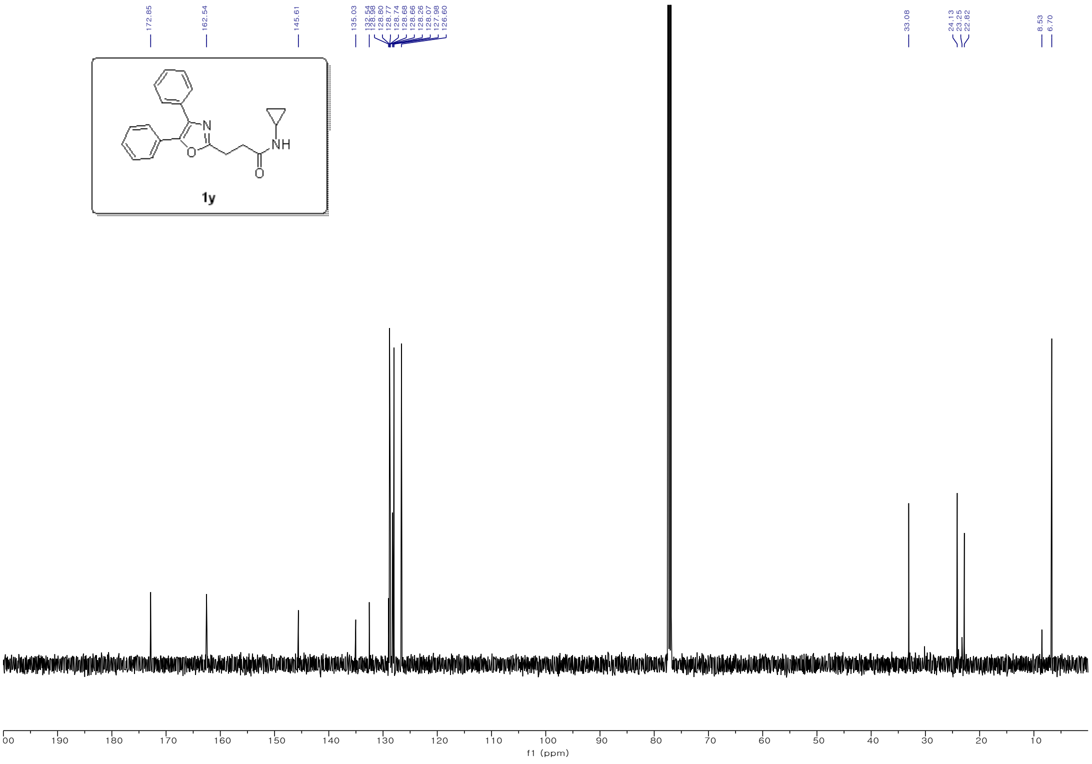

125 MHz, ^{13}C NMR in CDCl$_3$

N-cyclopropyl-4-(N,N-dipropylsulfamoyl)benzamide (1z).

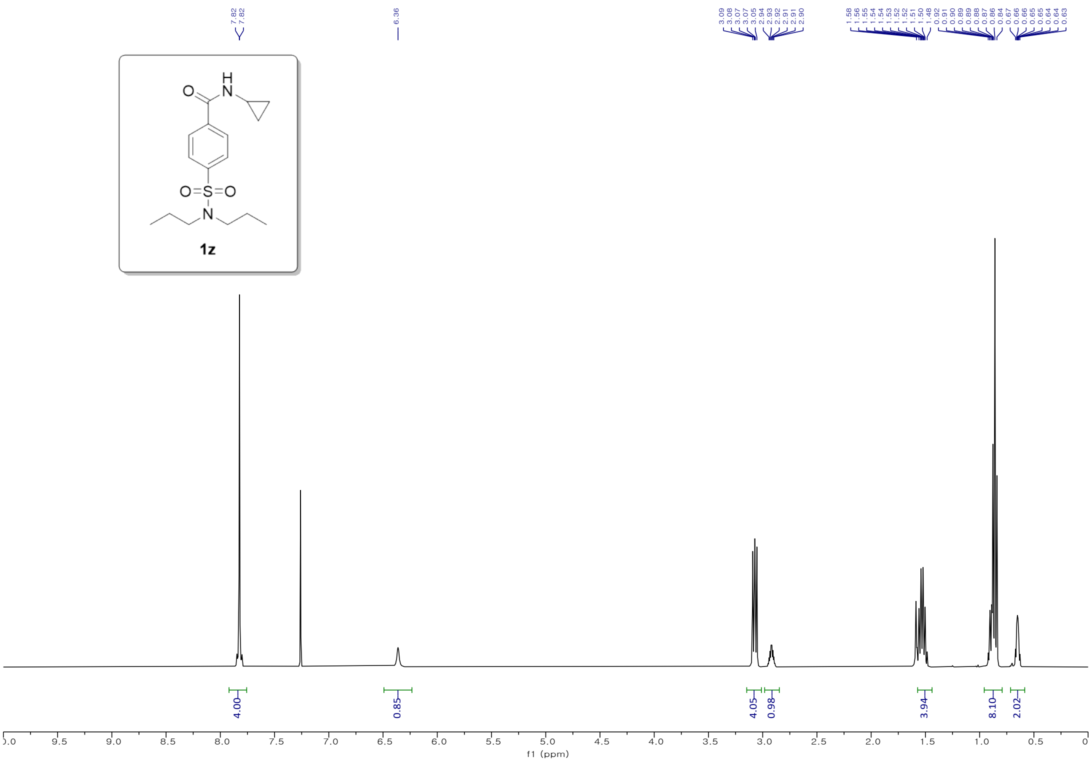

400 MHz, ^{1}H NMR in $CDCl_3$

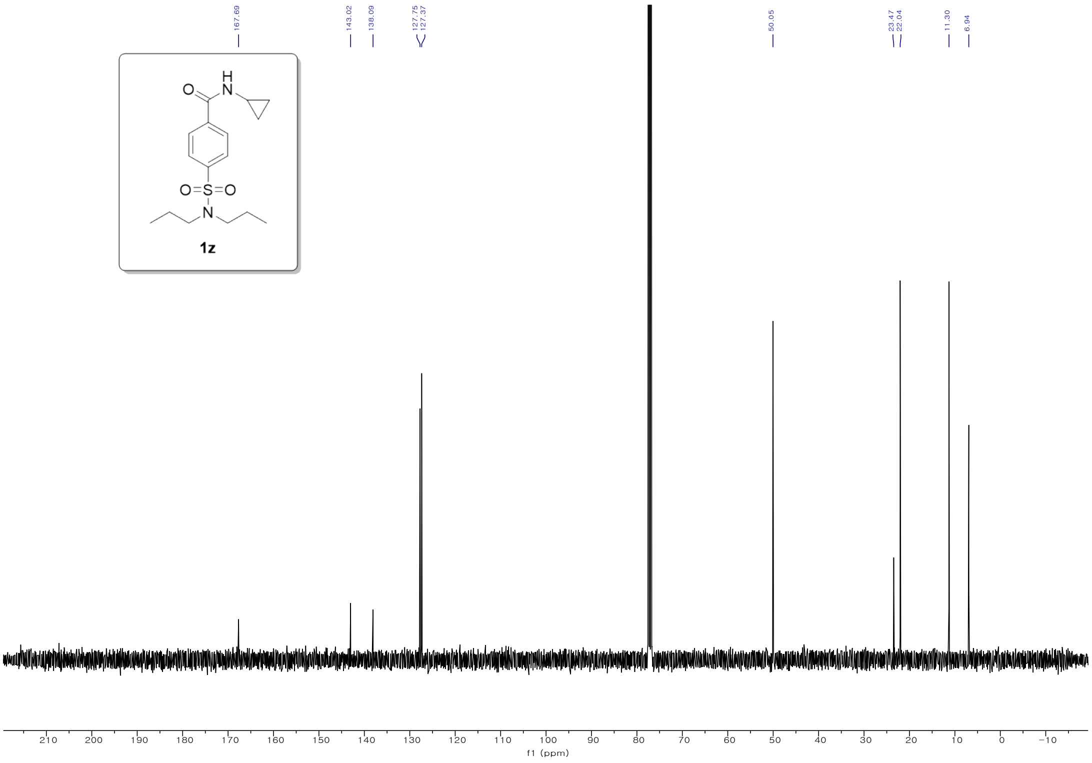

100 MHz, ^{13}C NMR in $CDCl_3$

2-(((2-(4-chlorophenoxy)-2-methylpropanoyl)oxy)methyl)-1-((N,4-dimethylphenyl)sulfonamido)pyridin-1-ium tetrafluoroborate (2s).

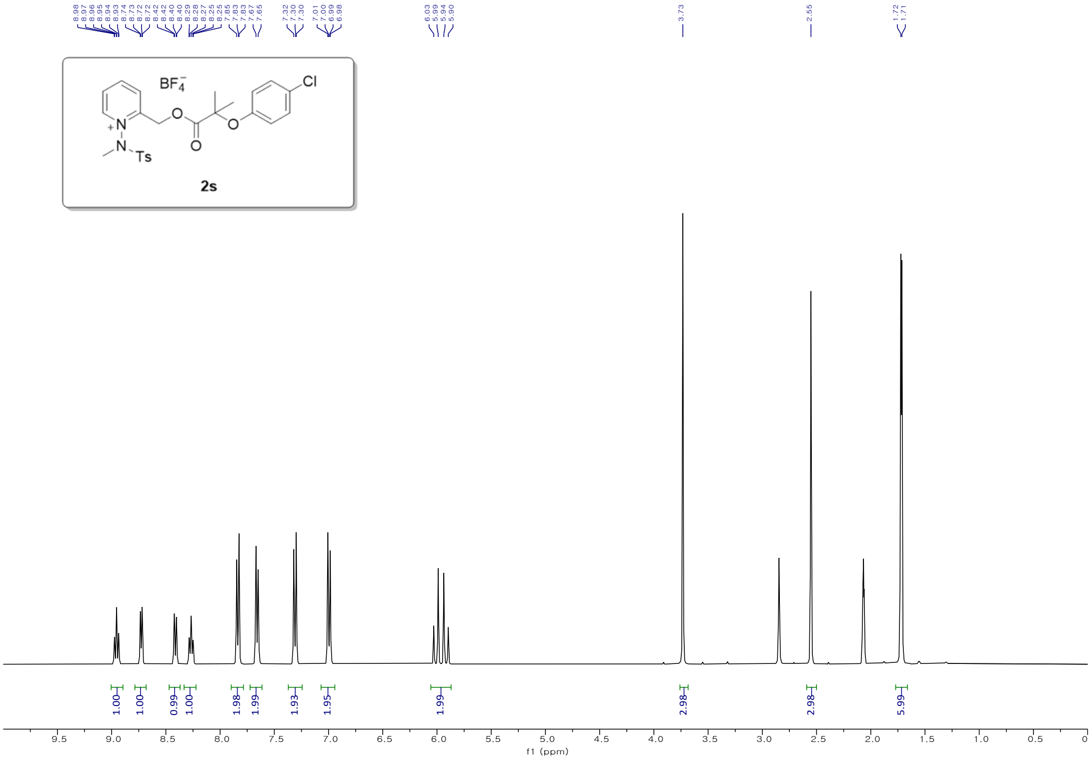

400 MHz, ^{1}H NMR in C_3D_6O

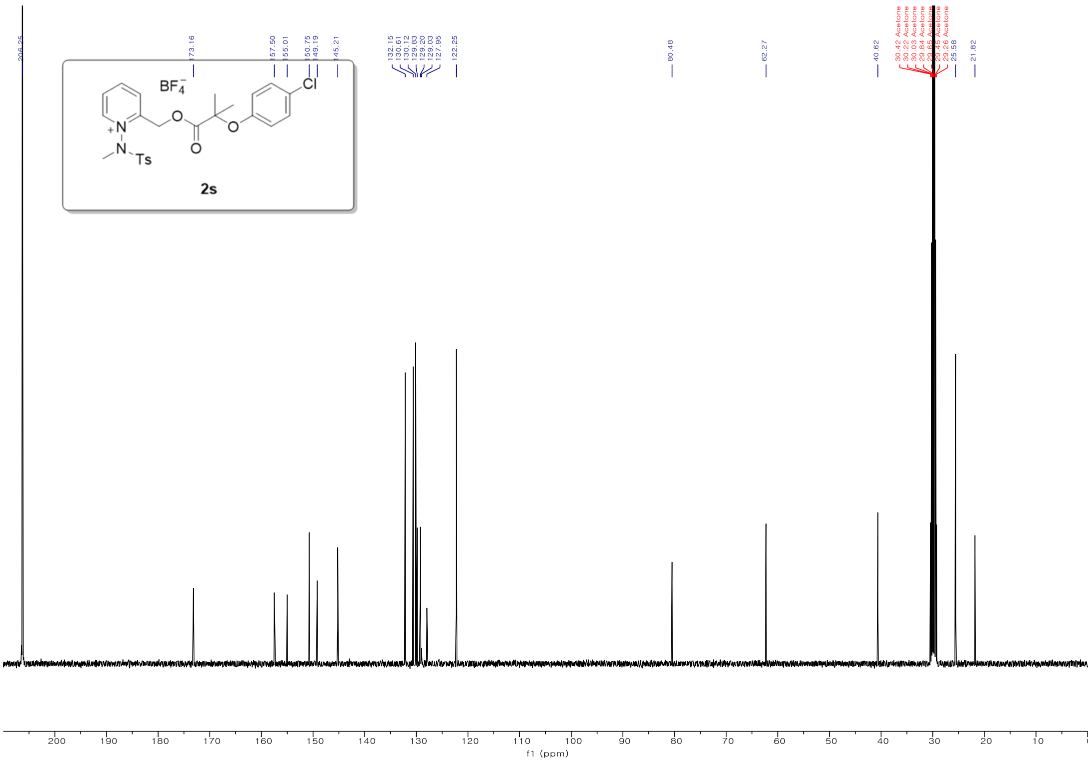

100 MHz, ^{13}C NMR in C_3D_6O

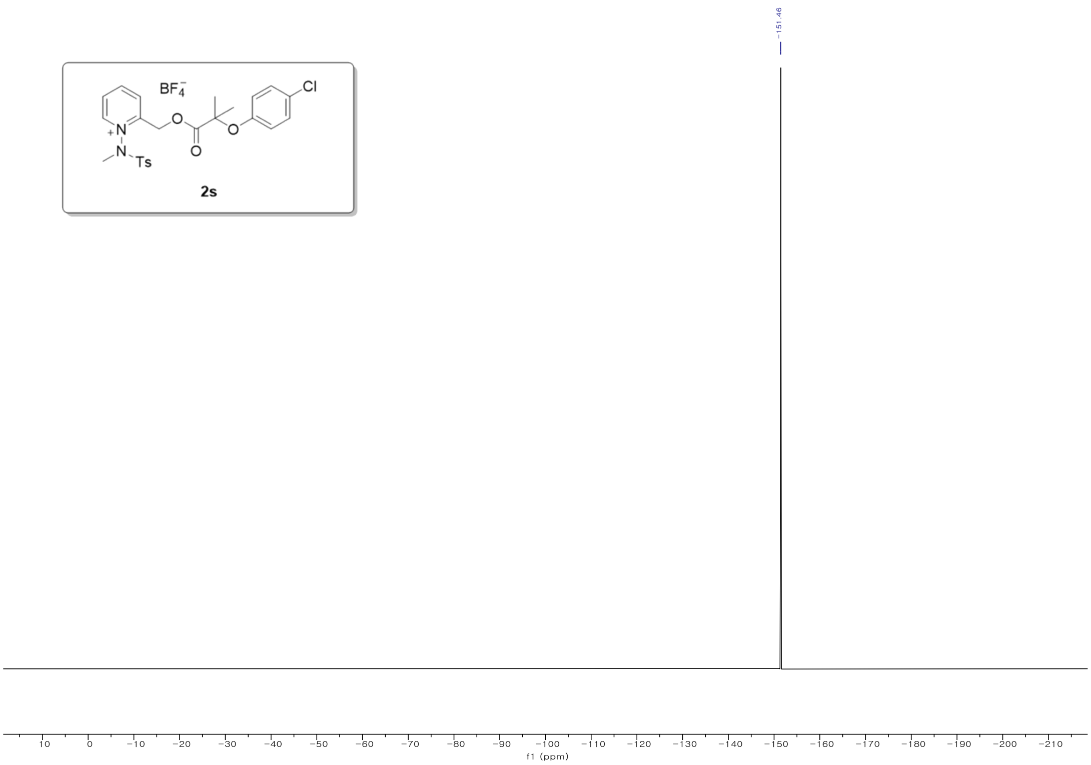

376 MHz, ^{19}F NMR in C_3D_6O

1-((N,4-dimethylphenyl)sulfonamido)-2-(((2-propylpentanoyl)oxy)methyl)pyridin-1-ium tetrafluoroborate (2t).

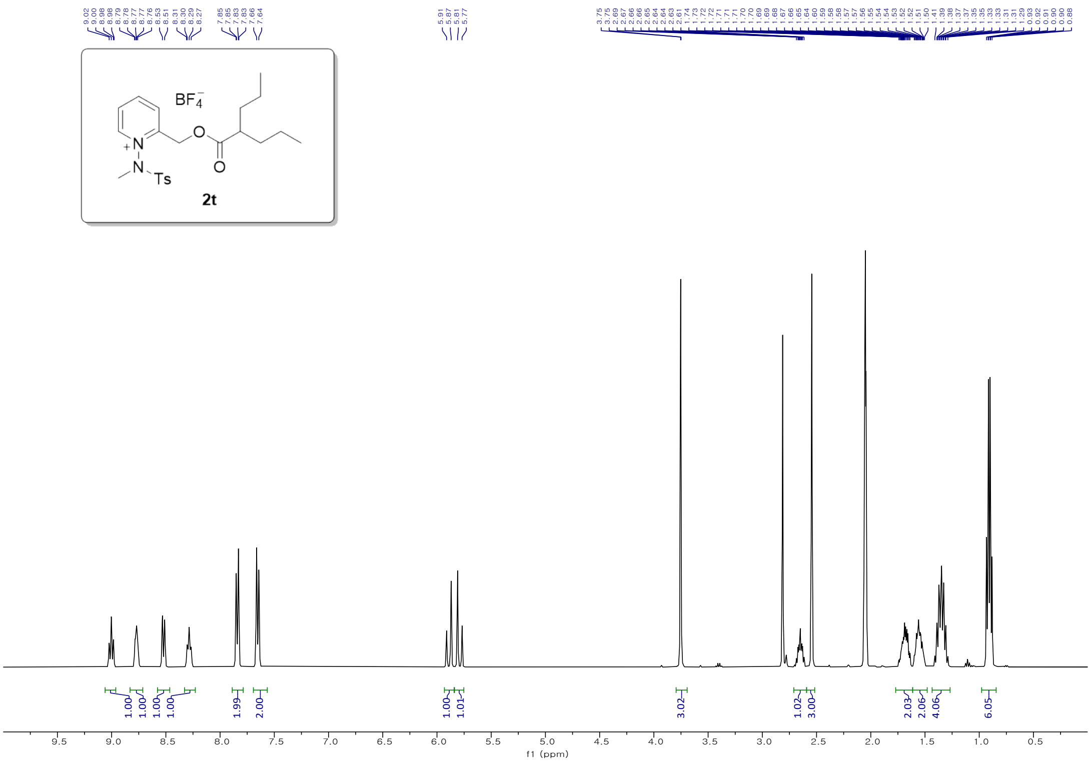

400 MHz, ^{1}H NMR in C_3D_6O

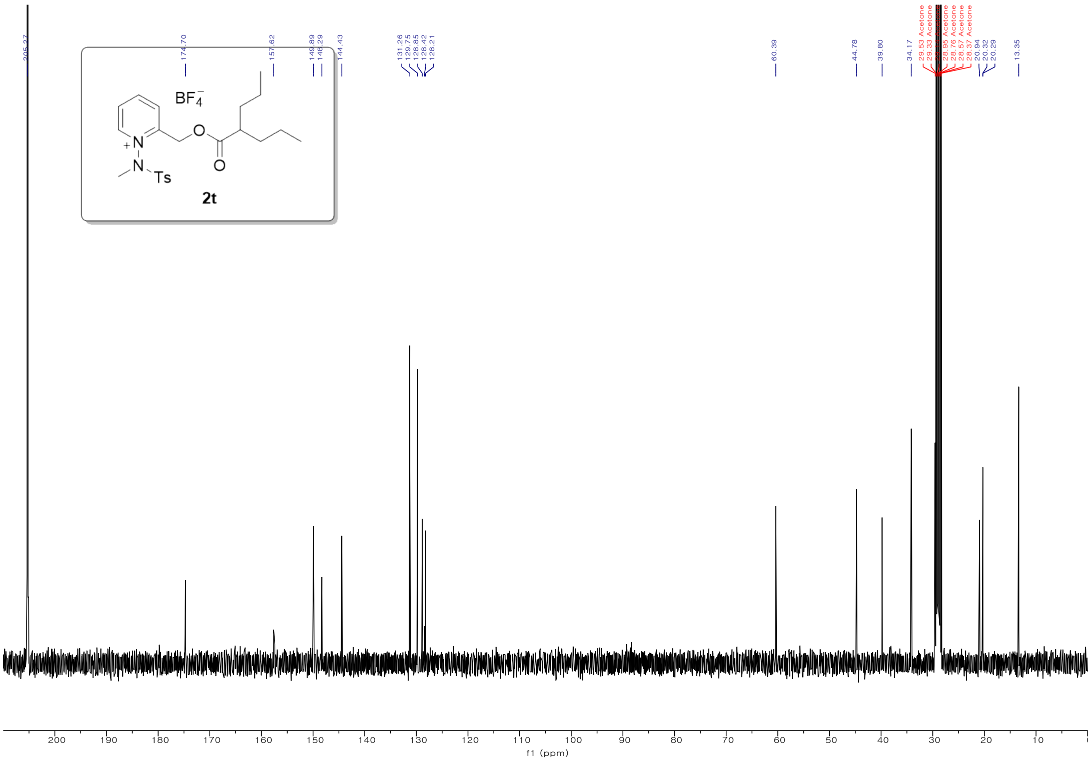

100 MHz, ^{13}C NMR in C_3D_6O

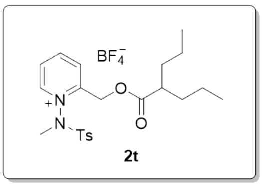

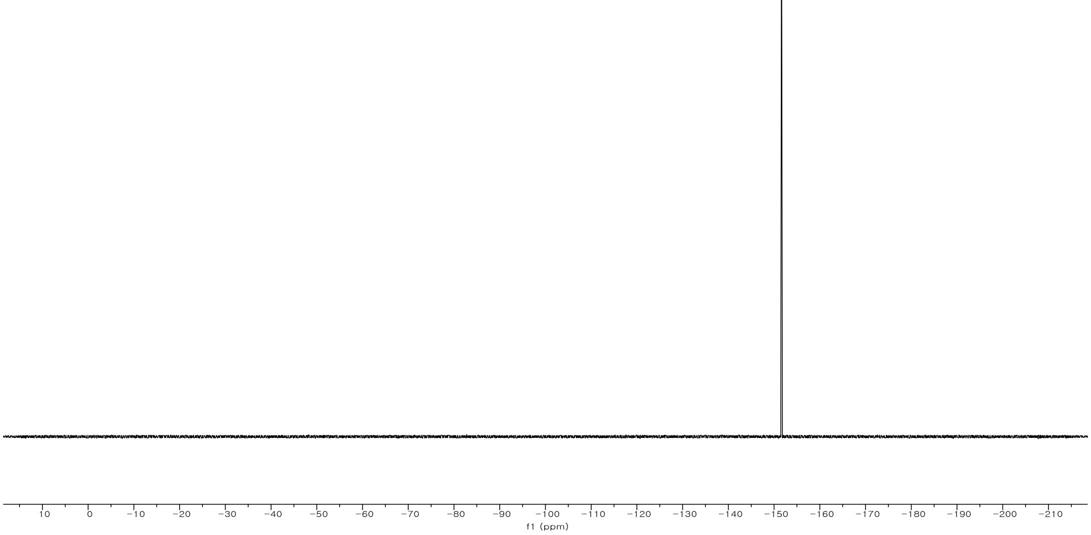

376 MHz, ^{19}F NMR in C_3D_6O

4-methoxy-N-(1-methoxy-3-(2-phenylpyridin-4-yl)propyl)benzamide (3a)

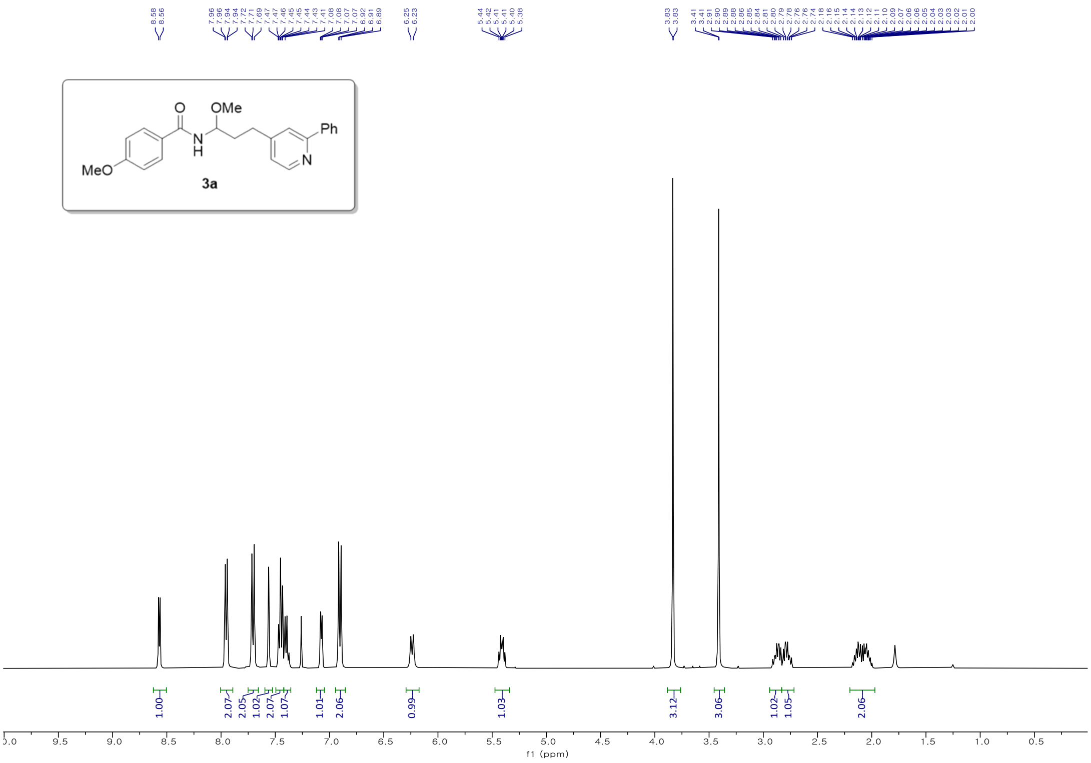

400 MHz, ^{1}H NMR in CDCl$_3$

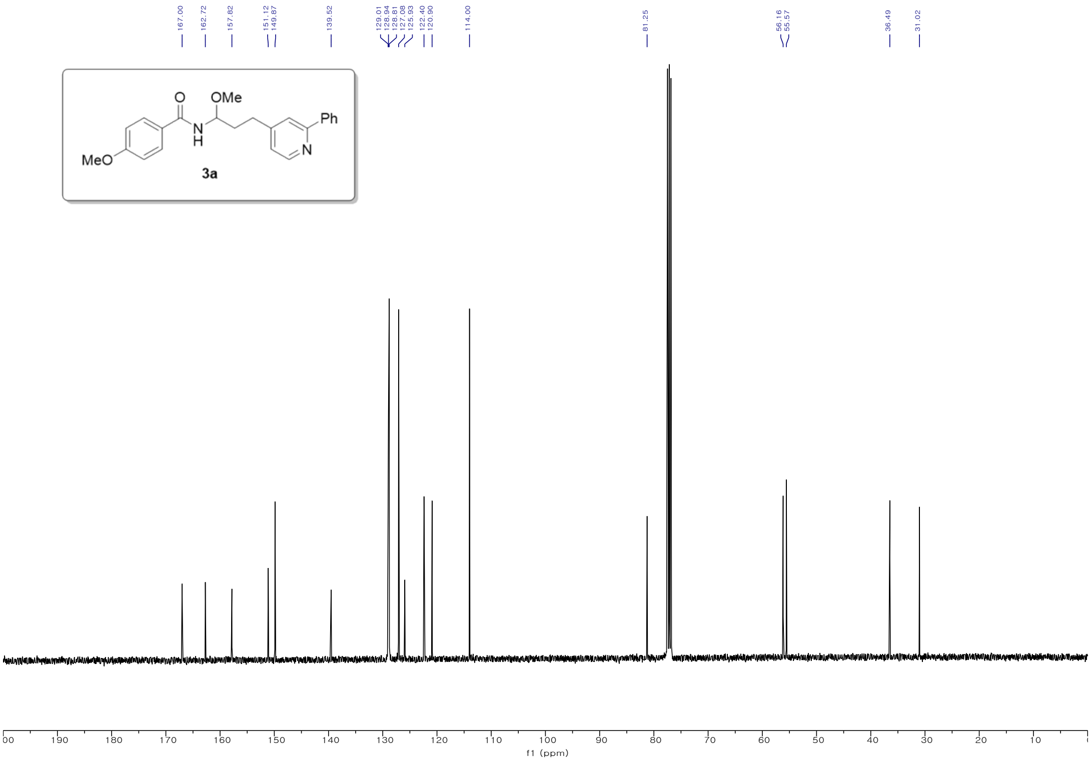

100 MHz, ^{13}C NMR in CDCl$_3$

4-methoxy-N-(1-methoxy-3-(2-methylpyridin-4-yl)propyl)benzamide (3b)

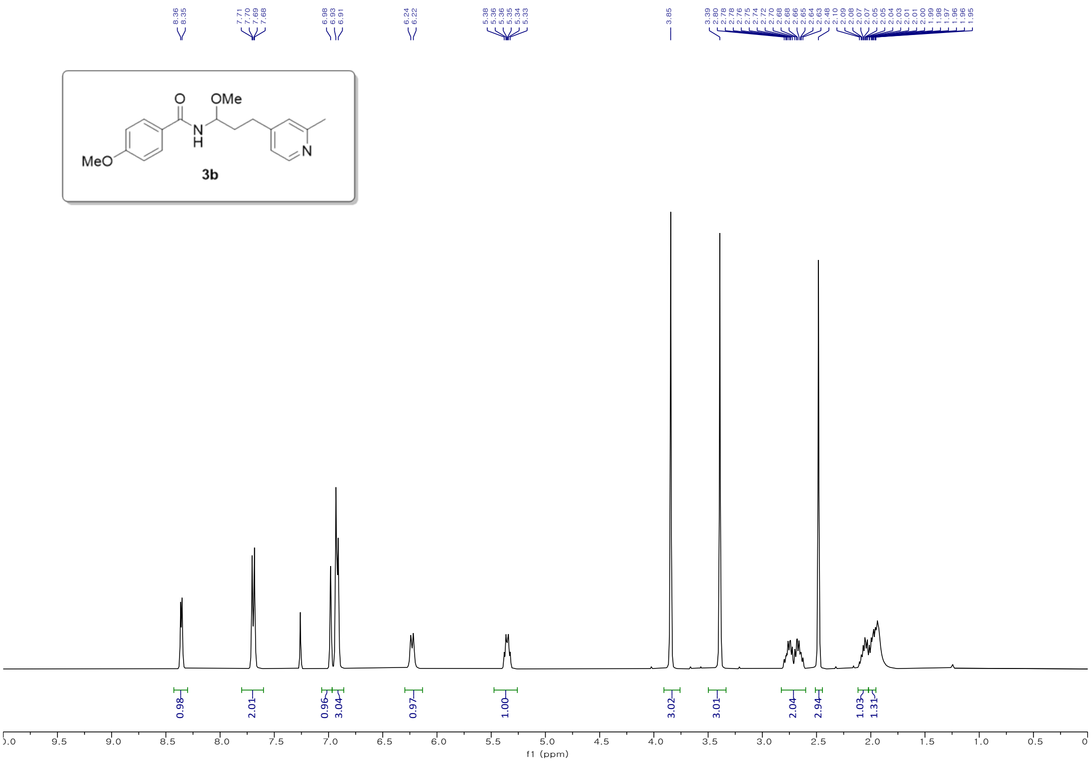

400 MHz, ^{1}H NMR in $CDCl_3$

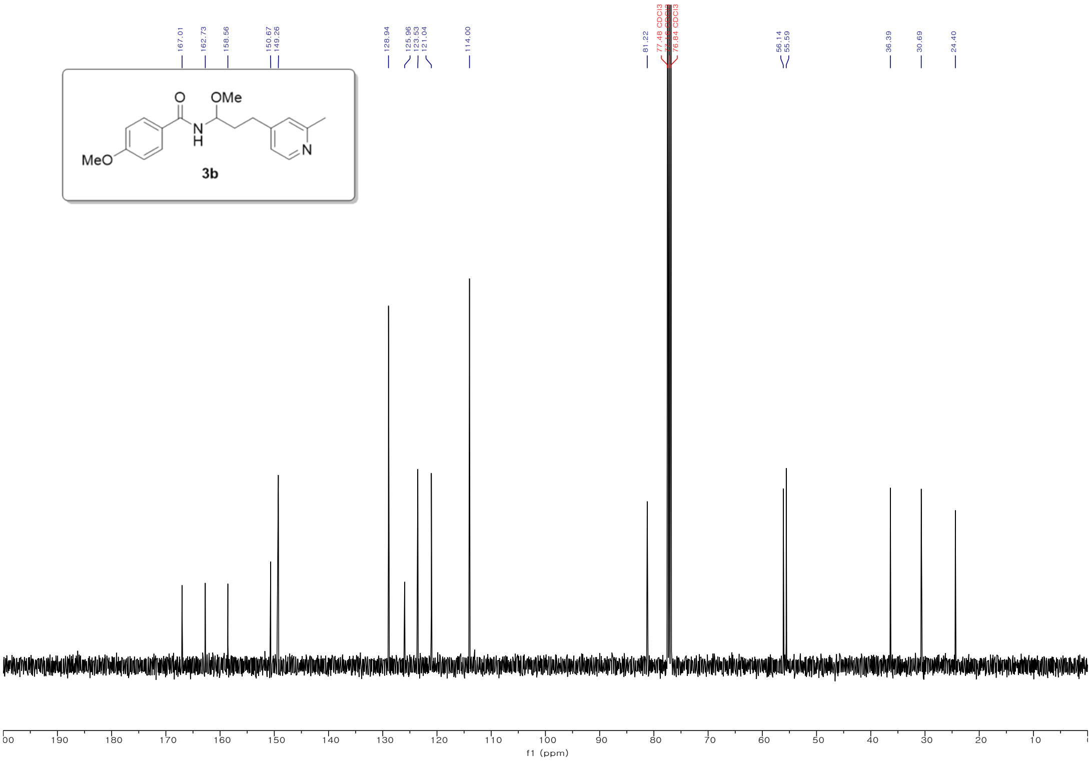

100 MHz, ^{13}C NMR in $CDCl_3$

methyl 4-(3-methoxy-3-(4-methoxybenzamido)propyl)picolinate (3c)

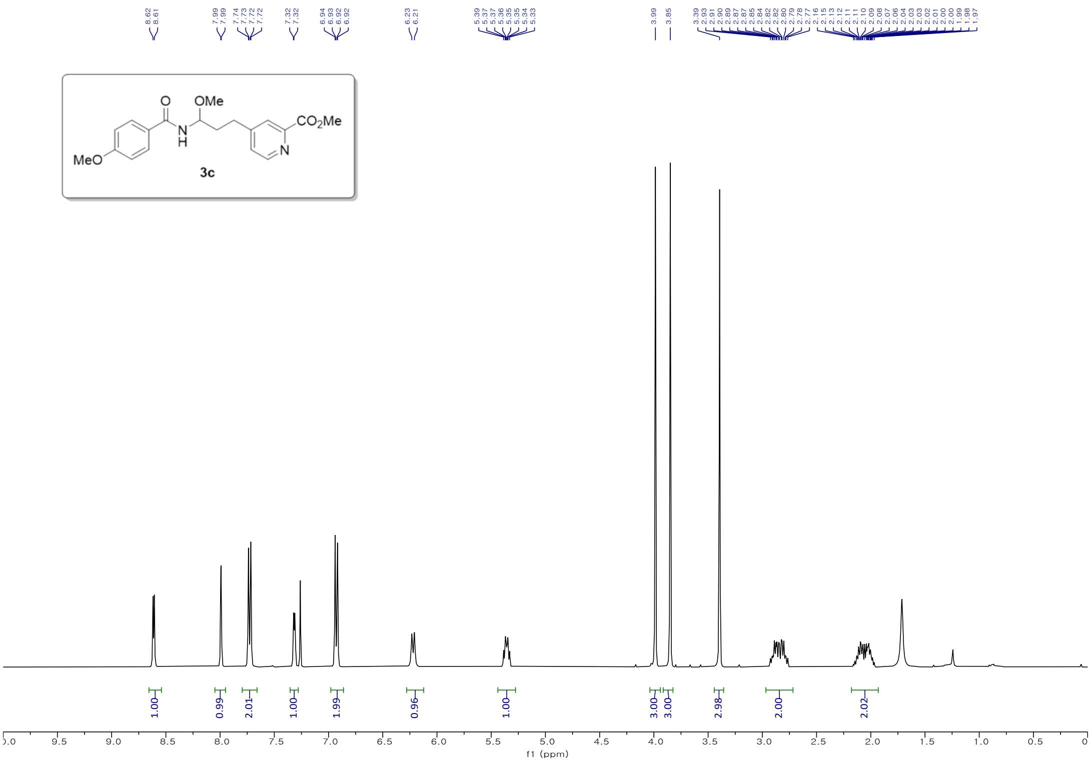

400 MHz, ^{1}H NMR in $CDCl_3$

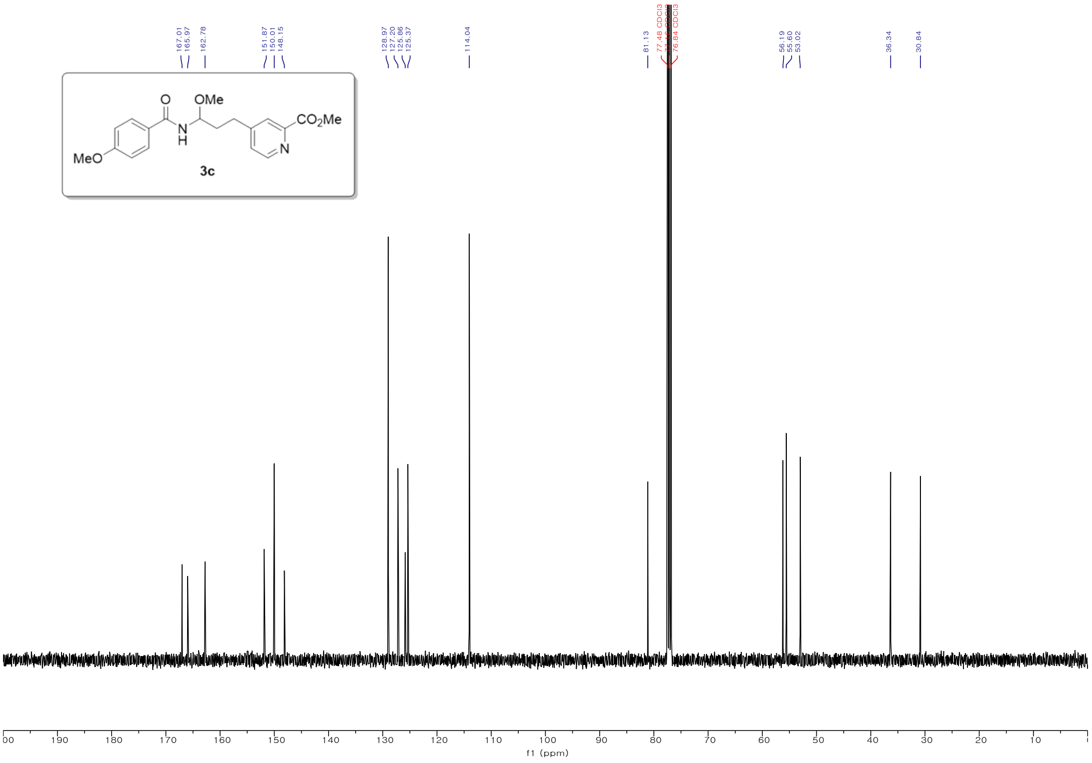

100 MHz, ^{13}C NMR in $CDCl_3$

4-methoxy-N-(1-methoxy-3-(2-(4-methoxyphenyl)pyridin-4-yl)propyl)benzamide (3d).

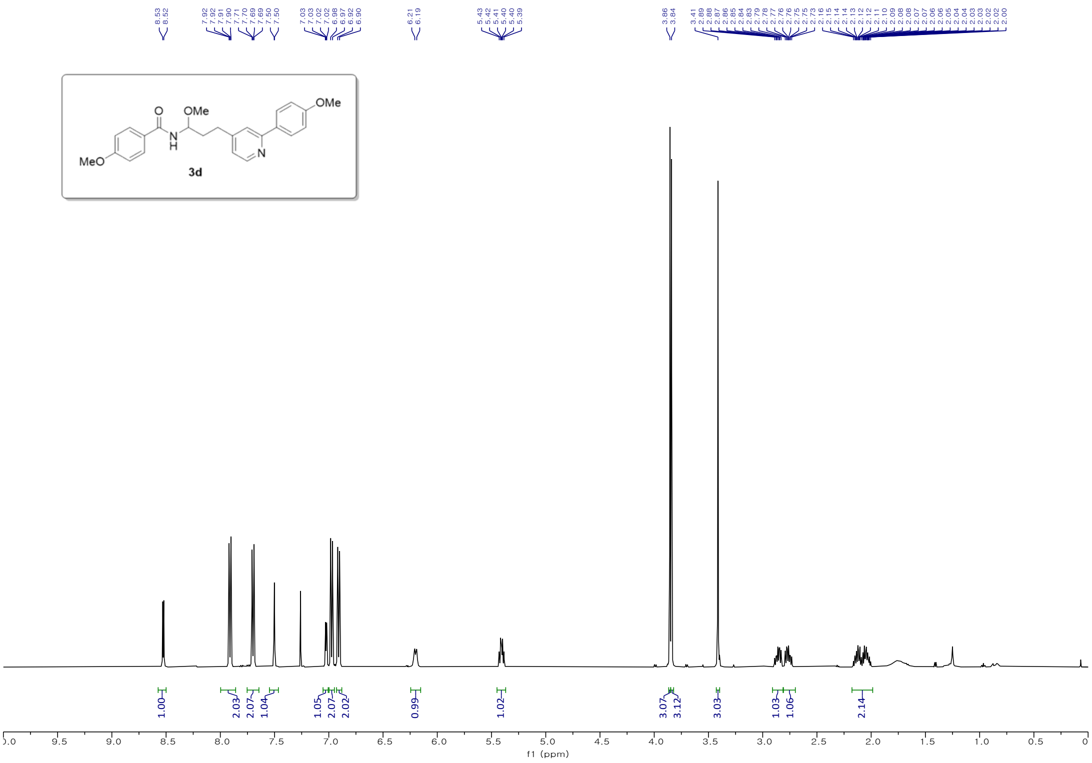

500 MHz, ^{1}H NMR in $CDCl_{3i}$

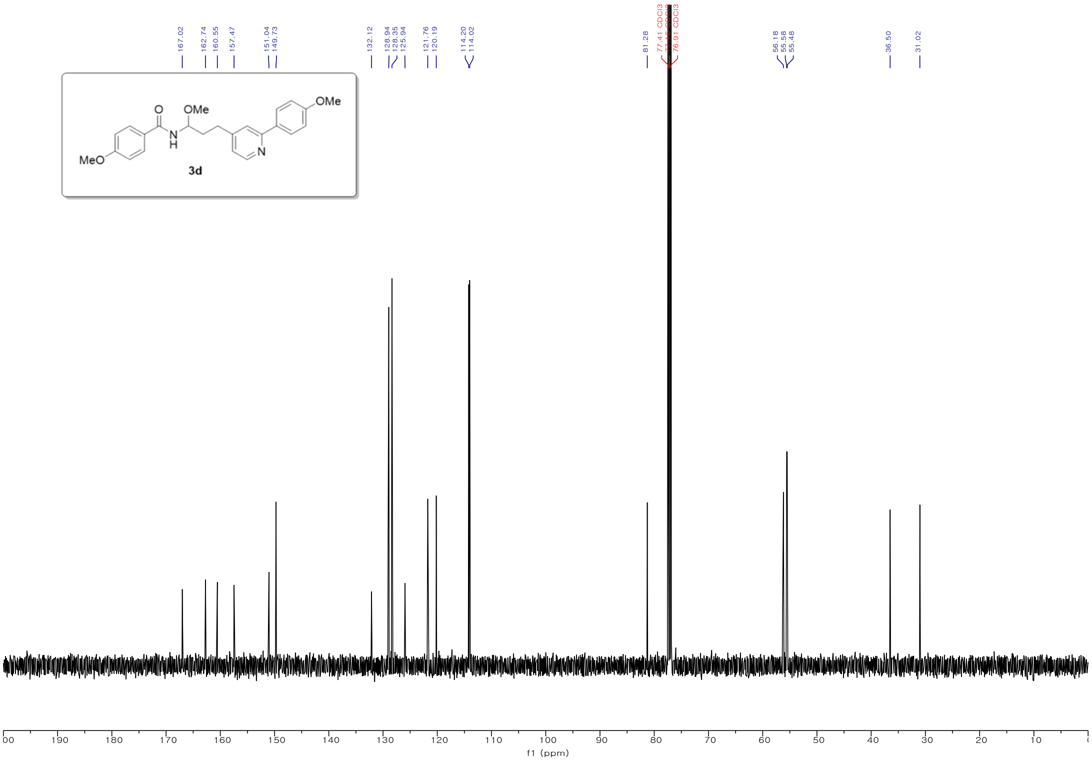

125 MHz, ^{13}C NMR in $CDCl_3$

4-methoxy-N-(1-methoxy-3-(2-(4-(trifluoromethyl)phenyl)pyridin-4-yl)propyl)benzamide (3e)

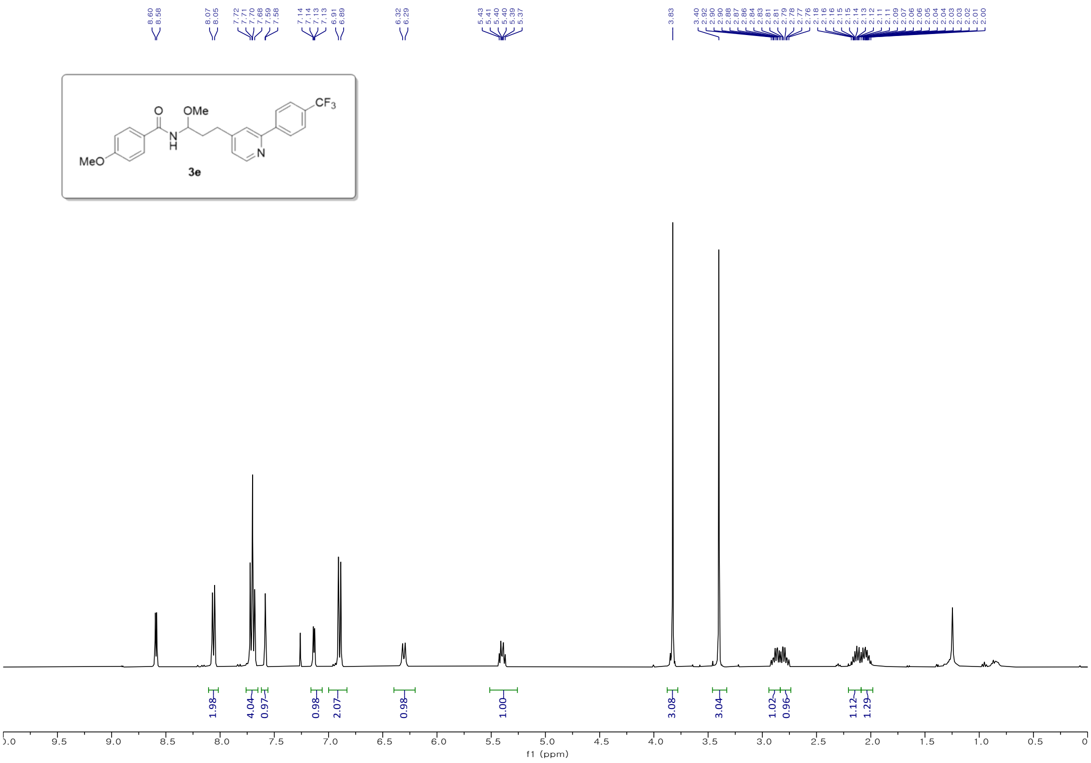

400 MHz, ^{1}H NMR in $CDCl_3$

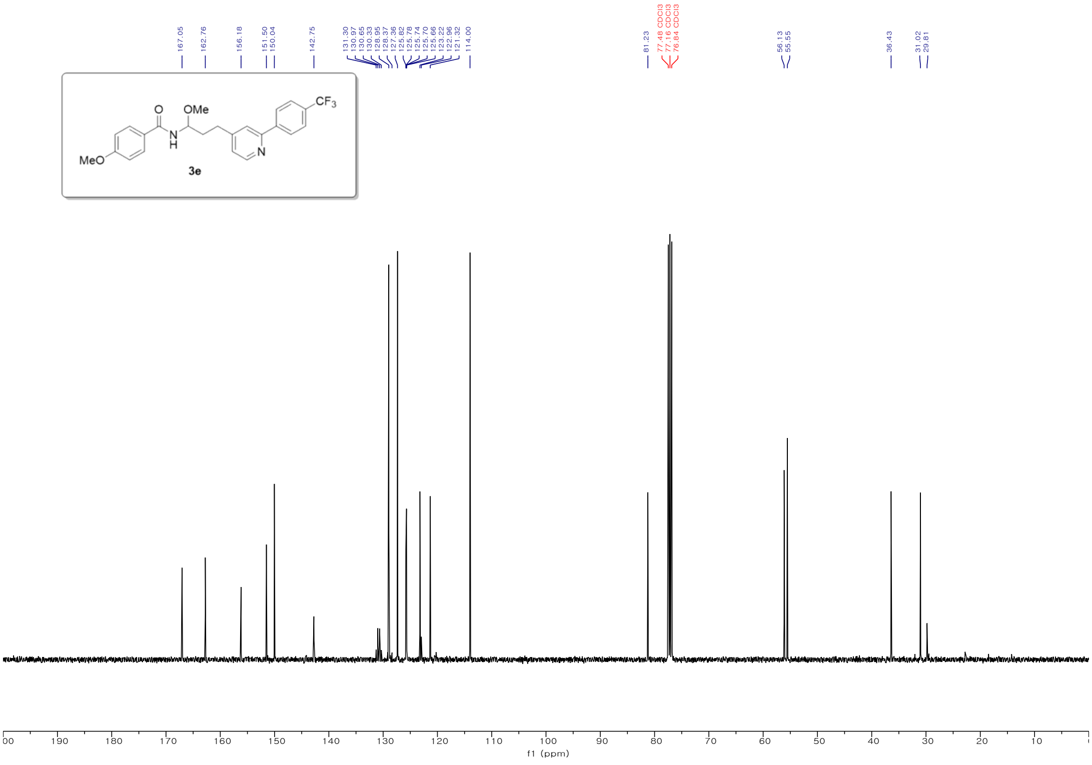

100 MHz, ^{13}C NMR in $CDCl_3$

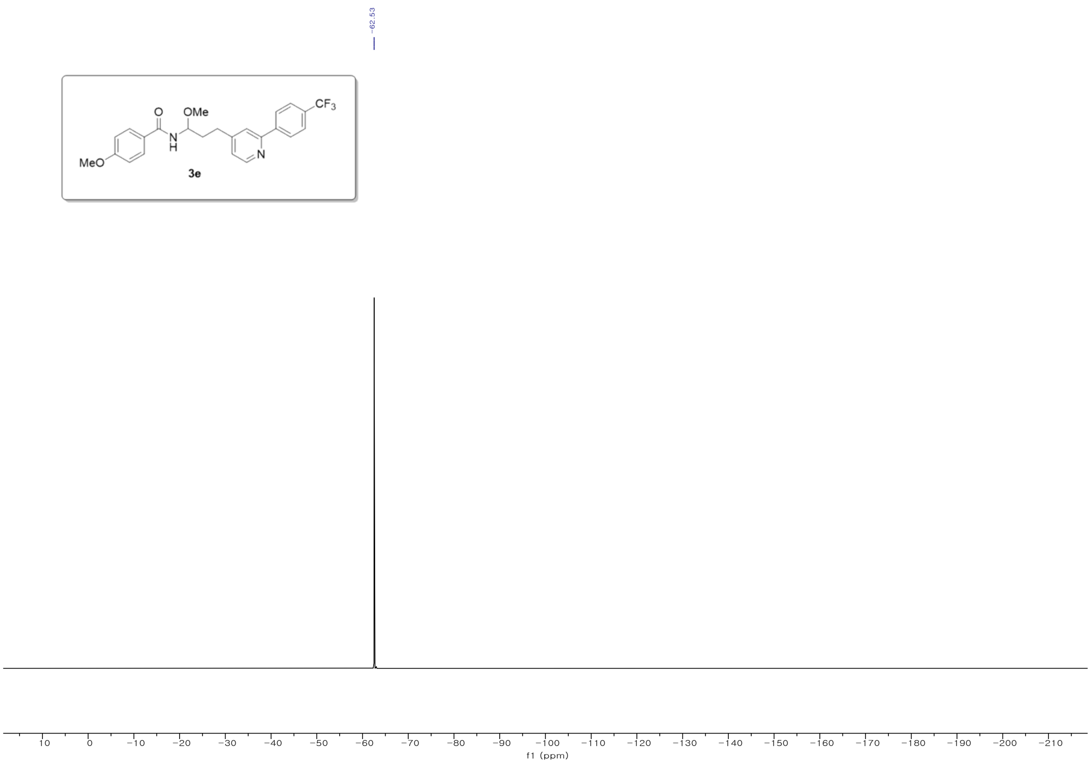

376 MHz, ^{19}F NMR in $CDCl_3$

N-(3-([2,2'-bipyridin]-4-yl)-1-methoxypropyl)-4-methoxybenzamide (3f).

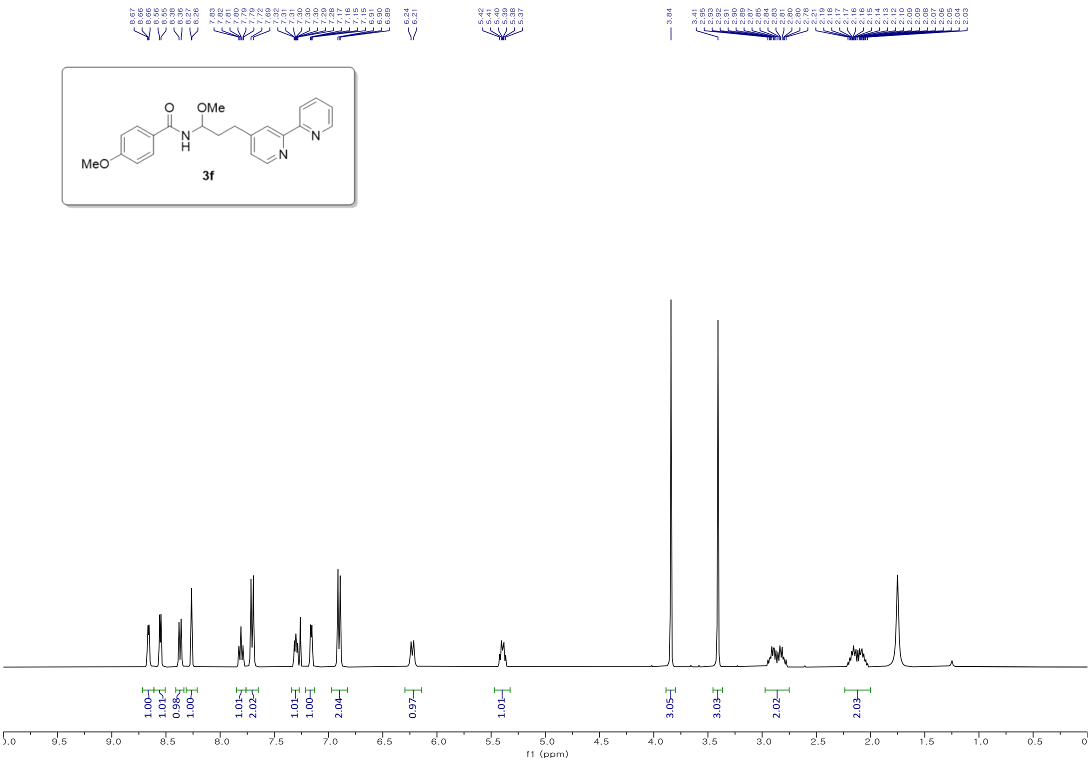

400 MHz, ^{1}H NMR in $CDCl_3$

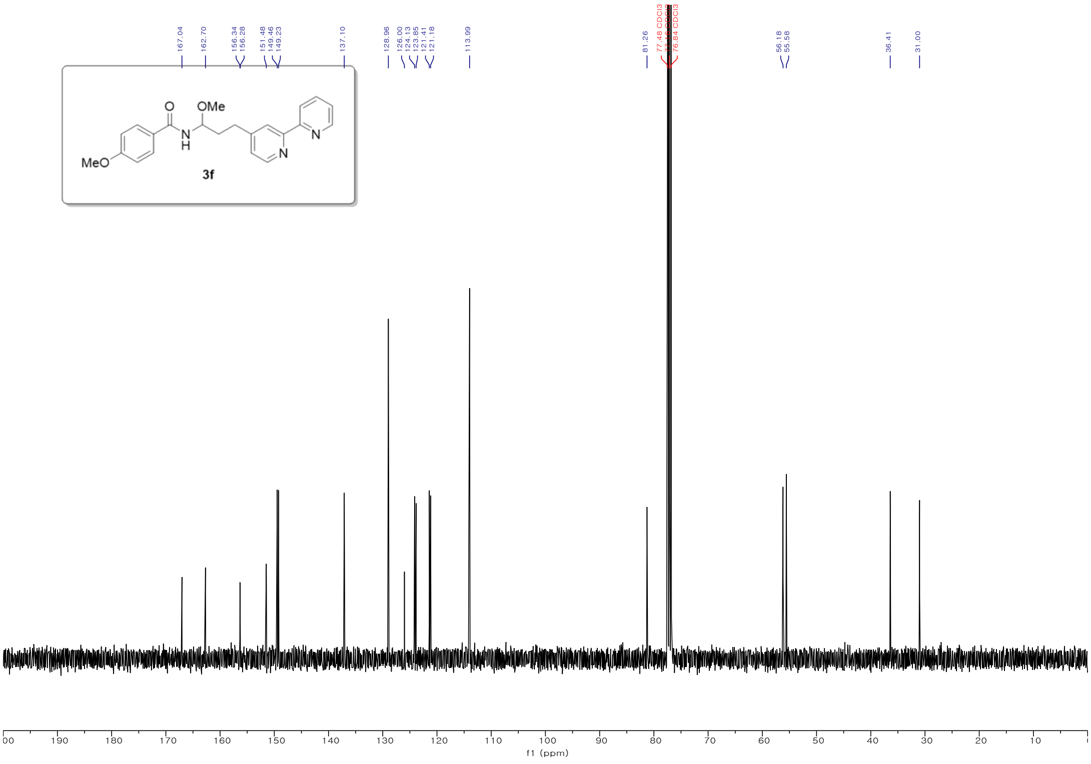

100 MHz, ^{13}C NMR in $CDCl_3$

4-methoxy-N-(1-methoxy-3-(3-methylpyridin-4-yl)propyl)benzamide (3g)

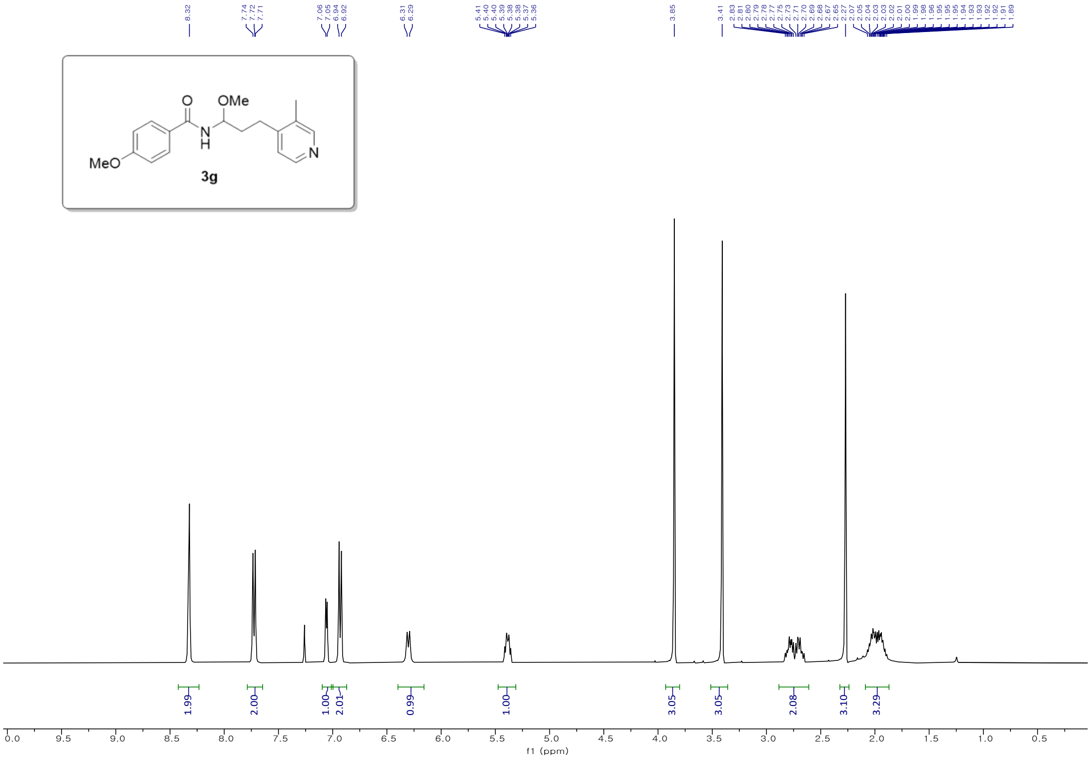

400 MHz, ^{1}H NMR in $CDCl_3$

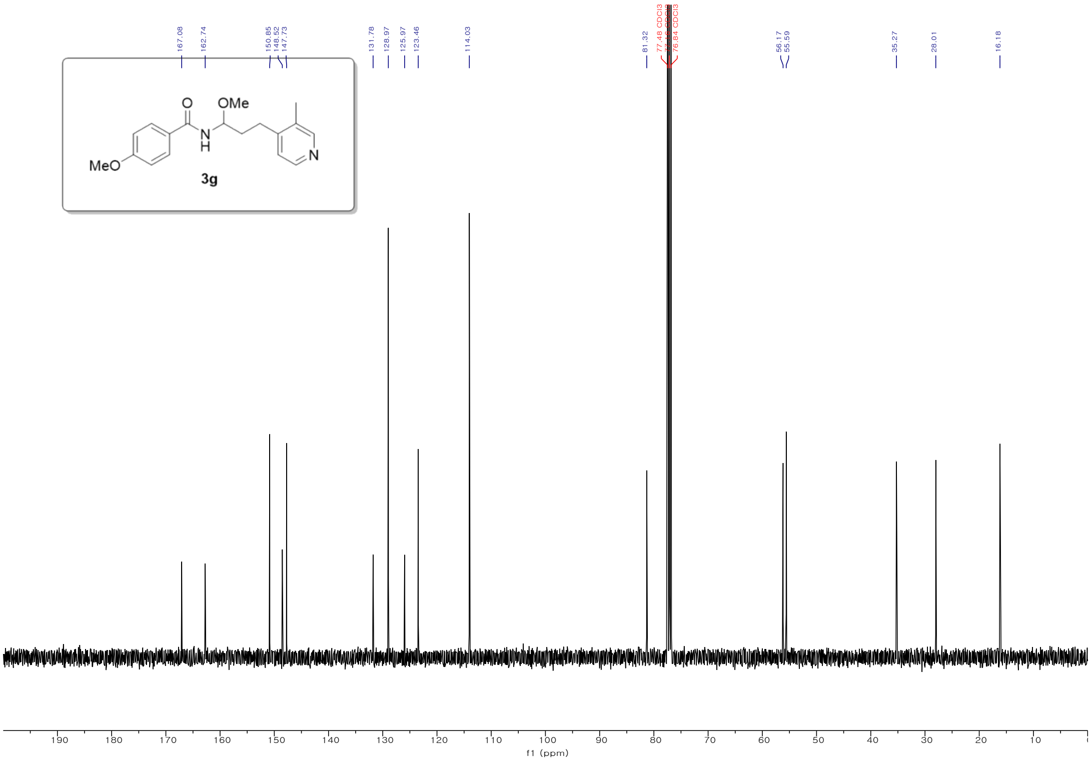

100 MHz, ^{13}C NMR in $CDCl_3$

N-(3-(3-iodopyridin-4-yl)-1-methoxypropyl)-4-methoxybenzamide (3h).

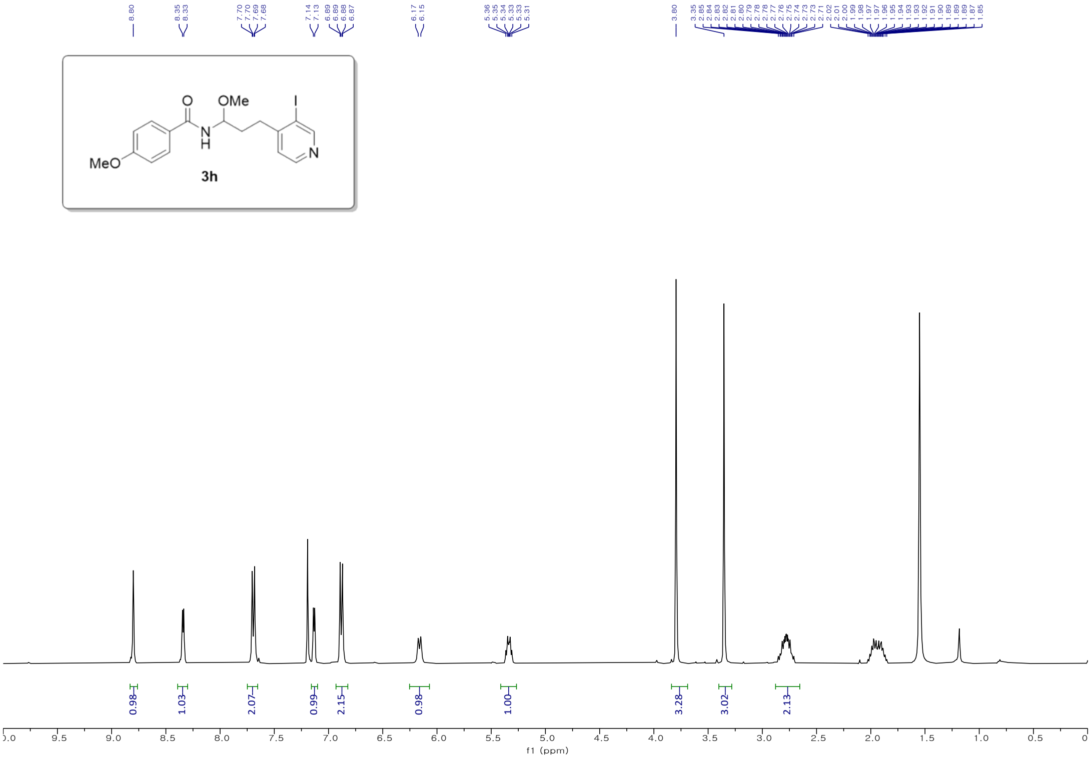

400 MHz, ^{1}H NMR in $CDCl_3$

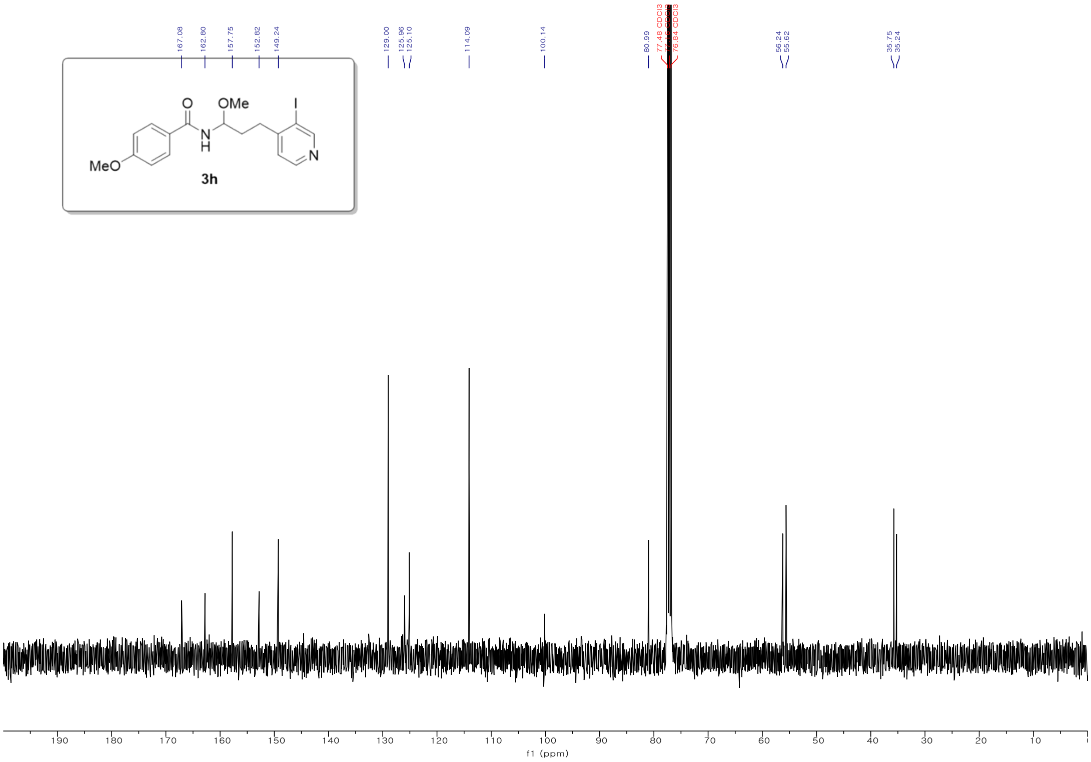

100 MHz, ^{13}C NMR in $CDCl_3$

methyl 4-(3-methoxy-3-(4-methoxybenzamido)propyl)nicotinate (3i).

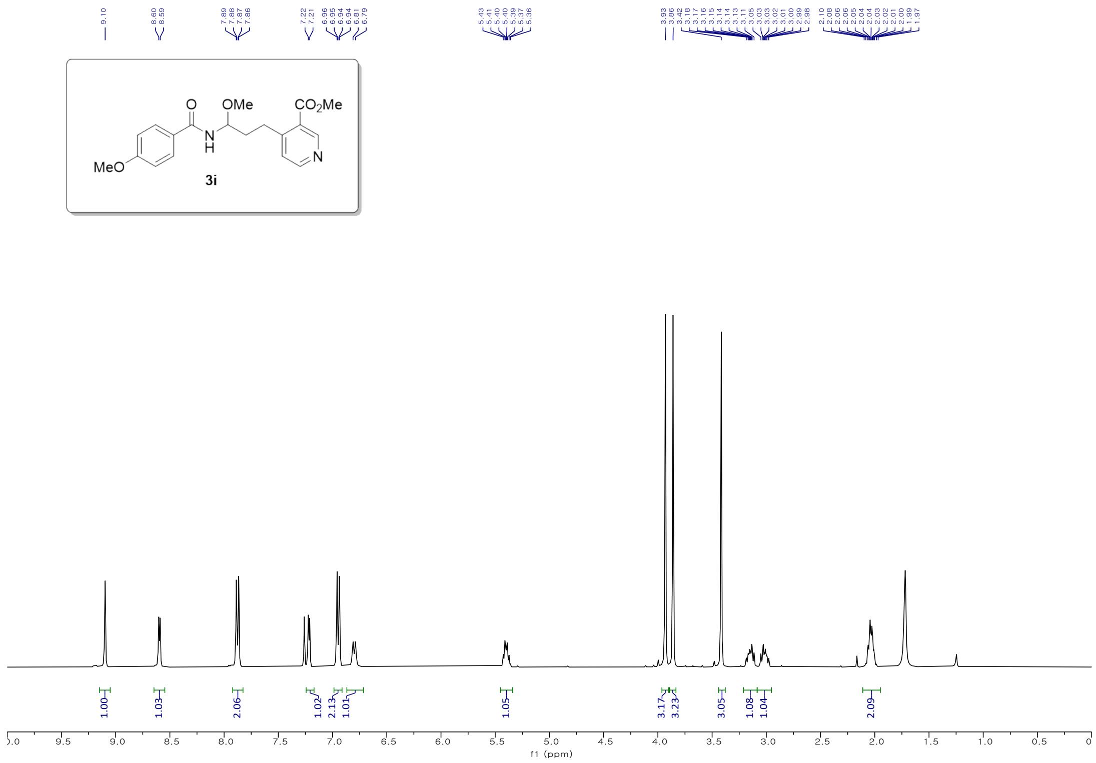

400 MHz, ^{1}H NMR in $CDCl_3$

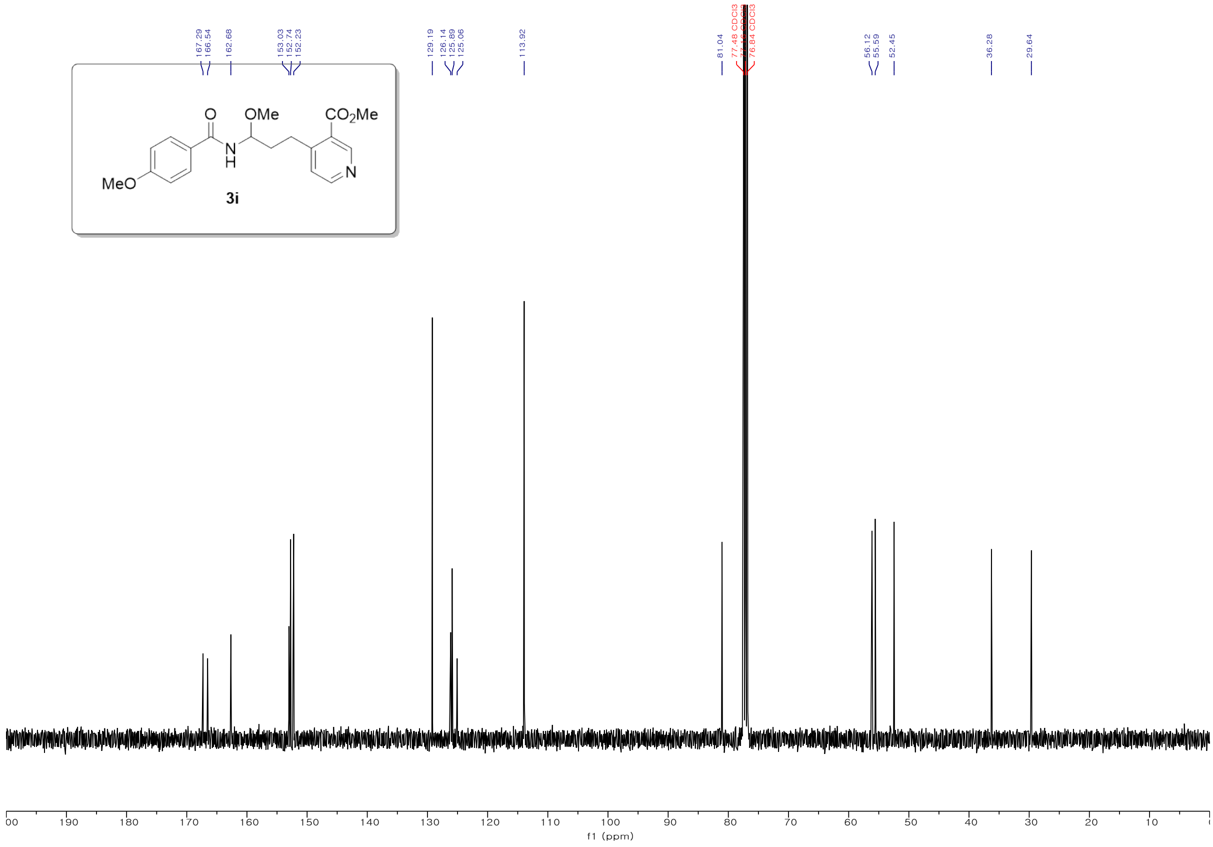

100 MHz, ^{13}C NMR in $CDCl_3$

4-methoxy-N-(1-methoxy-3-(3-methyl-2-phenylpyridin-4-yl)propyl)benzamide (3j).

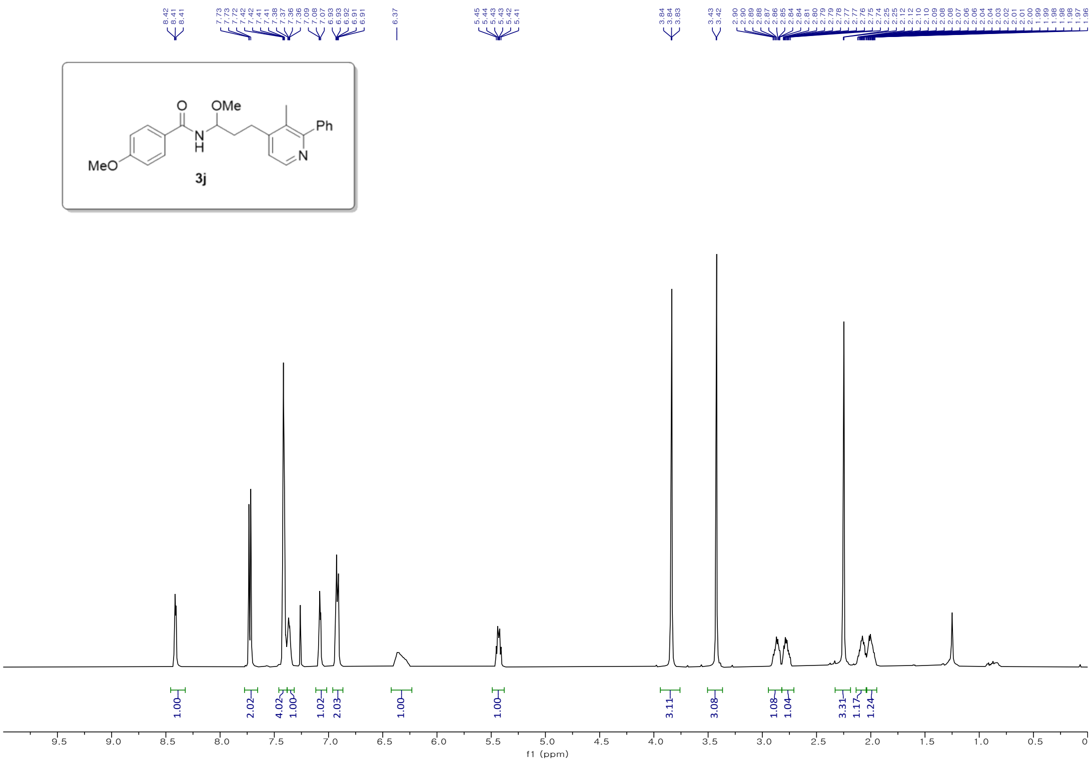

500 MHz, ^{1}H NMR in $CDCl_3$

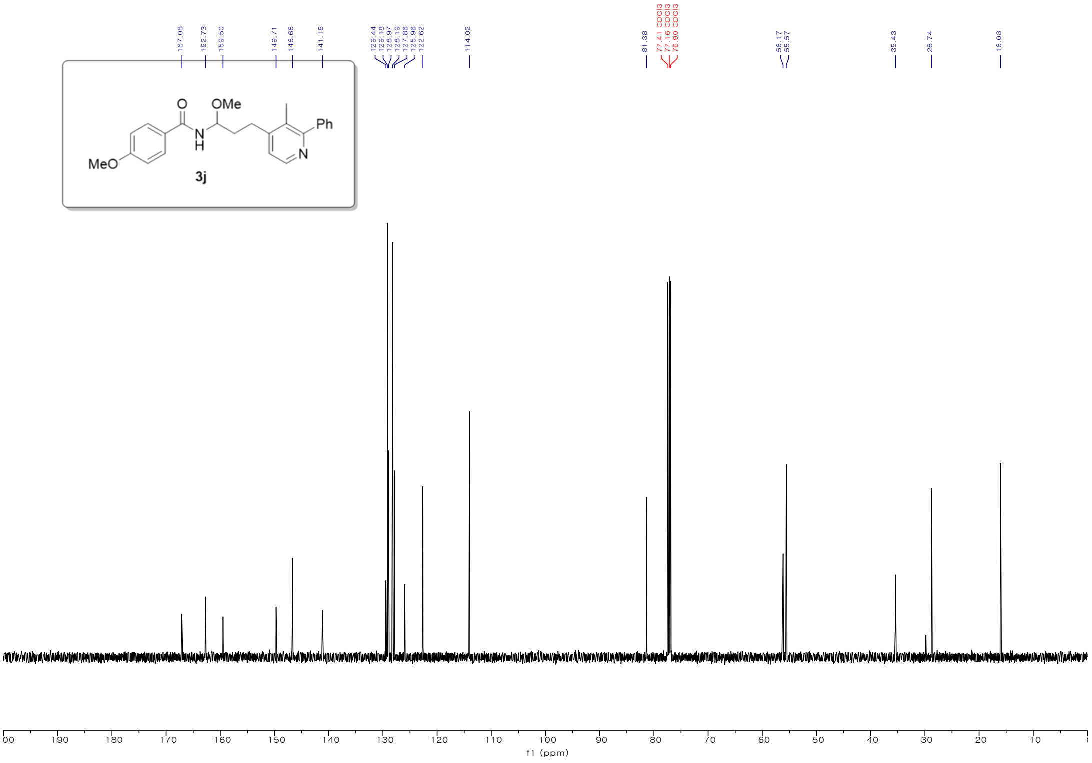

100 MHz, ^{13}C NMR in $CDCl_3$

N-(3-(5-bromo-2-phenylpyridin-4-yl)-1-methoxypropyl)-4-methoxybenzamide (3k).

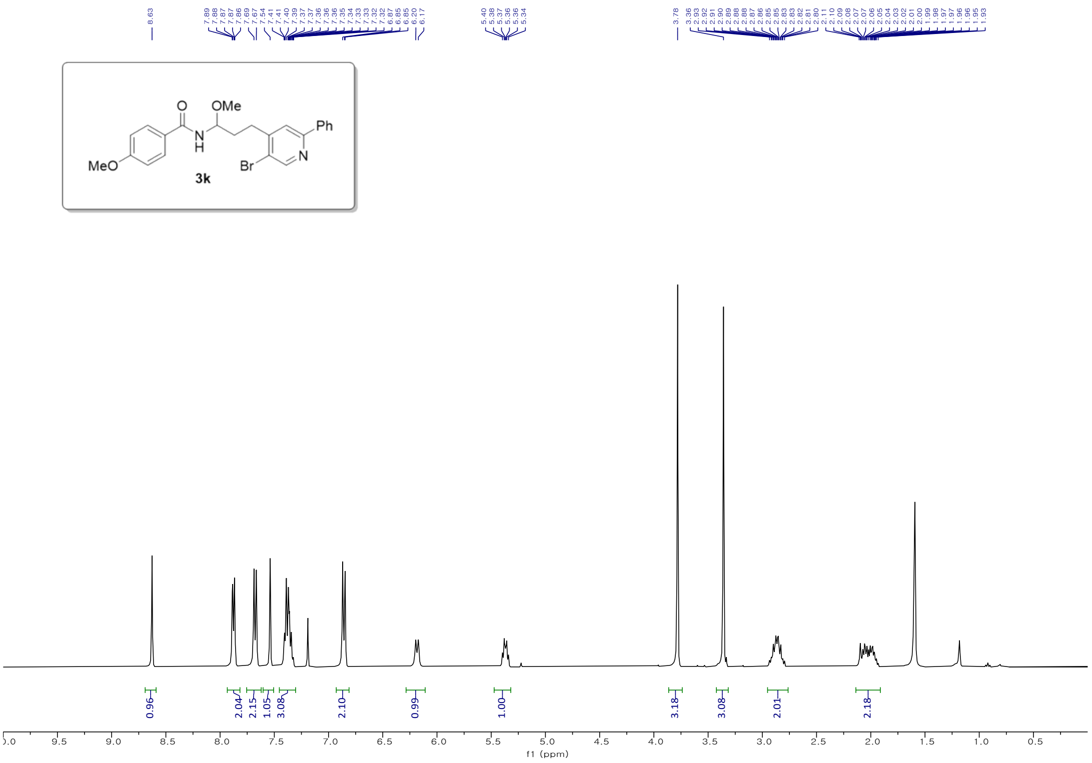

400 MHz, ^{1}H NMR in $CDCl_3$

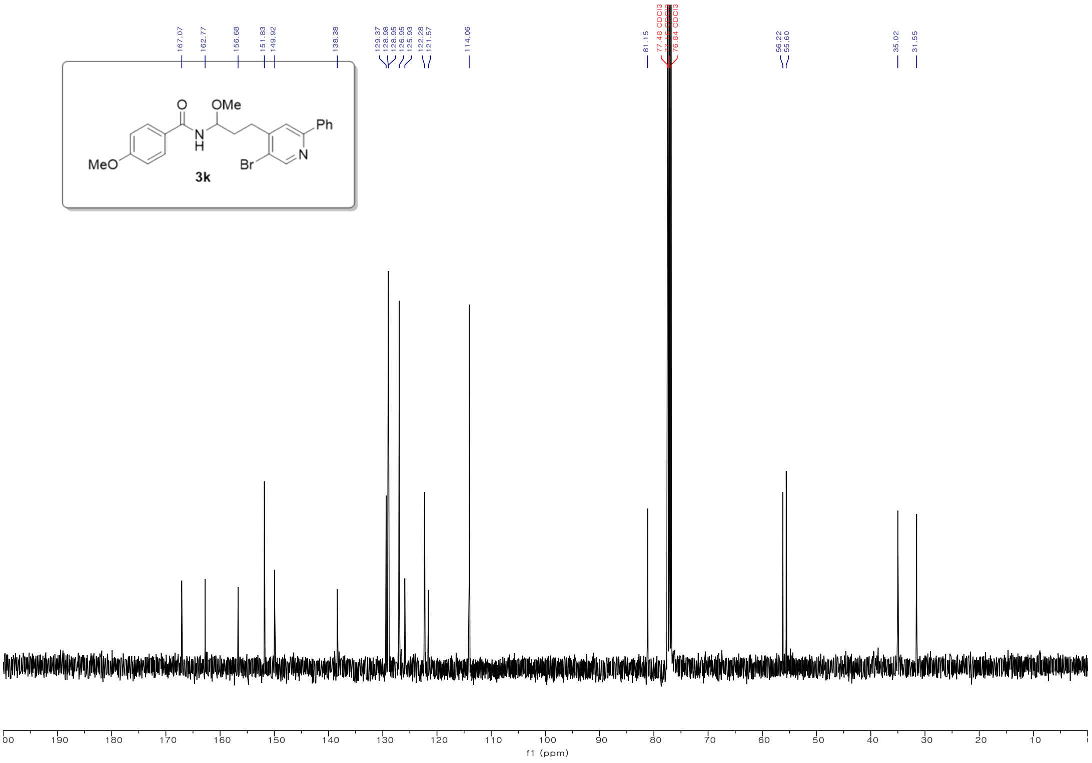

100 MHz, ^{13}C NMR in $CDCl_3$

4-methoxy-N-(1-methoxy-3-(2-methyl-6-phenylpyridin-4-yl)propyl)benzamide (3l).

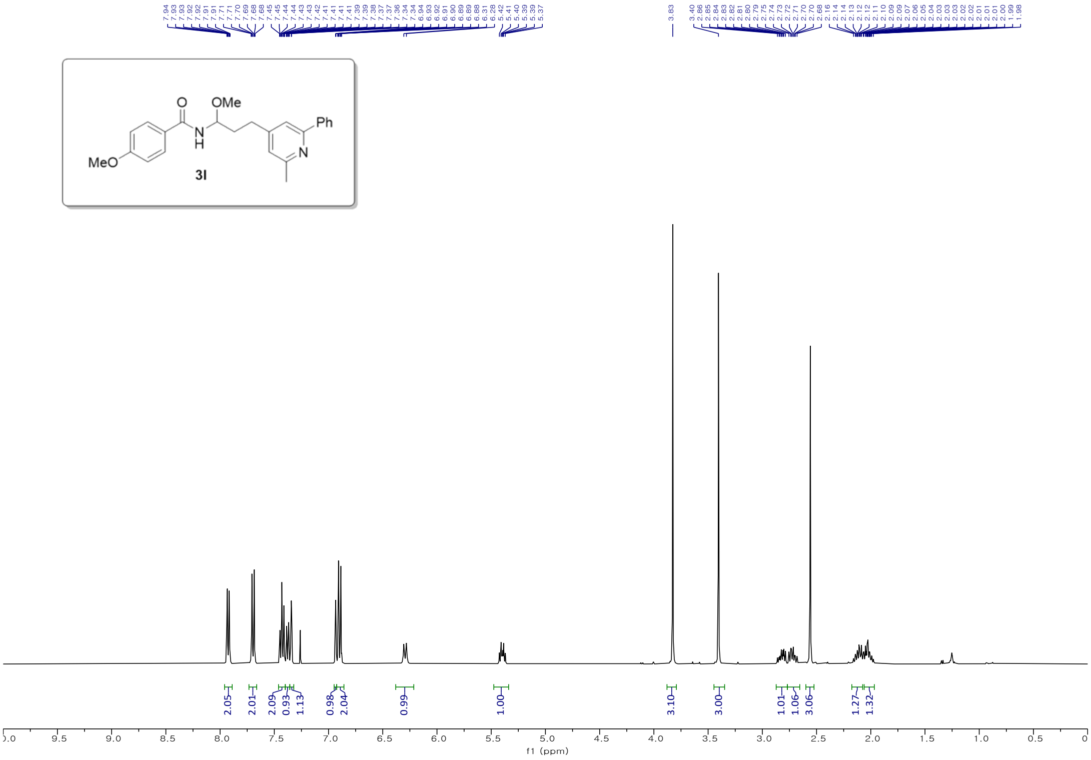

400 MHz, ^{1}H NMR in $CDCl_3$

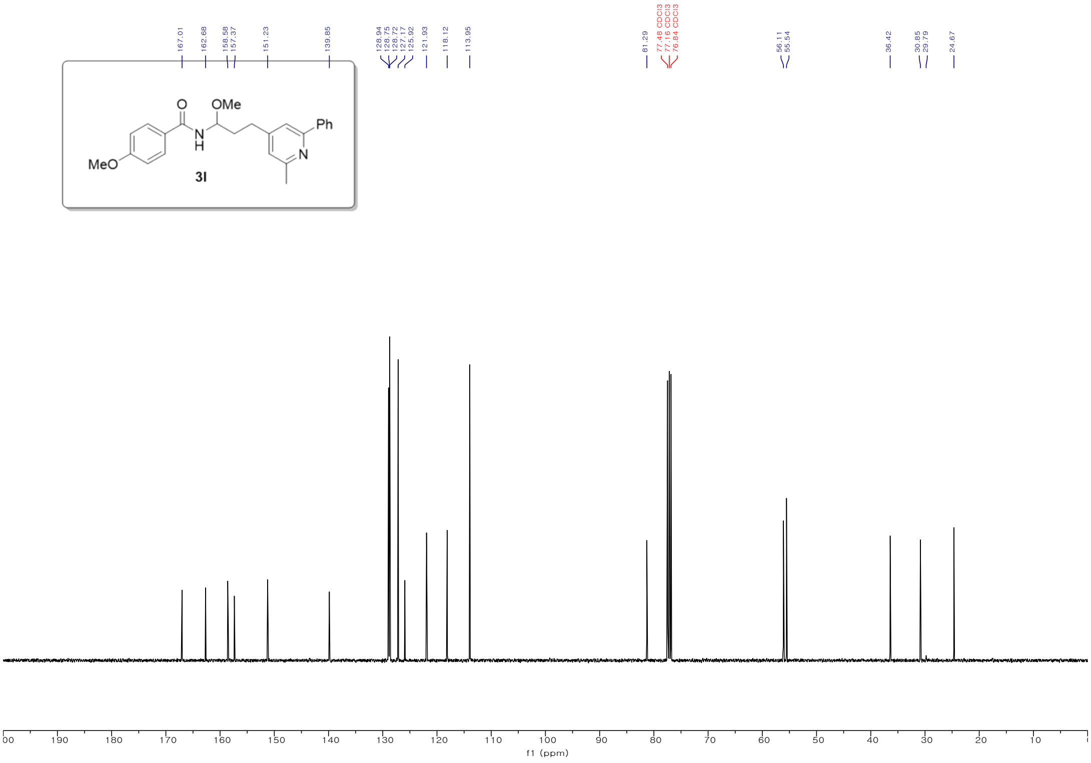

100 MHz, ^{13}C NMR in $CDCl_3$

4-methoxy-N-(1-methoxy-3-(5,6,7,8-tetrahydroquinolin-4-yl)propyl)benzamide (3m).

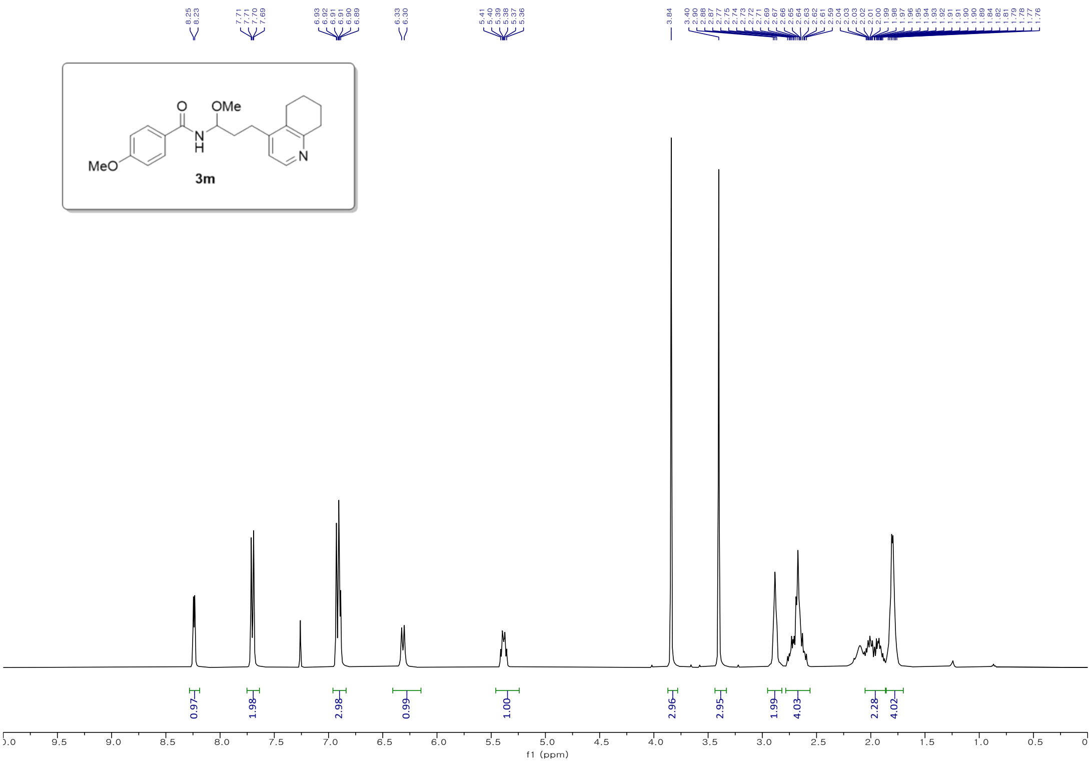

400 MHz, ^{1}H NMR in $CDCl_3$

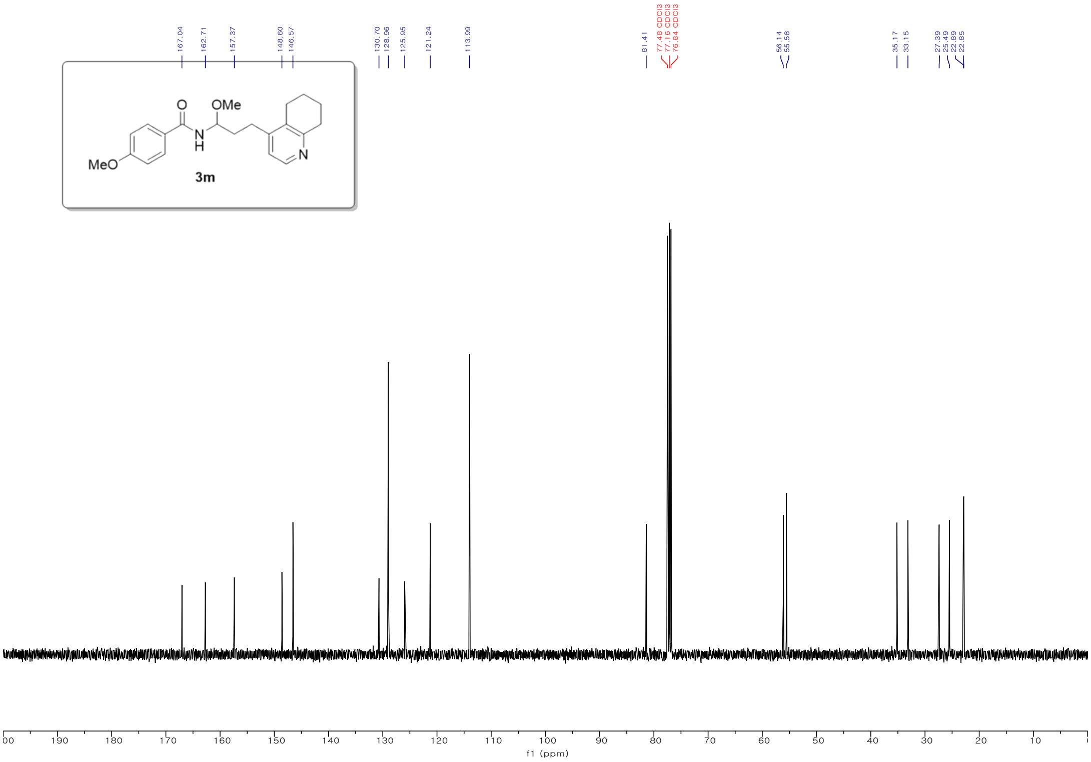

100 MHz, ^{13}C NMR in $CDCl_3$

4-methoxy-N-(1-methoxy-3-(pyridin-4-yl)propyl)benzamide (3n)

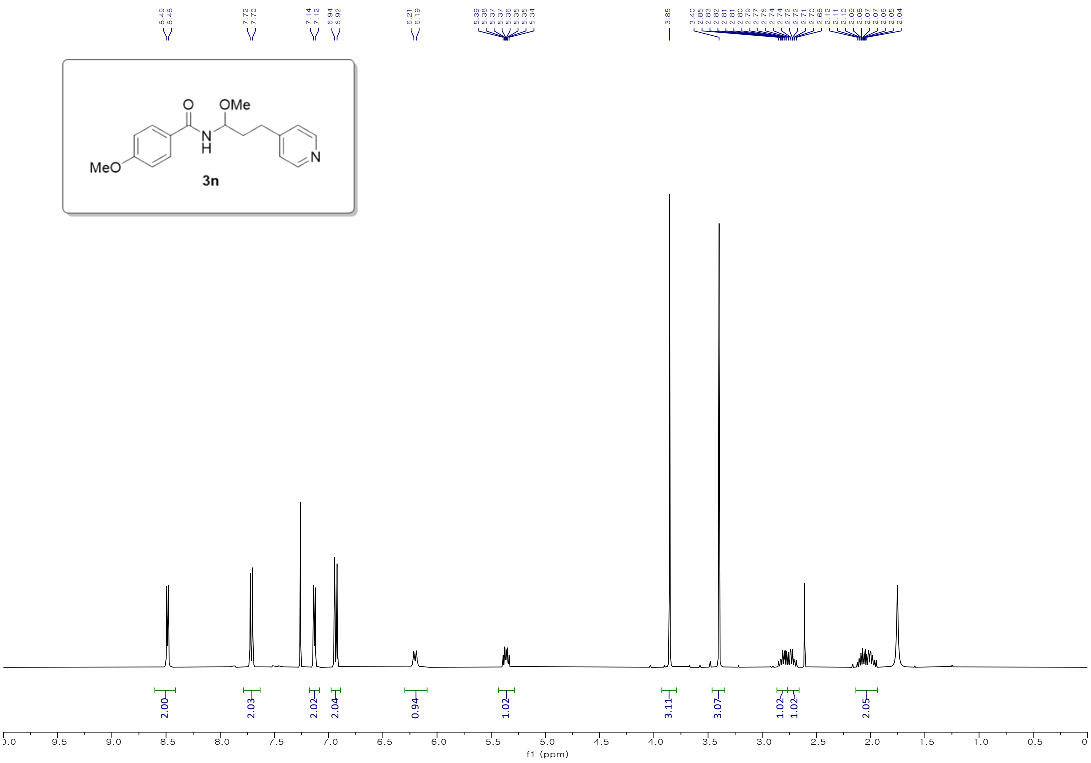

400 MHz, ^1H NMR in $CDCl_3$

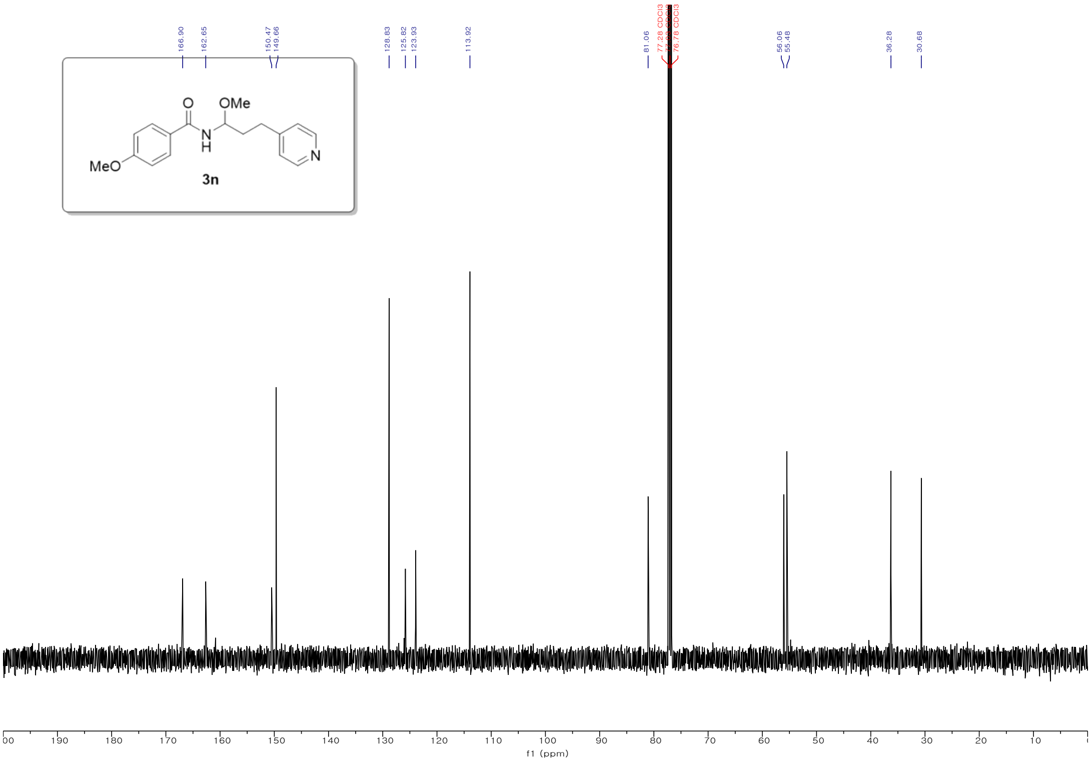

125 MHz, ^{13}C NMR in $CDCl_3$

4-methoxy-N-(1-methoxy-3-(2-phenylquinolin-4-yl)propyl)benzamidebenzamide (3o).

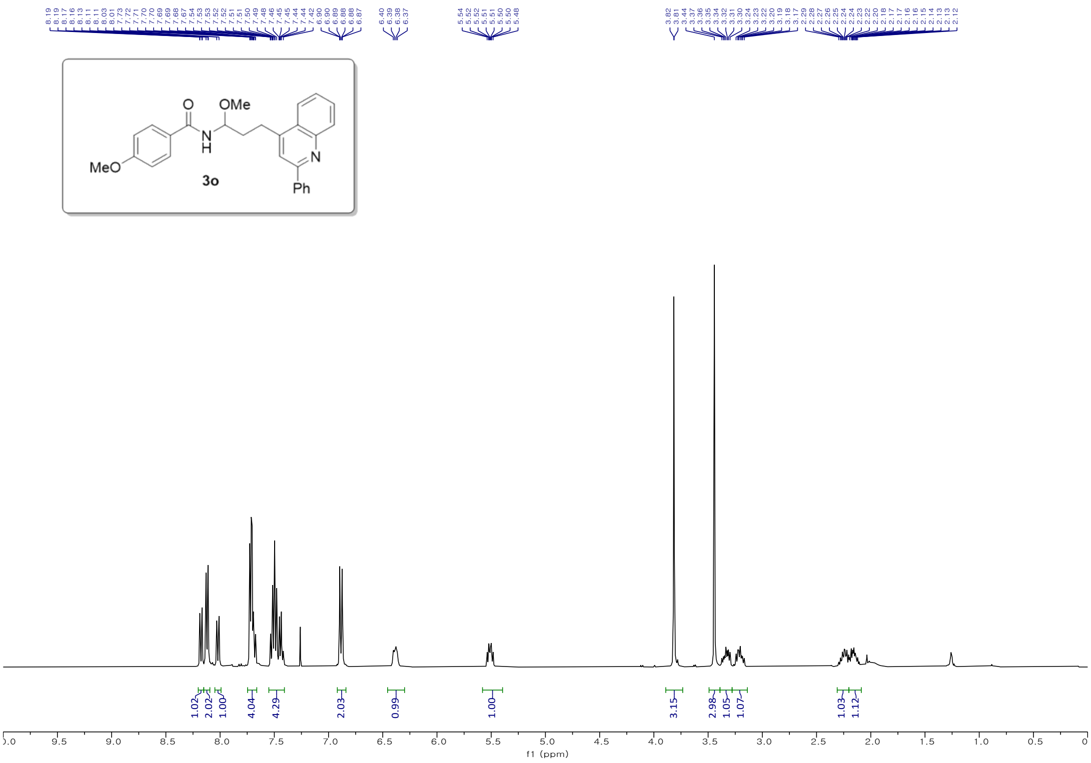

400 MHz, ^{1}H NMR in $CDCl_3$

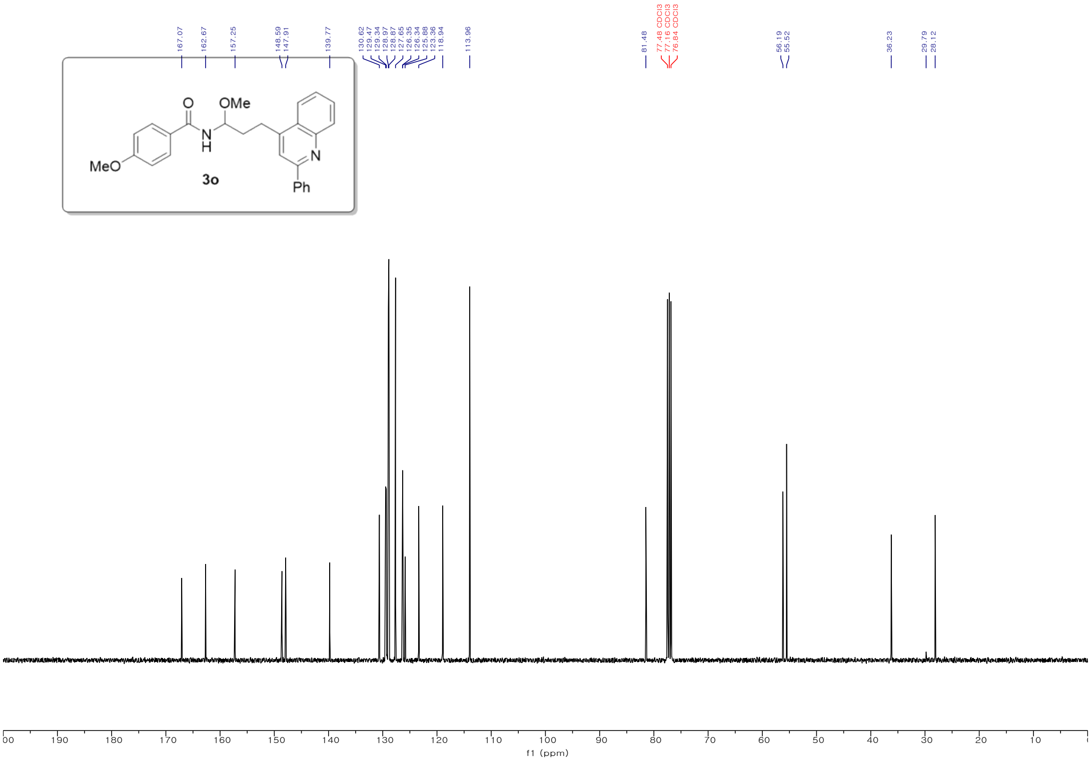

100 MHz, ^{13}C NMR in $CDCl_3$

4-methoxy-N-(1-methoxy-3-(6-methylquinolin-4-yl)propyl)benzamide (3p)

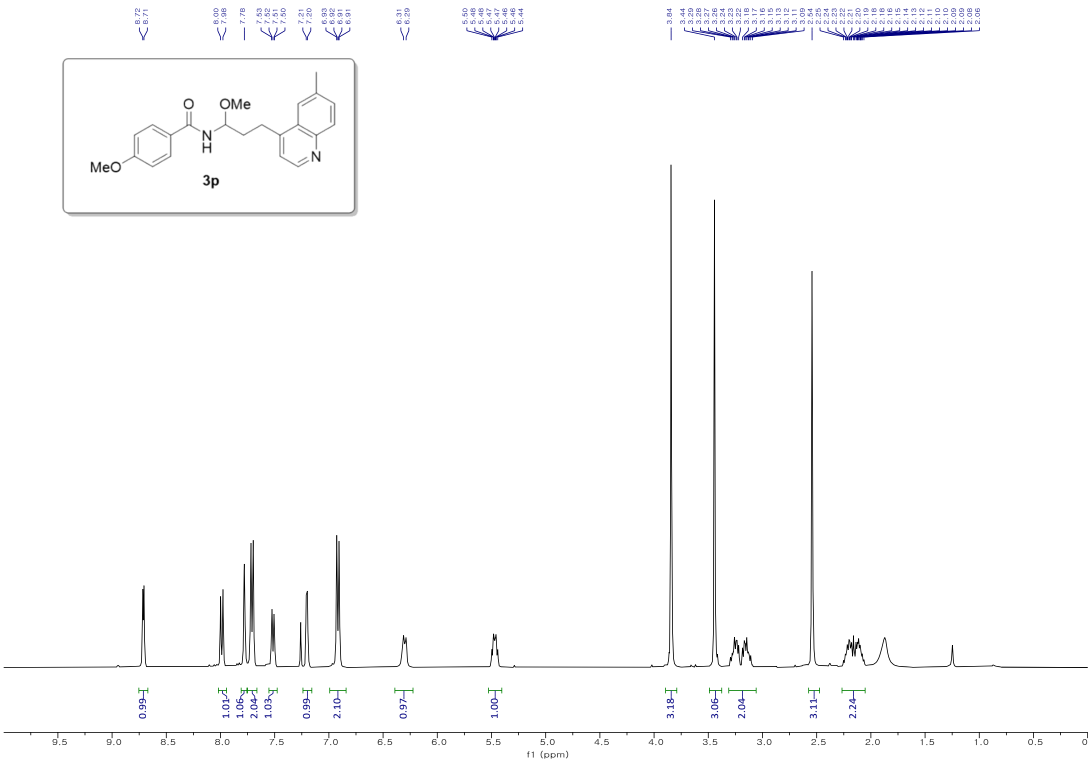

400 MHz, ^{1}H NMR in CDCl$_3$

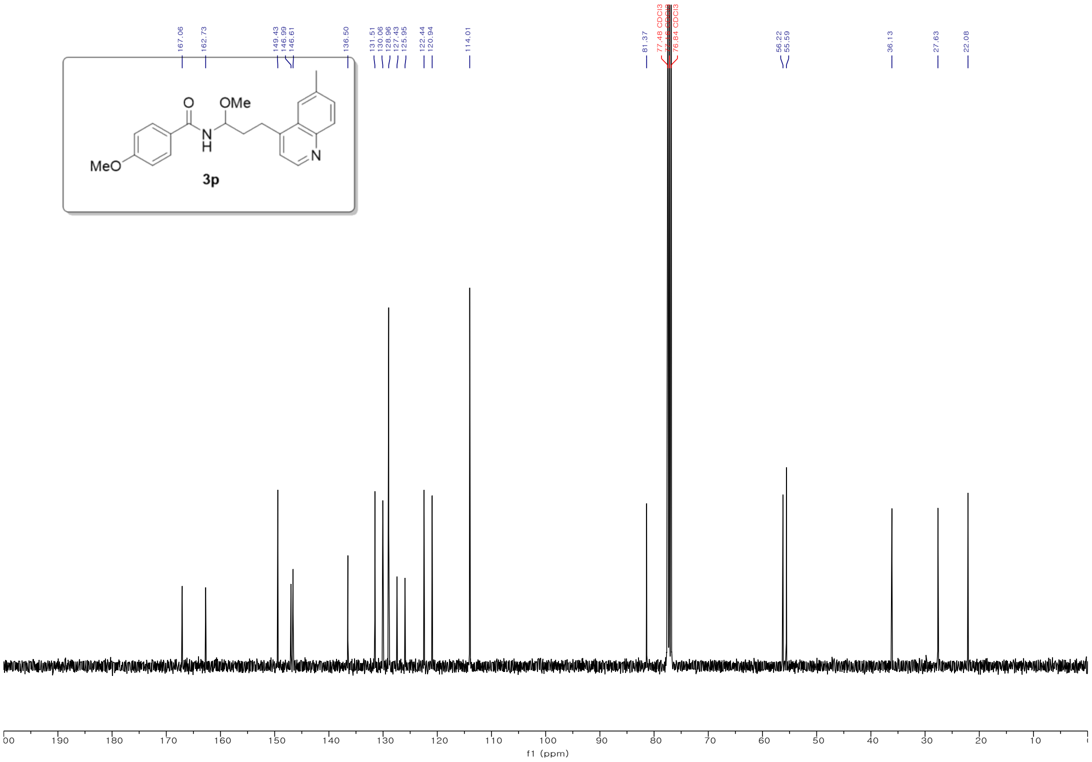

100 MHz, ^{13}C NMR in CDCl$_3$

2-chloro-N-(4-chloro-3-(4-(3-methoxy-3-(4-methoxybenzamido)propyl)pyridin-2-yl)phenyl)-4-(methylsulfonyl)benzamidebenzamide (3q).

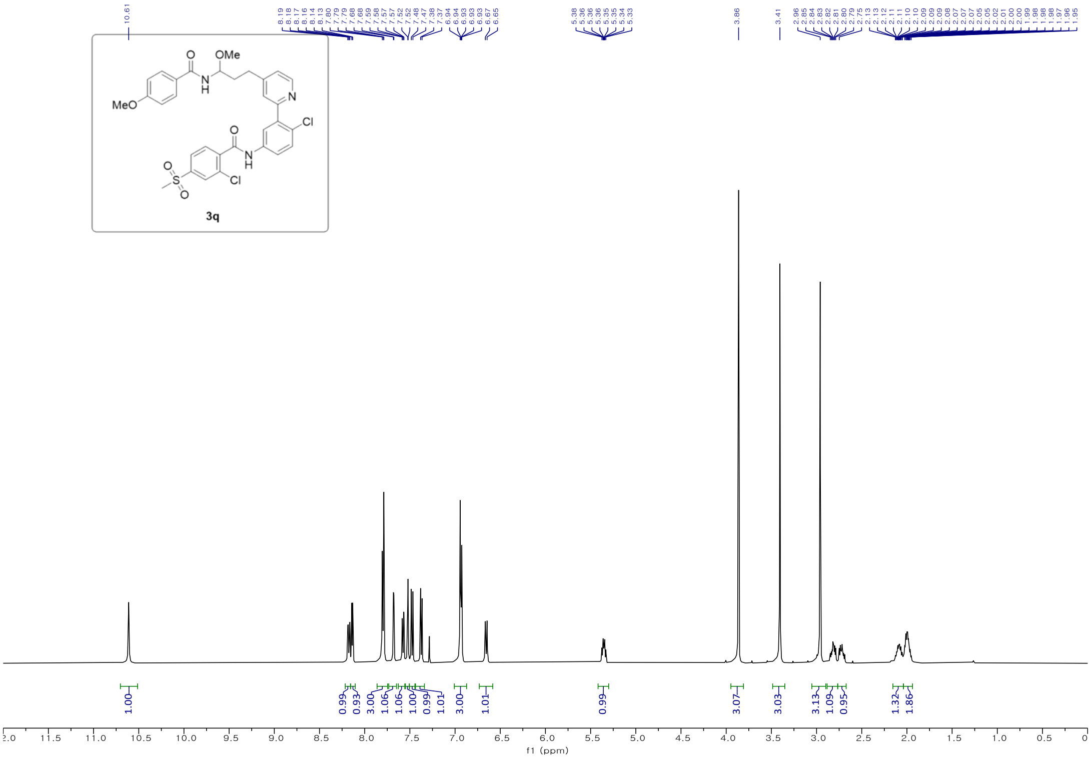

500 MHz, ^{1}H NMR in $CDCl_3$

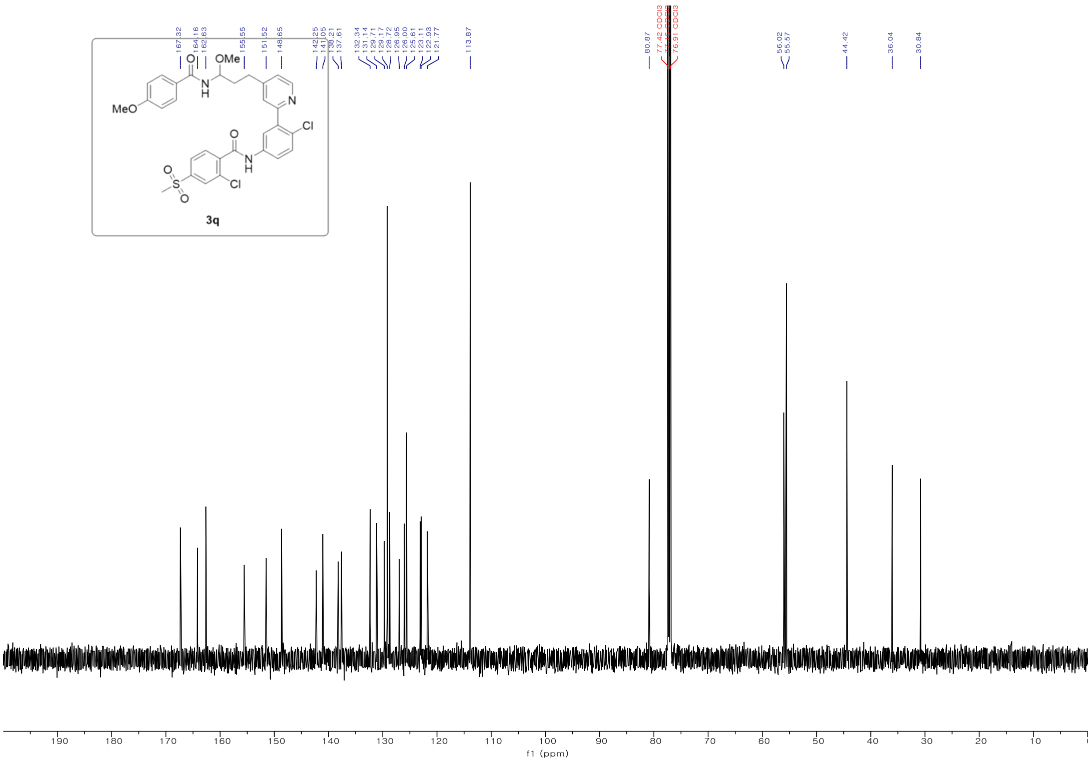

125 MHz, ^{13}C NMR in $CDCl_3$

((4-(3-methoxy-3-(4-methoxybenzamido)propyl)pyridin-2-yl)methylene)bis(4,1-phenylene) diacetate (3r).

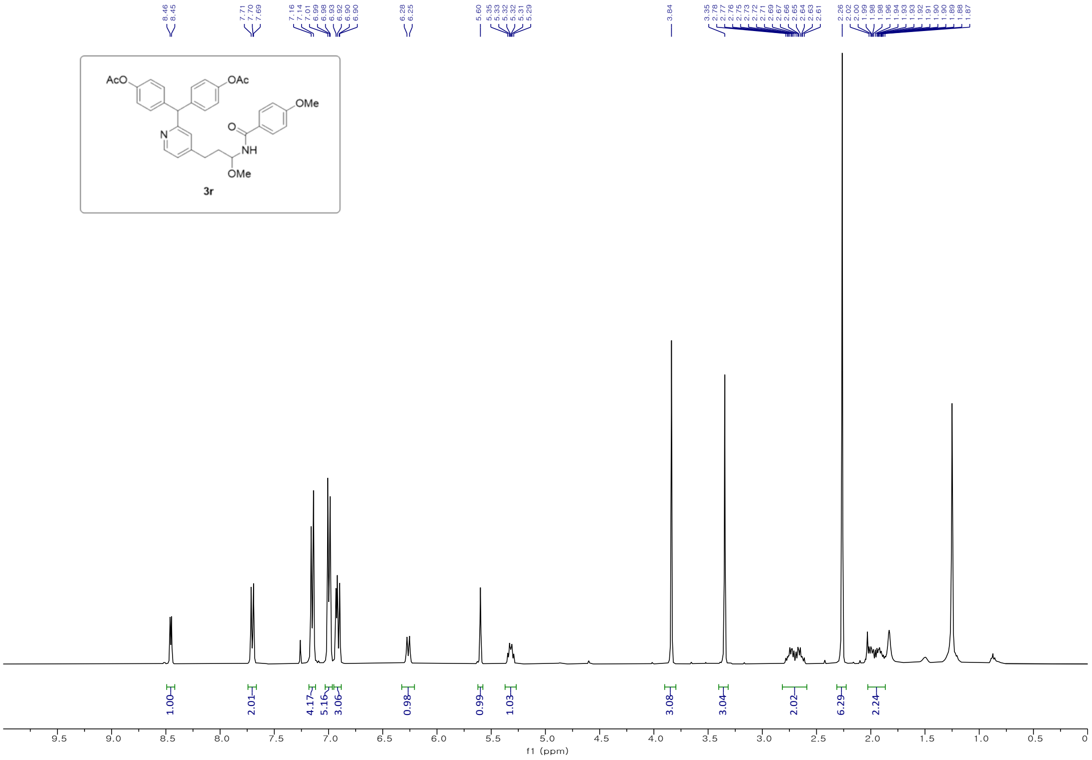

400 MHz, ^{1}H NMR in $CDCl_3$

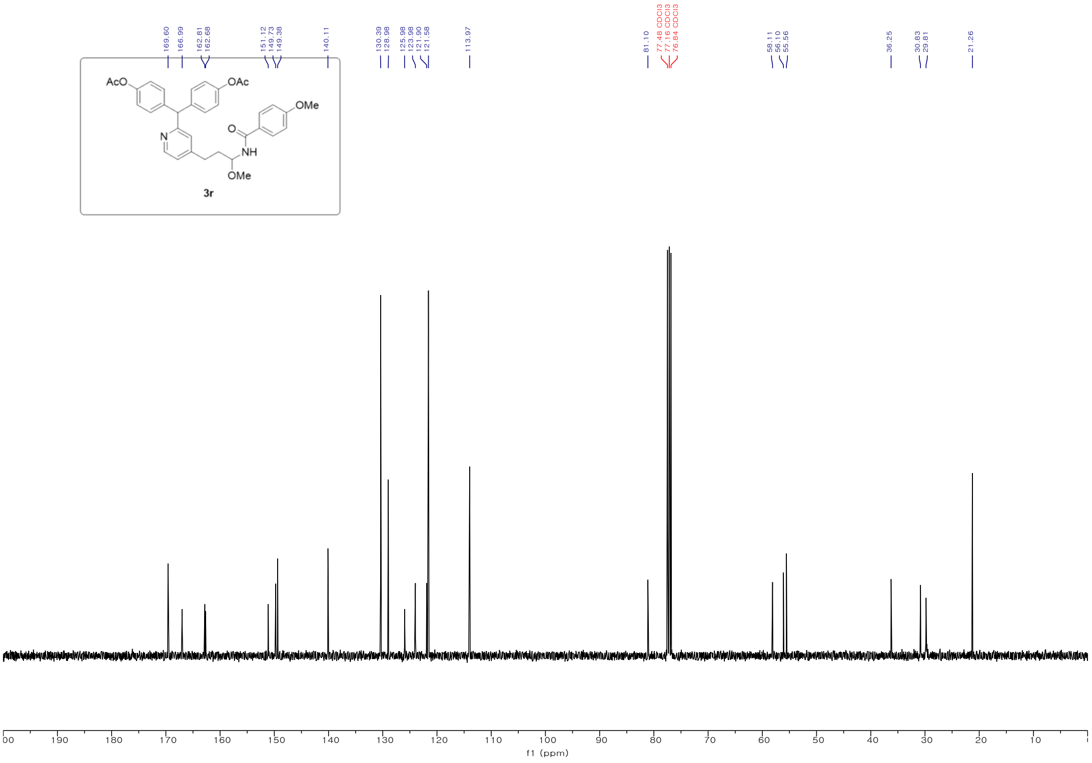

100 MHz, ^{13}C NMR in $CDCl_3$

(4-(3-methoxy-3-(4-methoxybenzamido)propyl)pyridin-2-yl)methyl 2-(4-chlorophenyl)-2-methylpropanoate (3s).

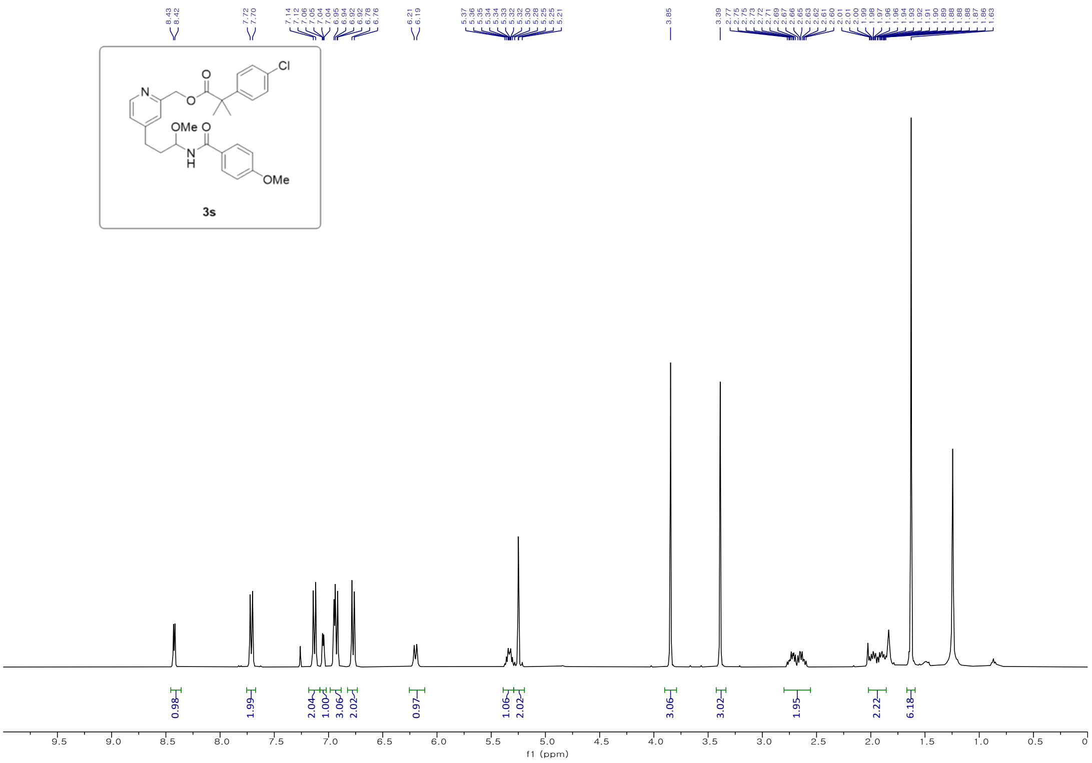

400 MHz, ^{1}H NMR in $CDCl_3$

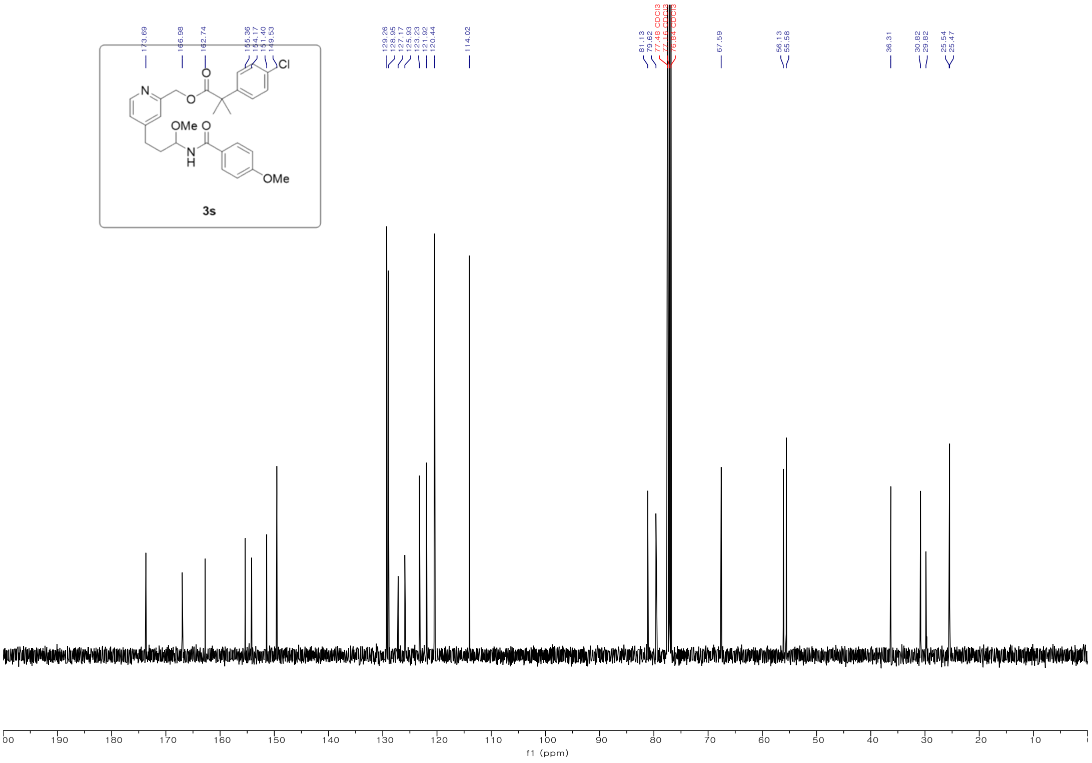

100 MHz, ^{13}C NMR in $CDCl_3$

(4-(3-methoxy-3-(4-methoxybenzamido)propyl)pyridin-2-yl)methyl 2-propylpentanoate (3t).

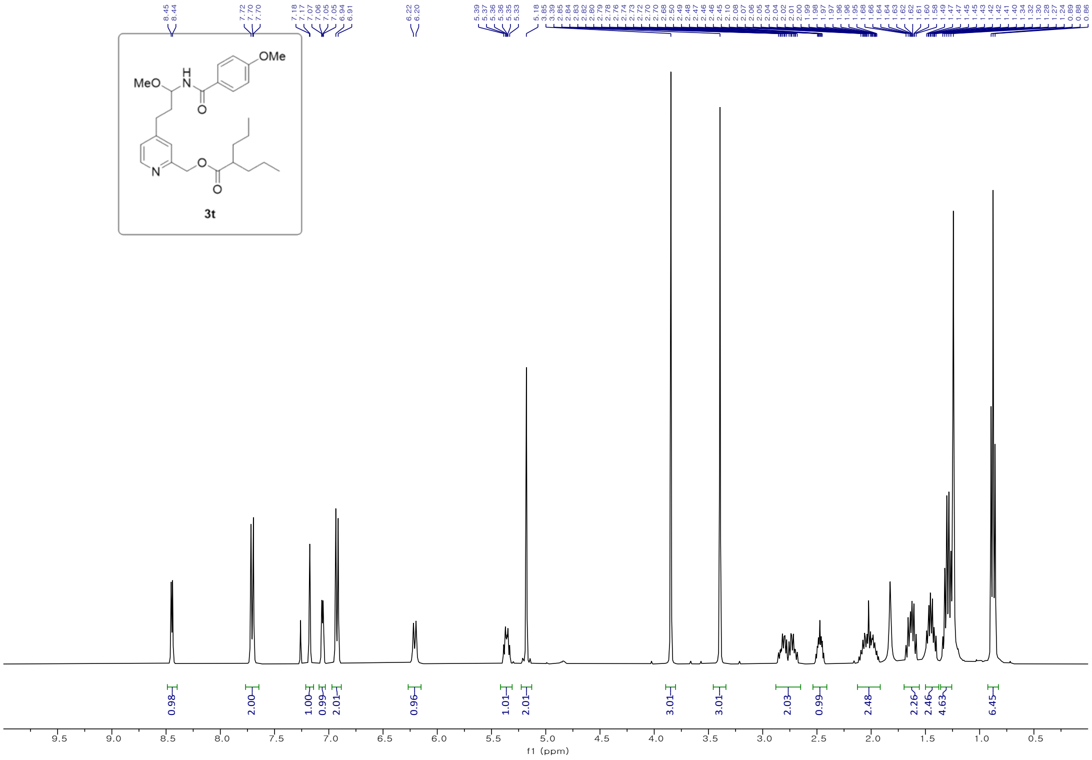

400 MHz, ^{1}H NMR in $CDCl_3$

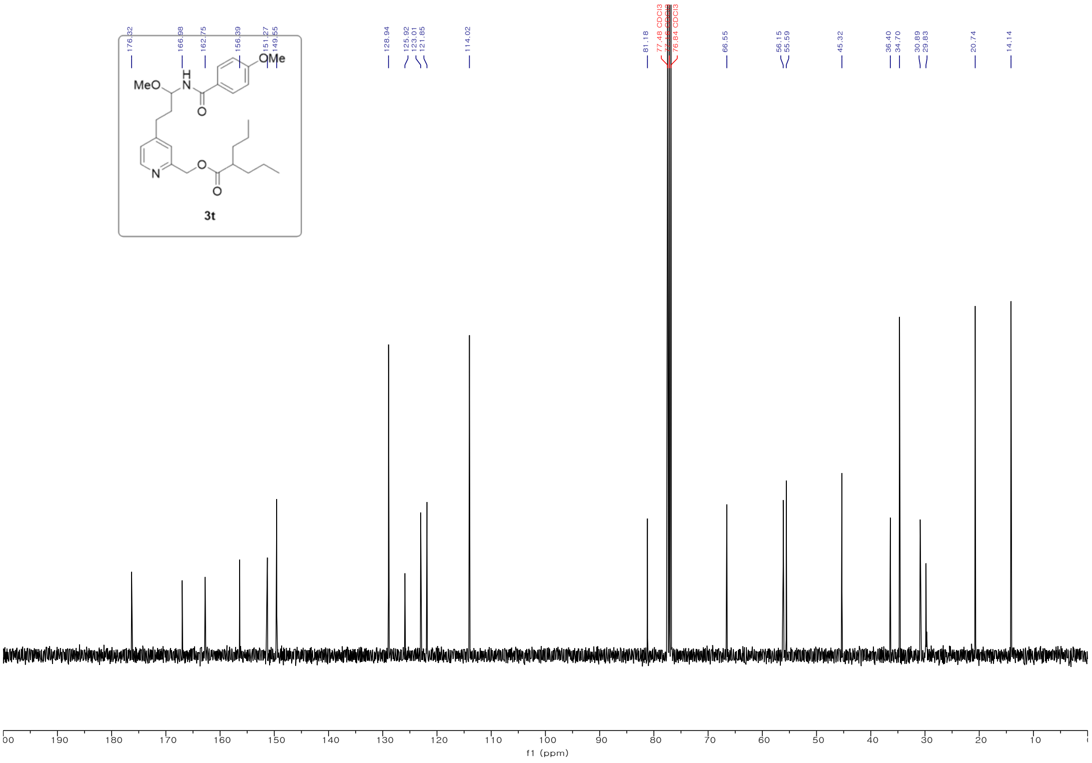

100 MHz, ^{13}C NMR in $CDCl_3$

N-(1-methoxy-3-(2-phenylpyridin-4-yl)propyl)benzamide (4a)

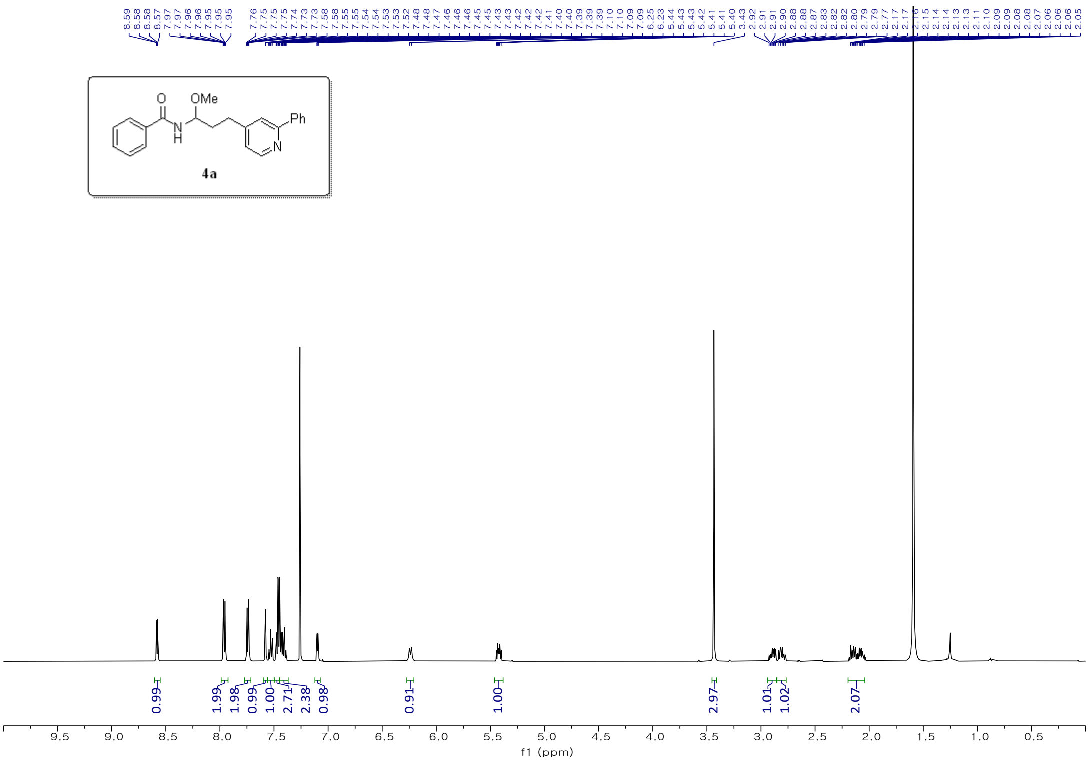

500 MHz, ^{1}H NMR in $CDCl_3$

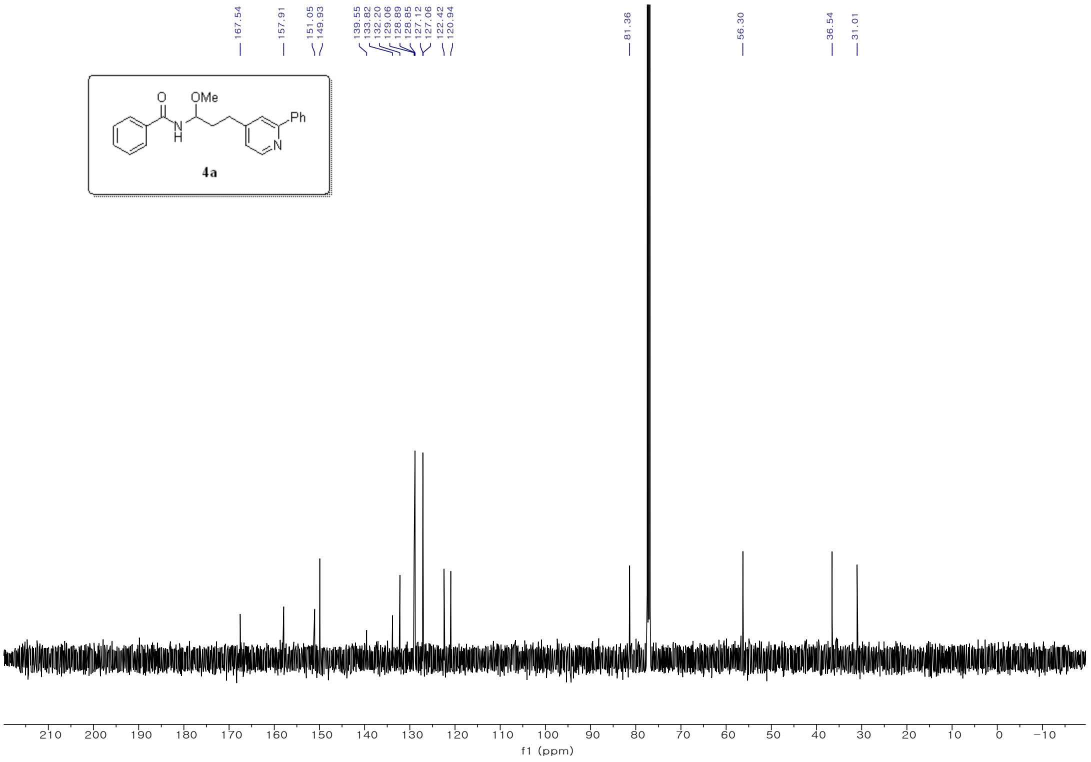

125 MHz, ^{13}C NMR in $CDCl_3$

N-(1-methoxy-3-(2-phenylpyridin-4-yl)propyl)-[1,1'-biphenyl]-4-carboxamide (4b)

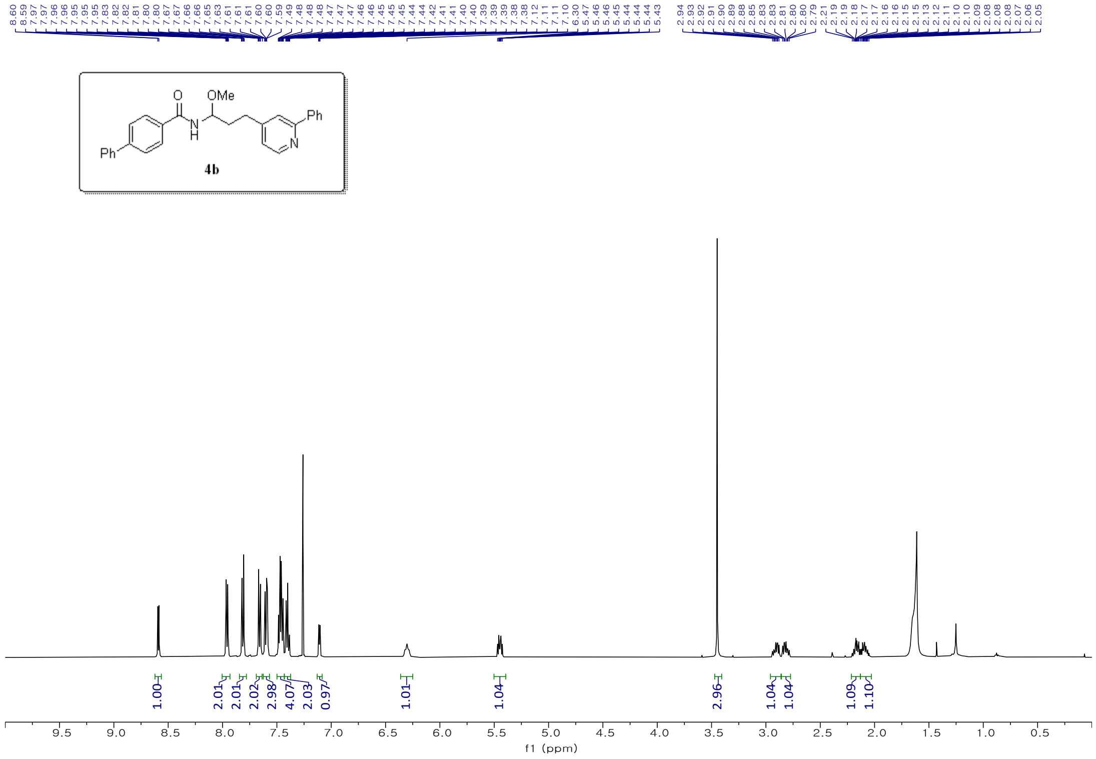

500 MHz, ^{1}H NMR in $CDCl_3$

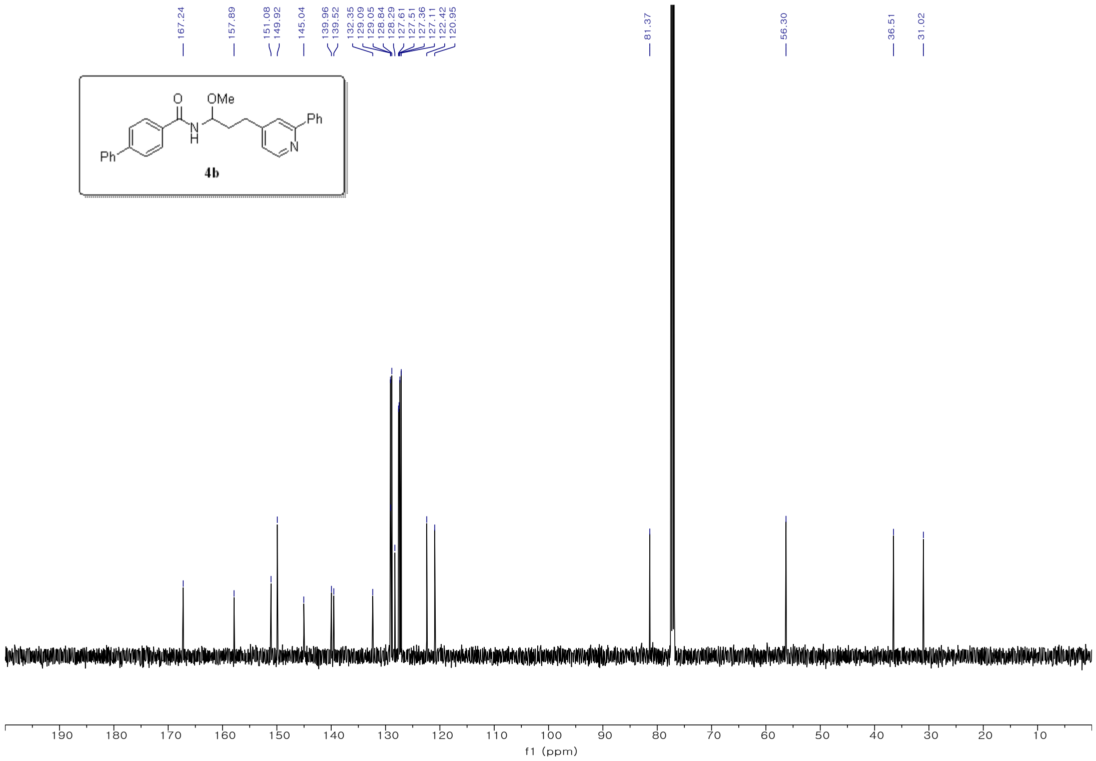

125 MHz, ^{13}C NMR in $CDCl_3$

4-chloro-N-(1-methoxy-3-(2-phenylpyridin-4-yl)propyl)benzamide (4c)

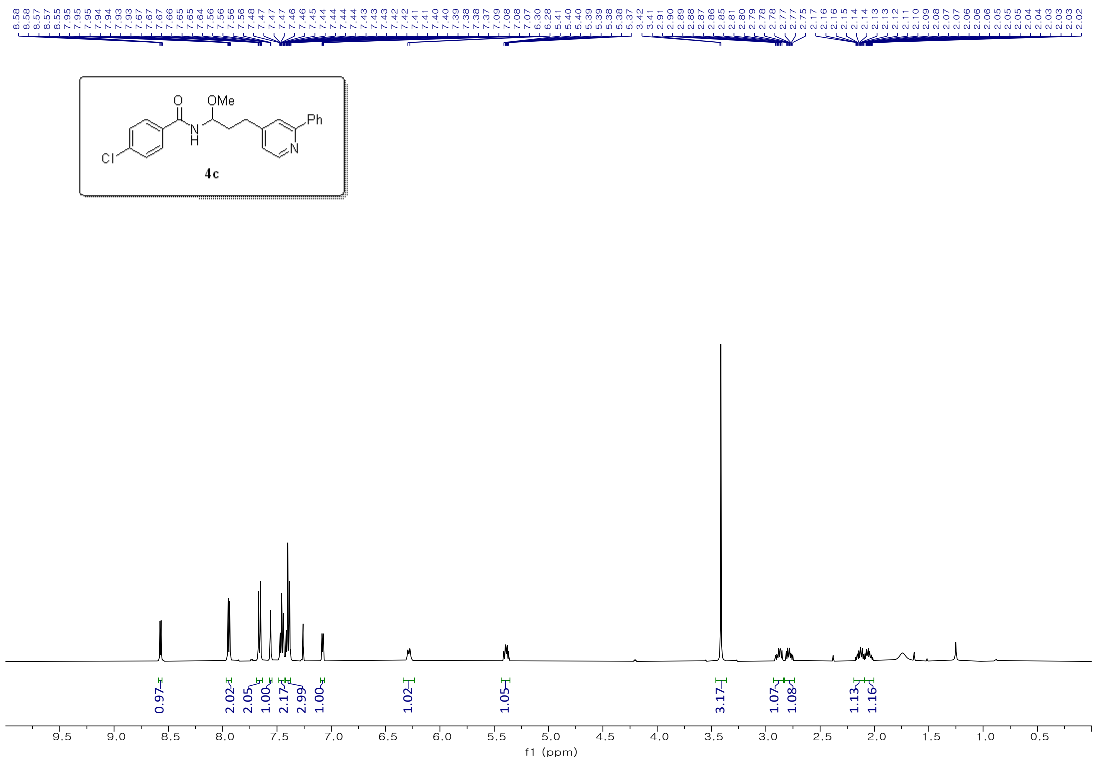

500 MHz, ^{1}H NMR in $CDCl_3$

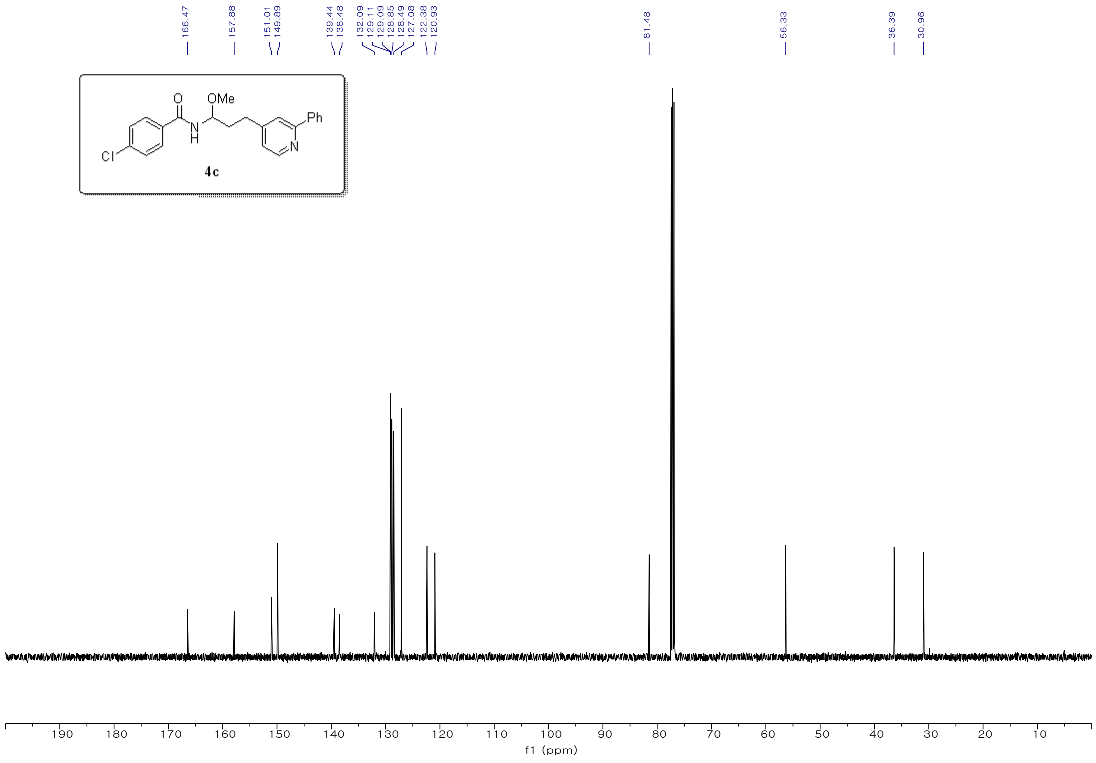

125 MHz, ^{13}C NMR in $CDCl_3$

N-(1-methoxy-3-(2-phenylpyridin-4-yl)propyl)-4-(trifluoromethyl)benzamide (4d)

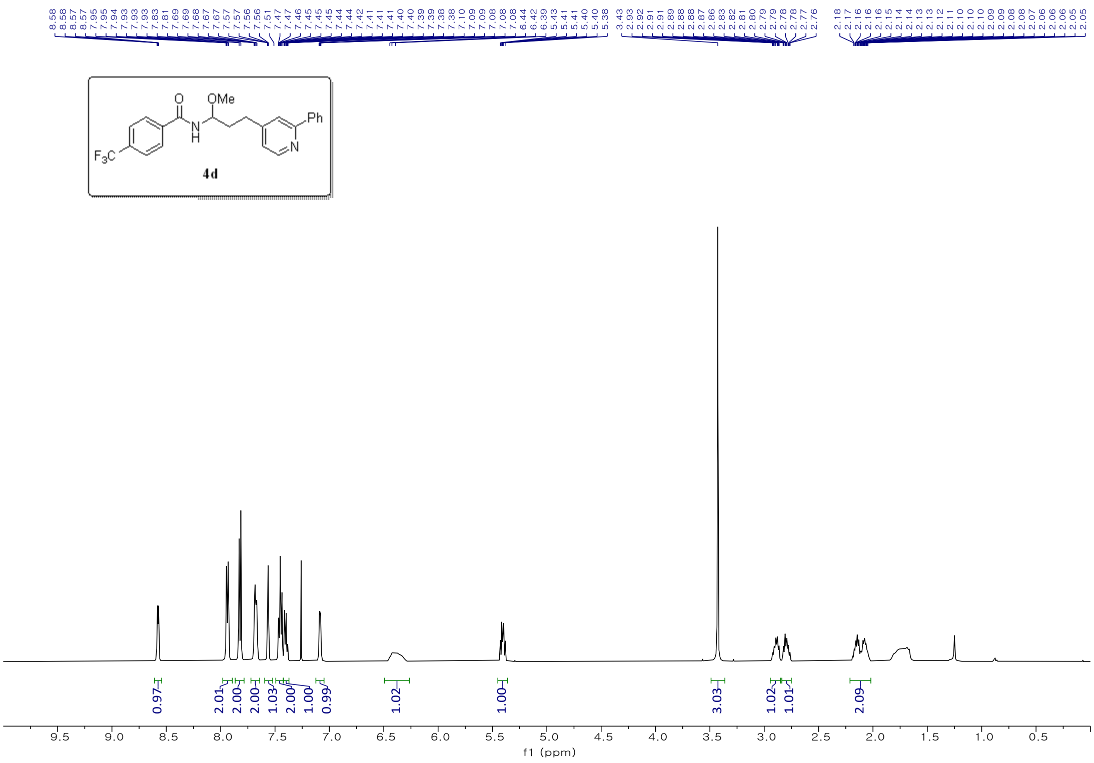

500 MHz, ^{1}H NMR in $CDCl_3$

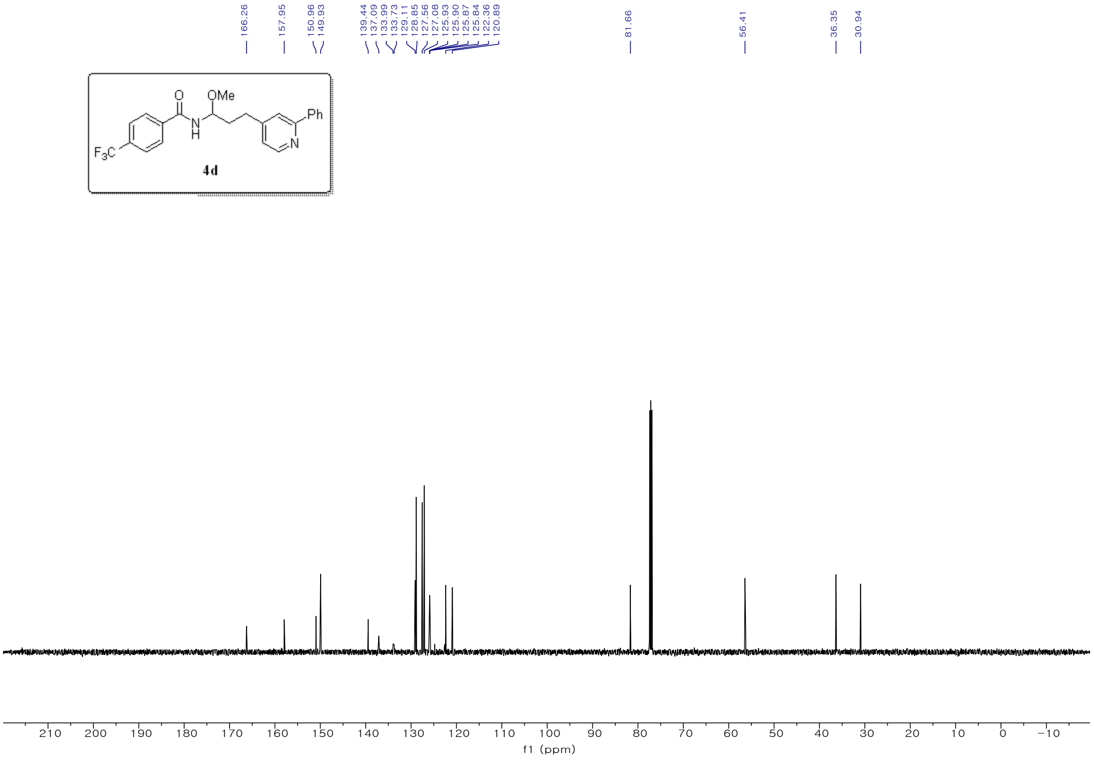

125 MHz, ^{13}C NMR in $CDCl_3$

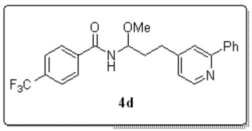

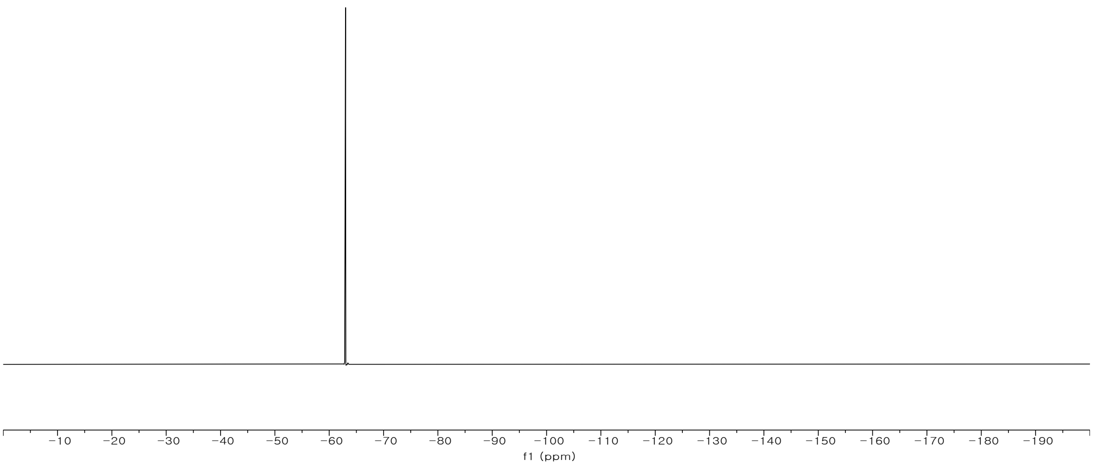

471 MHz, ^{19}F NMR in $CDCl_3$

methyl 4-((1-methoxy-3-(2-phenylpyridin-4-yl)propyl)carbamoyl)benzoate (4e)

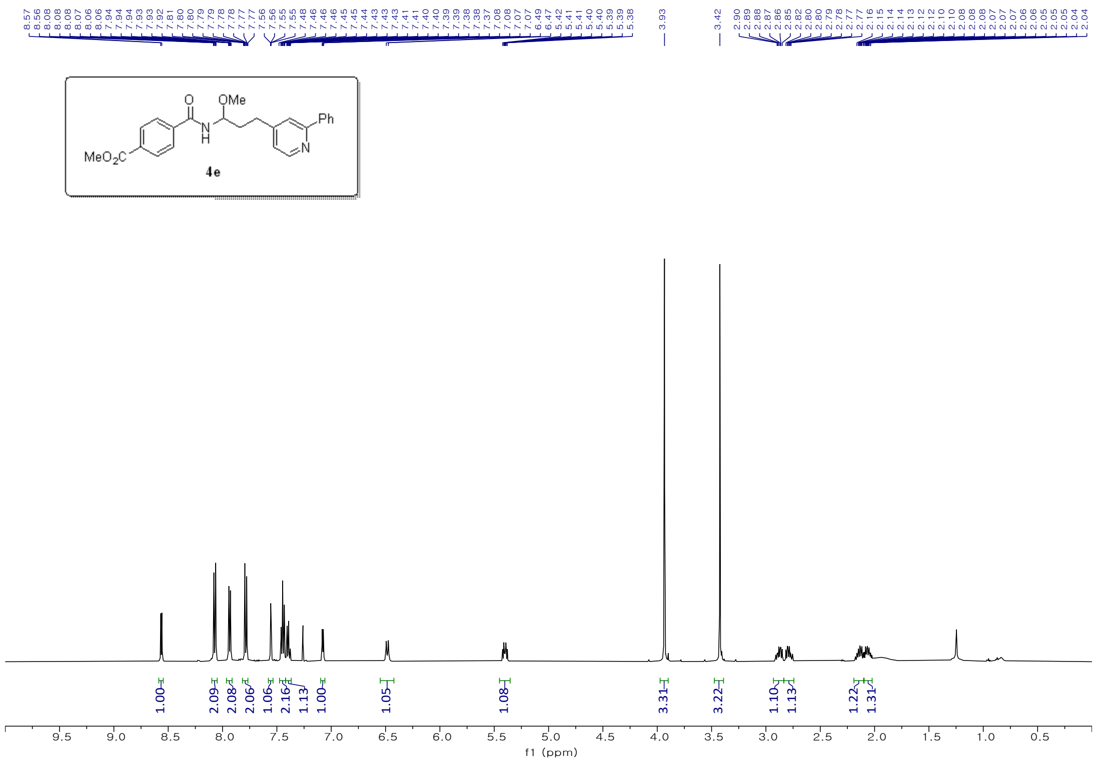

500 MHz, ^{1}H NMR in $CDCl_3$

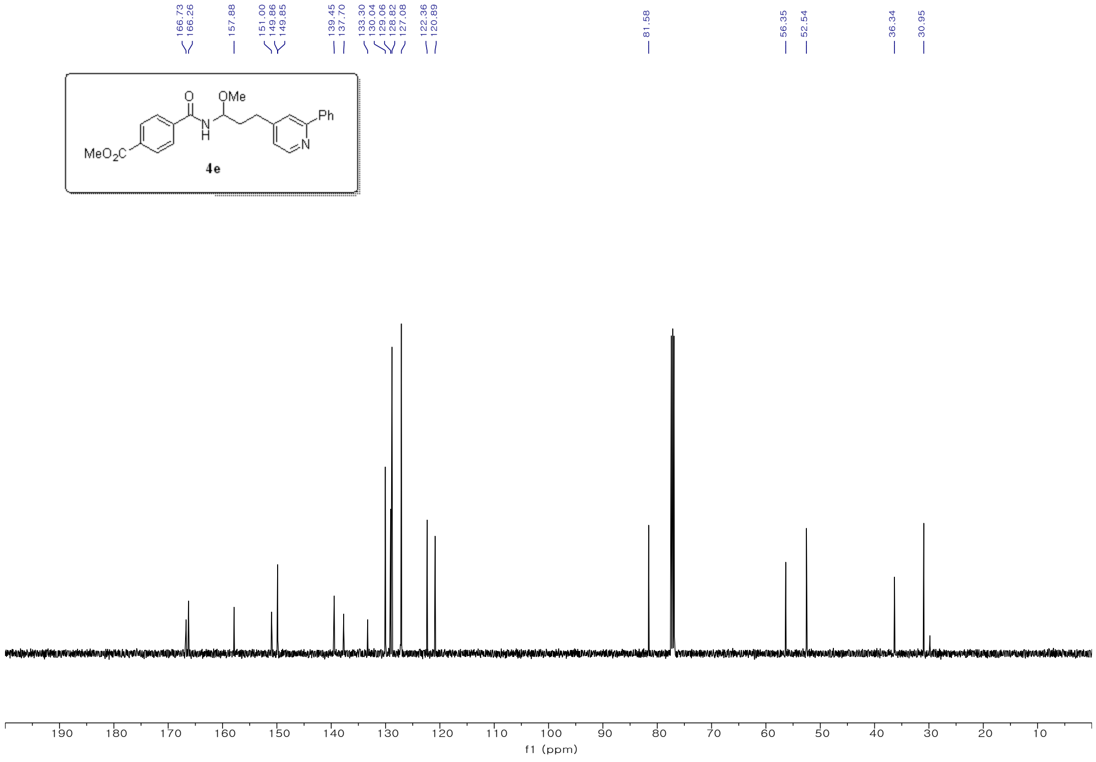

125 MHz, ^{13}C NMR in $CDCl_3$

4-cyano-N-(1-methoxy-3-(2-phenylpyridin-4-yl)propyl)benzamide (4f)

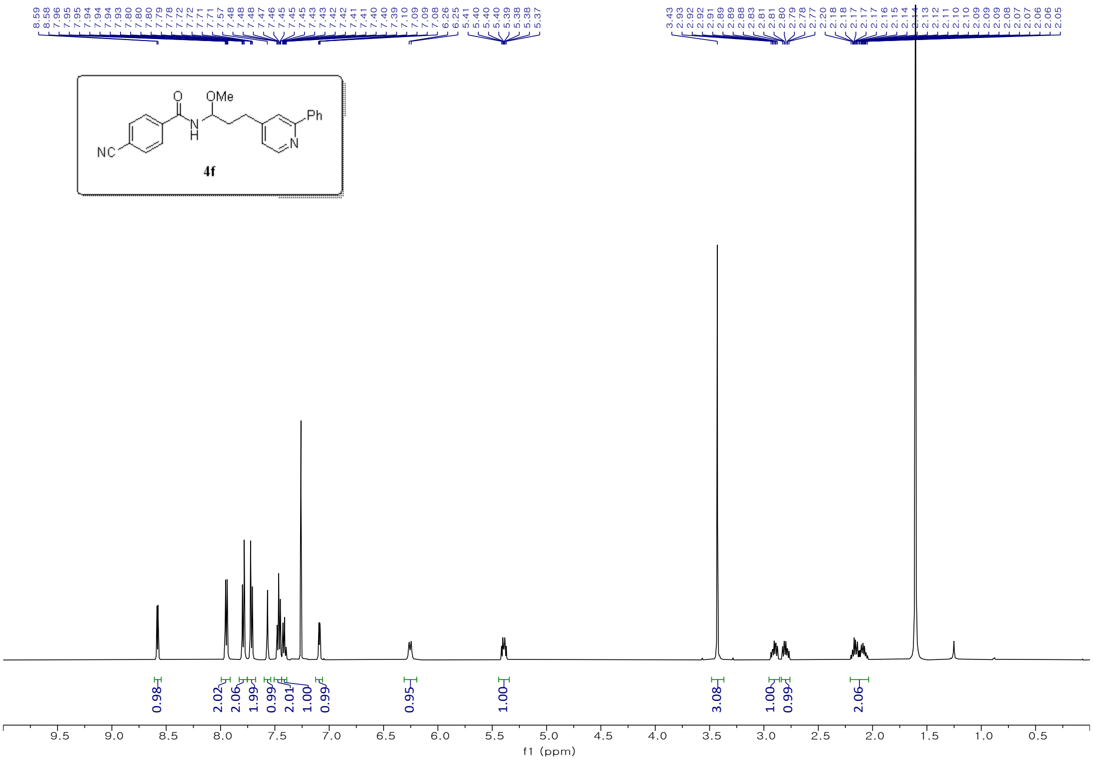

500 MHz, ^{1}H NMR in CDCl$_3$

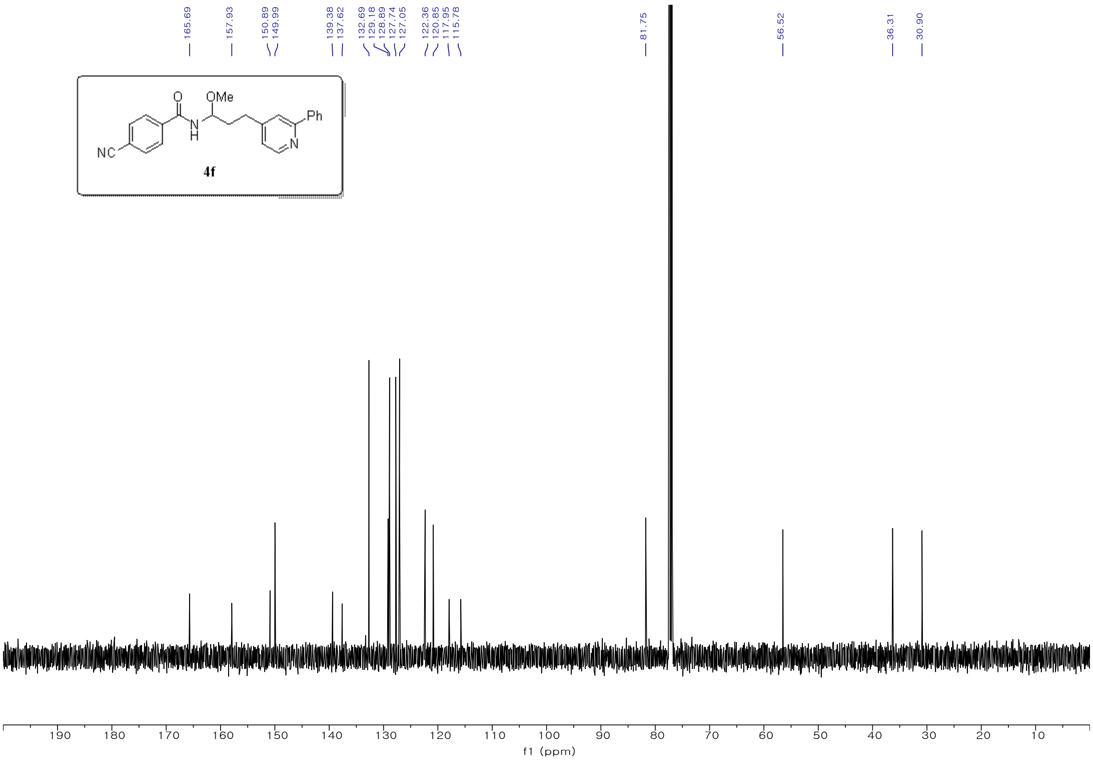

125 MHz, ^{13}C NMR in CDCl$_3$

3-methoxy-N-(1-methoxy-3-(2-phenylpyridin-4-yl)propyl)benzamide (4g)

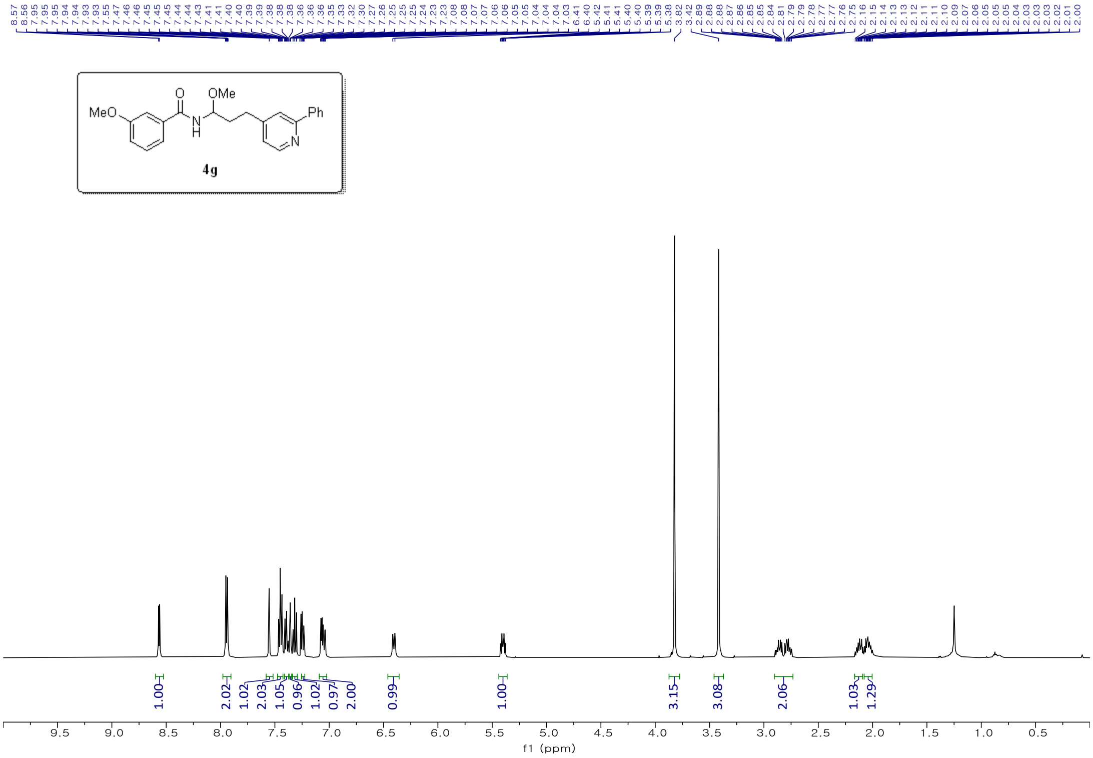

500 MHz, ^{1}H NMR in $CDCl_3$

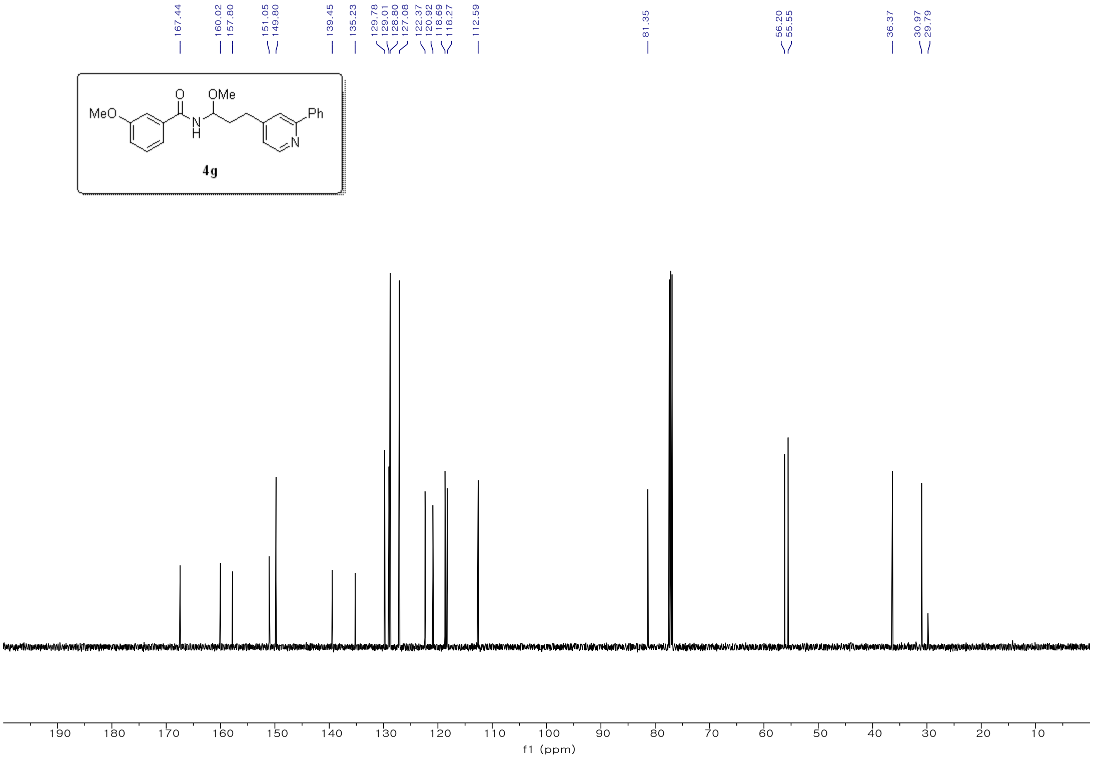

125 MHz, ^{13}C NMR in $CDCl_3$

3-bromo-N-(1-methoxy-3-(2-phenylpyridin-4-yl)propyl)benzamide (4h)

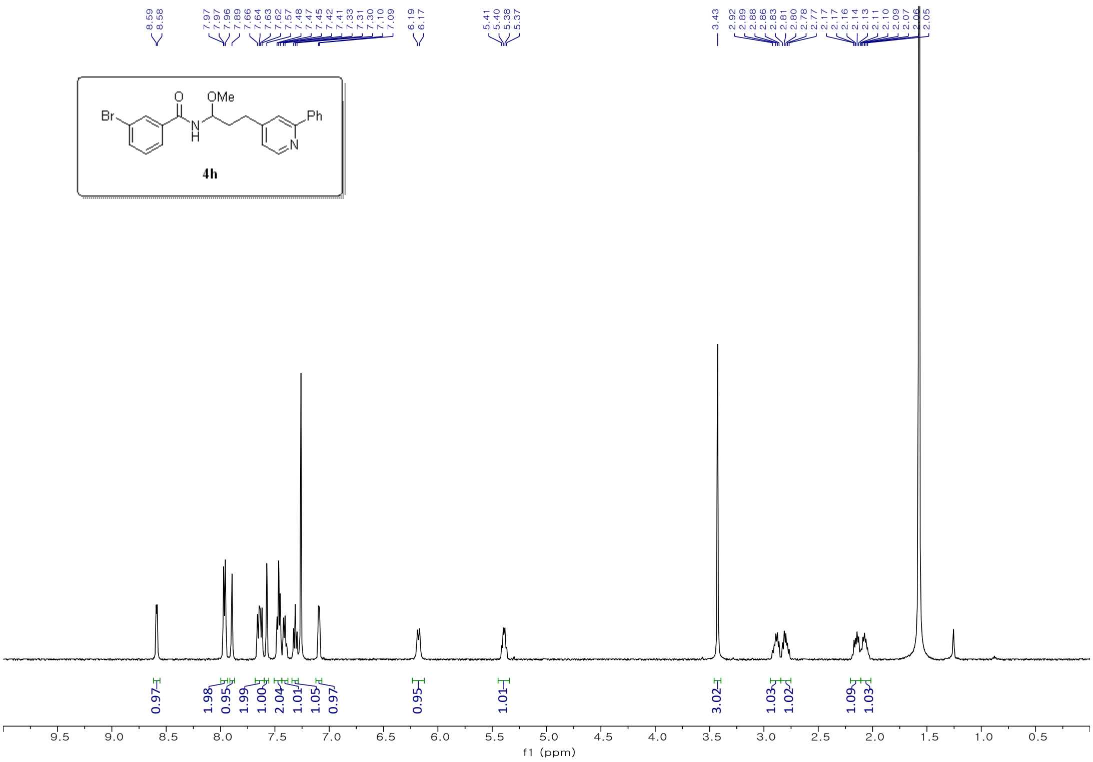

500 MHz, ^{1}H NMR in $CDCl_3$

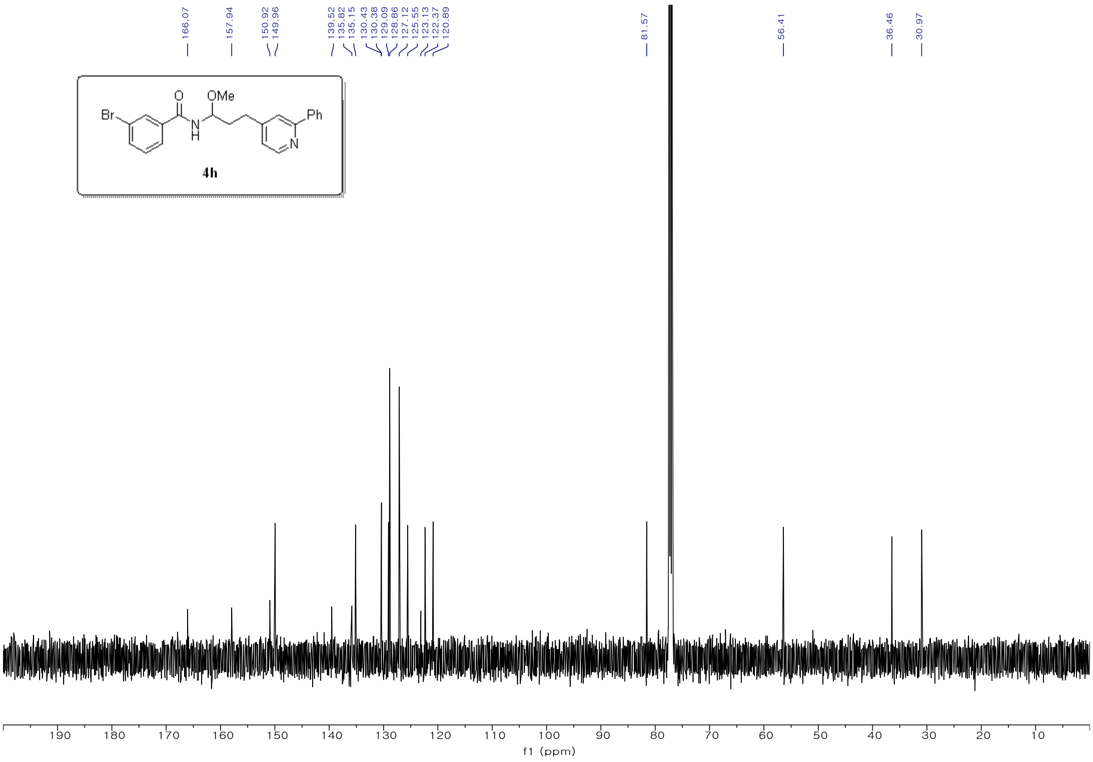

125 MHz, ^{13}C NMR in $CDCl_3$

2-methoxy-N-(1-methoxy-3-(2-phenylpyridin-4-yl)propyl)benzamide (4i)

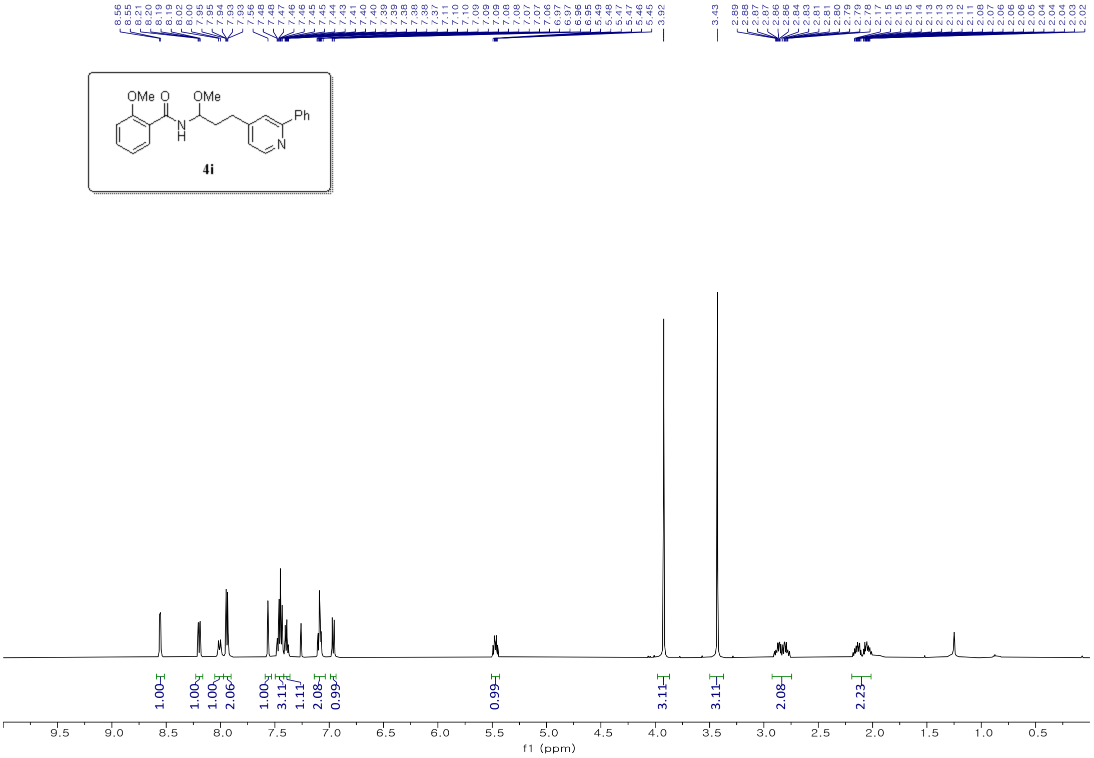

500 MHz, ^{1}H NMR in CDCl$_3$

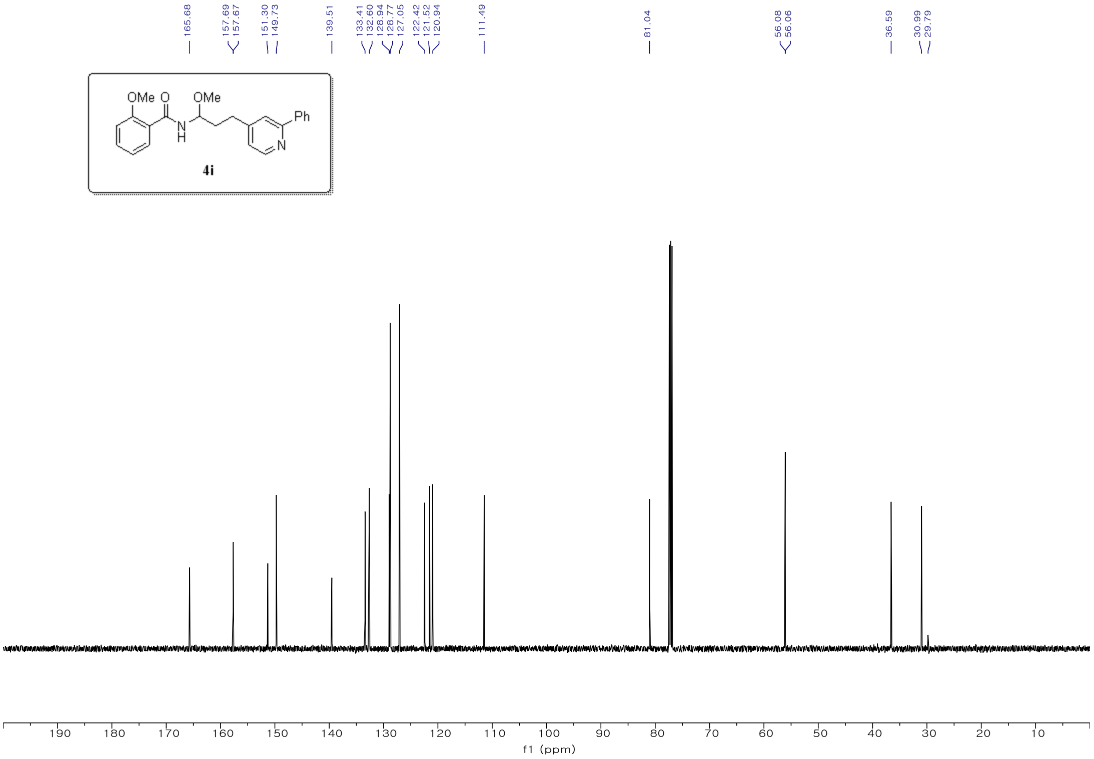

125 MHz, ^{13}C NMR in CDCl$_3$

2-bromo-N-(1-methoxy-3-(2-phenylpyridin-4-yl)propyl)benzamide (4j)

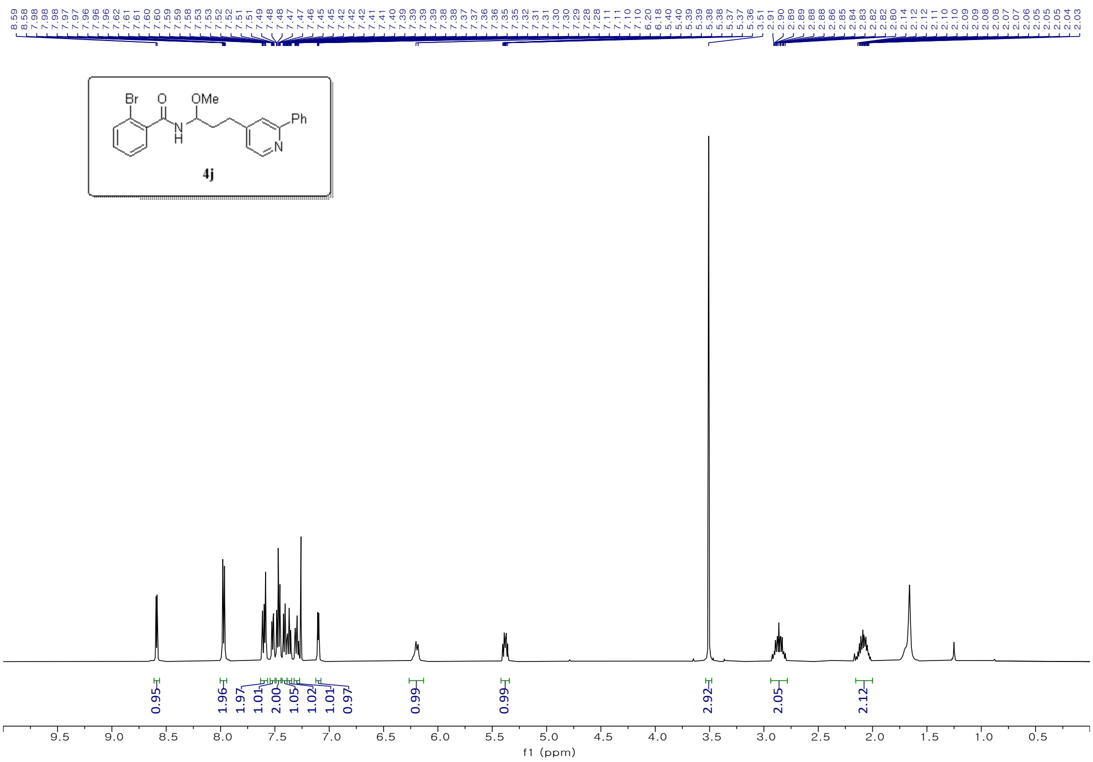

500 MHz, ¹H NMR in CDCl₃

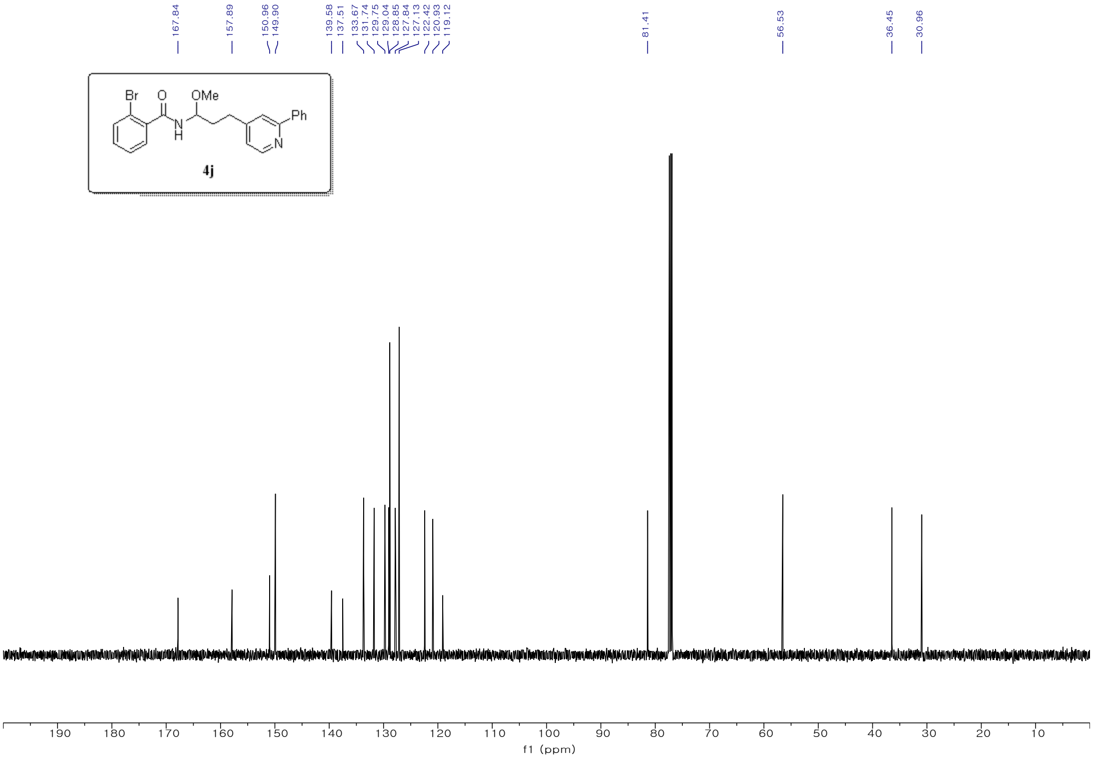

125 MHz, ¹³C NMR in CDCl₃

N-(1-methoxy-3-(2-phenylpyridin-4-yl)propyl)-2-naphthamide (4k)

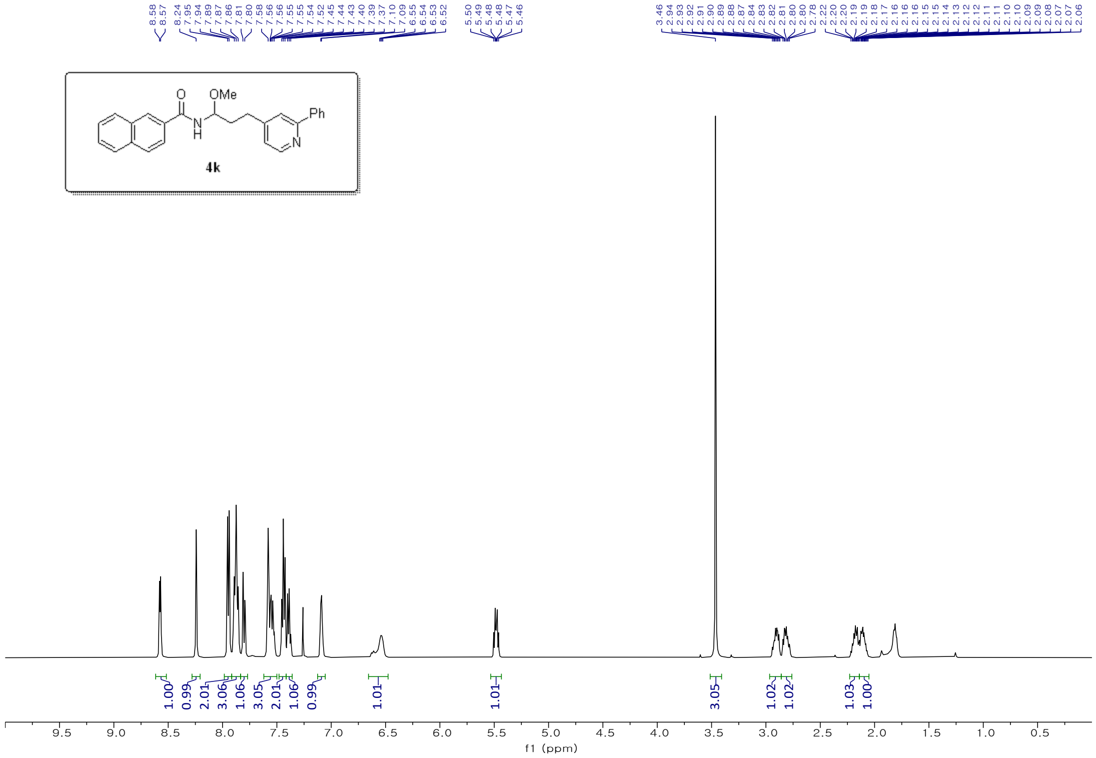

500 MHz, ^{1}H NMR in CDCl$_3$

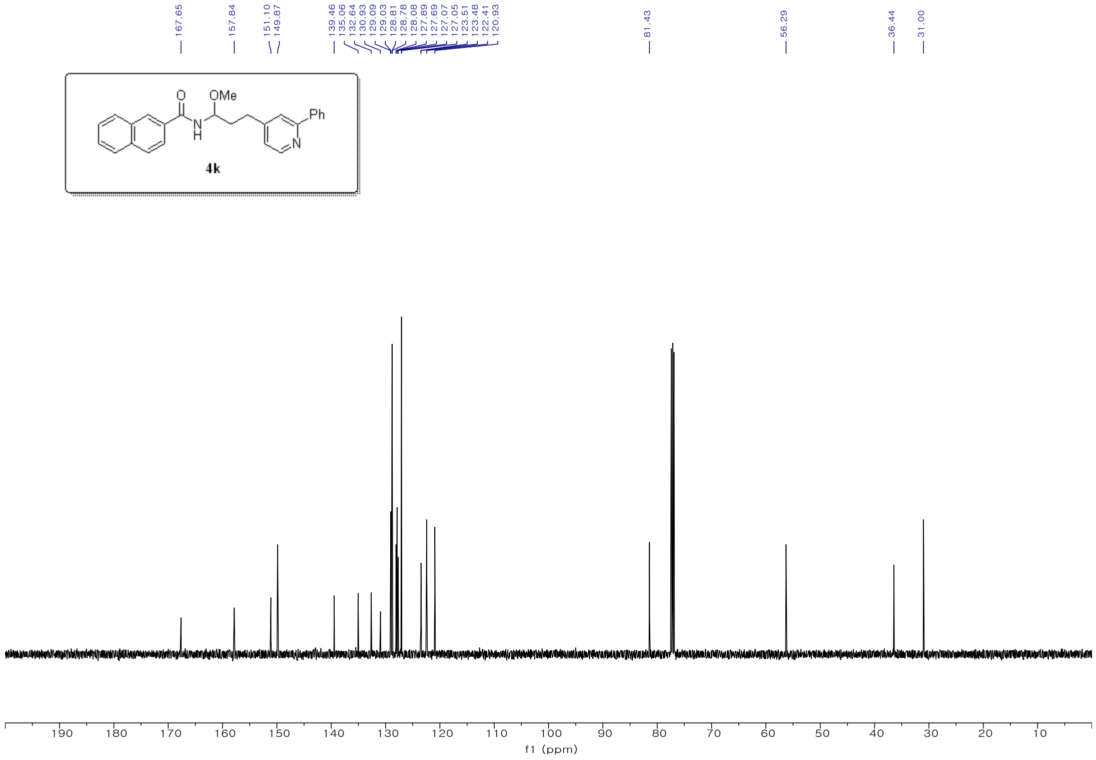

125 MHz, ^{13}C NMR in CDCl$_3$

N-(1-methoxy-3-(2-phenylpyridin-4-yl)propyl)thiophene-2-carboxamide (4l)

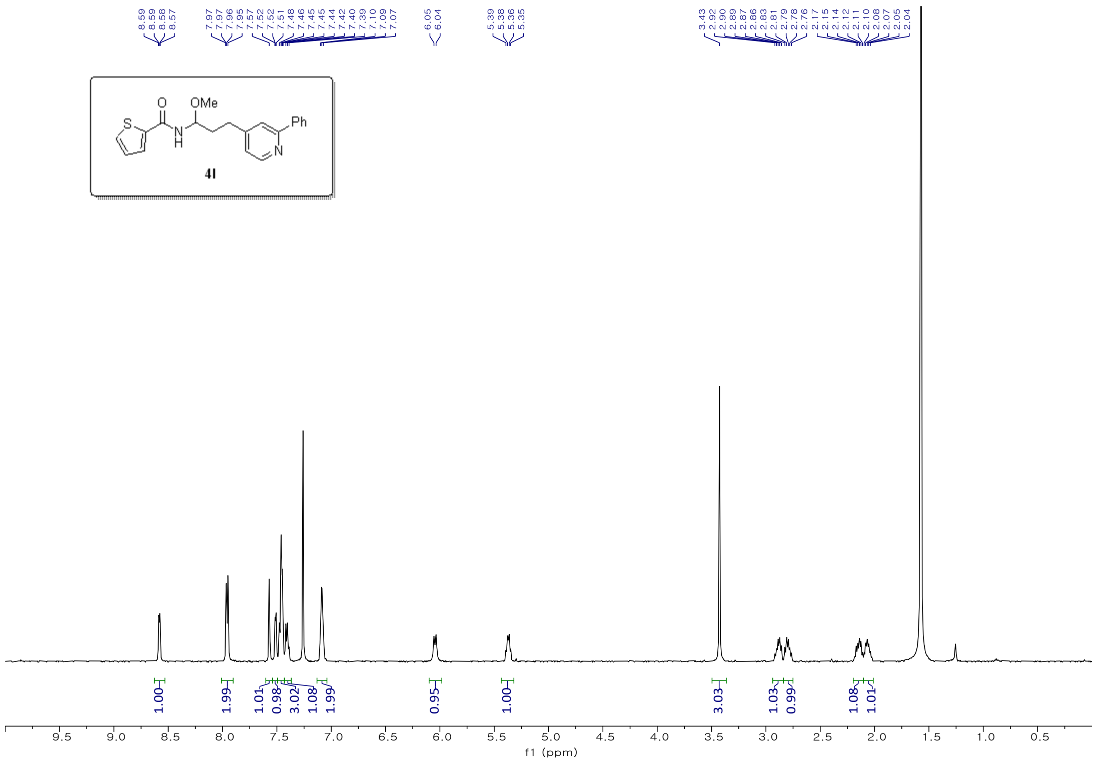

500 MHz, ^{1}H NMR in $CDCl_3$

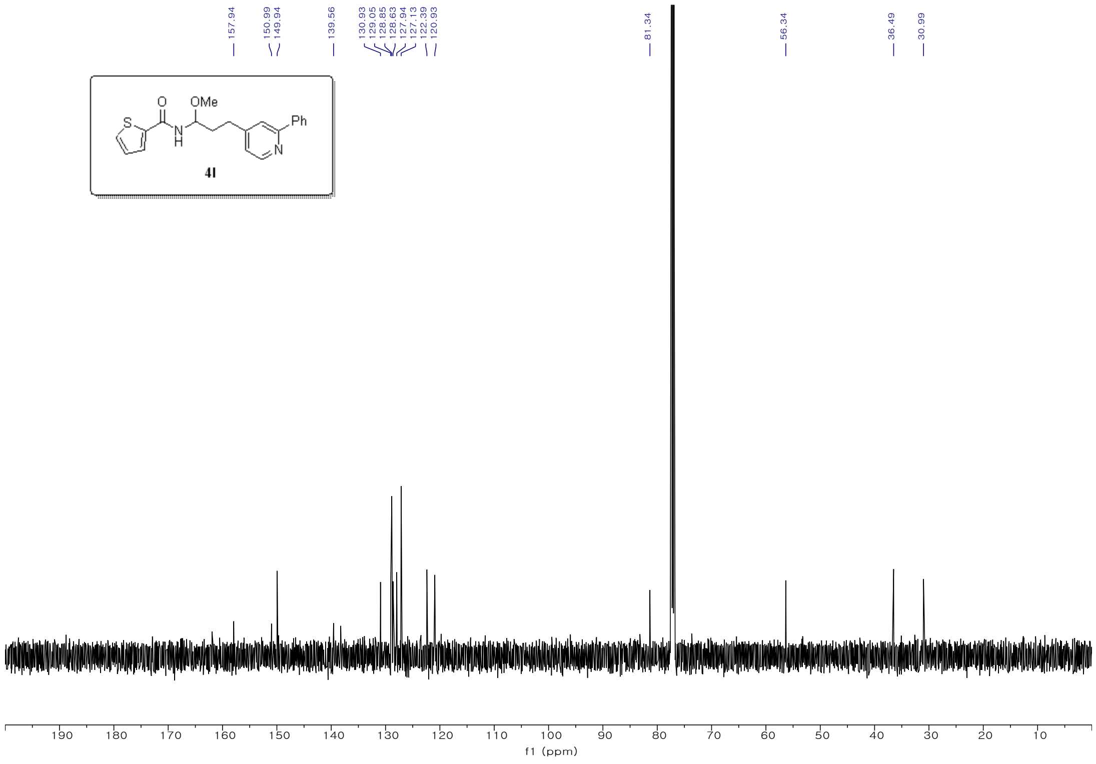

125 MHz, ^{13}C NMR in $CDCl_3$

4-methoxy-N-(1-methoxy-4-(2-phenylpyridin-4-yl)butyl)benzamide (4m).

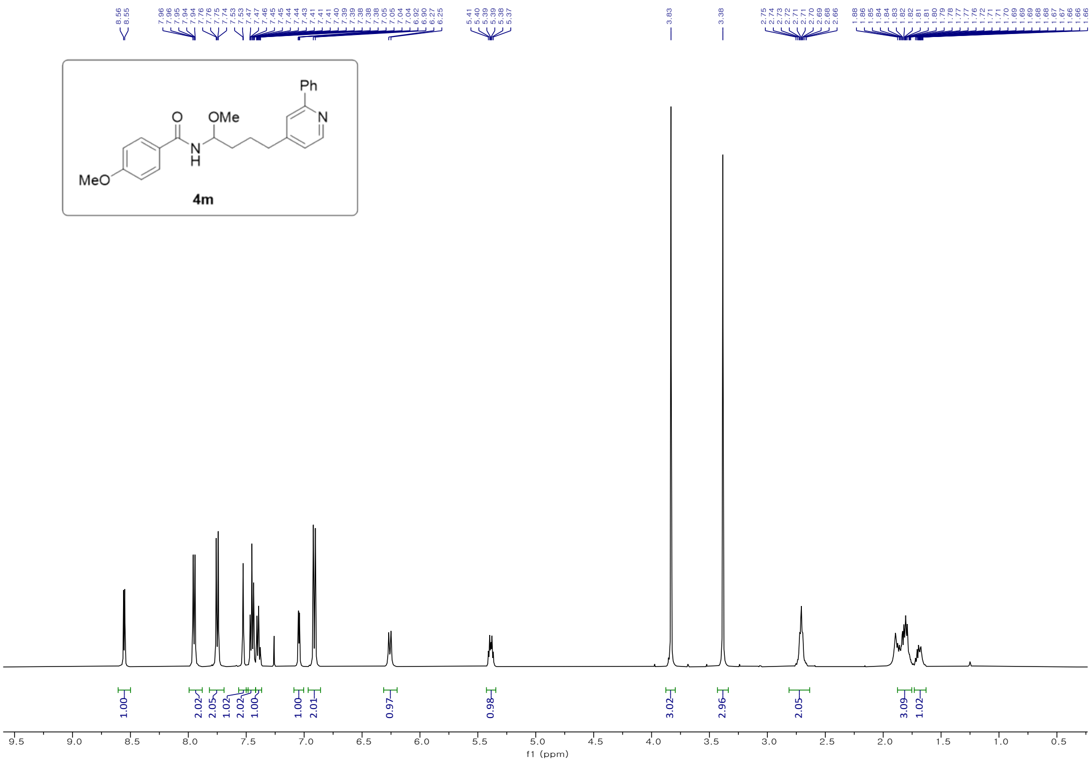

500 MHz, ^{1}H NMR in $CDCl_3$

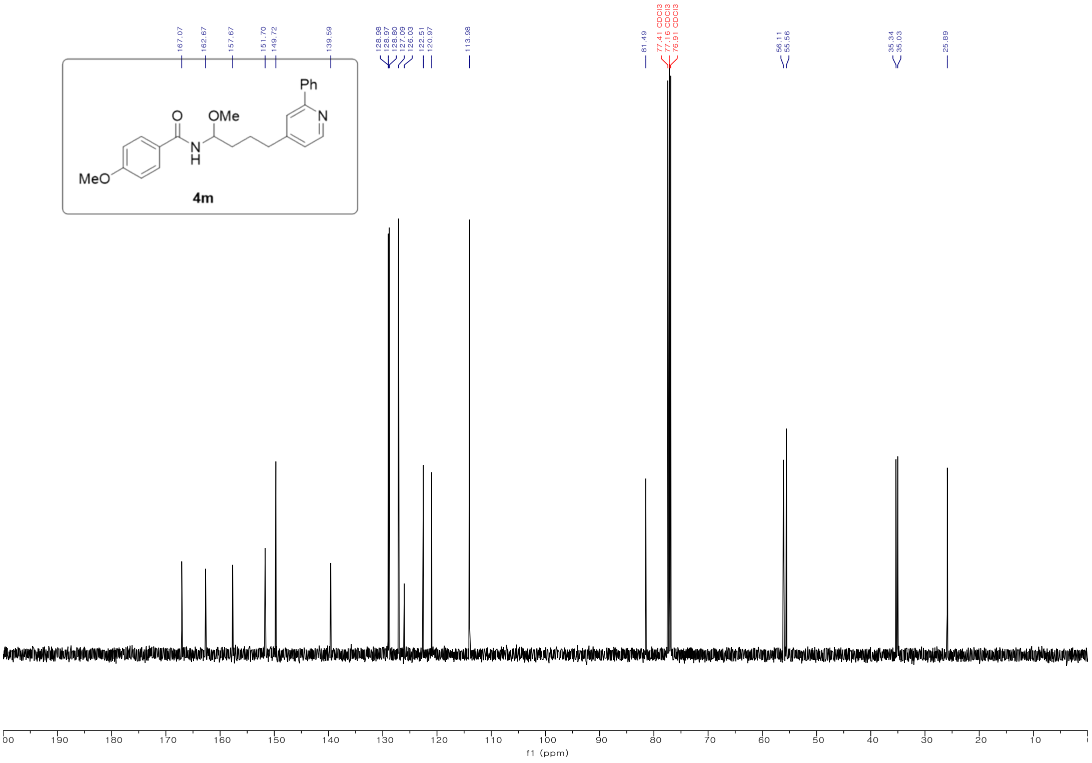

125 MHz, ^{13}C NMR in $CDCl_3$

N-(1-methoxy-3-(2-phenylpyridin-4-yl)propyl)pentanamide (4n)

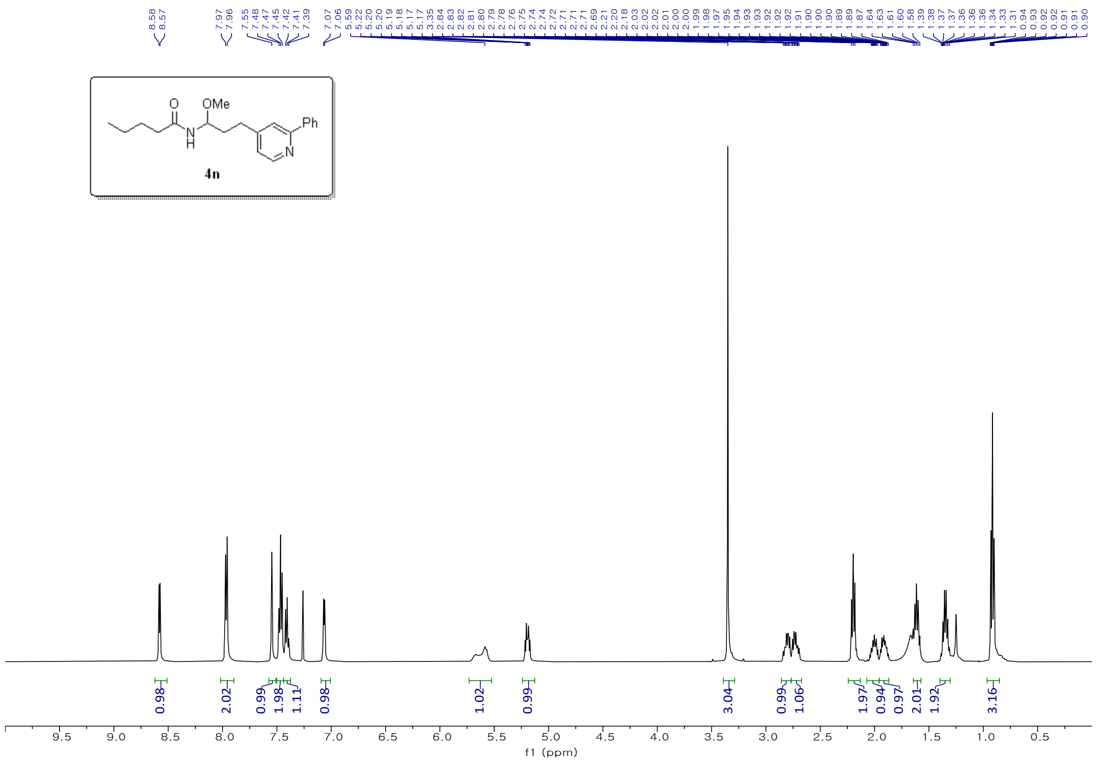

500 MHz, ^{1}H NMR in $CDCl_3$

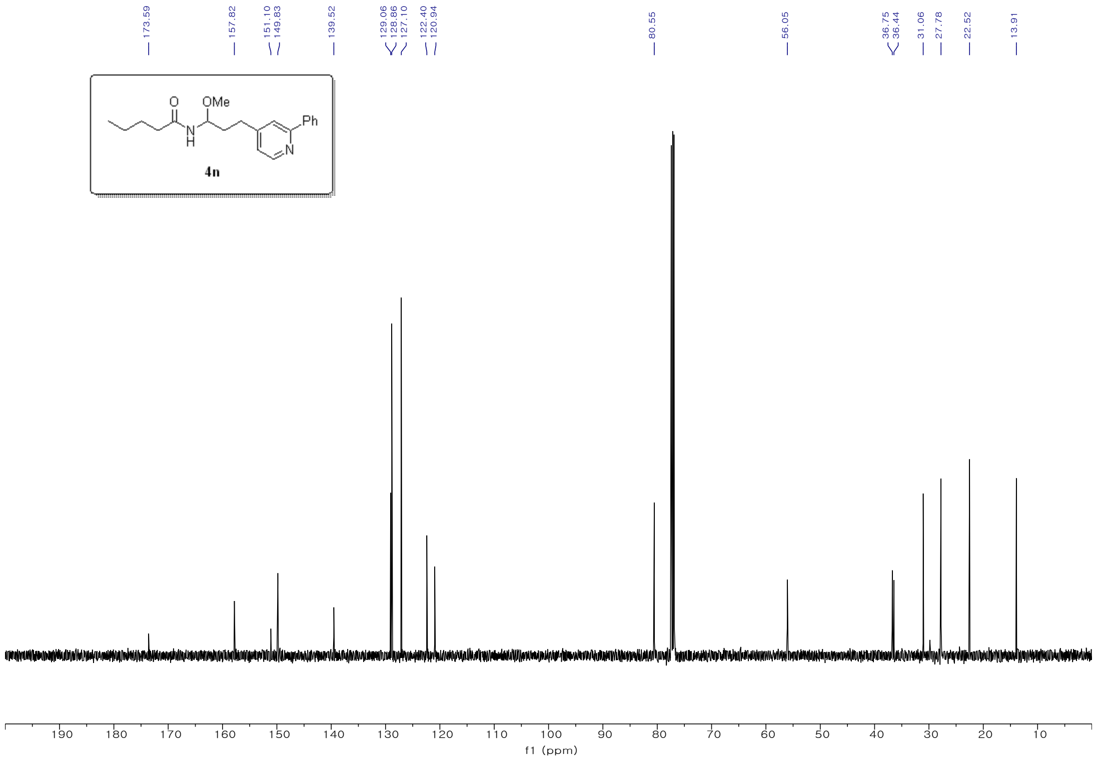

125 MHz, ^{13}C NMR in $CDCl_3$

N-(1-methoxy-3-(2-phenylpyridin-4-yl)propyl)-2-phenylacetamide (4o)

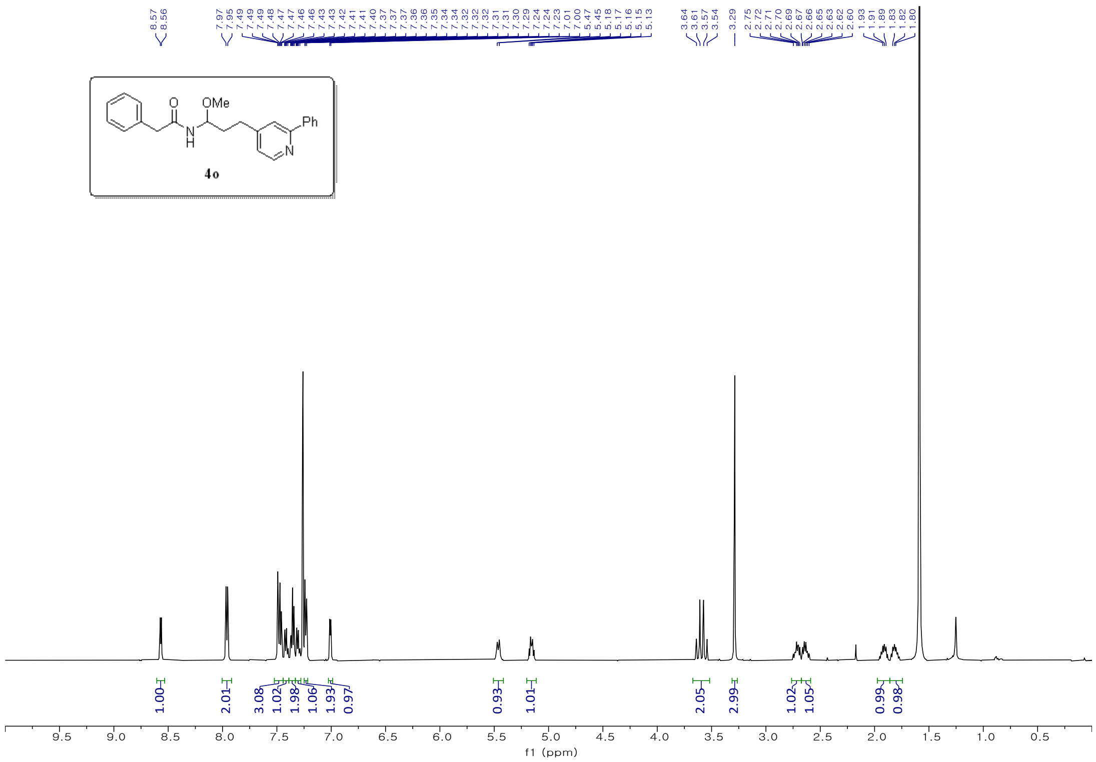

500 MHz, ^{1}H NMR in $CDCl_3$

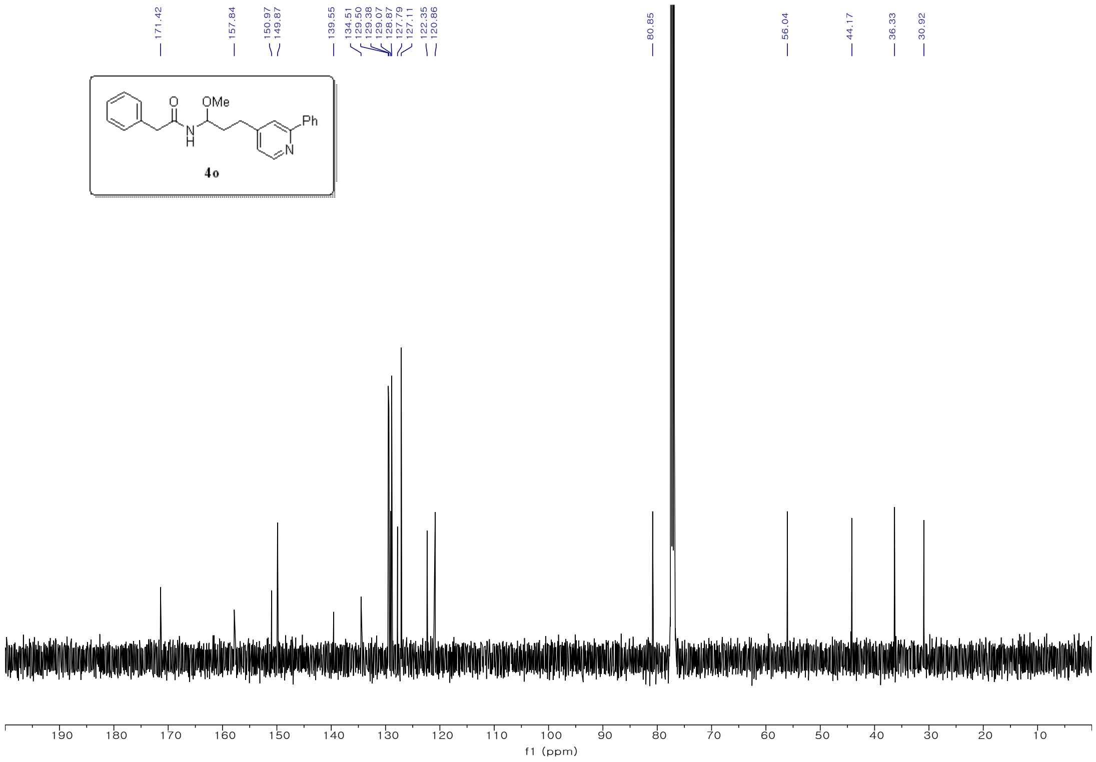

125 MHz, ^{13}C NMR in $CDCl_3$

N-(1-methoxy-3-(2-phenylpyridin-4-yl)propyl)cyclohexanecarboxamide (4p)

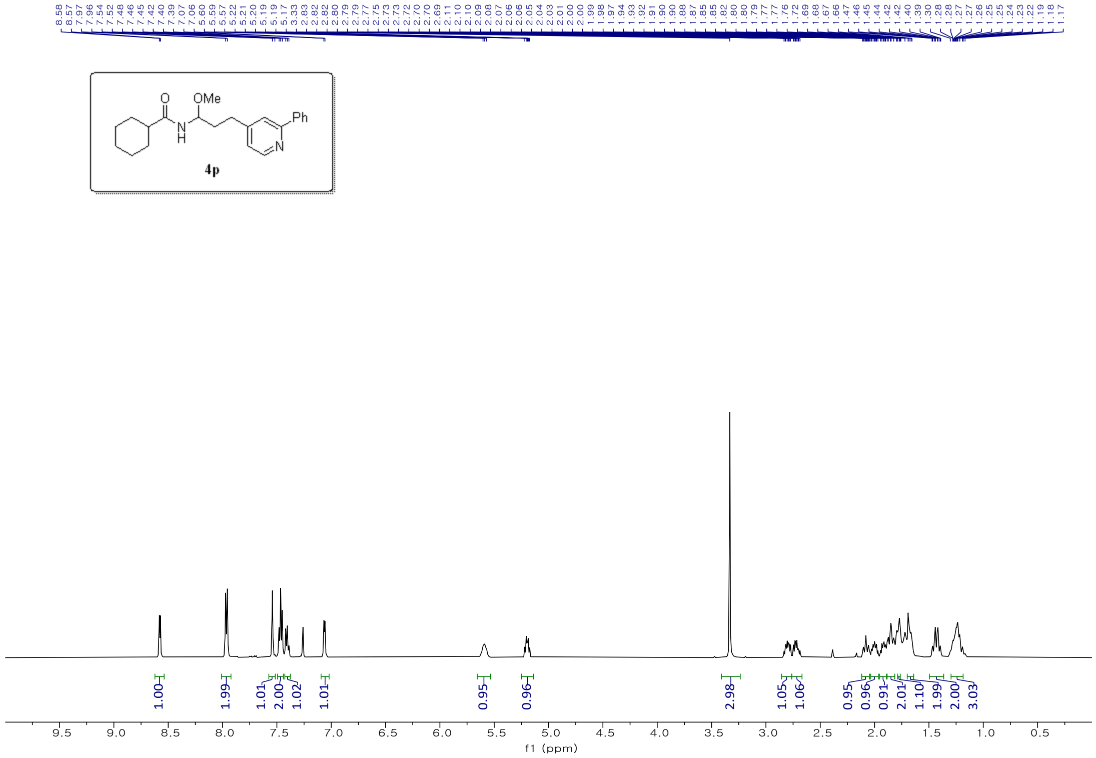

500 MHz, ^{1}H NMR in CDCl$_3$

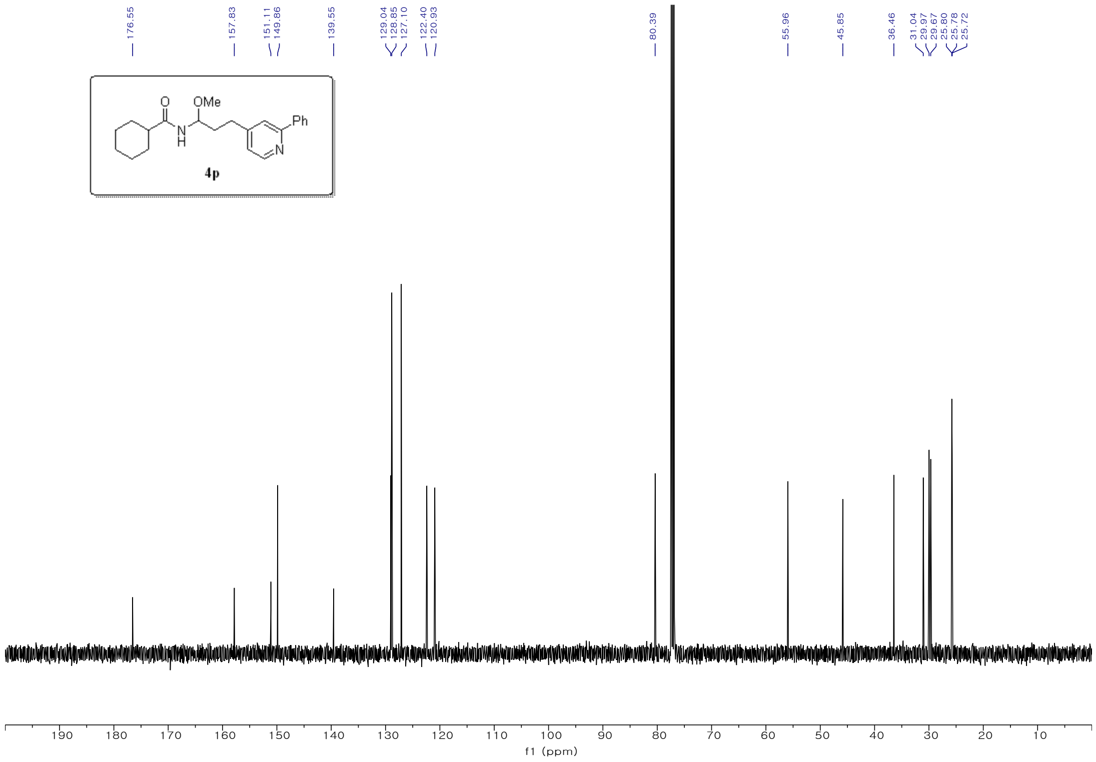

125 MHz, ^{13}C NMR in CDCl$_3$

N-(1-methoxy-3-(2-phenylpyridin-4-yl)propyl)-2-phenoxyacetamide (4q)

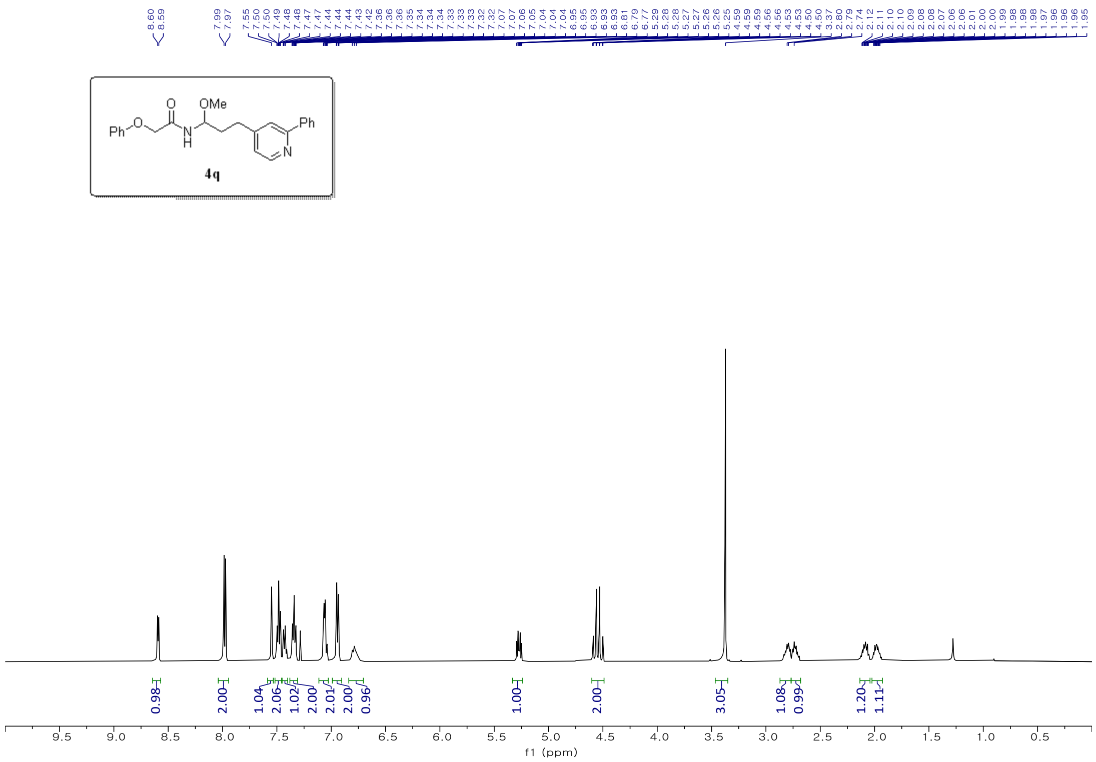

500 MHz, ^{1}H NMR in $CDCl_3$

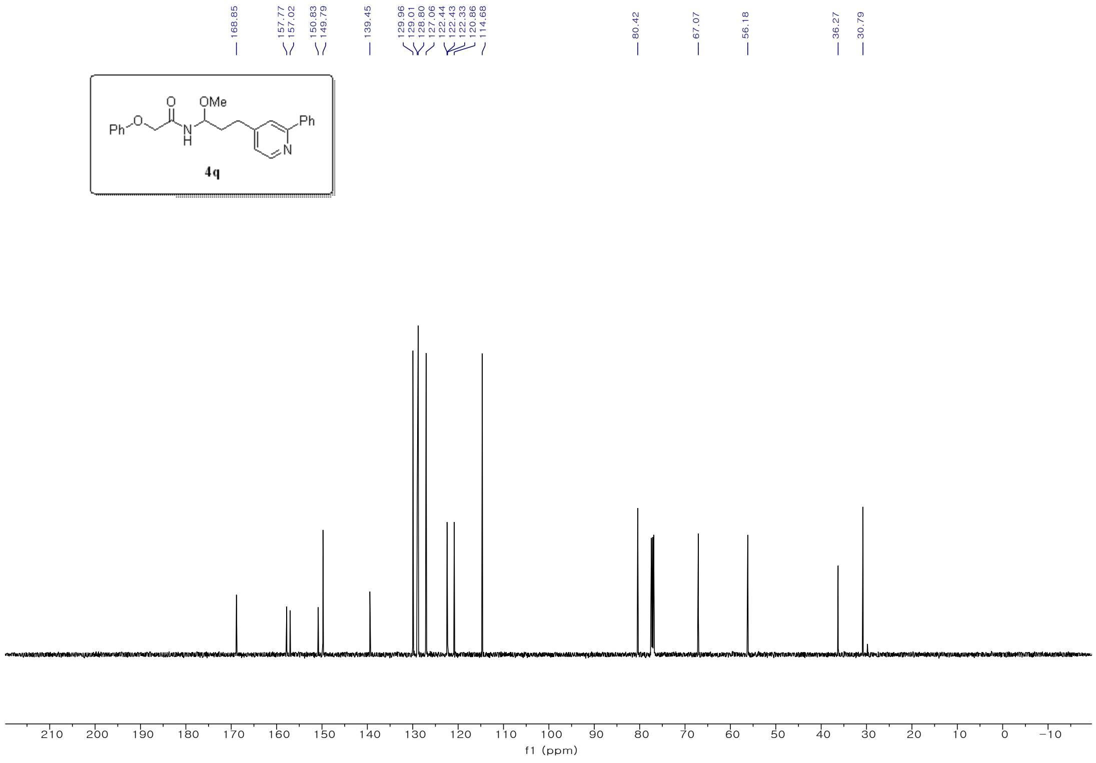

125 MHz, ^{13}C NMR in $CDCl_3$

benzyl (1-methoxy-3-(2-phenylpyridin-4-yl)propyl)carbamate (4r)

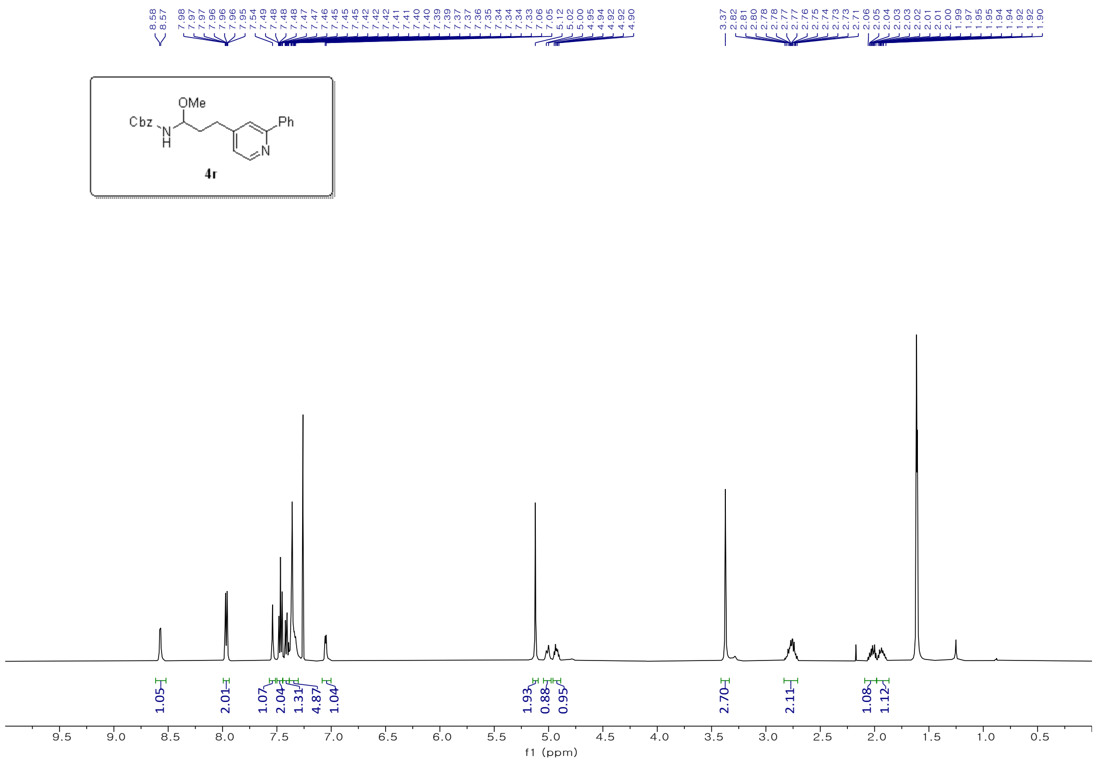

500 MHz, ^{1}H NMR in $CDCl_3$

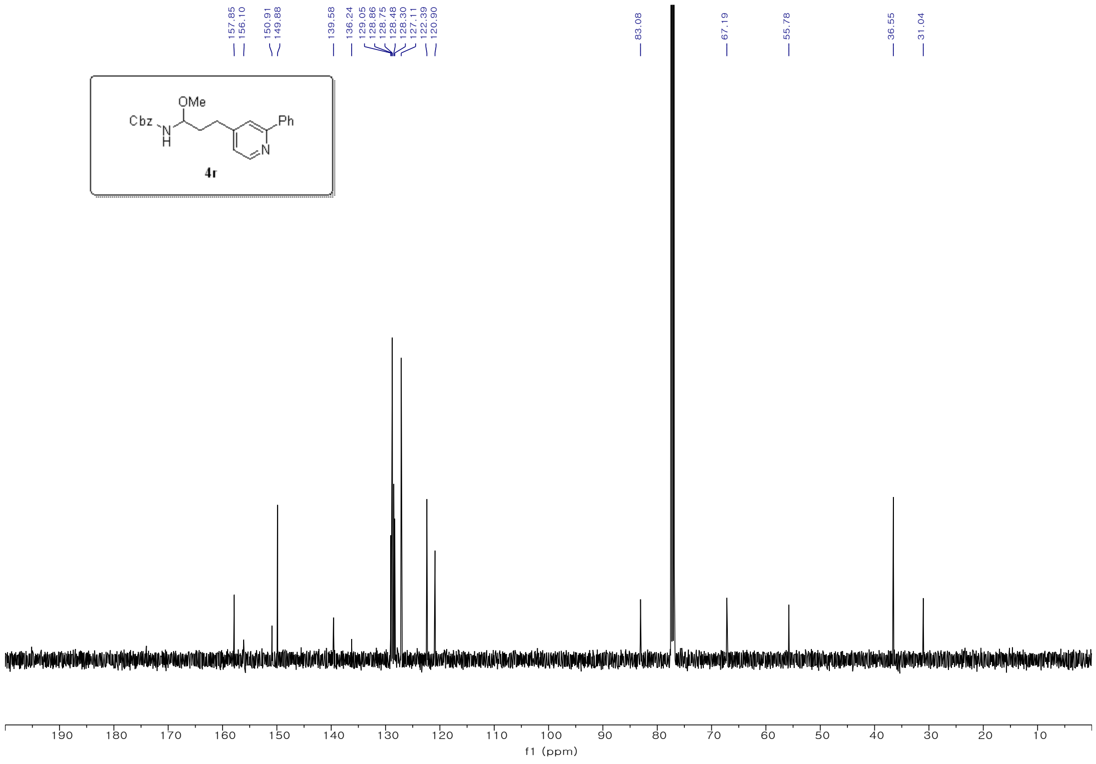

125 MHz, ^{13}C NMR in $CDCl_3$

2,2,2-trichloroethyl (1-methoxy-3-(2-phenylpyridin-4-yl)propyl)carbamate (4s)

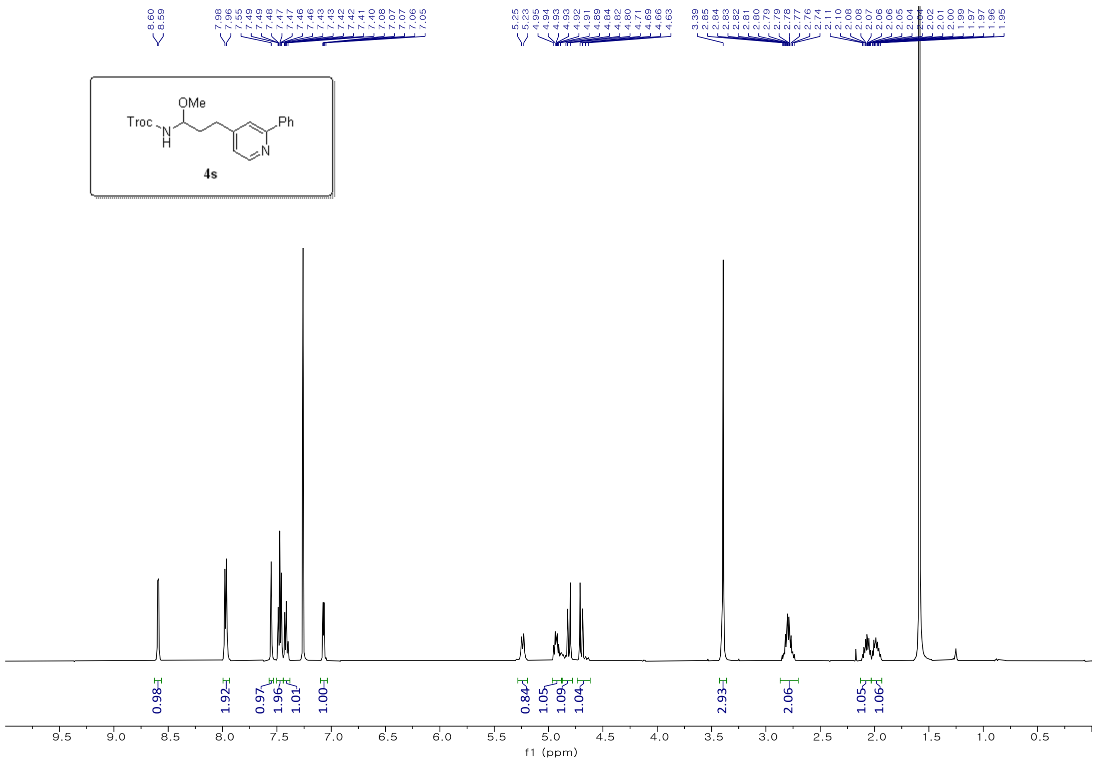

500 MHz, ^{1}H NMR in $CDCl_3$

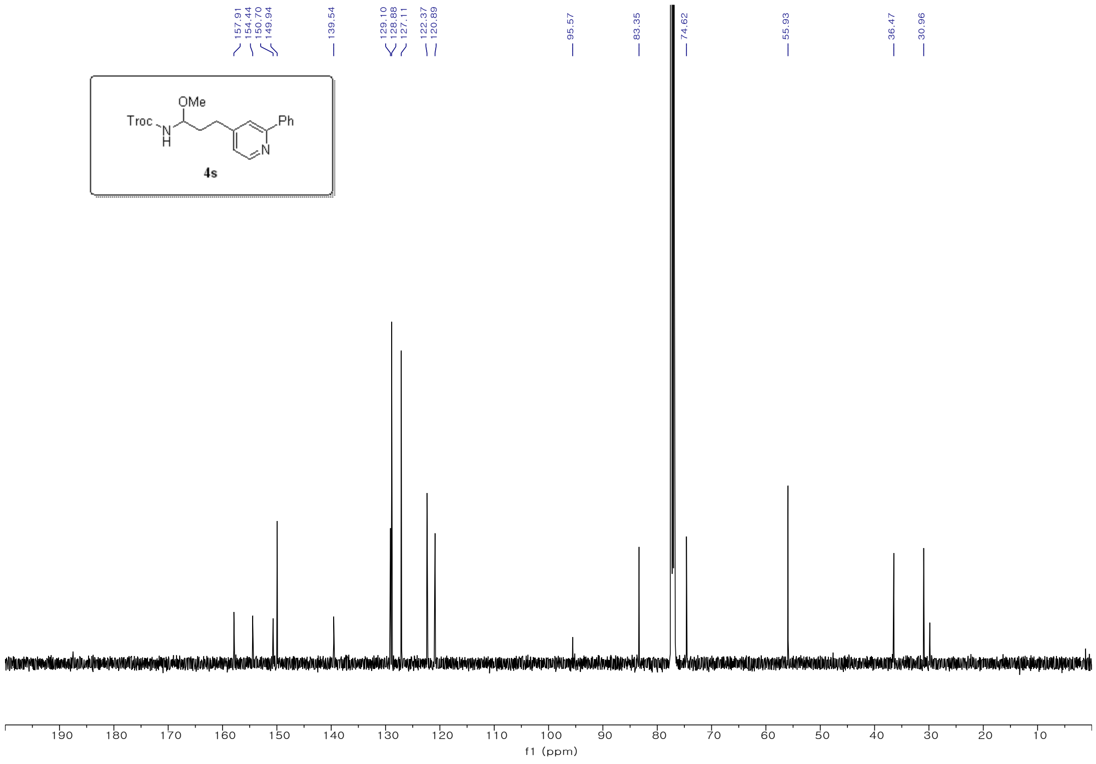

125 MHz, ^{13}C NMR in $CDCl_3$

4-methoxy-N-(1-methoxy-4-phenyl-3-(2-phenylpyridin-4-yl)butyl)benzamide (4t)

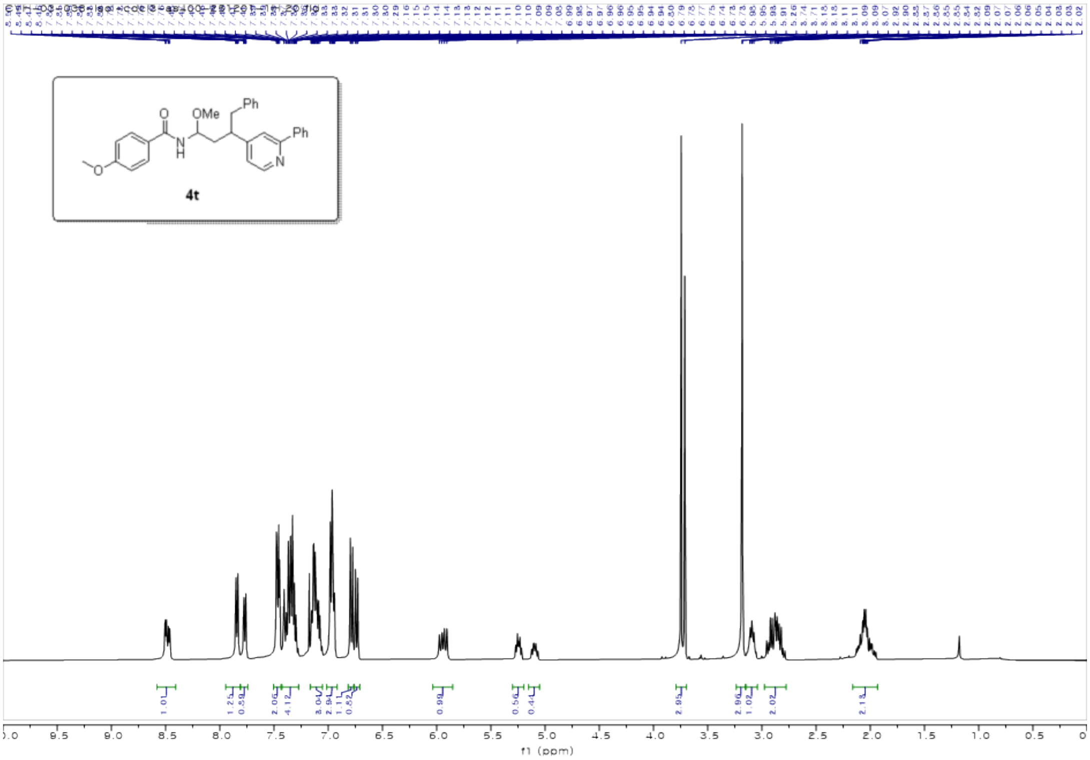

400 MHz, ^{1}H NMR in $CDCl_3$

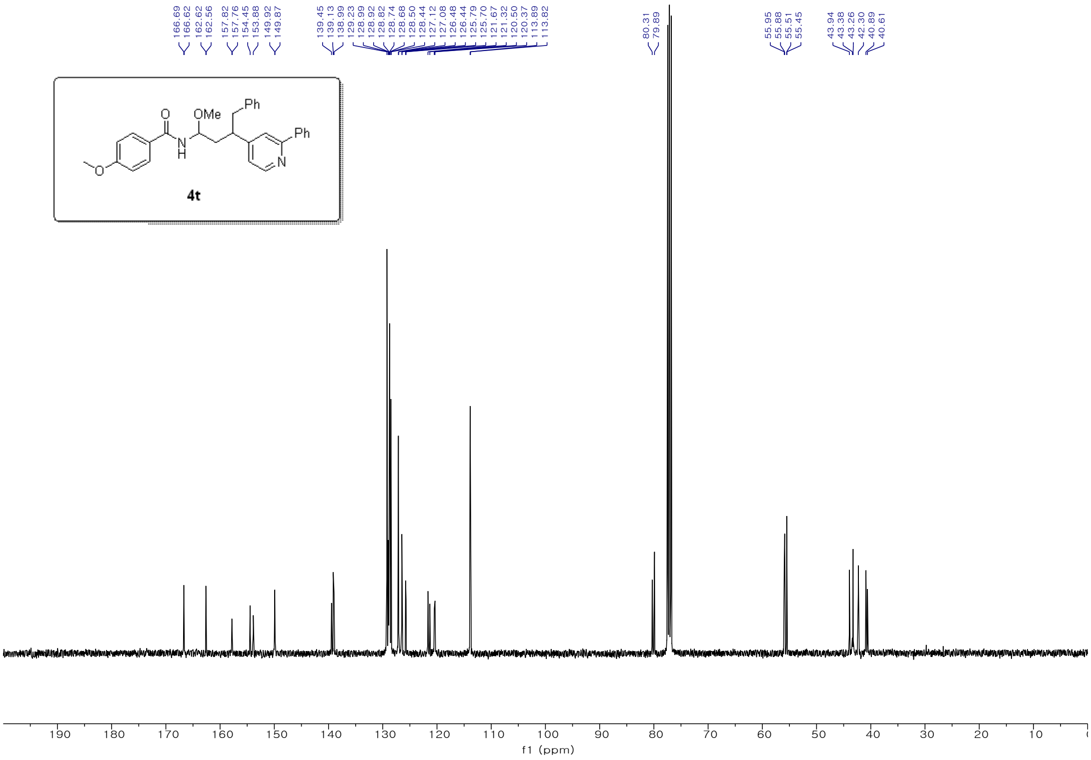

100 MHz, ^{13}C NMR in $CDCl_3$

N-(3,3-difluoro-1-methoxy-3-(2-phenylpyridin-4-yl)propyl)-4-methoxybenzamide (4u)

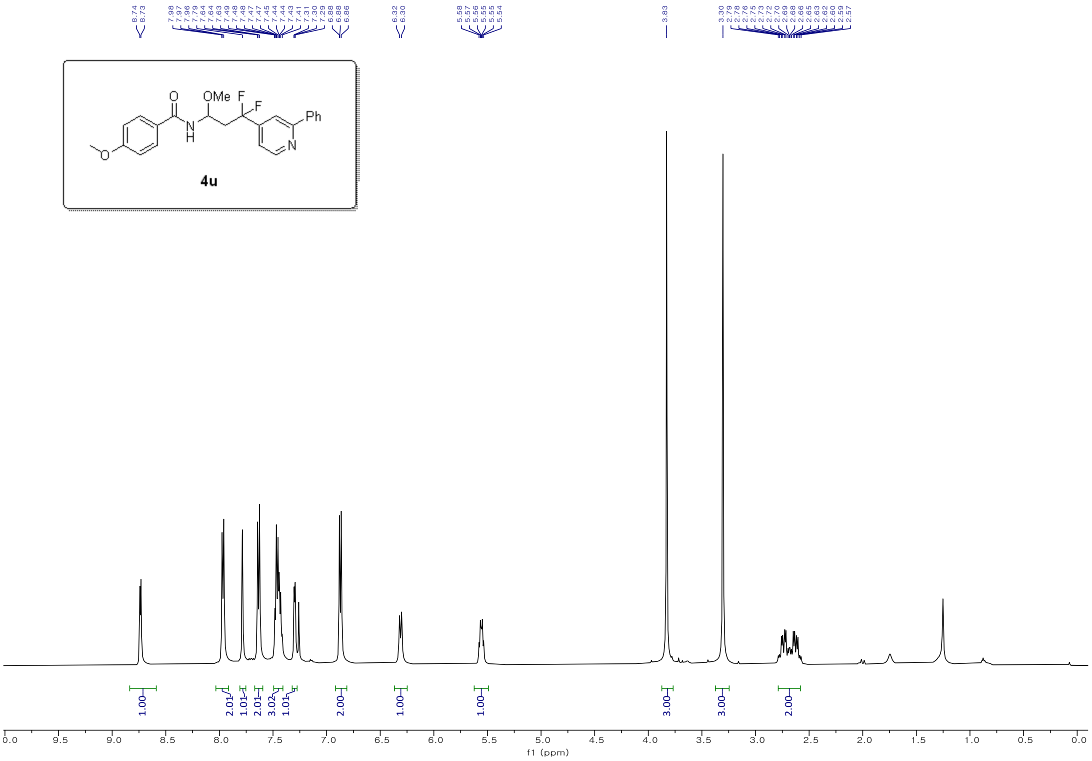

500 MHz, ^{1}H NMR in $CDCl_3$

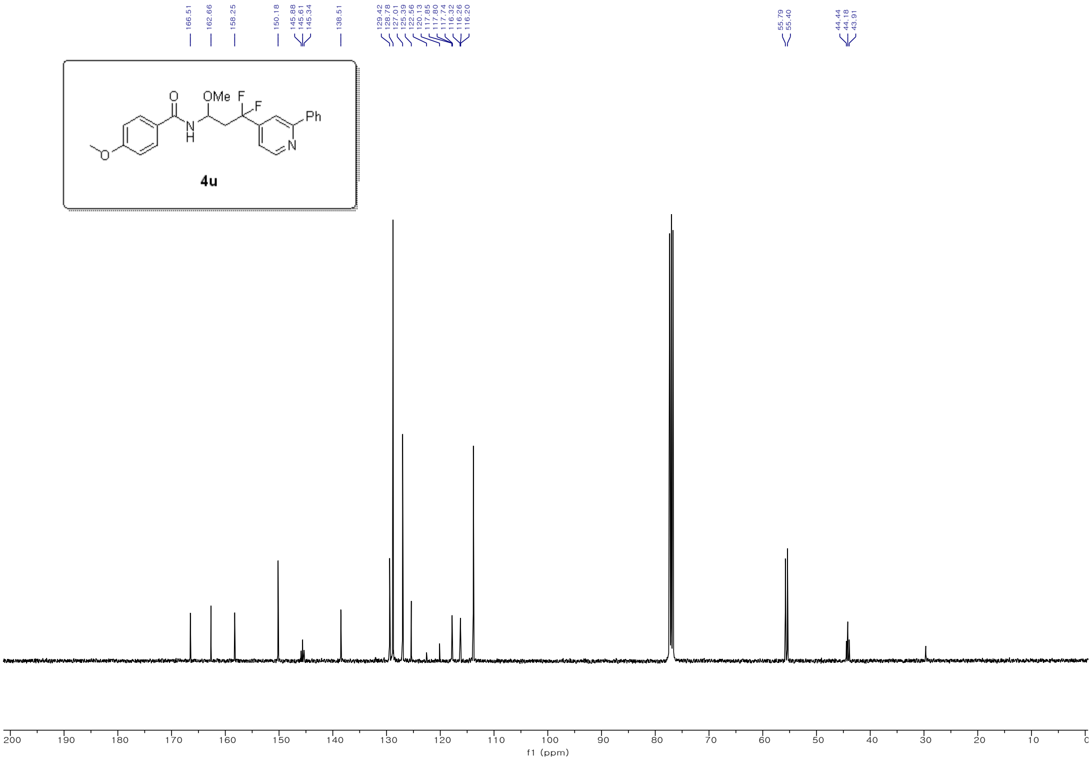

100 MHz, ^{13}C NMR in $CDCl_3$

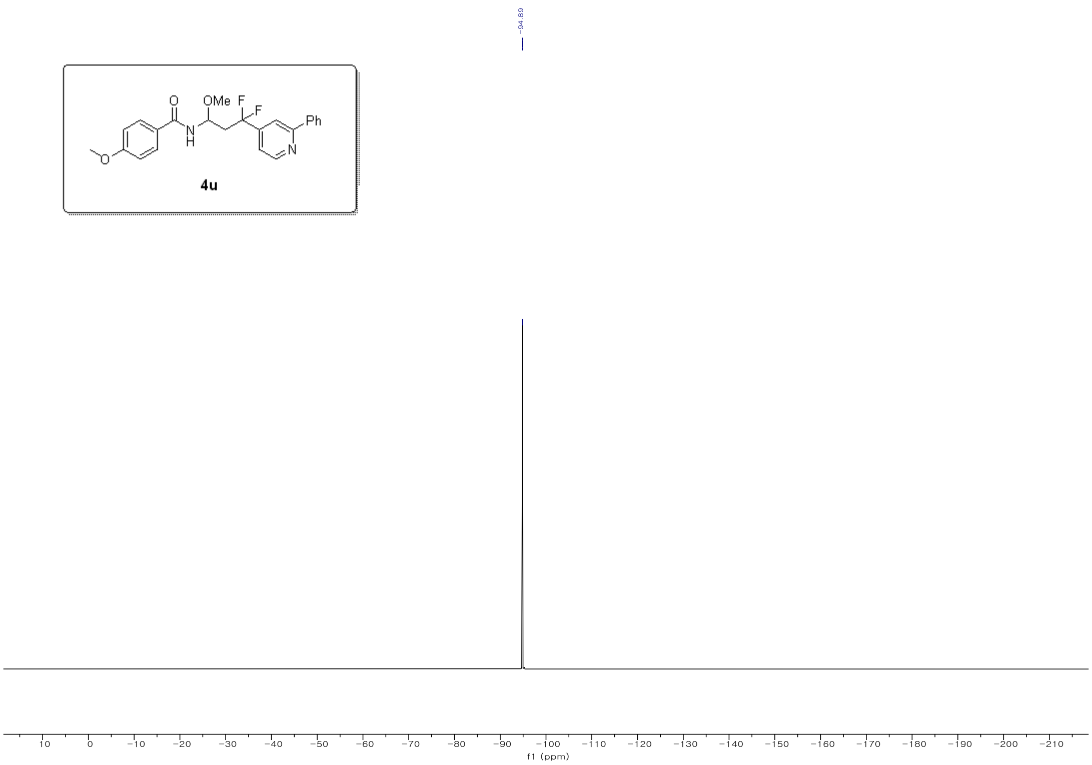

376 MHz, ^{19}F NMR in $CDCl_3$

4-methoxy-N-(1-methoxy-3-(2-phenylpyridin-4-yl)propyl)-N-methylbenzamide (4v)

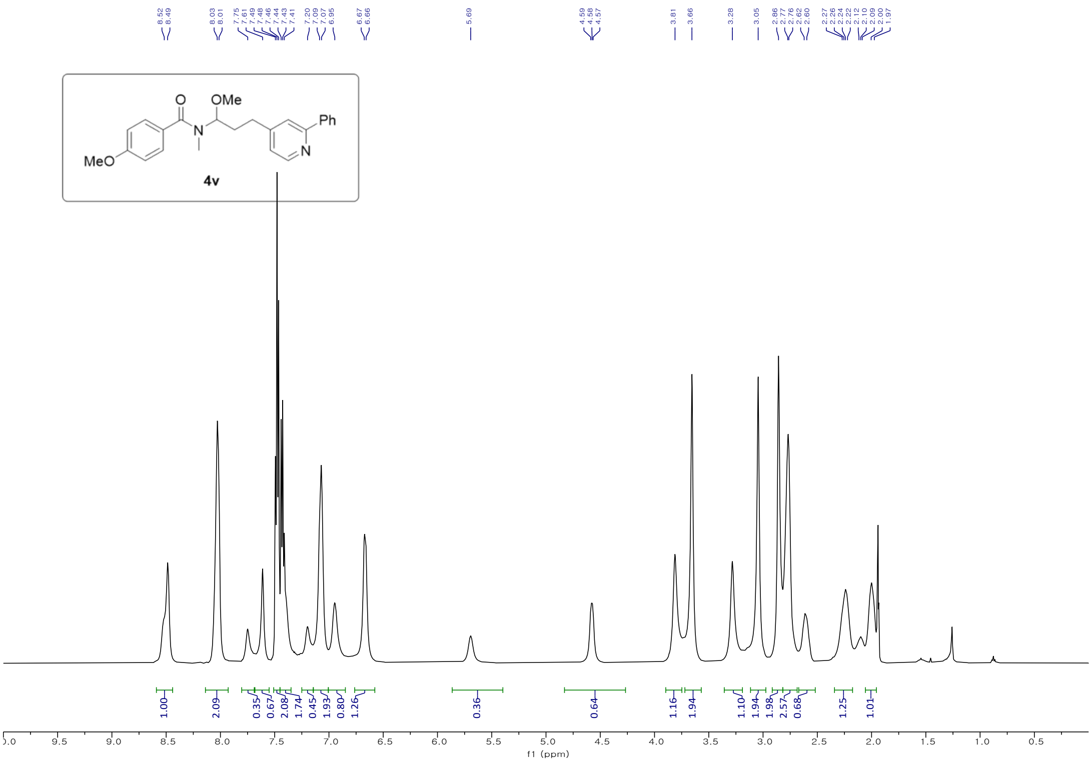

500 MHz, ^{1}H NMR in C_2D_3N

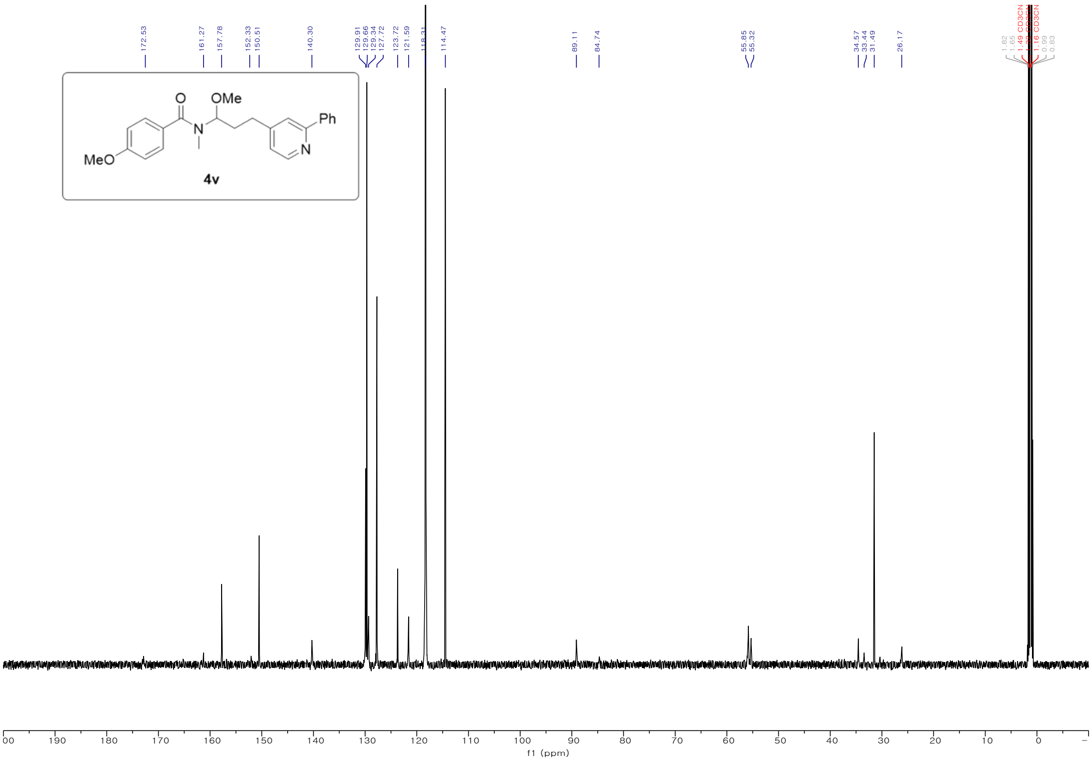

125 MHz, ^{13}C NMR in C_2D_3N

N-(1-hydroxy-3-(2-phenylpyridin-4-yl)propyl)-4-methoxybenzamide (4w).

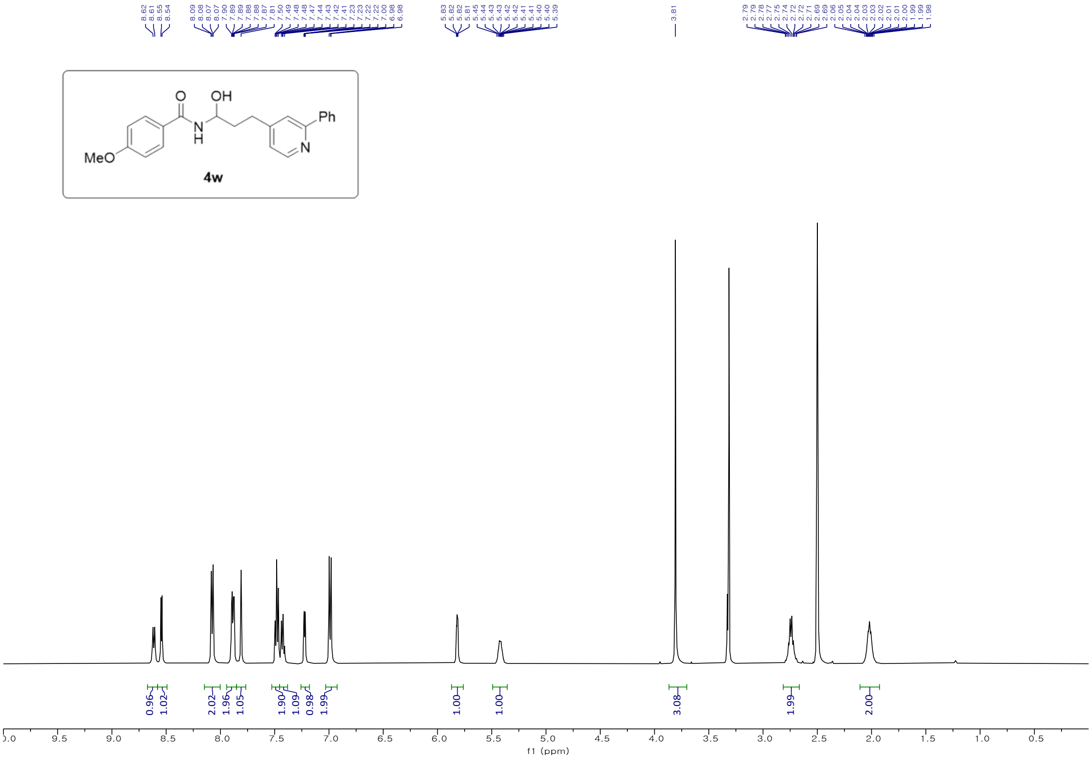

500 MHz, ^{1}H NMR in C_2D_6SO

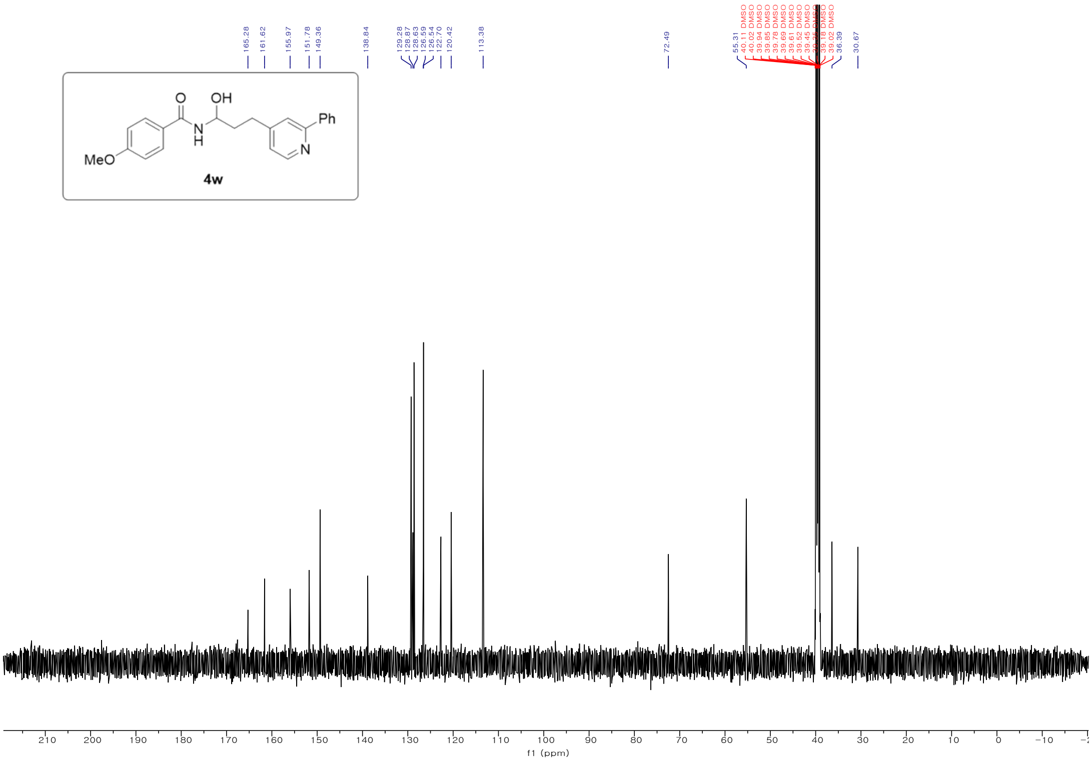

125 MHz, ^{13}C NMR in C_2D_6SO

N-(1-methoxy-3-(2-phenylpyridin-4-yl)propyl)-2-(11-oxo-6,11-dihydrodibenzo[b,e]oxepin-2-yl)acetamide (4x)

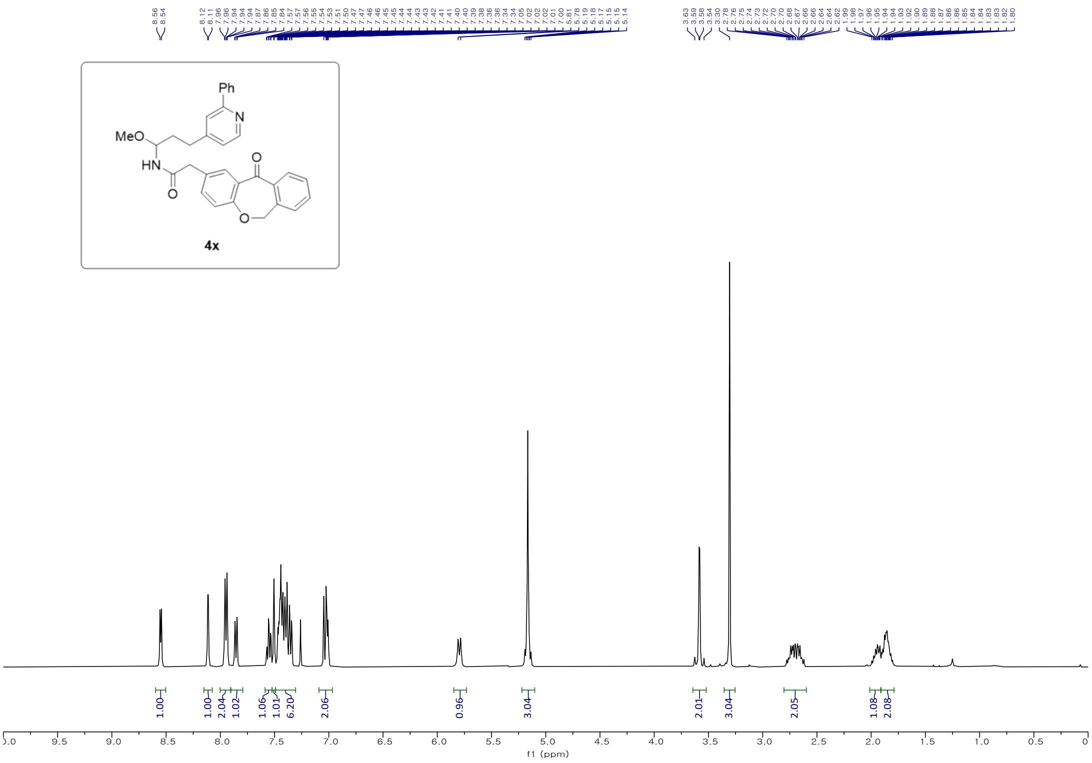

400 MHz, ^{1}H NMR in $CDCl_3$

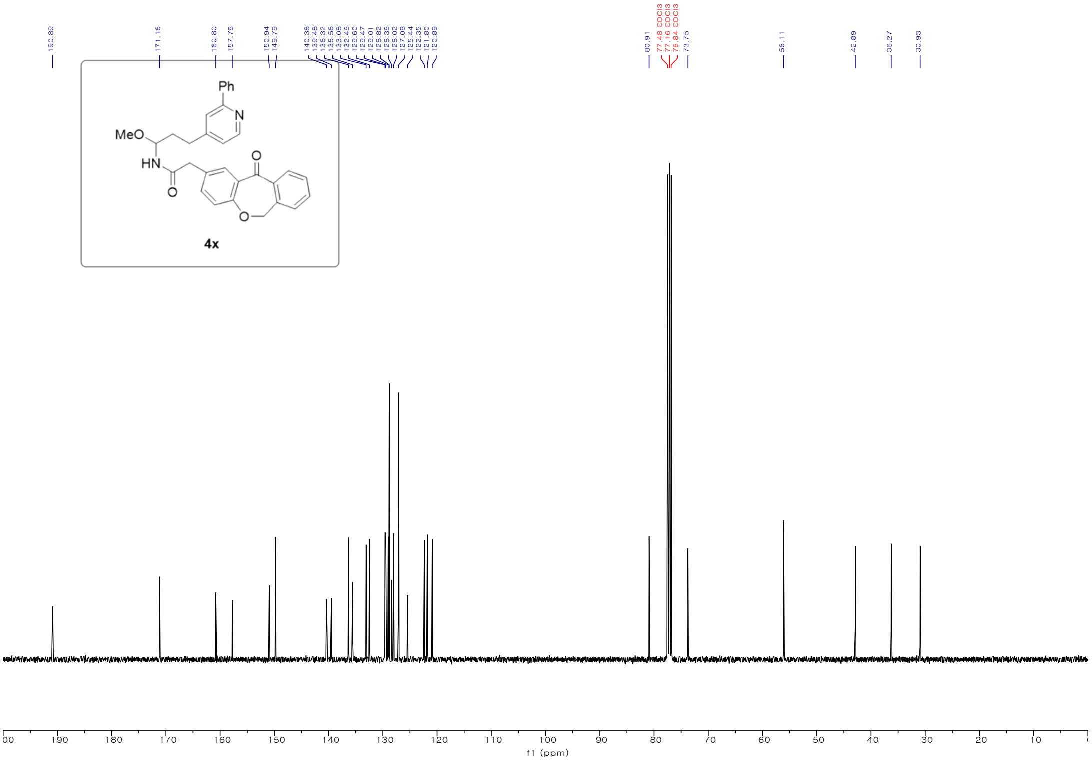

100 MHz, ^{13}C NMR in $CDCl_3$

3-(4,5-diphenyloxazol-2-yl)-N-(1-methoxy-3-(2-phenylpyridin-4-yl)propyl)propanamide (4y)

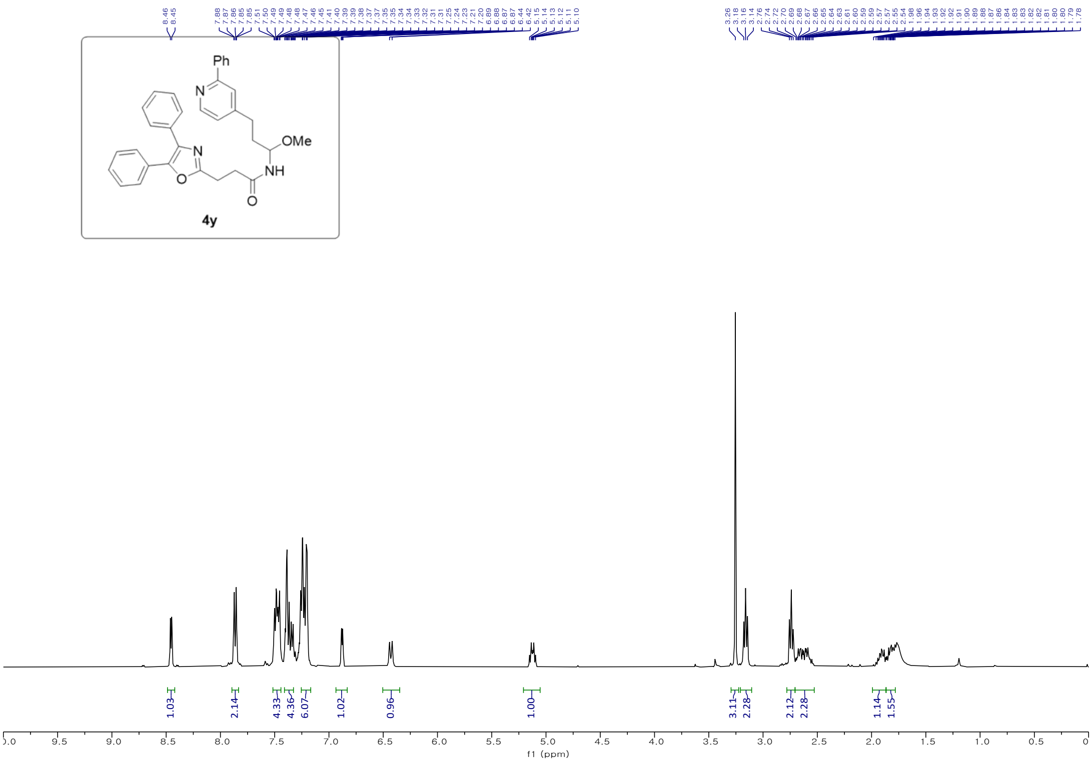

400 MHz, ^{1}H NMR in $CDCl_3$

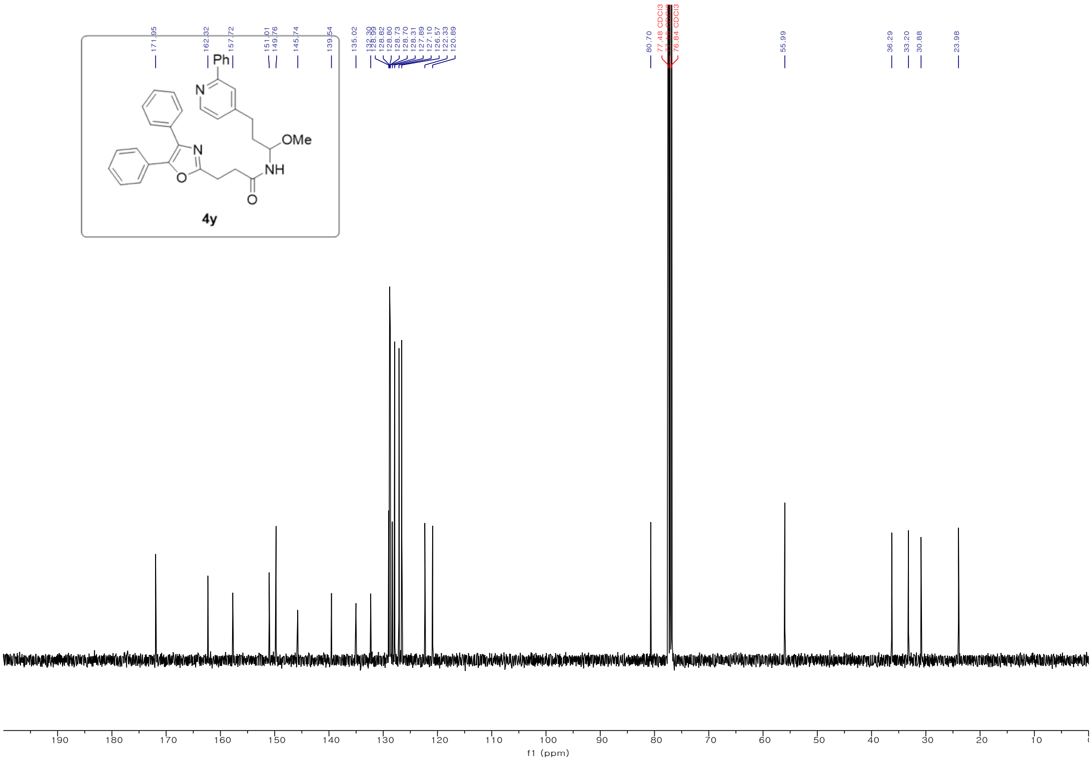

100 MHz, ^{13}C NMR in $CDCl_3$

4-(N,N-dipropylsulfamoyl)-N-(1-methoxy-3-(2-phenylpyridin-4-yl)propyl)benzamide (4z)

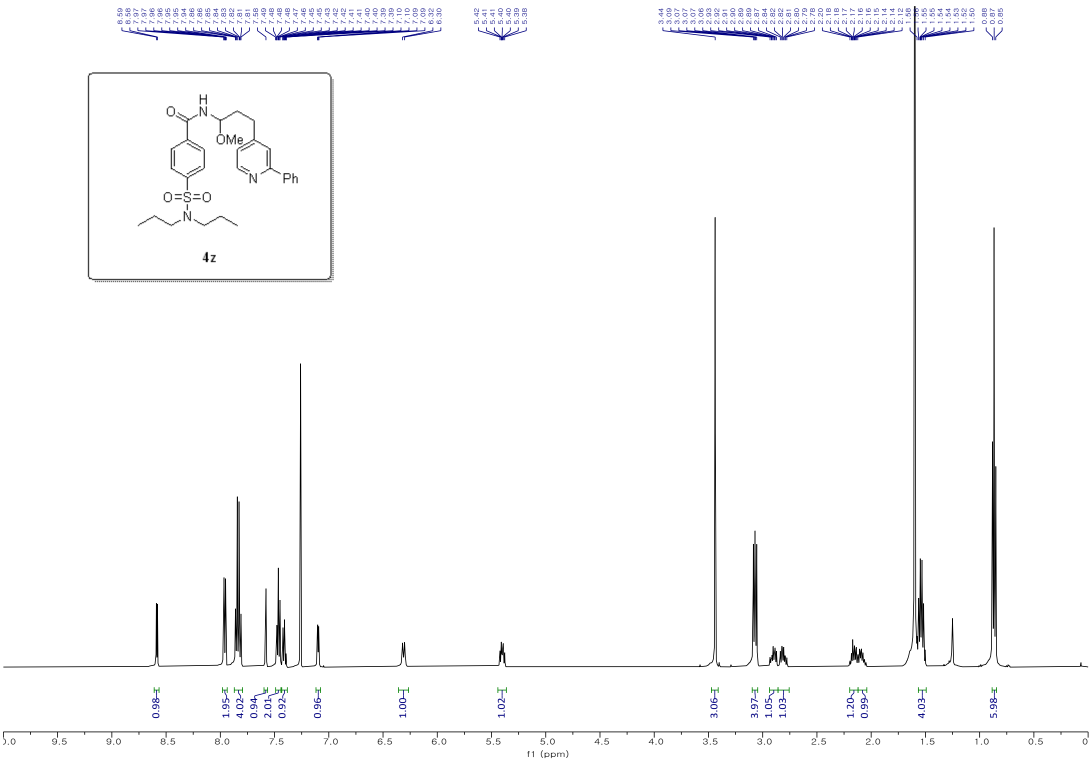

500 MHz, ^{1}H NMR in CDCl$_3$

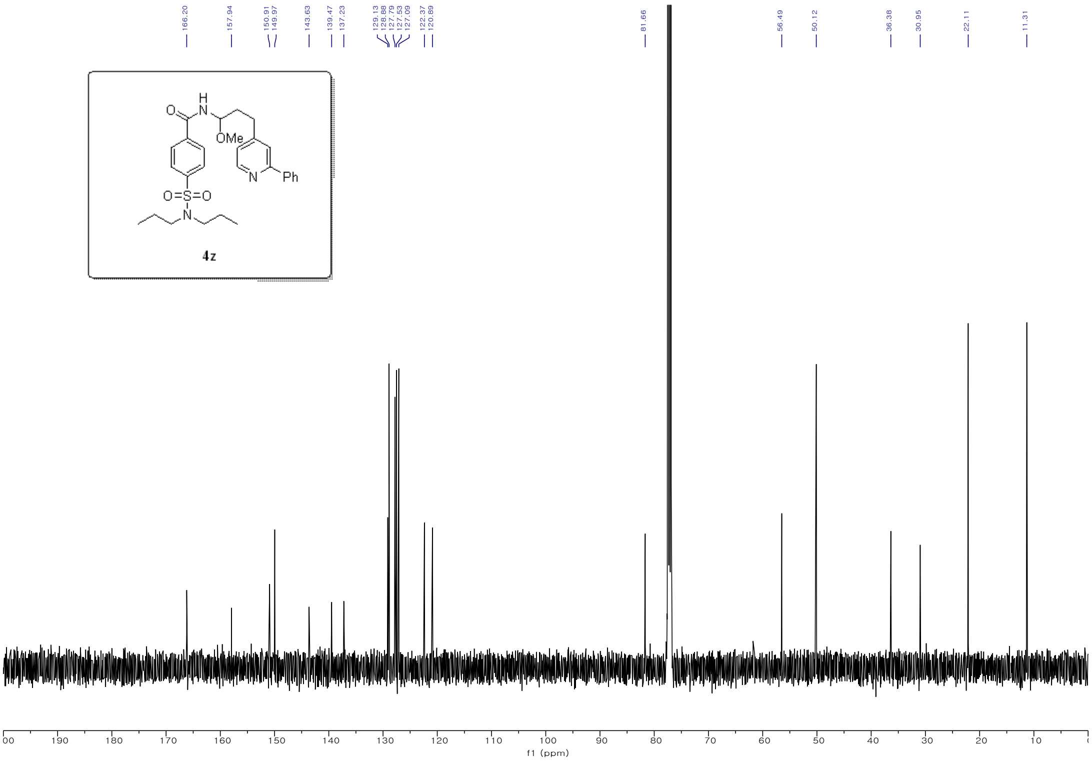

125 MHz, ^{13}C NMR in CDCl$_3$

N-(1-azido-3-(2-phenylpyridin-4-yl)propyl)-4-methoxybenzamide (5a).

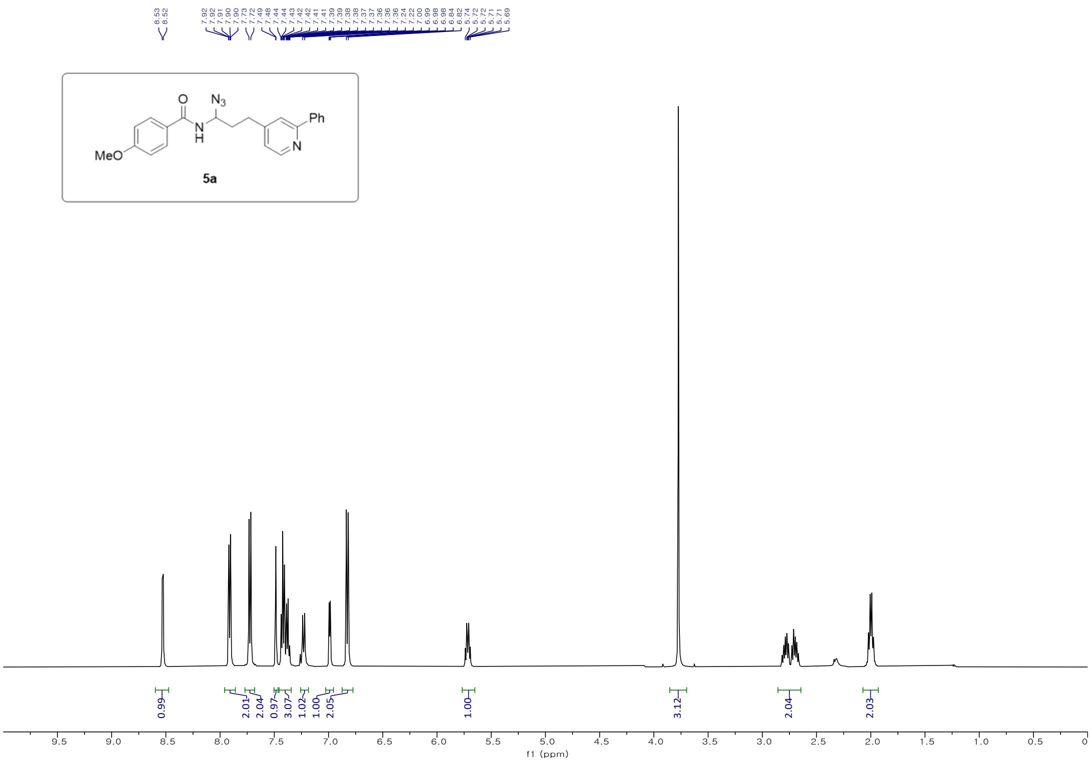

500 MHz, ^{1}H NMR in CDCl$_3$

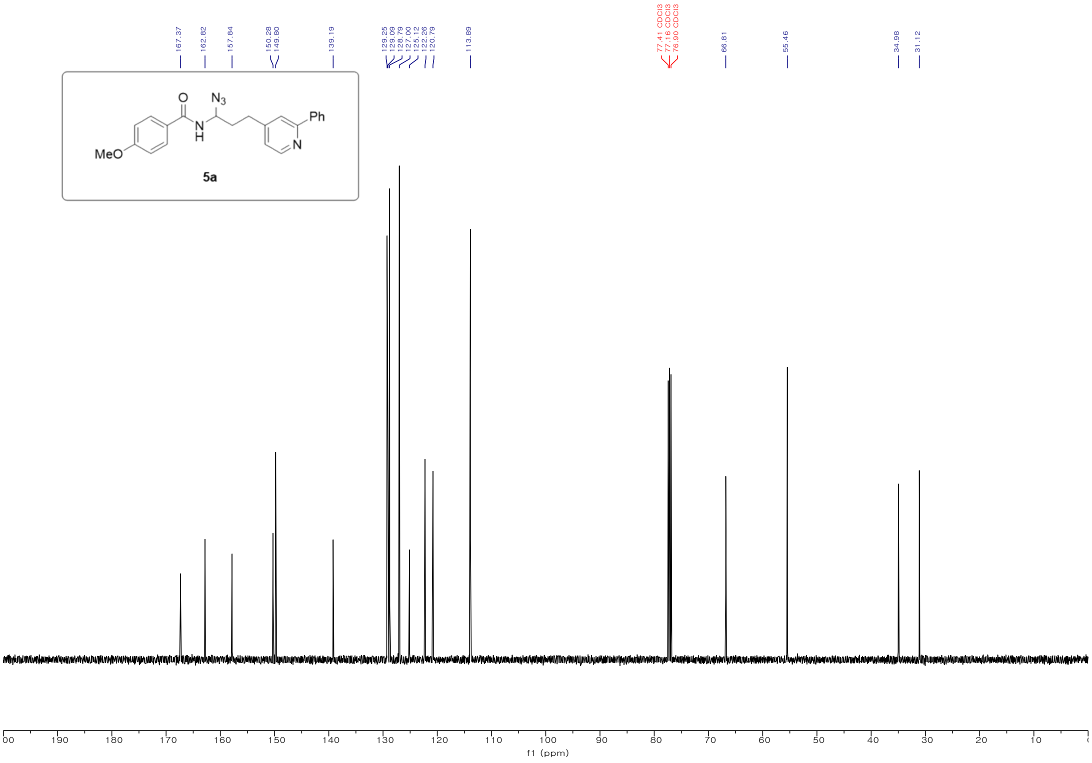

125 MHz, ^{13}C NMR in CDCl$_3$

N-(1-azido-3-(2-phenylquinolin-4-yl)propyl)-4-methoxybenzamide (5b).

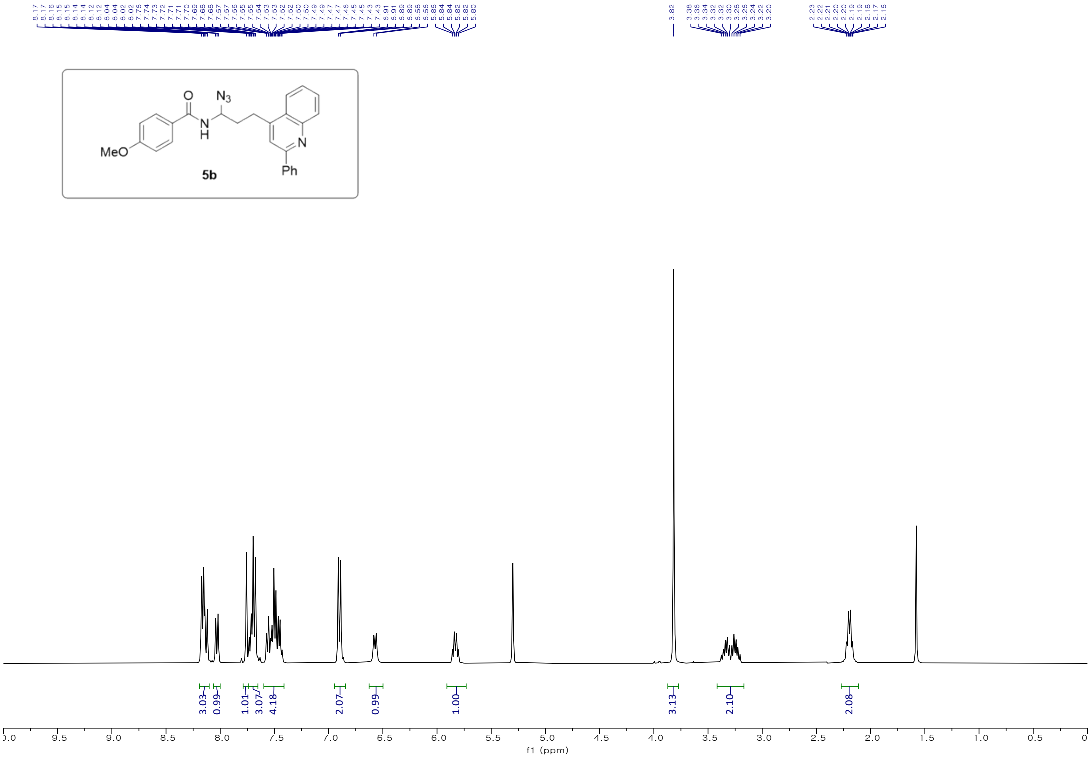

400 MHz, ^{1}H NMR in CD_2Cl_2

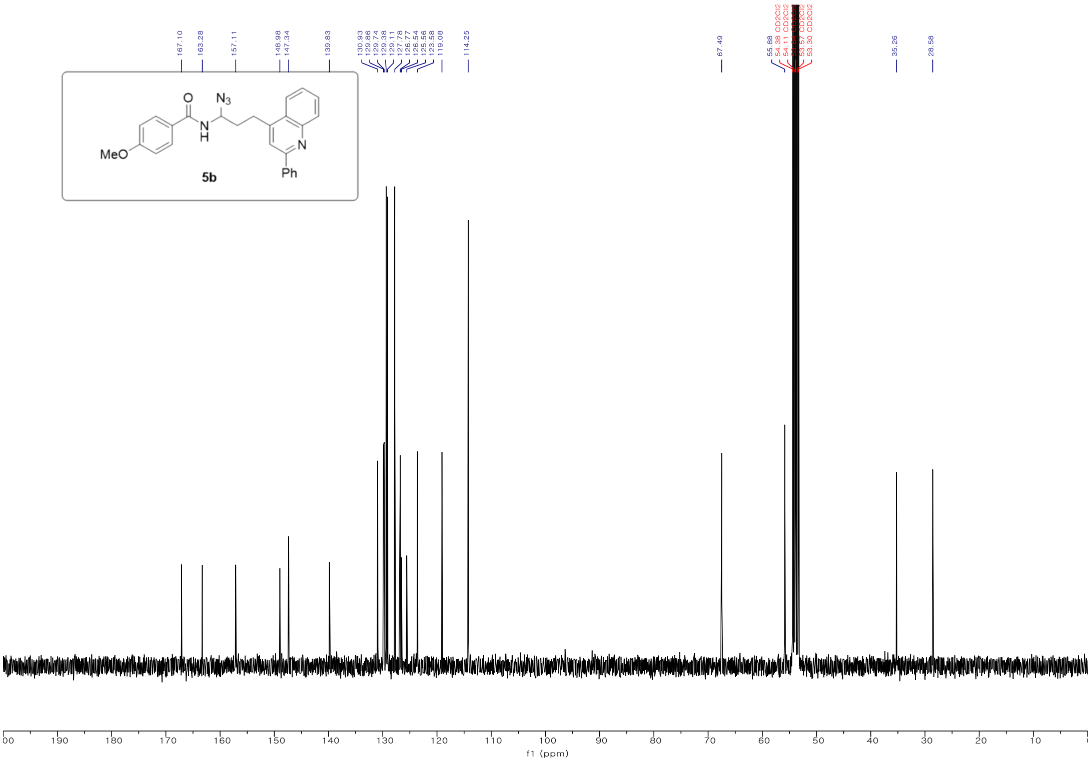

100 MHz, ^{13}C NMR in CD_2Cl_2

N-(1-azido-3-(2-phenylpyridin-4-yl)propyl)-2-methoxybenzamide (5c)

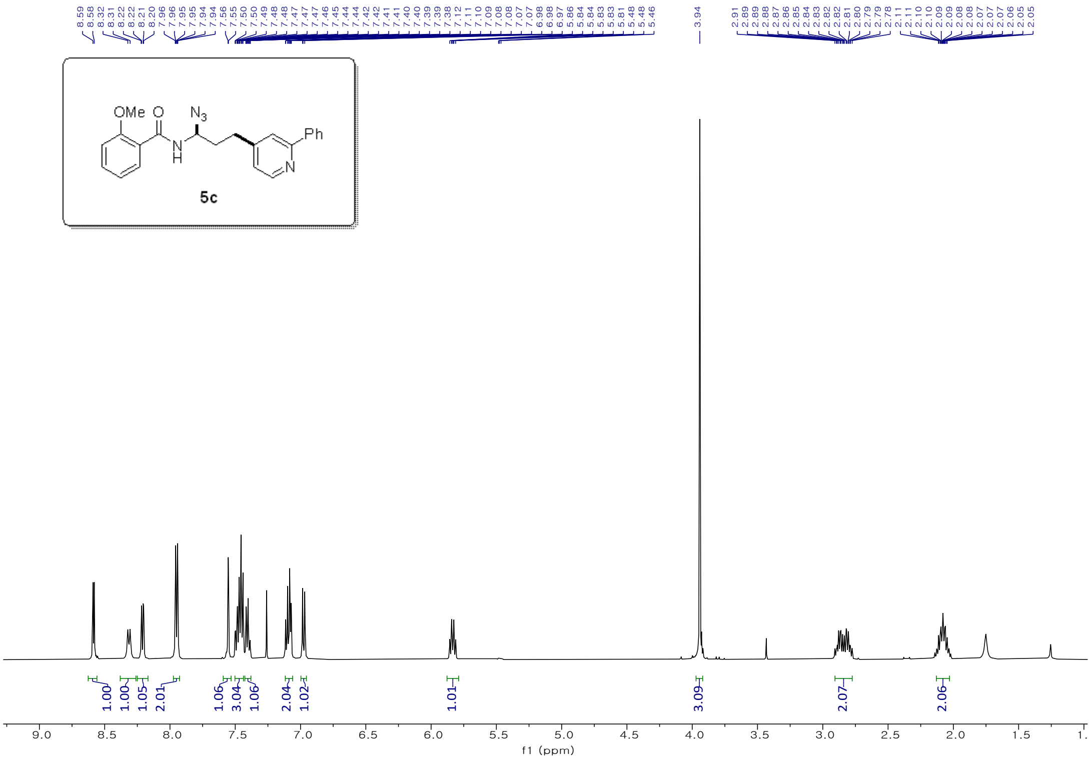

500 MHz, ^{1}H NMR in $CDCl_3$

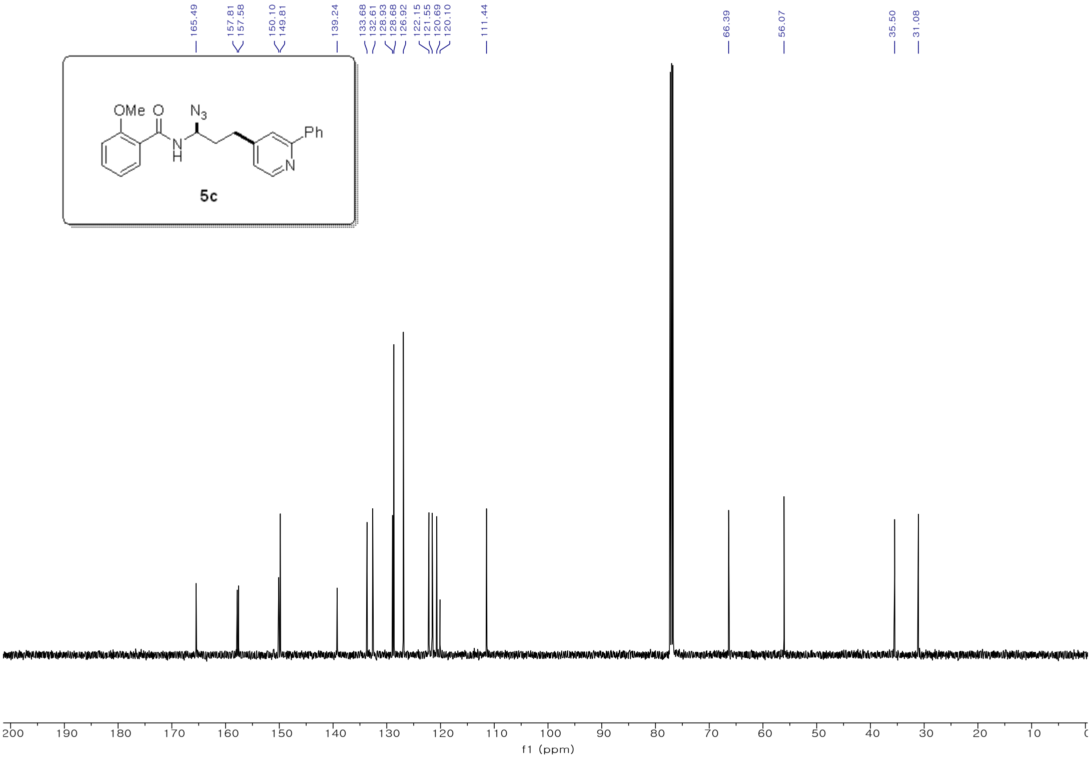

125 MHz, ^{13}C NMR in $CDCl_3$

N-(1-azido-3-(2-phenylpyridin-4-yl)propyl)-2-naphthamide (5d)

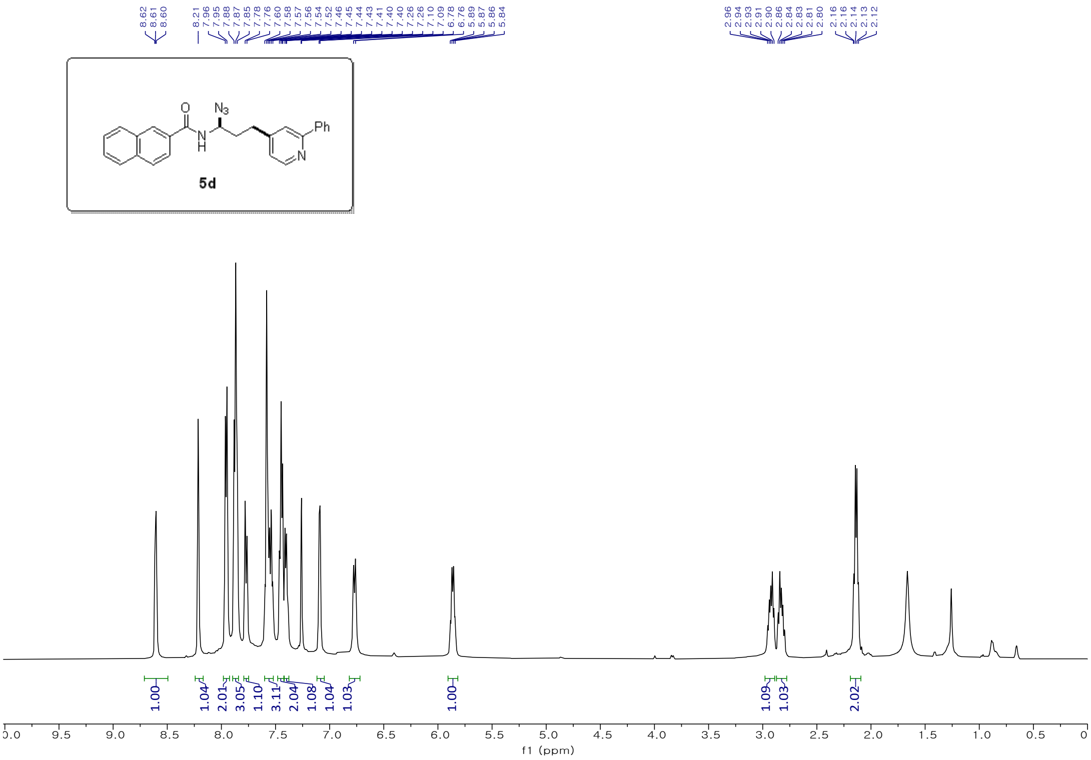

500 MHz, ^{1}H NMR in $CDCl_3$

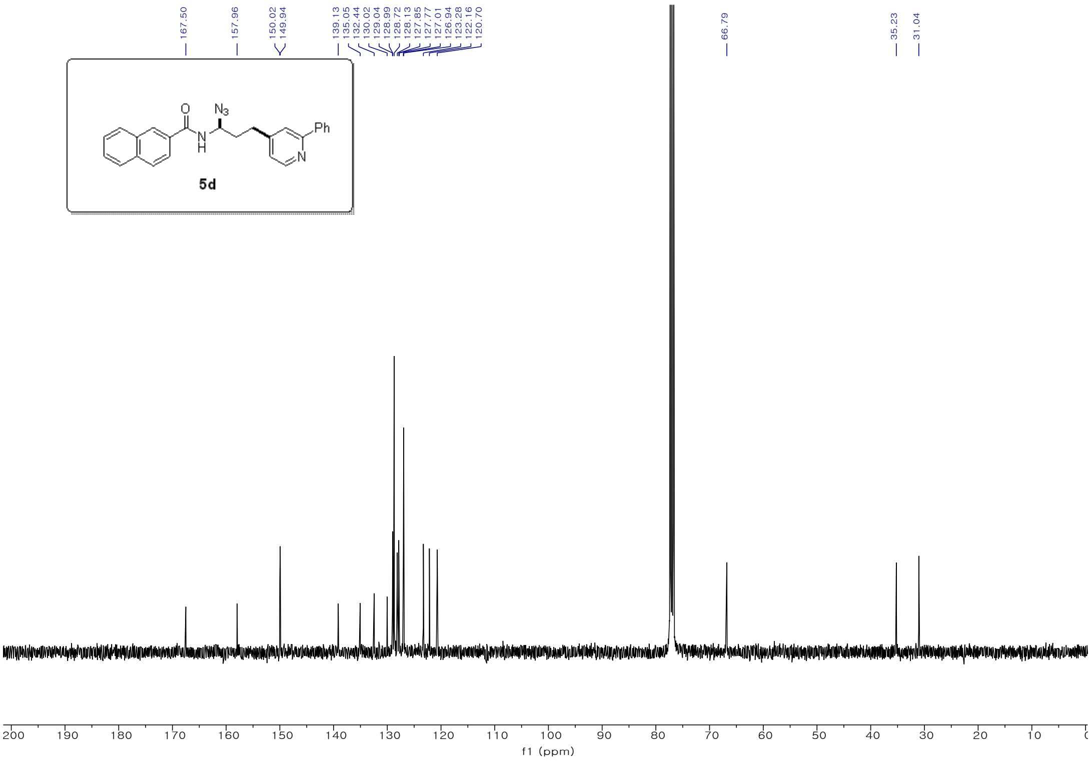

100 MHz, ^{13}C NMR in $CDCl_3$

N-(1-azido-3-(2-((1-(4-phenoxyphenoxy)propan-2-yl)oxy)pyridin-4-yl)propyl)-4-methoxybenzamide (5e)

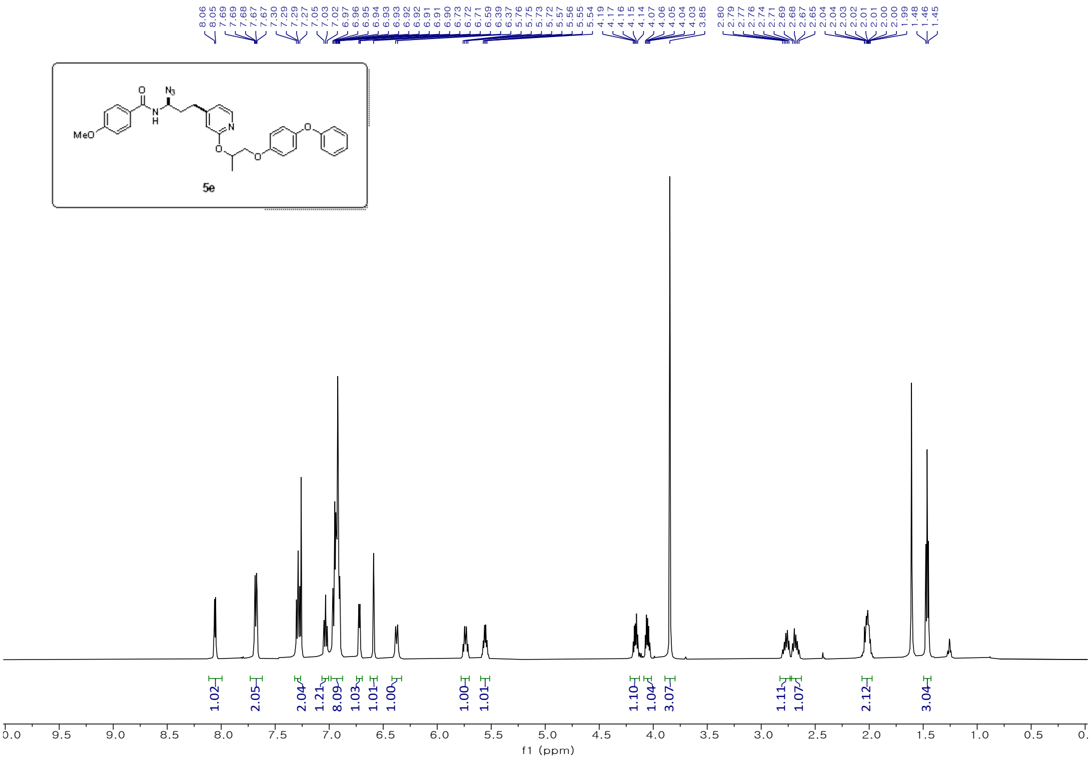

500 MHz, ^{1}H NMR in $CDCl_3$

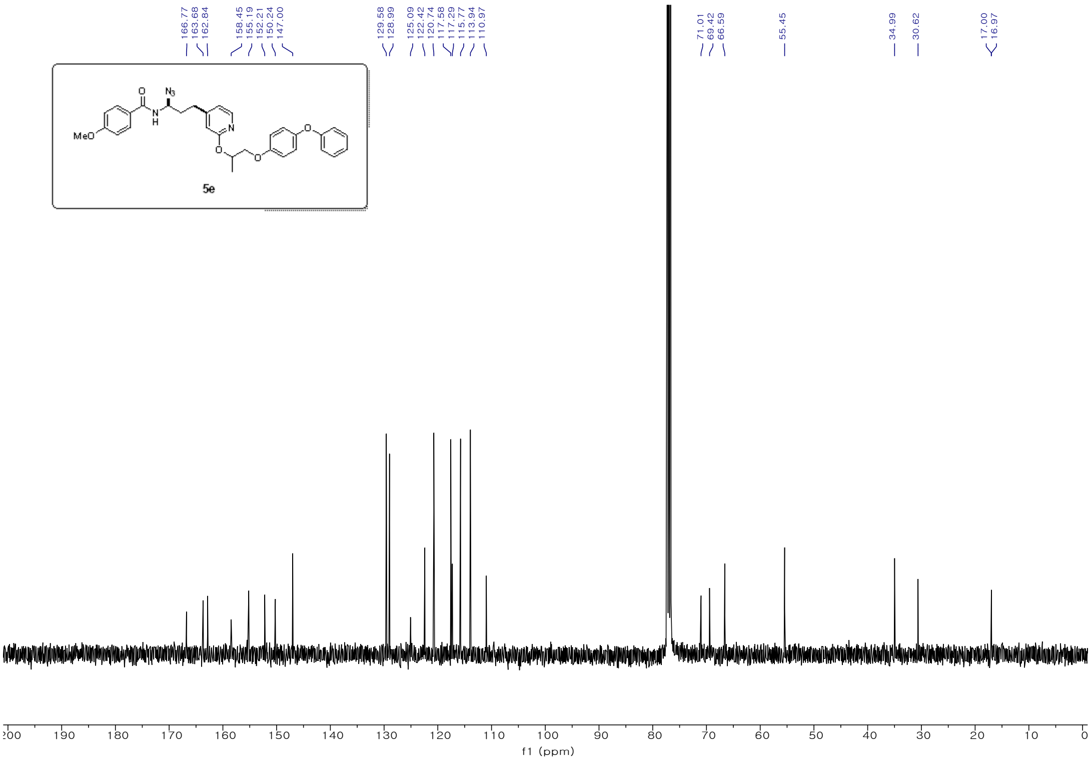

125 MHz, ^{13}C NMR in $CDCl_3$

((4-(3-azido-3-(4-methoxybenzamido)propyl)pyridin-2-yl)methylene)bis(4,1-phenylene) diacetate (5f)

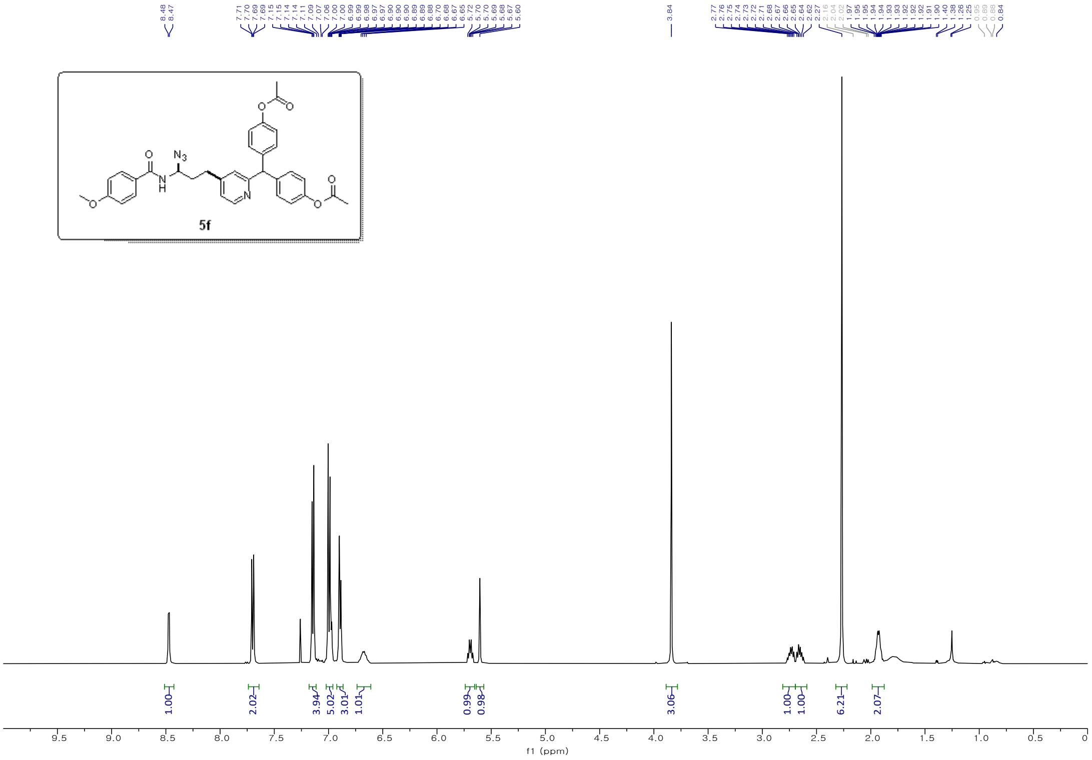

500 MHz, ^{1}H NMR in $CDCl_3$

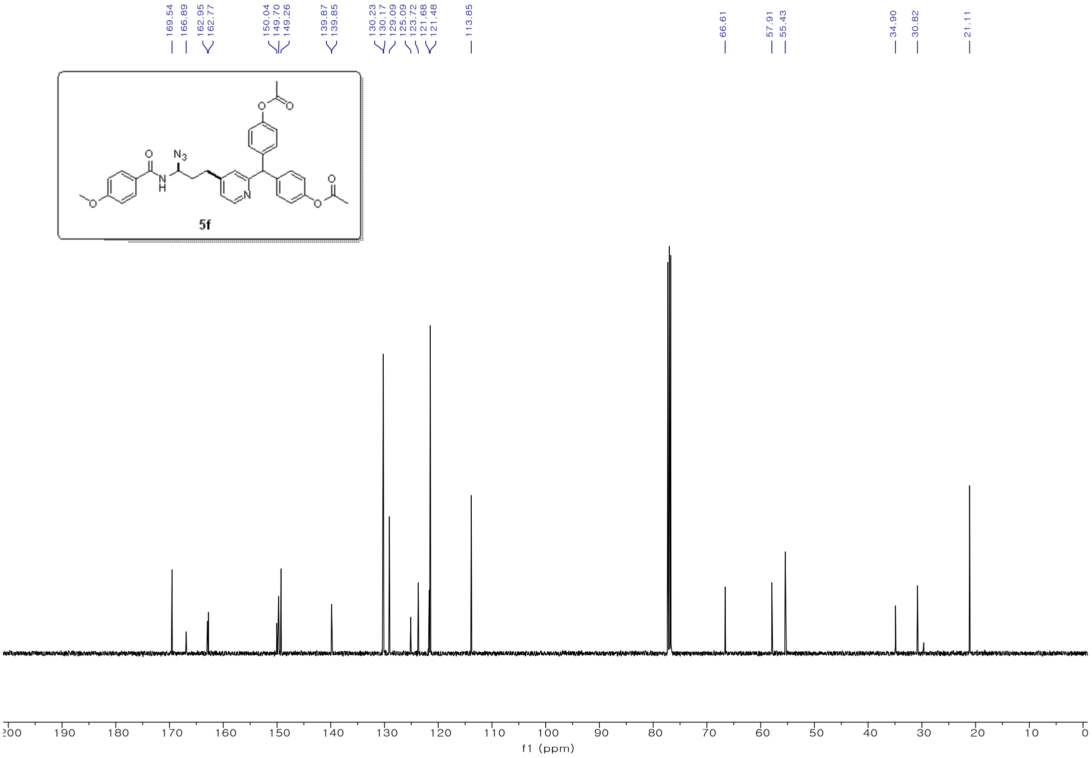

125 MHz, ^{13}C NMR in $CDCl_3$

N-(azido(2-phenylpyridin-4-yl)methyl)-2-(11-oxo-6,11-dihydrodibenzo[b,e]oxepin-2-yl)acetamide (5g).

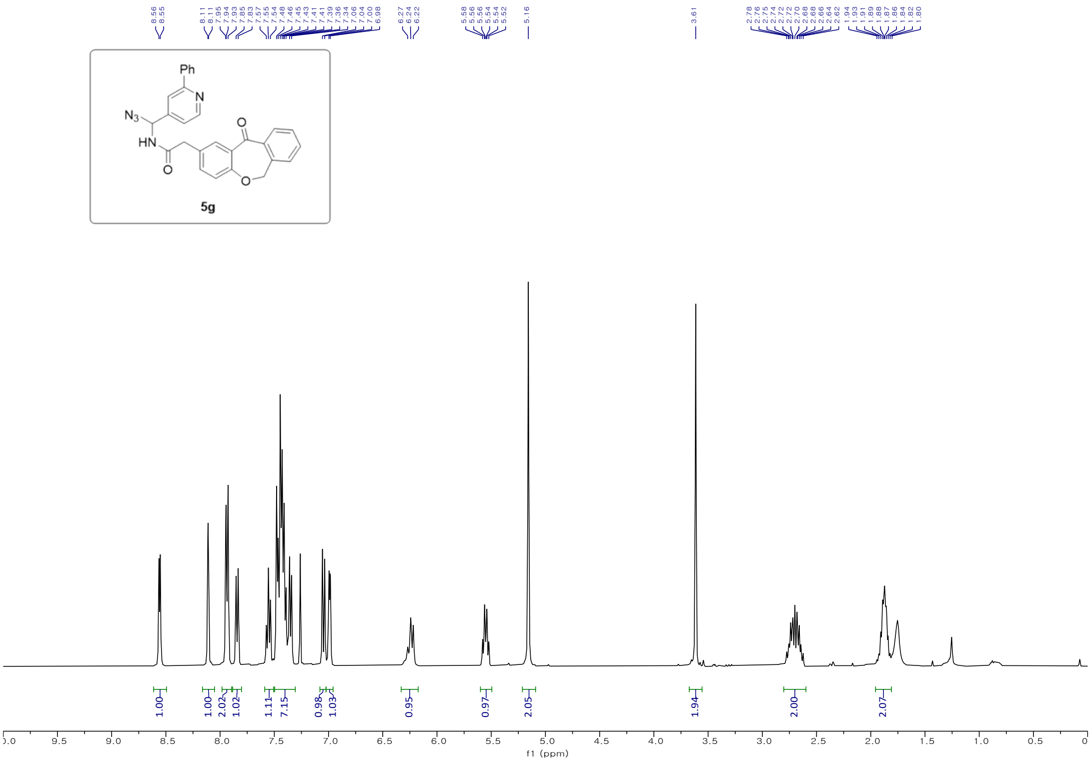

400 MHz, ^{1}H NMR in $CDCl_3$

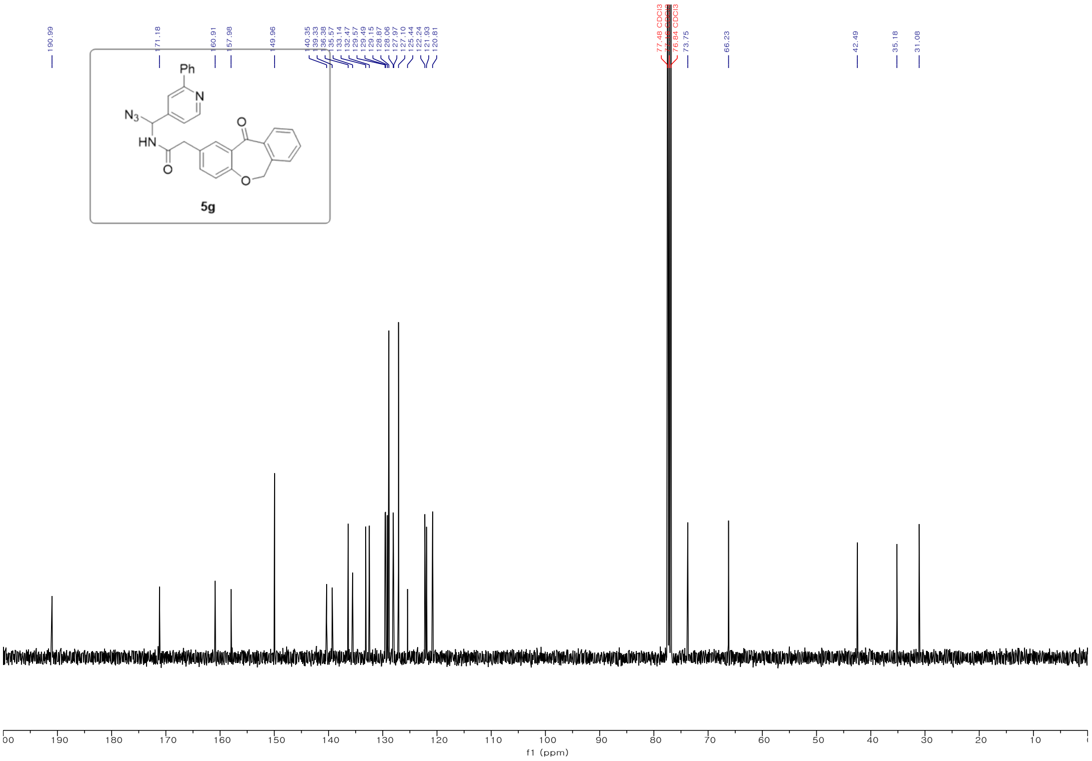

100 MHz, ^{13}C NMR in $CDCl_3$

4-methoxy-N-(5-(2-phenylpyridin-4-yl)pent-1-yn-3-yl)benzamide (6a).

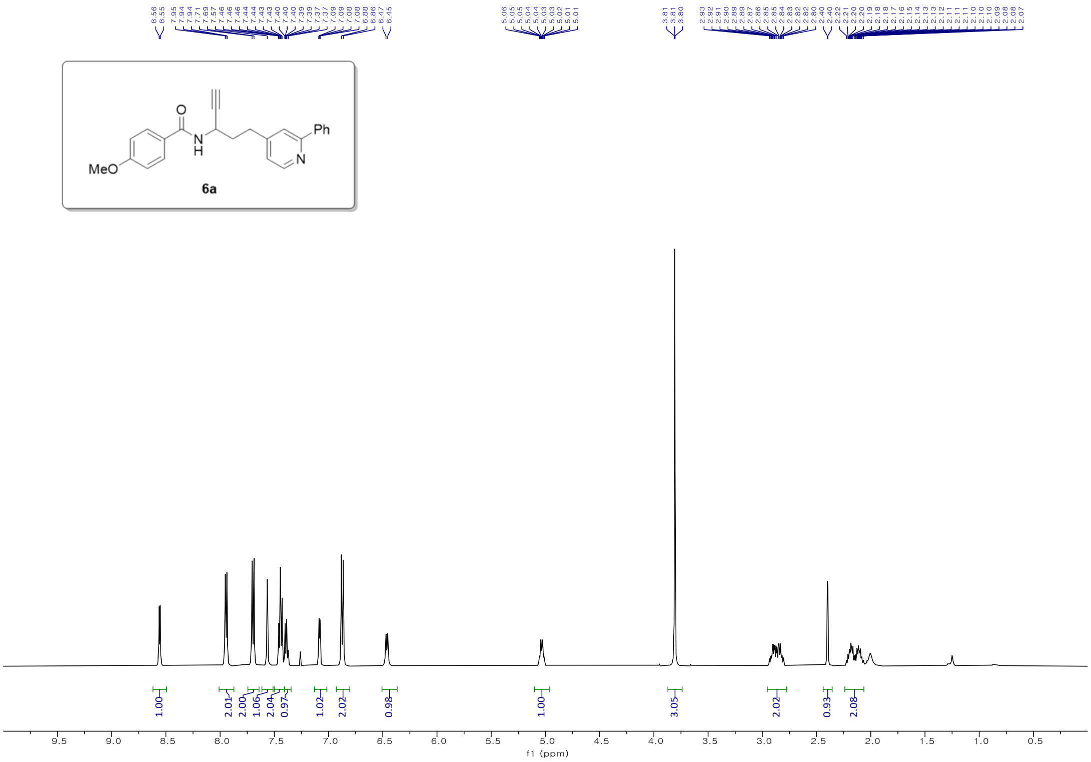

400 MHz, ^{1}H NMR in $CDCl_3$

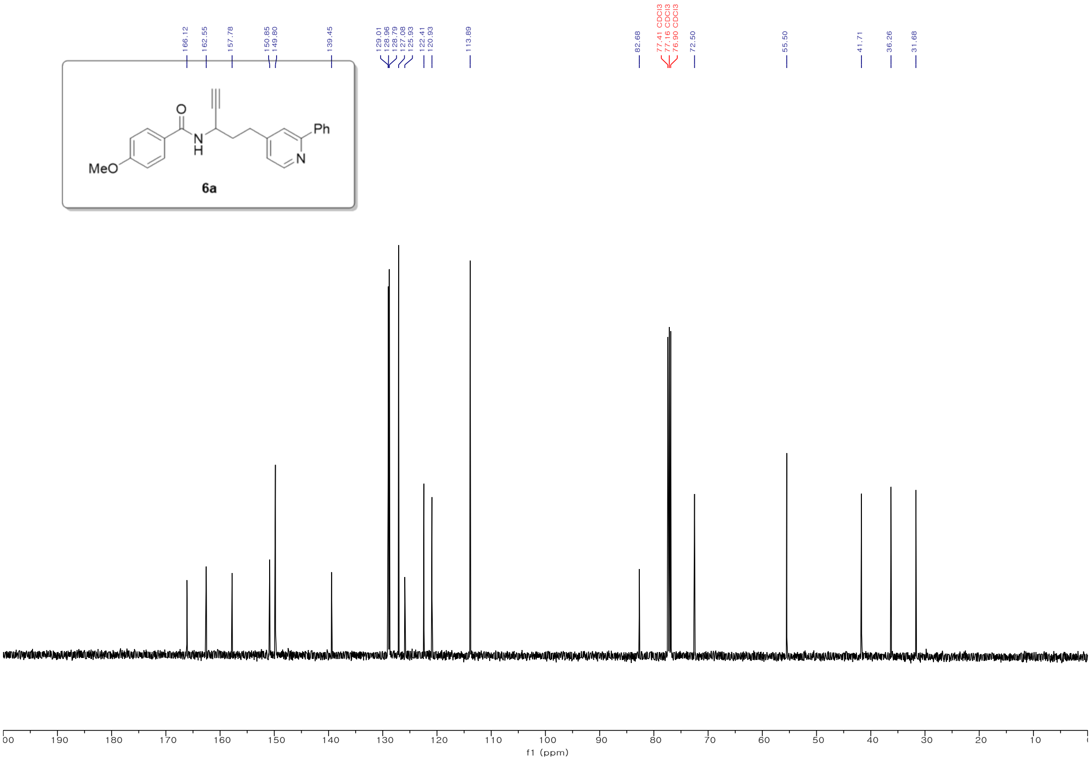

100 MHz, ^{13}C NMR in $CDCl_3$

4-methoxy-N-(1-(2-phenylpyridin-4-yl)pentan-3-yl)benzamide (6b).

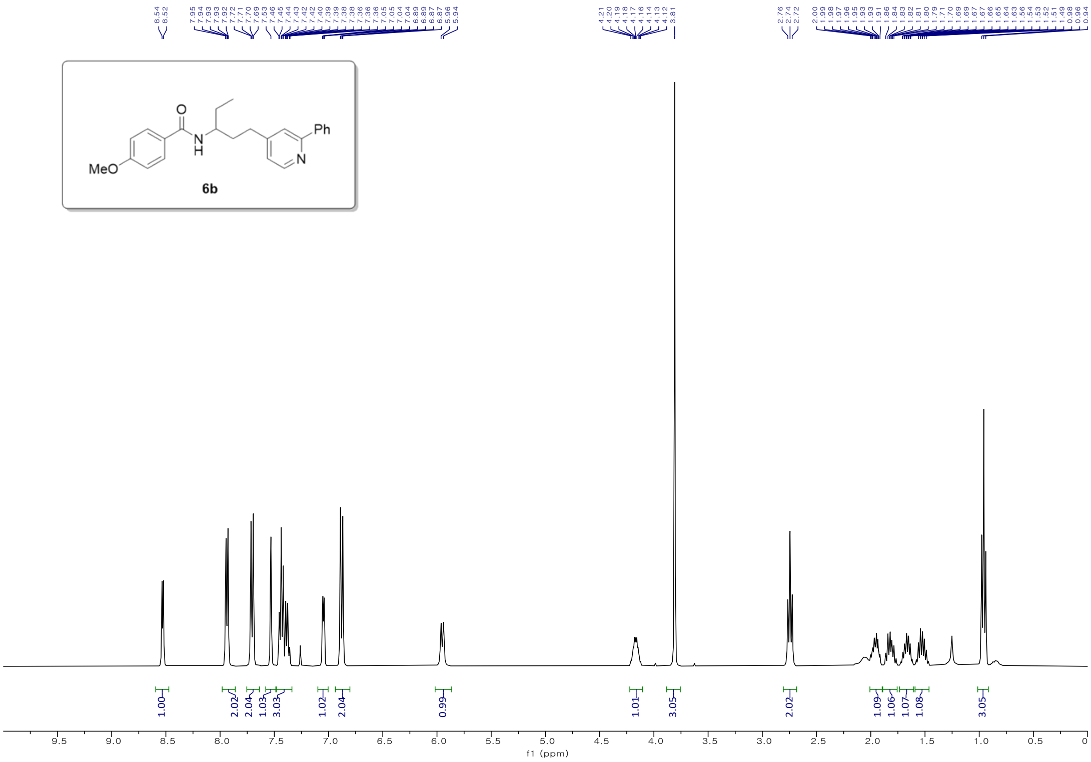

400 MHz, ^{1}H NMR in $CDCl_3$

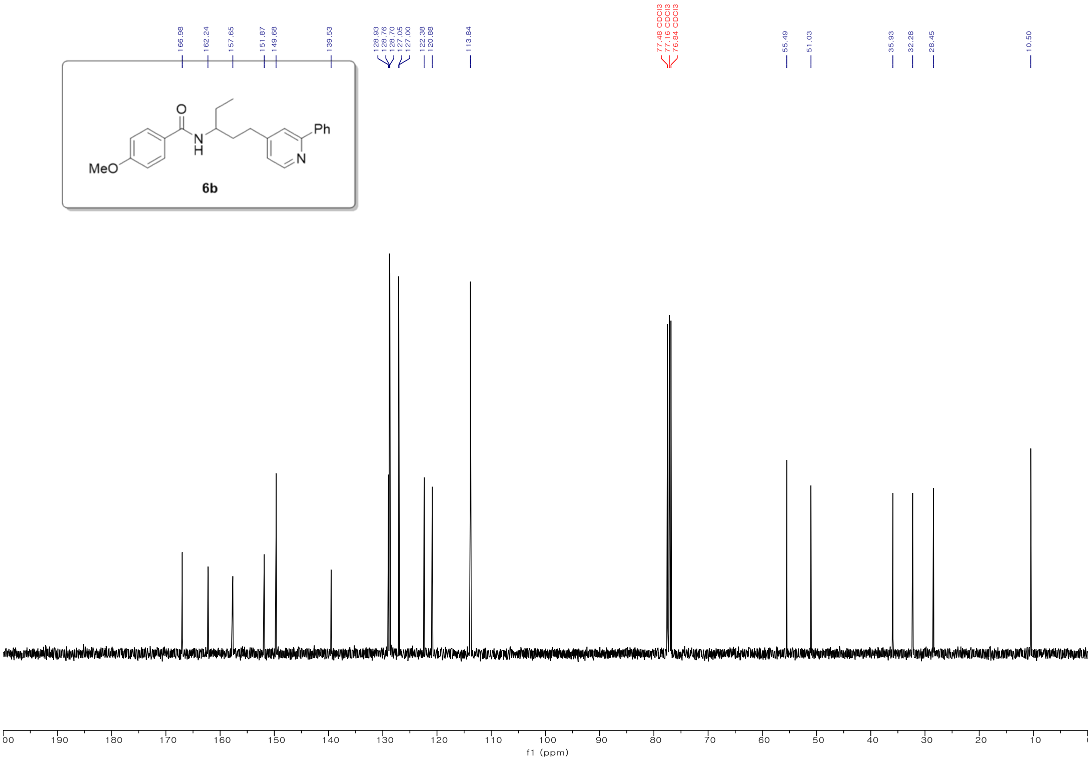

100 MHz, ^{13}C NMR in $CDCl_3$

N-(1-cyano-3-(2-phenylpyridin-4-yl)propyl)-4-methoxybenzamide (6c).

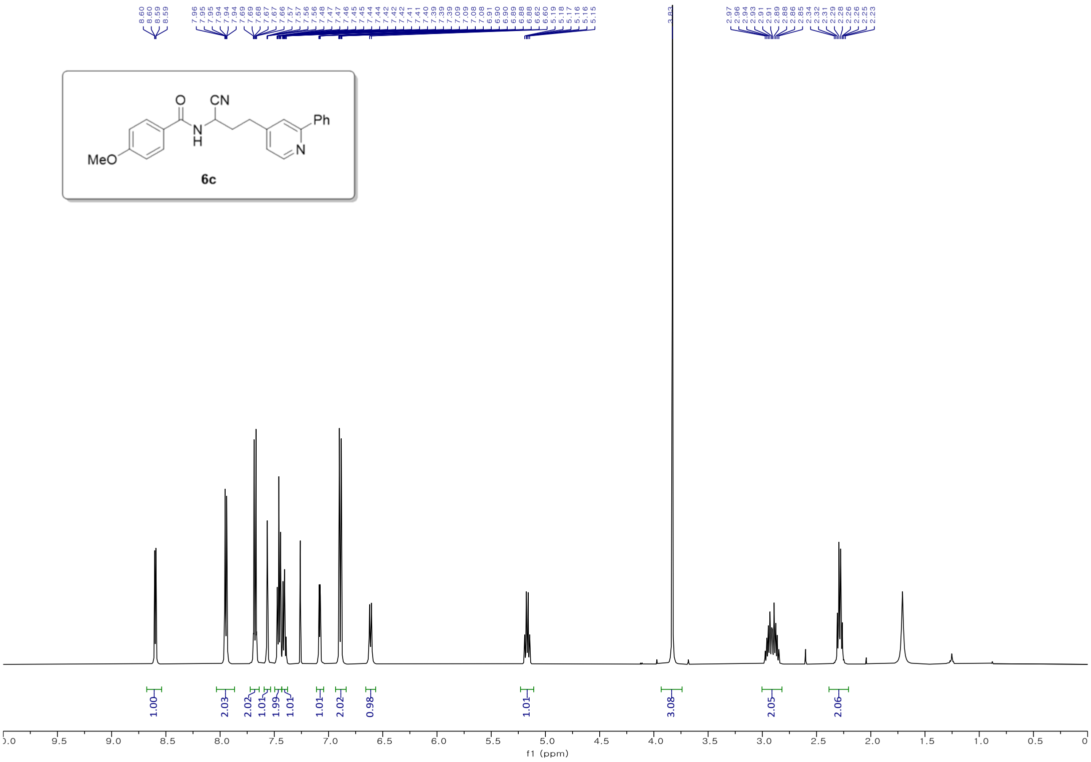

500 MHz, ^{1}H NMR in $CDCl_3$

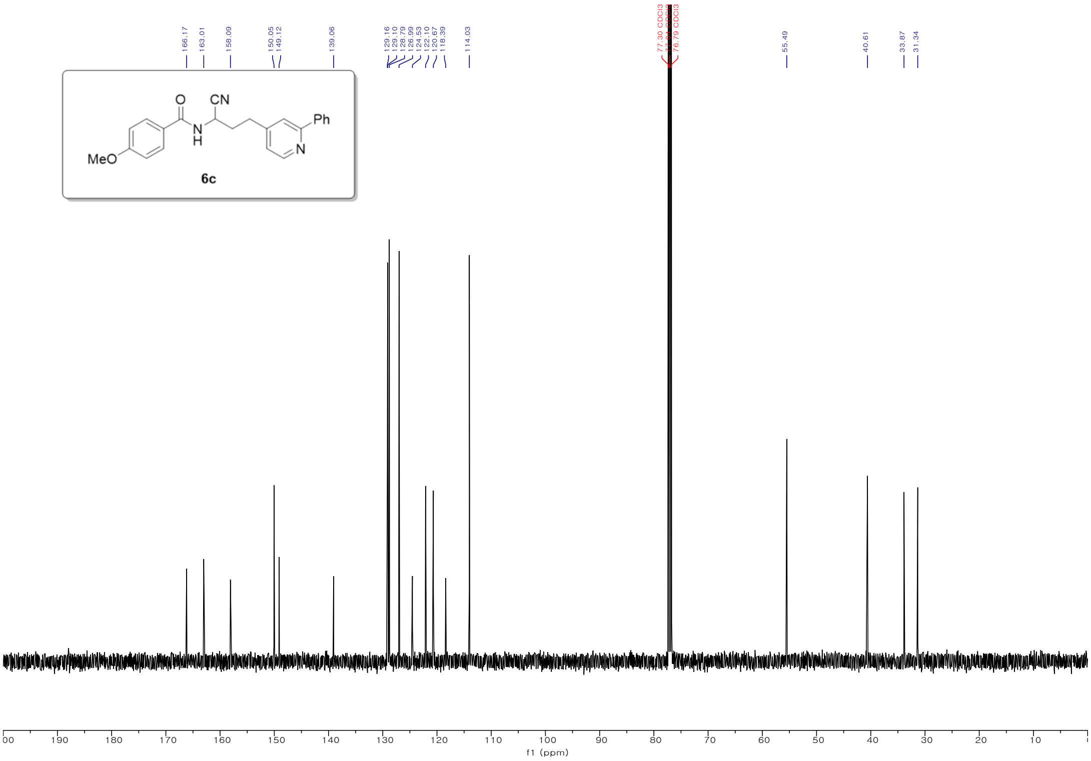

125 MHz, ^{13}C NMR in $CDCl_3$

diphenyl (1-(4-methoxybenzamido)-3-(2-phenylpyridin-4-yl)propyl)phosphonate (6d).

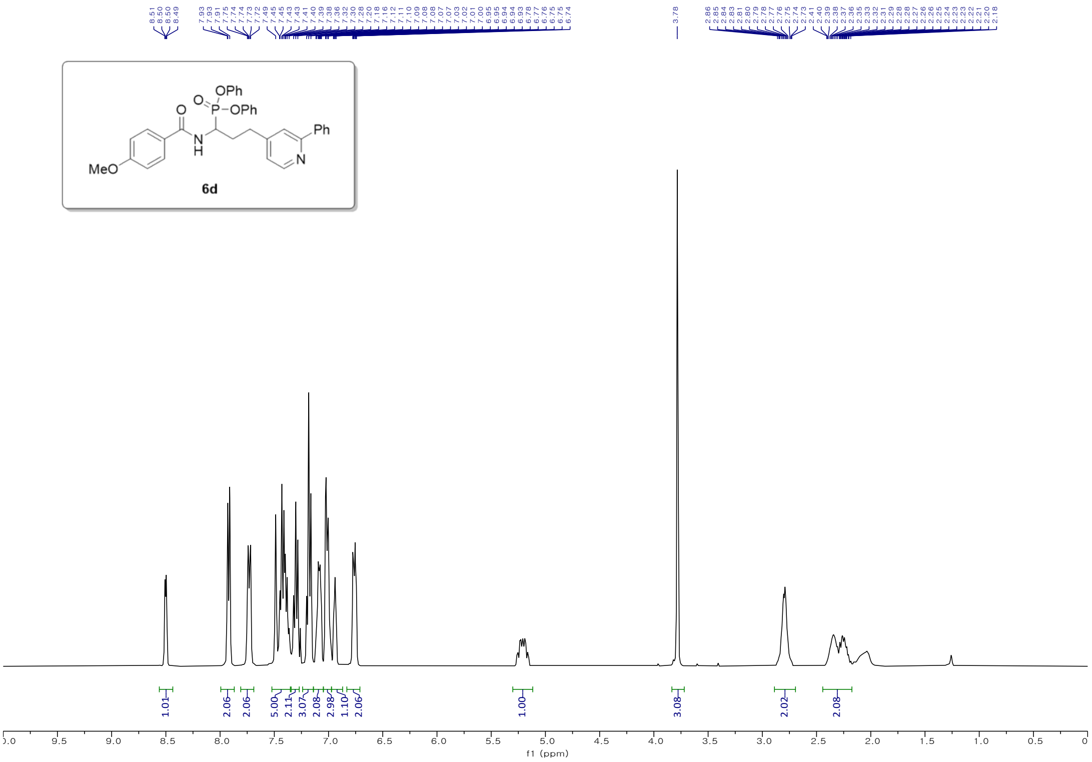

400 MHz, ^{1}H NMR in CDCl$_3$

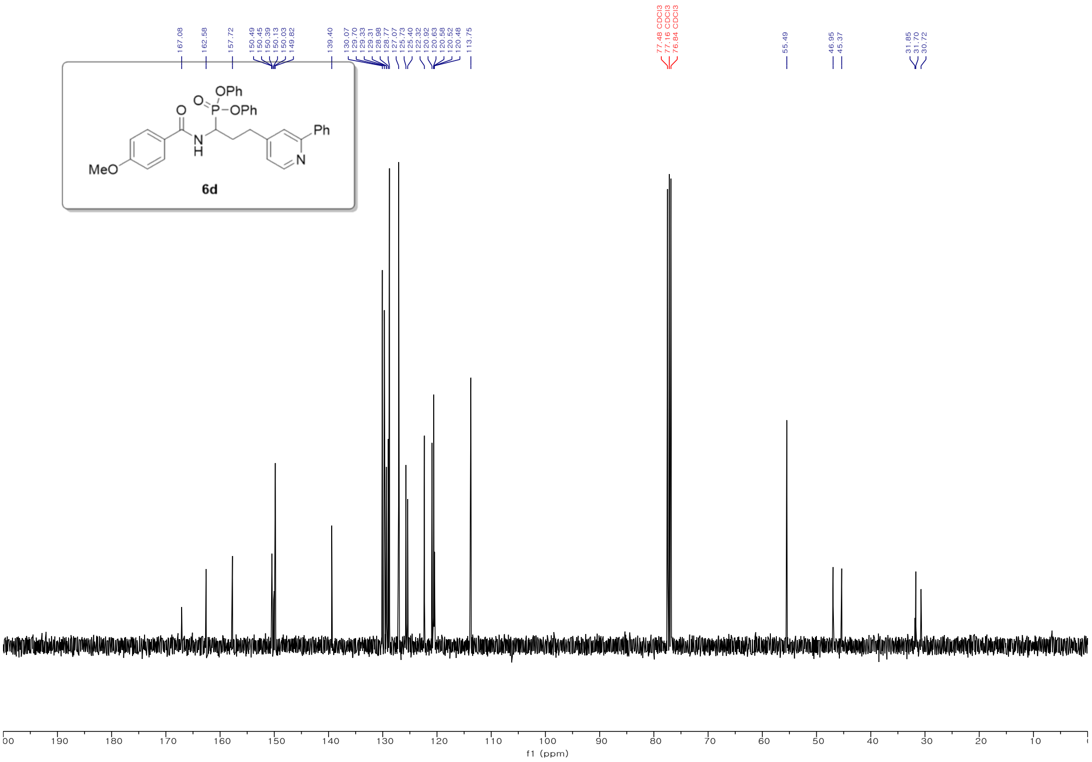

100 MHz, ^{13}C NMR in CDCl$_3$

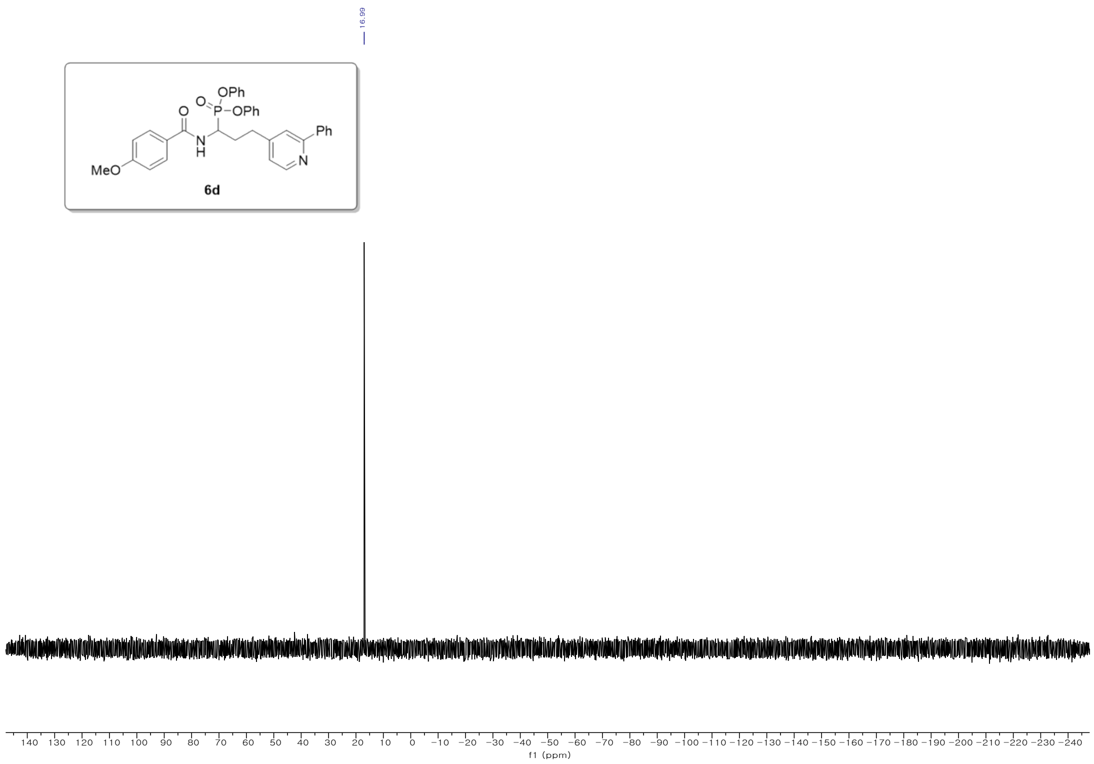

162 MHz, ^{31}P NMR in $CDCl_3$

4-methoxy-N-(1-(1-methyl-1H-indol-3-yl)-3-(2-phenylpyridin-4-yl)propyl)benzamide (6e).

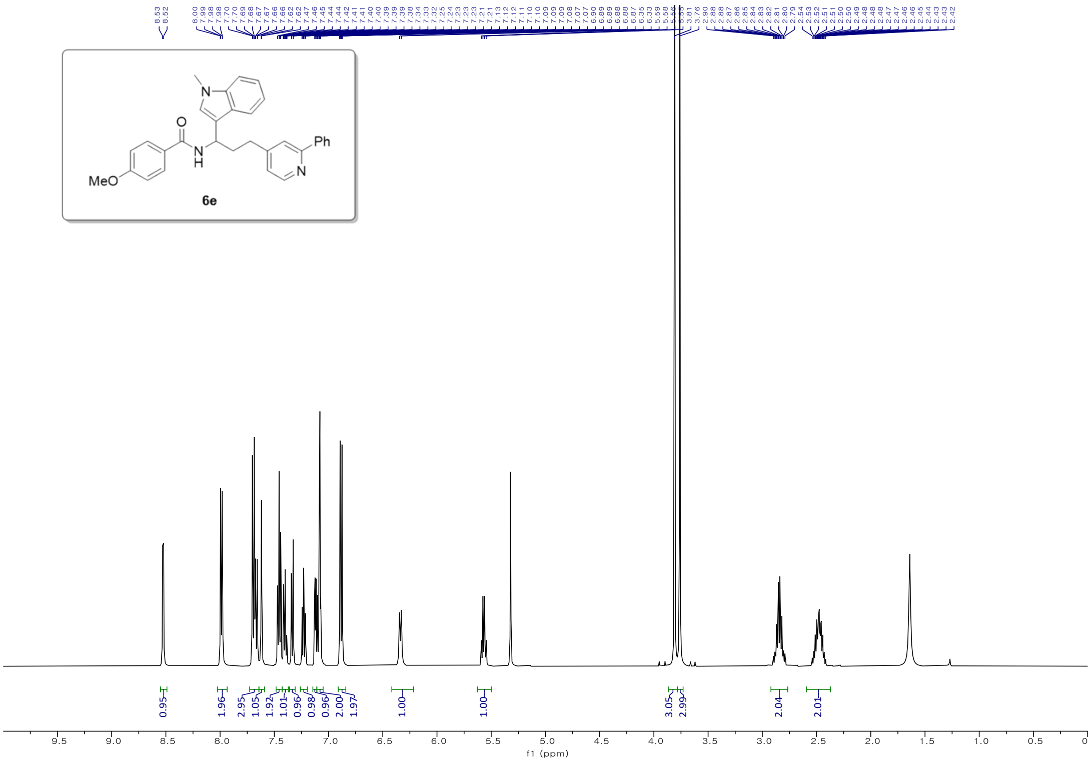

500 MHz, ^{1}H NMR in CD_2Cl_2

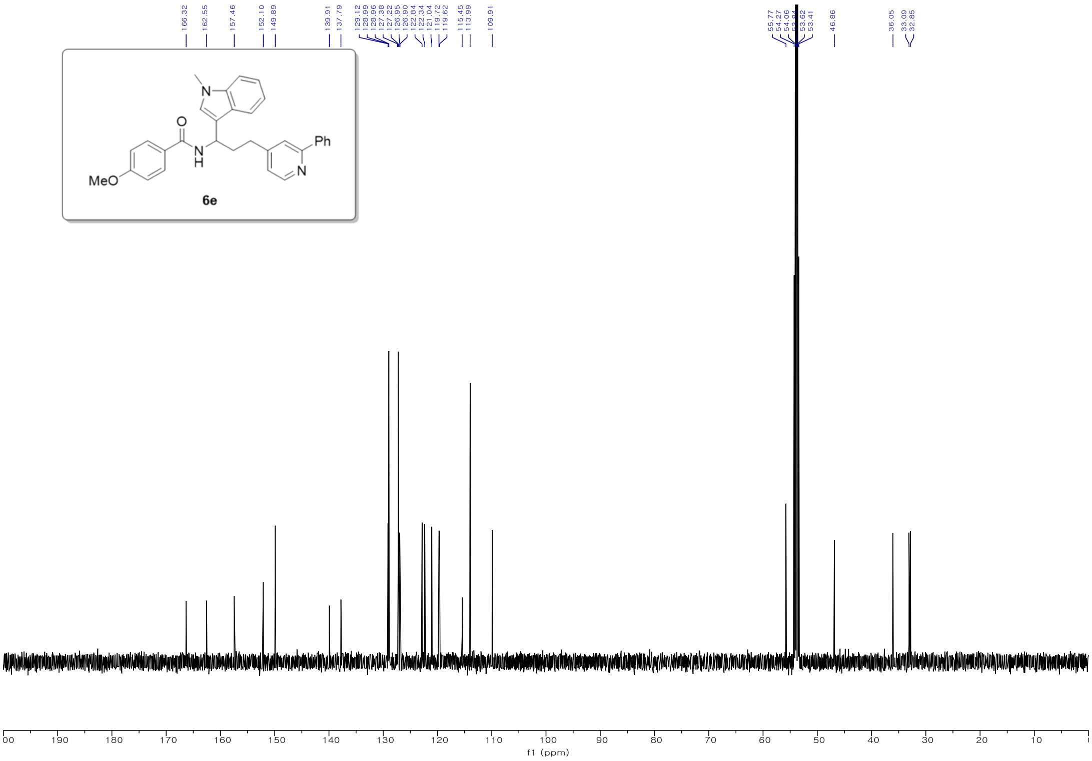

125 MHz, ^{13}C NMR in CD_2Cl_2

methyl 3-(4-methoxybenzamido)-2,2-dimethyl-5-(2-phenylpyridin-4-yl)pentanoate (6f).

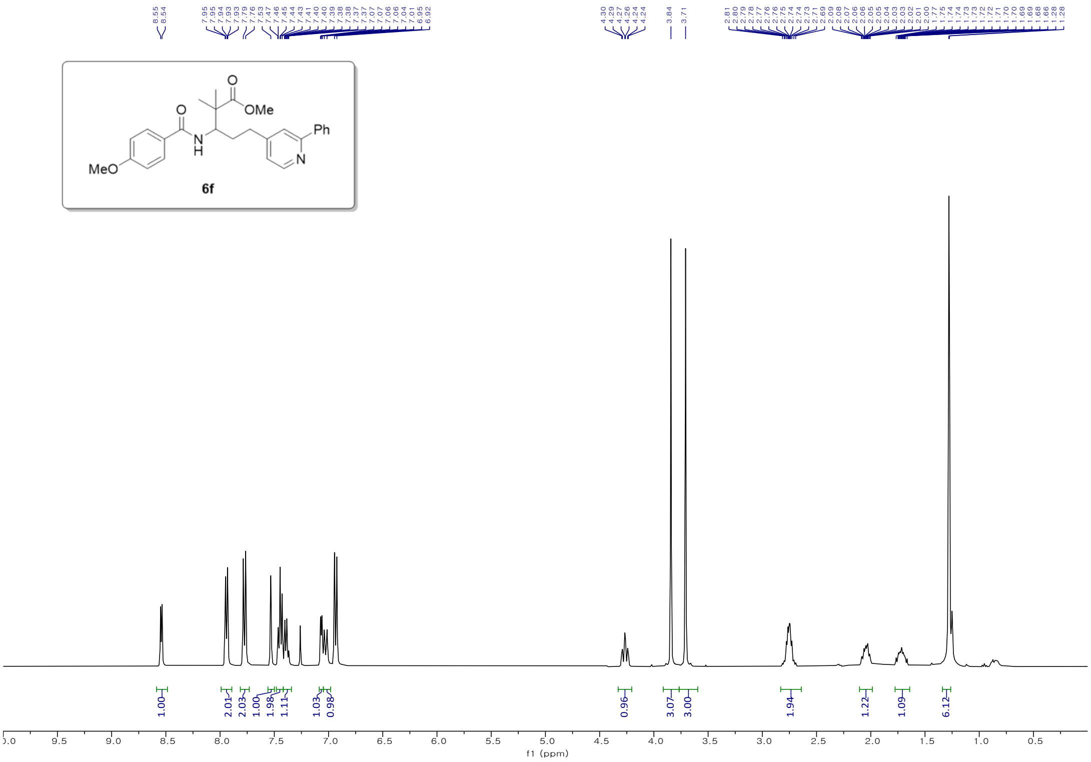

400 MHz, ^{1}H NMR in $CDCl_3$

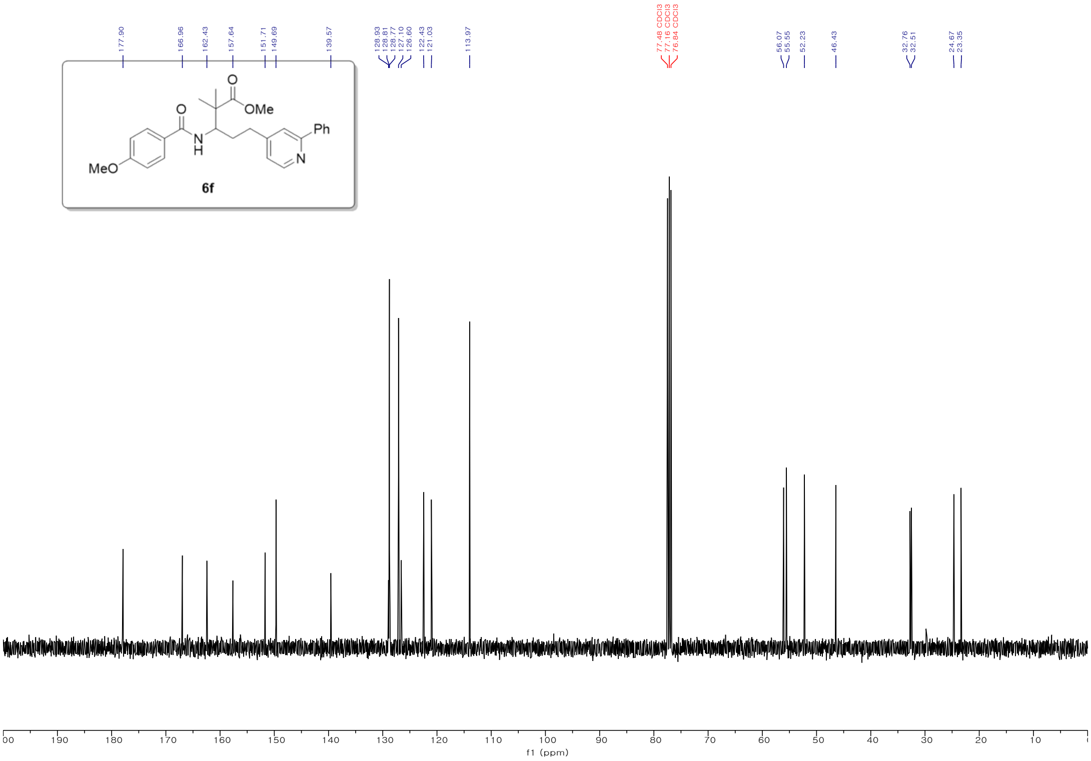

100 MHz, ^{13}C NMR in $CDCl_3$

4-methoxy-N-(1-oxo-5-(2-phenylpyridin-4-yl)pentan-3-yl)benzamide (6g).

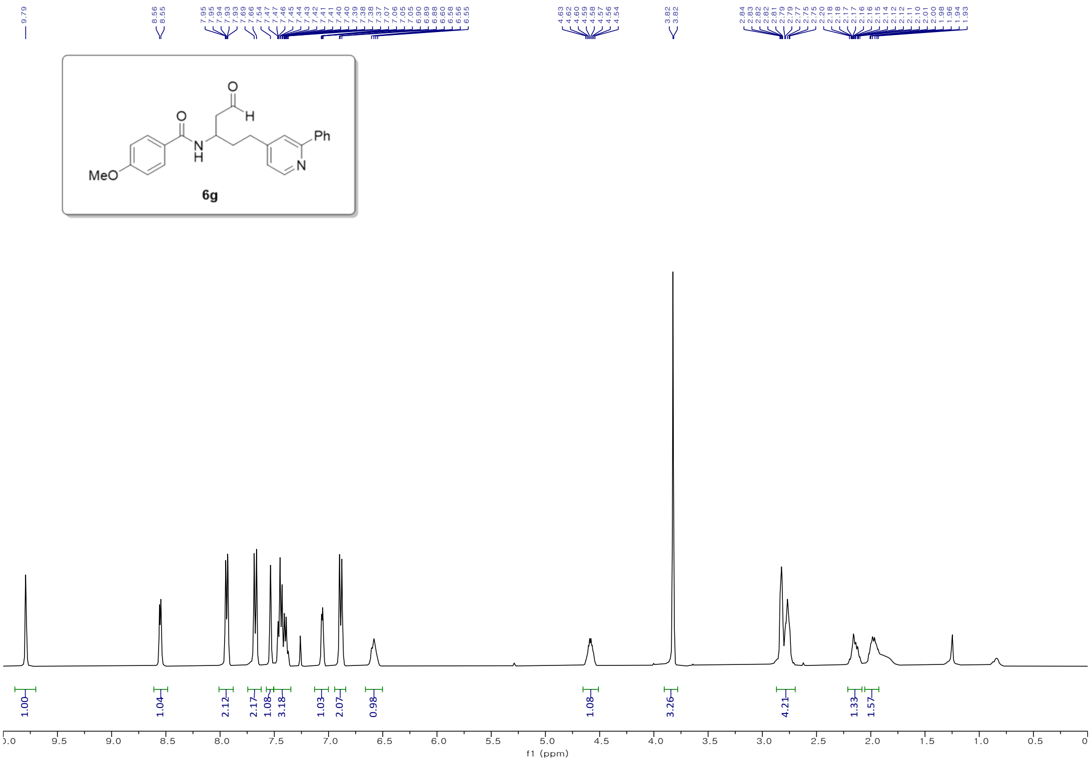

400 MHz, ^{1}H NMR in $CDCl_3$

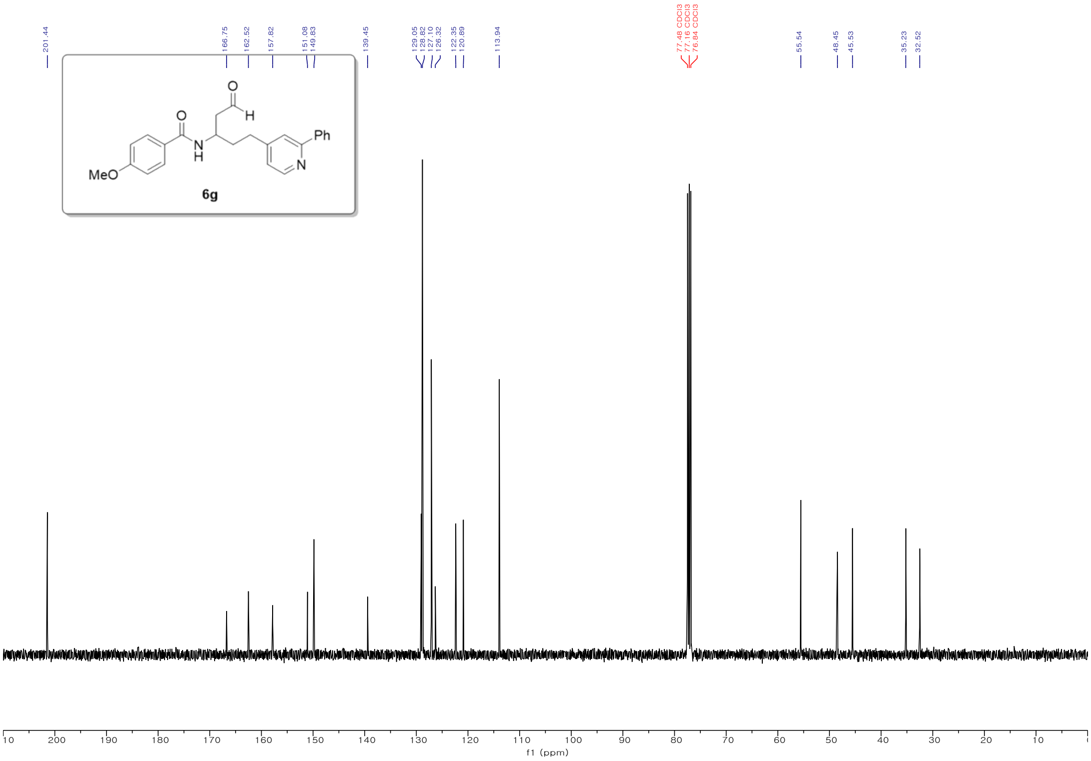

100 MHz, ^{13}C NMR in $CDCl_3$

(E)-4-methoxy-N-(1-(2-phenylpyridin-4-yl)hex-4-en-3-yl)benzamide (6h).

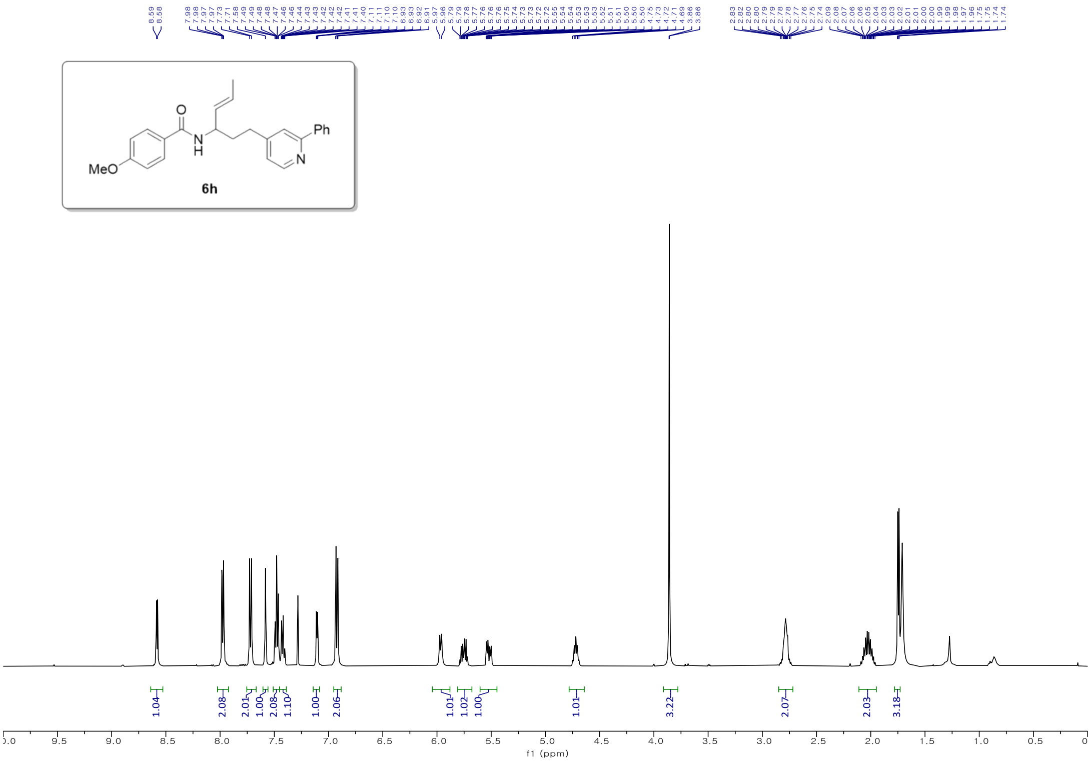

500 MHz, ^{1}H NMR in $CDCl_3$

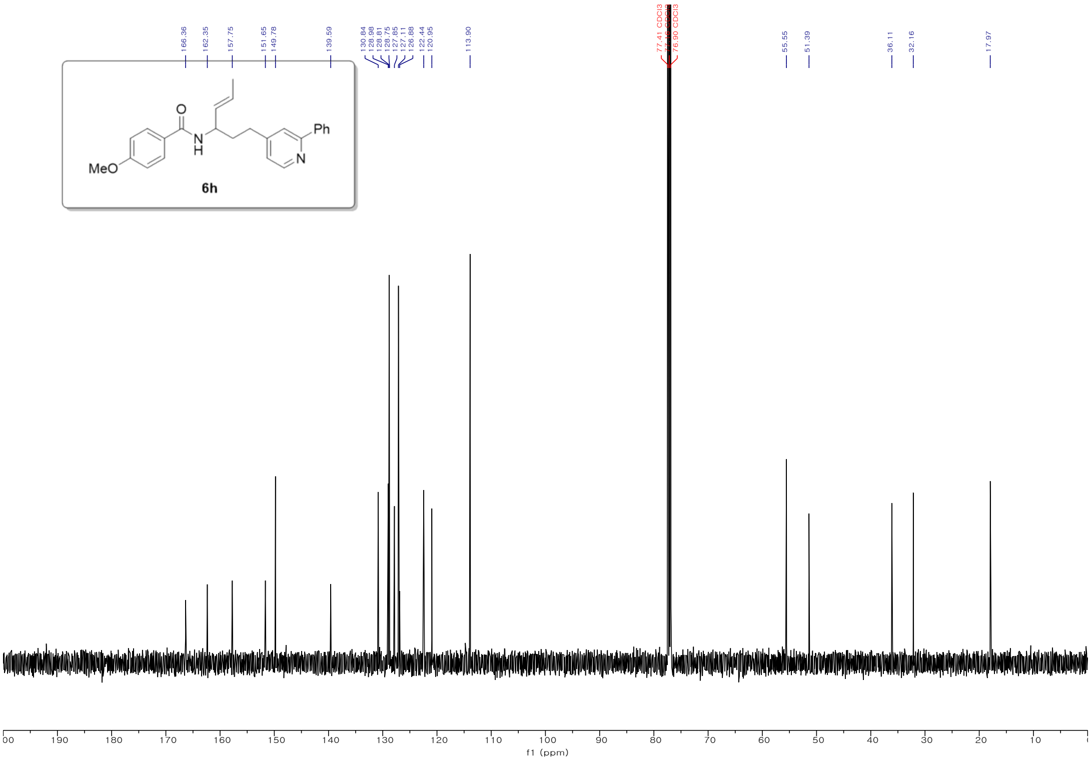

125 MHz, ^{13}C NMR in $CDCl_3$

N-(1-(((3s,5s,7s)-adamantan-1-yl)thio)-3-(2-phenylpyridin-4-yl)propyl)-4-methoxybenzamide (6i).

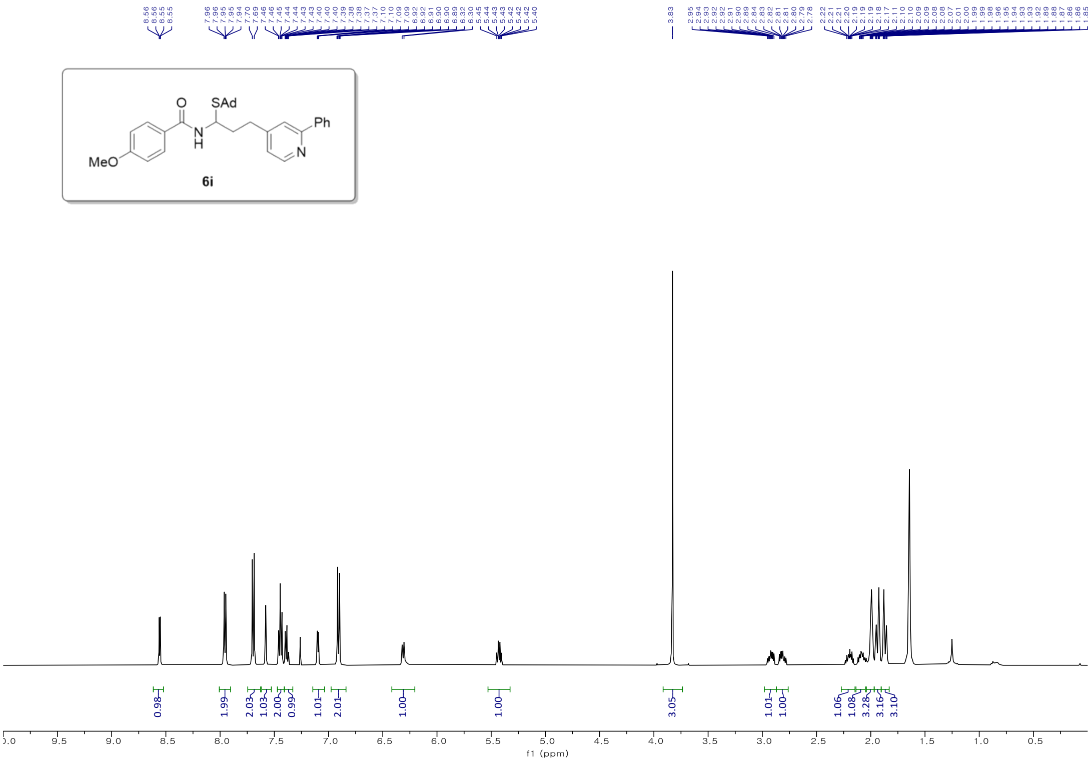

500 MHz, ^{1}H NMR in CDCl$_3$

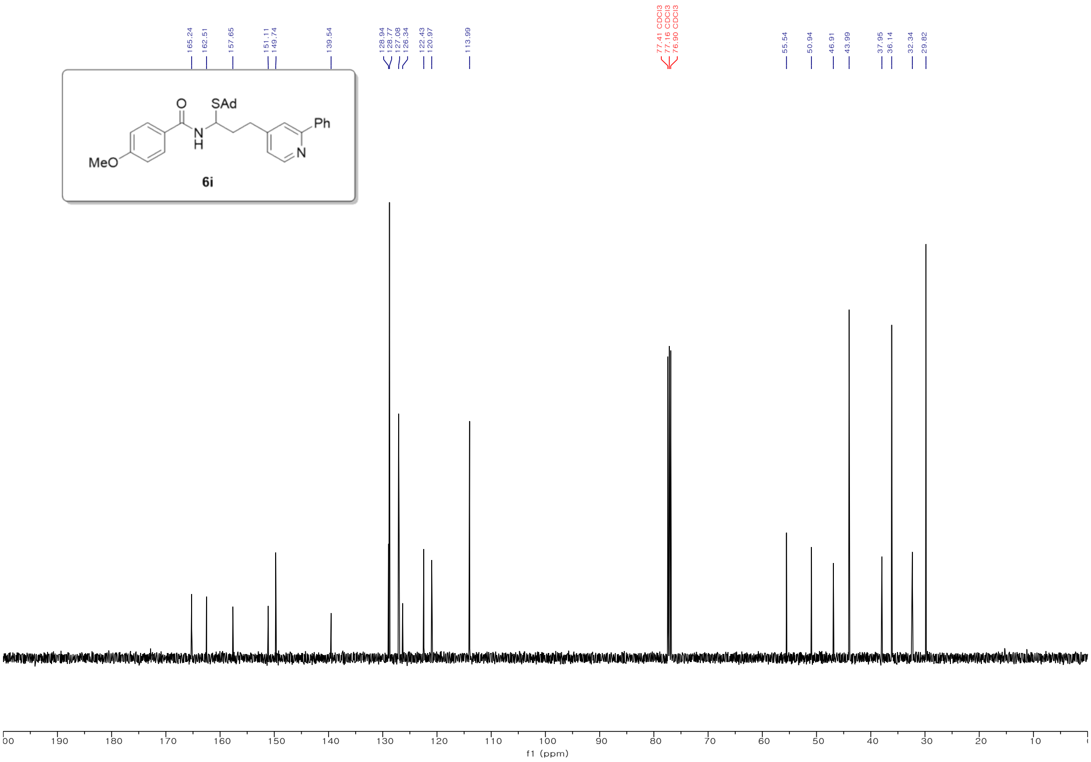

125 MHz, ^{13}C NMR in CDCl$_3$

methyl N-(acetyl-L-phenylalanyl)-S-(1-(4-methoxybenzamido)-3-(2-phenylpyridin-4-yl)propyl)-L-cysteinate (6j).

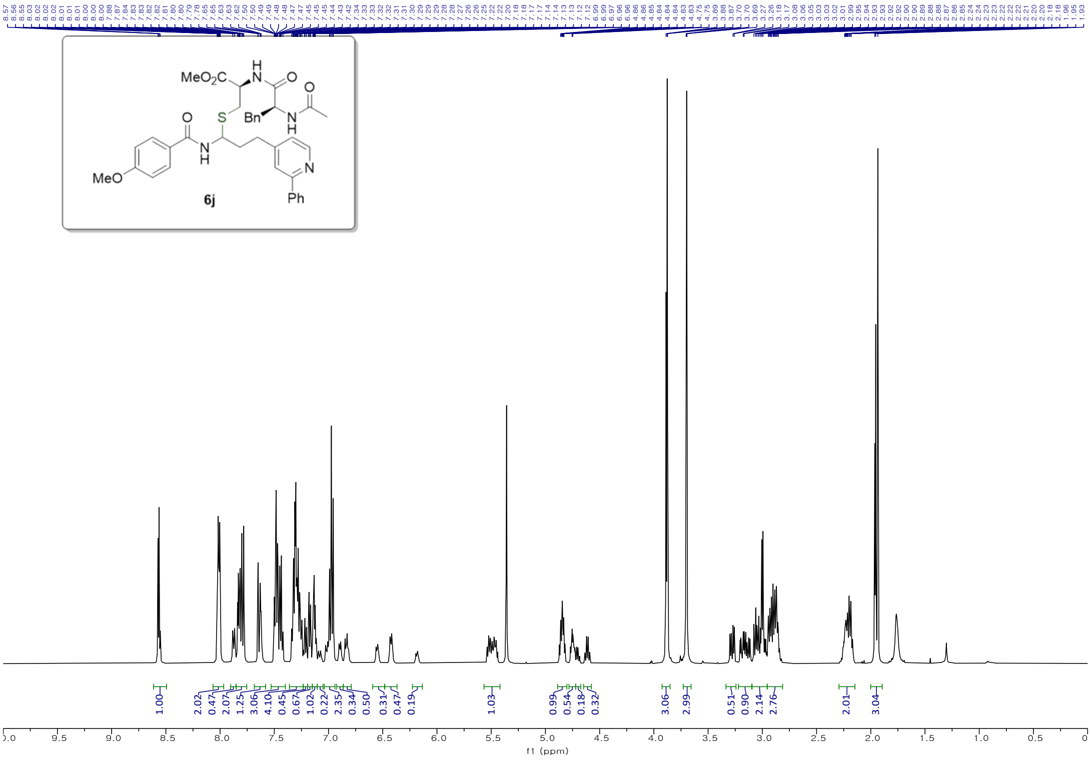

500 MHz, ^{1}H NMR in CD_2Cl_2

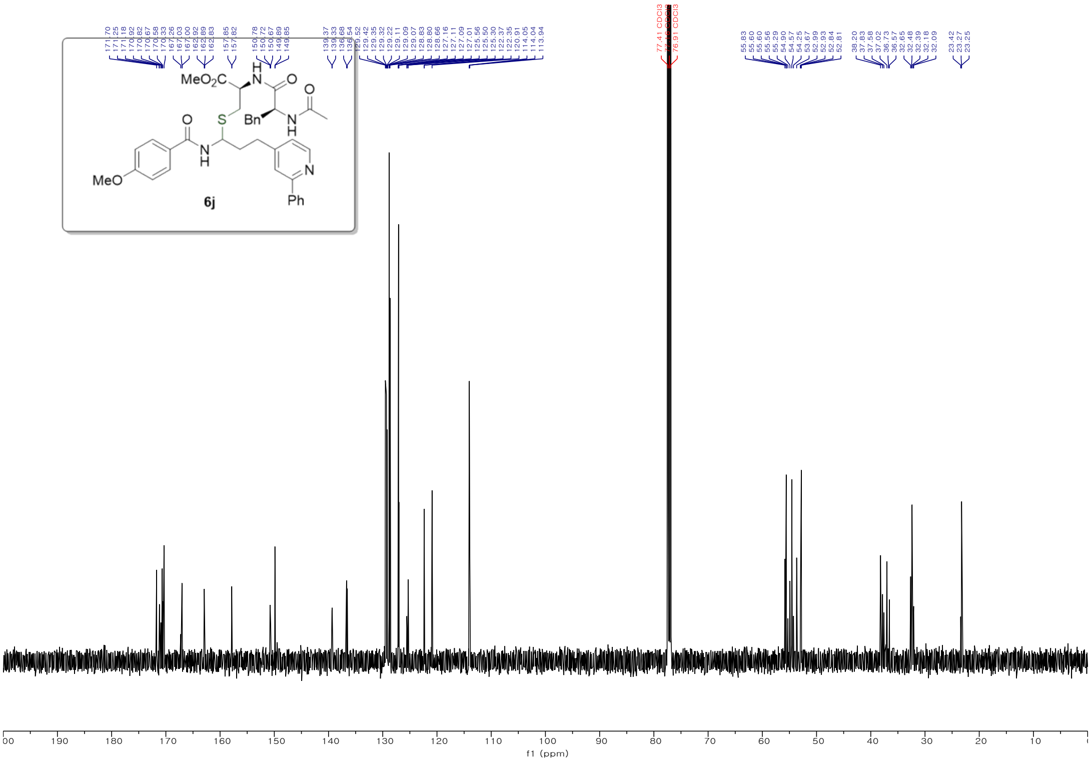

125 MHz, ^{13}C NMR in $CDCl_3$

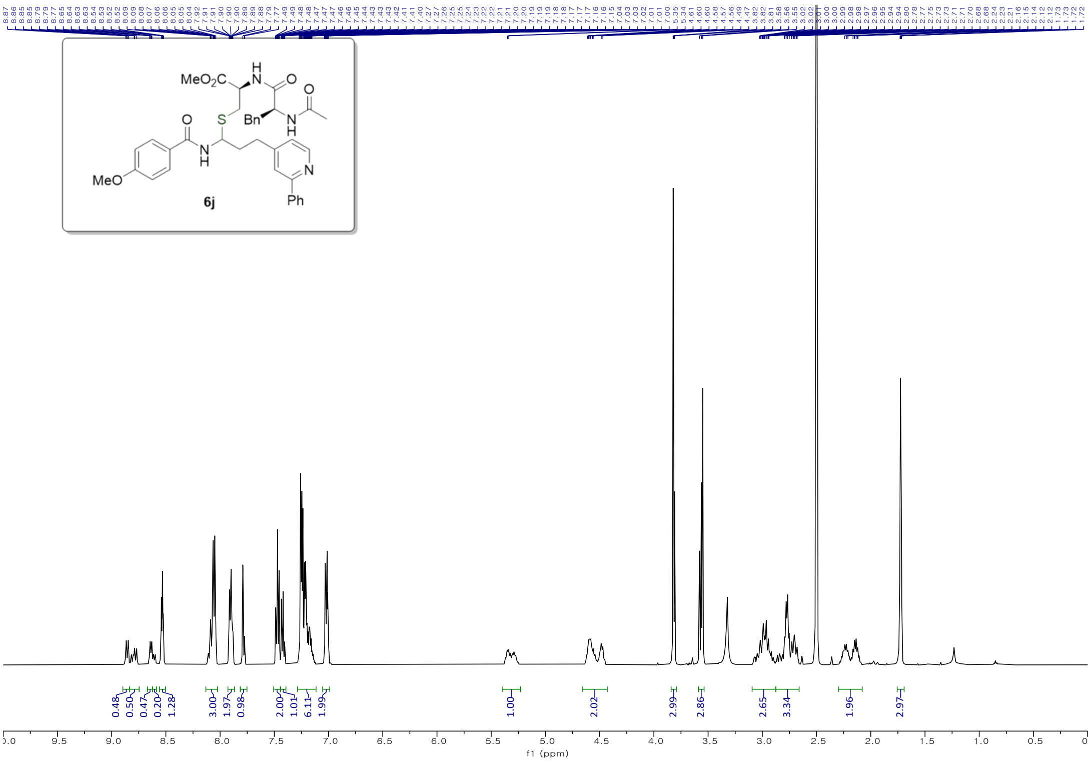

500 MHz, ^{1}H NMR in C_2D_6SO, 298 K

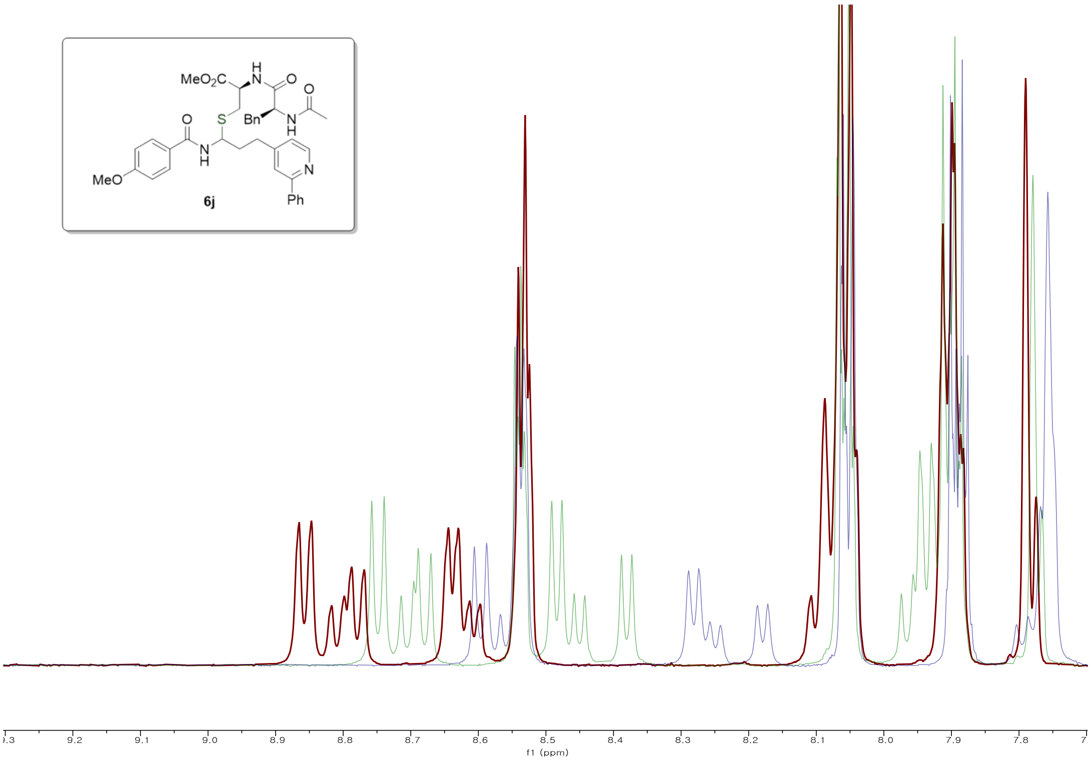

500 MHz, ^{1}H NMR in C_2D_6SO, 298 K, 323 K, 353 K (Red, Green, Blue)

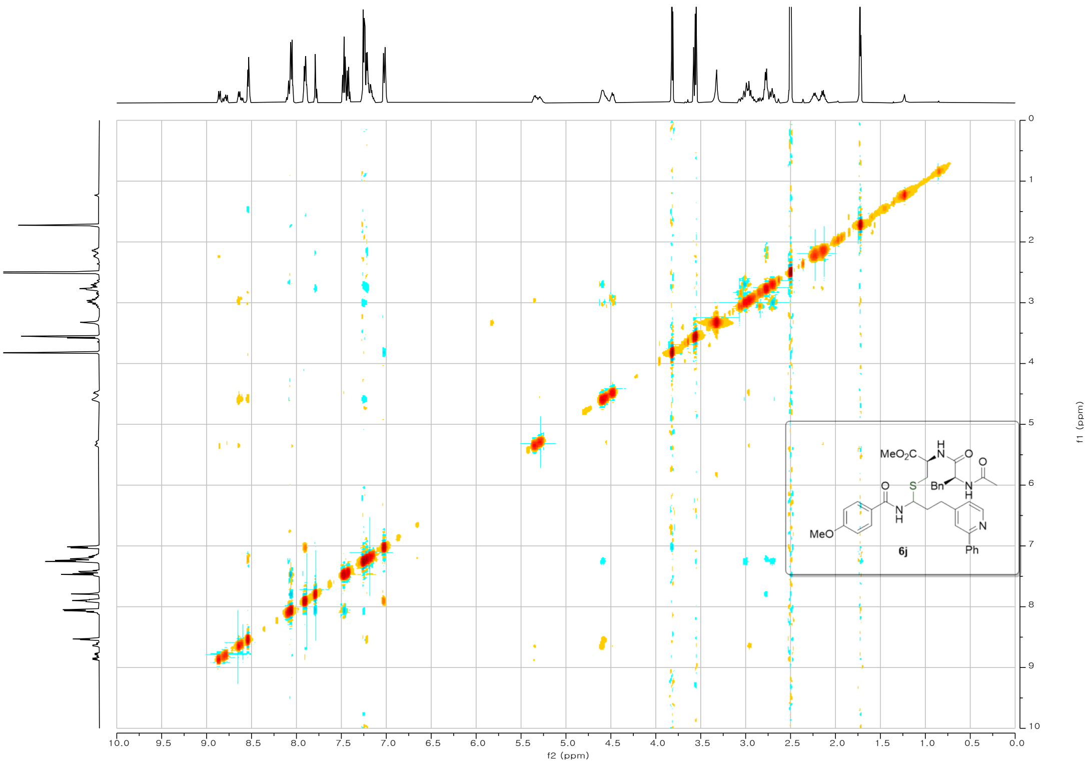

500 MHz, NOESY in C_2D_6SO, 298 K

tert-butyl (1-(4-methoxybenzamido)-3-(2-phenylpyridin-4-yl)propyl)carbamate (6k).

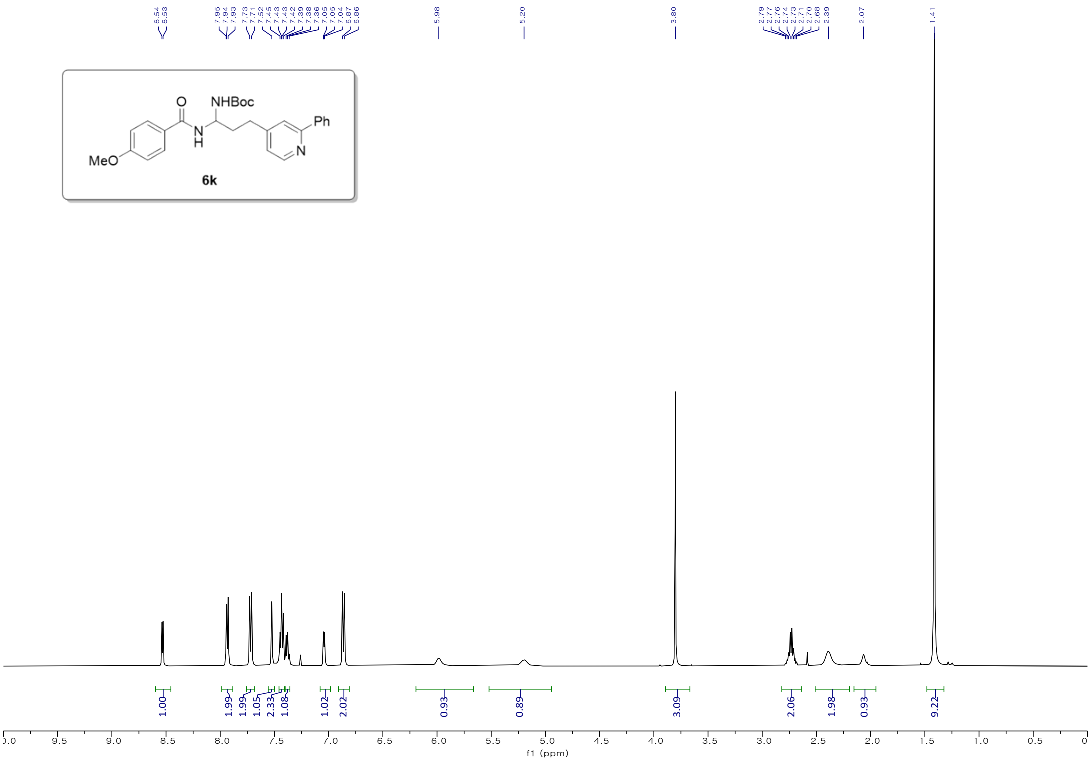

500 MHz, ^{1}H NMR in $CDCl_3$

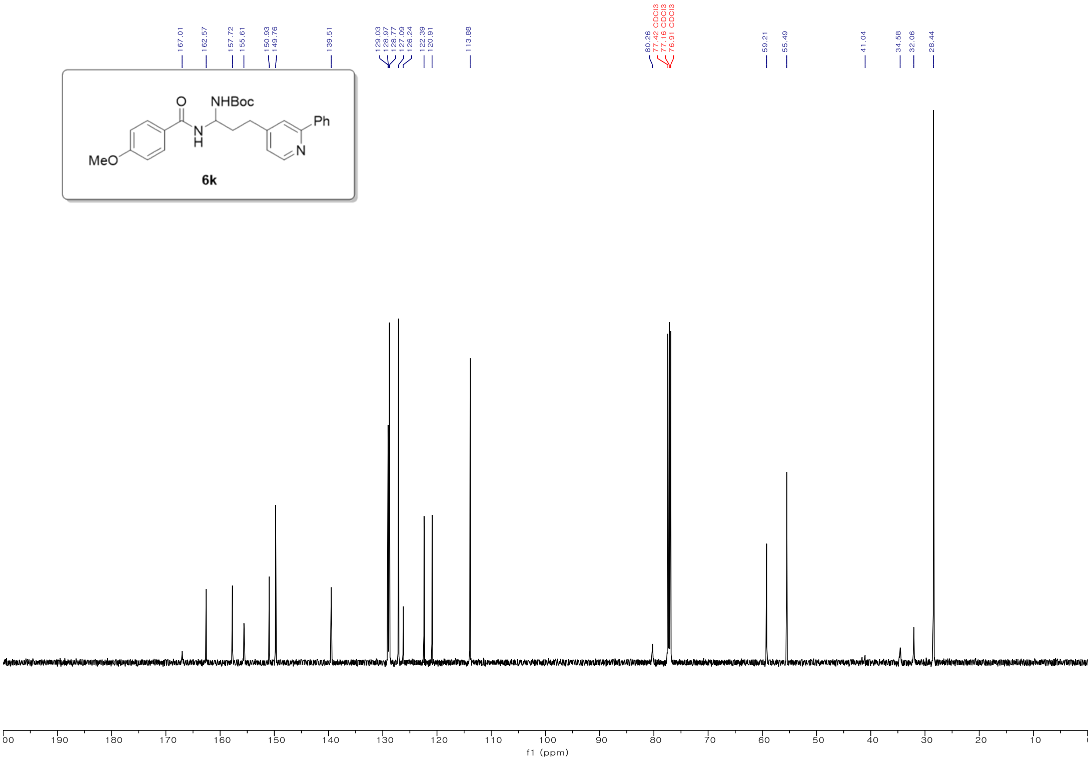

125 MHz, ^{13}C NMR in $CDCl_3$

methyl acetyl-L-phenylalanyl-L-cysteinate (6ja).

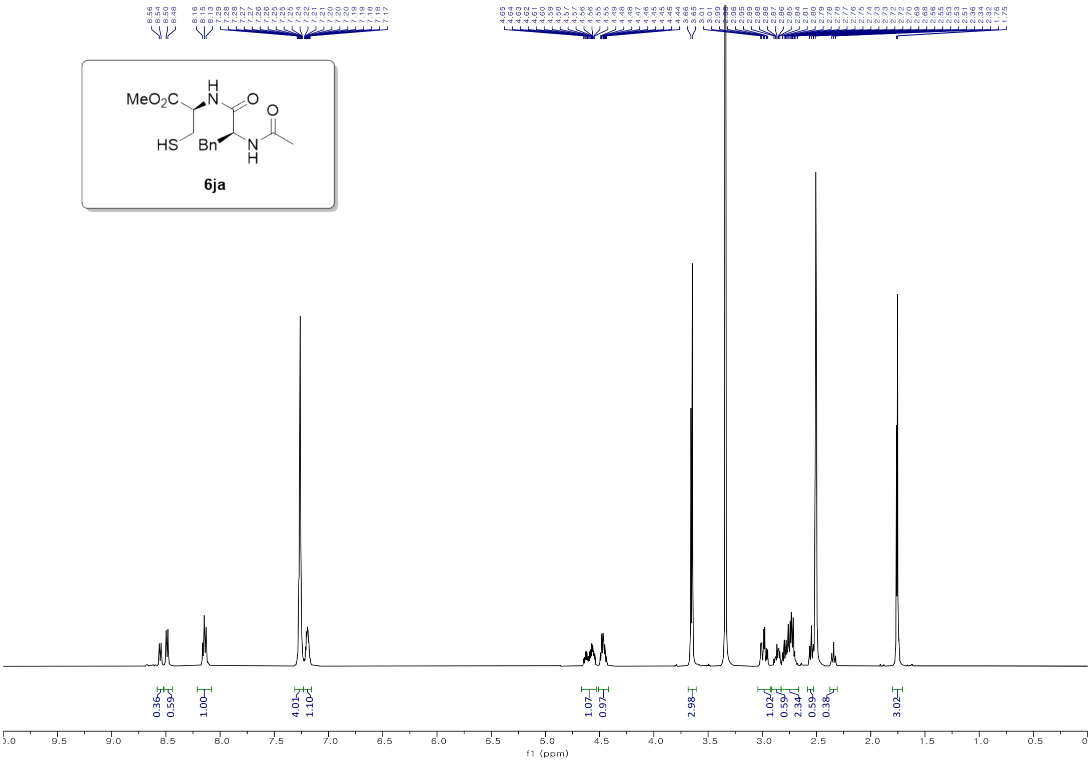

500 MHz, ¹H NMR in C2D6SO

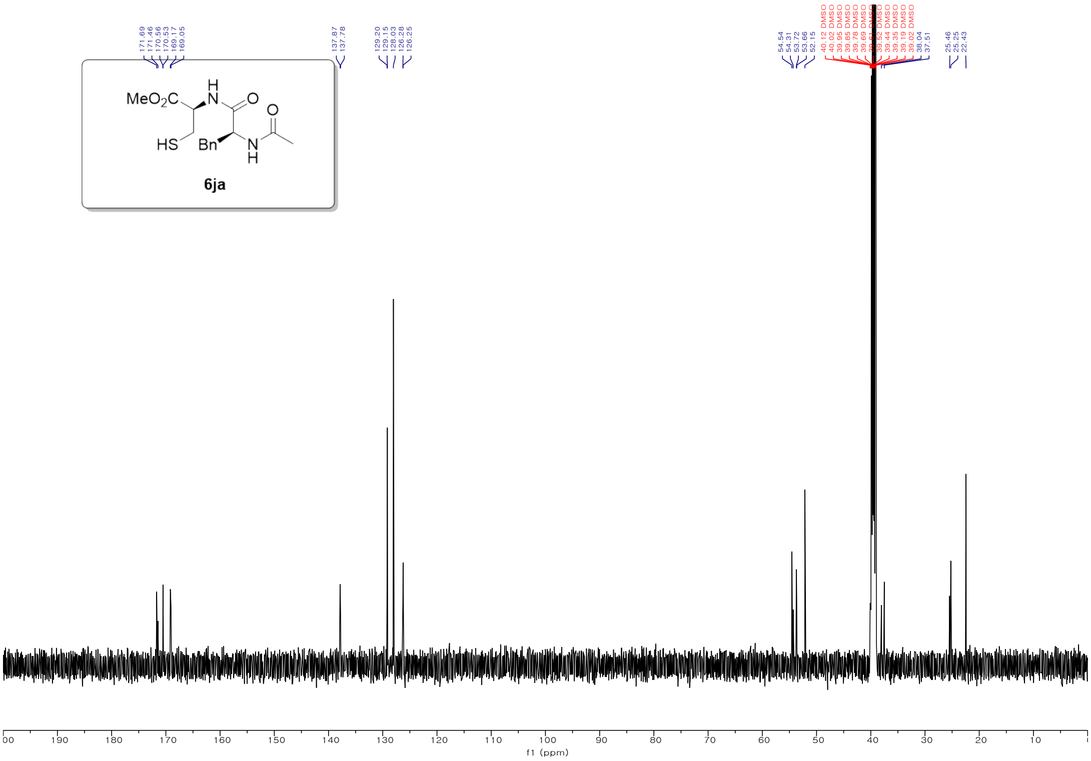

125 MHz, ¹³C NMR in C2D6SO

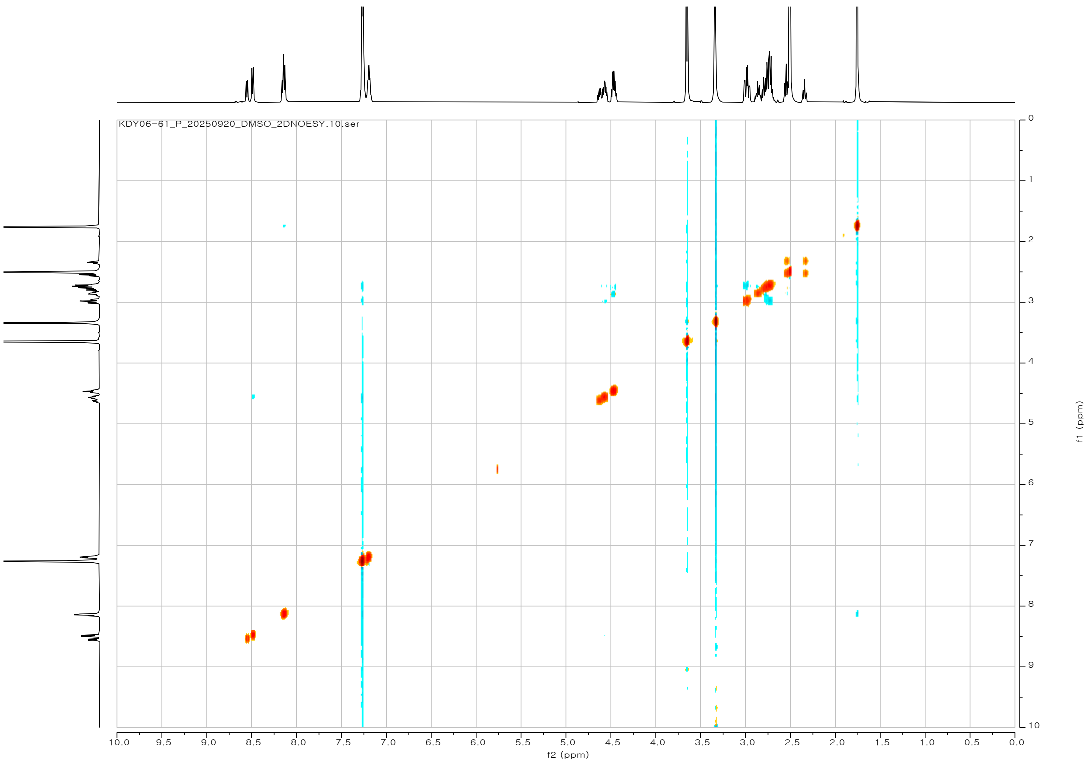

500 MHz, 2D NOESY NMR in C2D6SO

4-methoxy-N-(1-(2-phenylpyridin-4-yl)pentyl)benzamide (8).

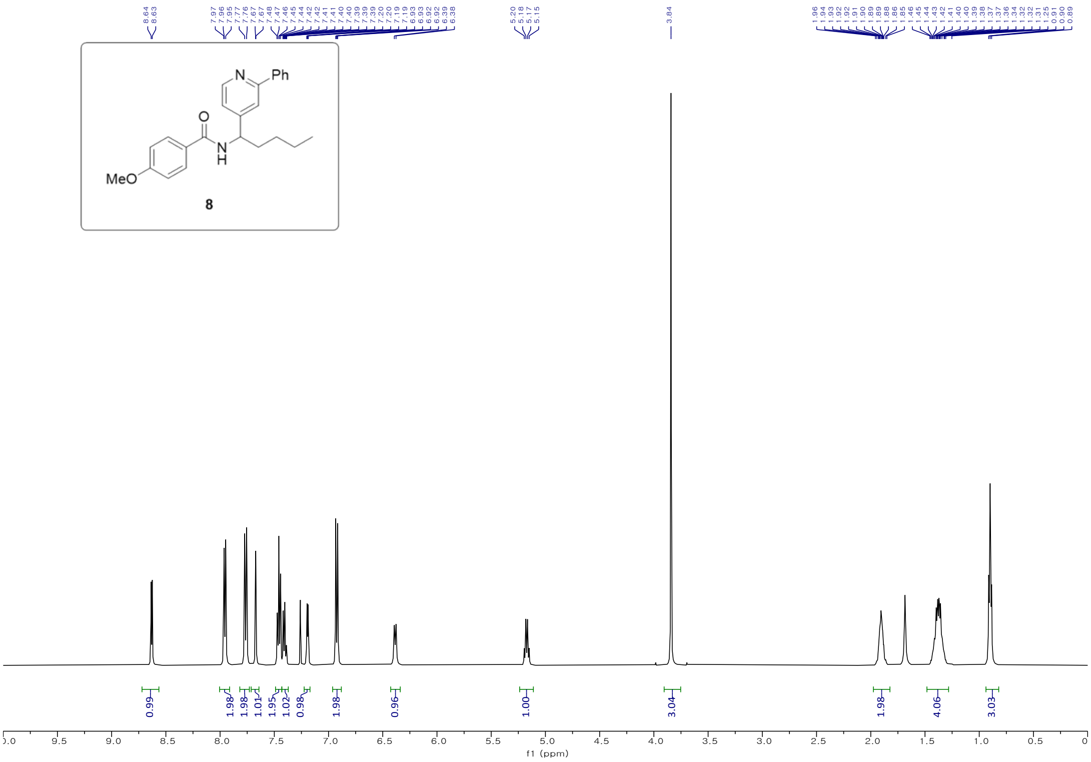

500 MHz, ^{1}H NMR in $CDCl_3$

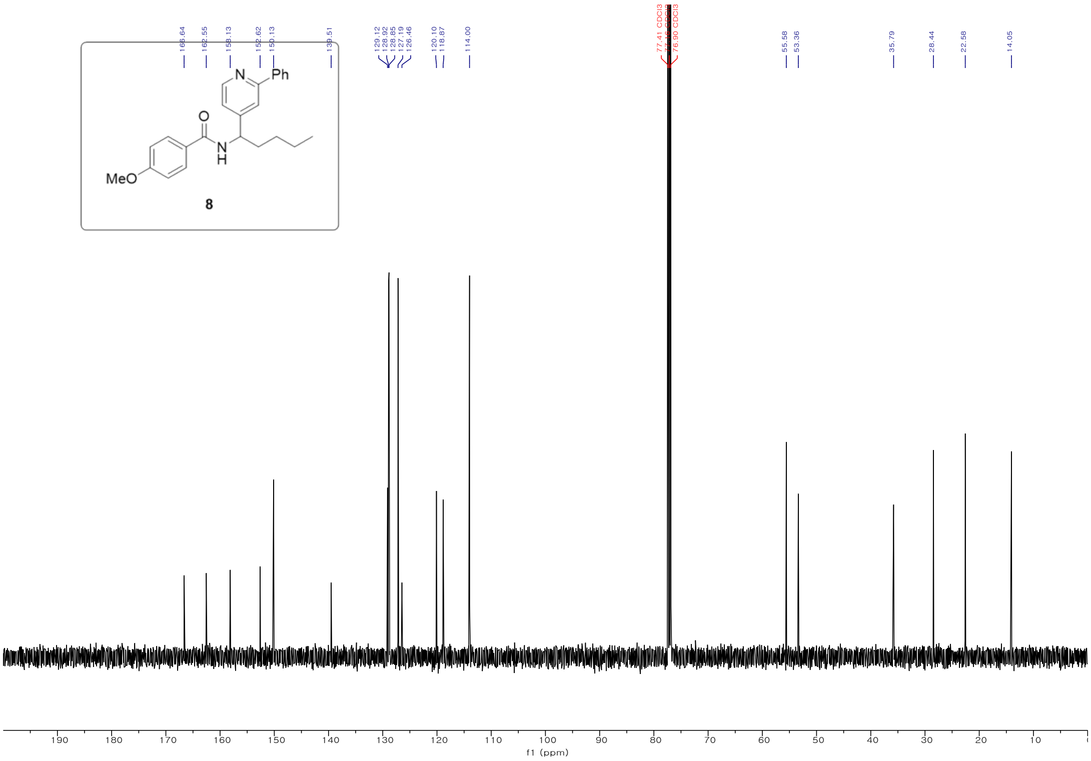

125 MHz, ^{13}C NMR in $CDCl_3$
